# Plasma Proteomic Signature as a Predictor of Age Advancement in People Living With HIV

**DOI:** 10.1111/acel.14468

**Published:** 2025-01-15

**Authors:** Adriana Navas, Vasiliki Matzaraki, Louise E. van Eekeren, Marc J. T. Blaauw, Albert L. Groenendijk, Wilhelm A. J. W. Vos, Maartje Jacobs‐Cleophas, Jéssica C. dos Santos, André J. A. M. van der Ven, Leo A. B. Joosten, Mihai G. Netea

**Affiliations:** ^1^ Department of Internal Medicine and Radboud Center of Infectious Diseases, Radboudumc Radboud University Nijmegen The Netherlands; ^2^ Department of Internal Medicine and Infectious Diseases Elizabeth‐Tweesteden Ziekenhuis Tilburg The Netherlands; ^3^ Department of Internal Medicine, ErasmusMC Erasmus University Rotterdam The Netherlands; ^4^ Department of Medical Microbiology and Infectious Diseases, ErasmusMC Erasmus University Rotterdam The Netherlands; ^5^ Department of Internal Medicine and Infectious Diseases OLVG Amsterdam The Netherlands; ^6^ Department of Medical Genetics Iuliu Hatieganu University of Medicine and Pharmacy Cluj‐Napoca Romania; ^7^ Department of Immunology and Metabolism, Life and Medical Sciences Institute University of Bonn Bonn Germany

**Keywords:** HIV, non‐AIDS‐related comorbidities, O‐link, premature aging, proteomics, senescence

## Abstract

Due to the increased burden of non‐AIDS‐related comorbidities in people living with HIV (PLHIV), identifying biomarkers and mechanisms underlying premature aging and the risk of developing age‐related comorbidities is a priority. Evidence suggests that the plasma proteome is an accurate source for measuring biological age and predicting age‐related clinical outcomes. To investigate whether PLHIV on antiretroviral therapy (ART) exhibit a premature aging phenotype, we profiled the plasma proteome of two independent cohorts of virally suppressed PLHIV (200HIV and 2000HIV) and one cohort of people without HIV (200FG) using O‐link technology. Next, we built a biological age‐prediction model and correlated age advancement (the deviation of the predicted age from the chronological age) with HIV‐related factors, comorbidities, and cytokines secreted by immune cells. We identified a common signature of 77 proteins associated with chronological age across all cohorts, most of which were involved in inflammatory and senescence‐related processes. PLHIV showed increased age advancement compared to people without HIV. In addition, age advancement in the 2000HIV cohort was positively associated with prior hepatitis C and cytomegalovirus (CMV) infections, non‐AIDS‐related comorbidities, ART duration, cumulative exposure to the protease inhibitor Ritonavir, as well as higher production of monocyte‐derived proinflammatory cytokines and chemokines and lower secretion of T‐cell derived cytokines. Our proteome‐based predictive model is a promising approach for calculating the age advancement in PLHIV. This will potentially allow for further characterization of the pathophysiological mechanisms linked to accelerated aging and enable monitoring the effectiveness of novel therapies aimed at reducing age‐related diseases in PLHIV.

## Graphical abstract

We developed a prediction model of biological age based on 77 plasma proteins that were associated with chronological age in two independent cohorts of PLHIV and one cohort of people without HIV. PLHIV exhibited an increased age advancement that was positively associated with previous co‐infections and non‐AIDS‐related comorbidities, a higher production of proinflammatory cytokines and chemokines, and a lower secretion of cytokines produced by T cells. The association of inflammatory age advancement with aging drivers indicates the applicability of this proteomic clock to further investigate the mechanisms of accelerated aging and explore therapeutic targets with the aim to ameliorate the high burden of non‐AIDS‐related comorbidities in PLHIV.

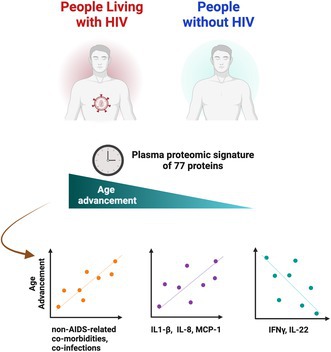

## 1 Introduction

People living with HIV (PLHIV) who are virally suppressed with anti‐retroviral treatment (ART) have improved life expectancy compared to untreated individuals. However, the increased risk and premature development of non‐AIDS‐related comorbidities (NARCs) remain present, presumably because of the systemic chronic inflammation caused by a persistent HIV infection, impaired gut integrity, and other factors (Bonnet et al. 2020; Deeks, Lewin, and Havlir 2013). The systemic chronic inflammation contributes to aging or senescence of the immune system (also termed “Inflammaging”); therefore, the low‐grade inflammation that is seen in PLHIV, even when using ART, may mirror a process of accelerated biological aging in these individuals (Breen et al. 2022; Horvath and Levine 2015).

While aging is accompanied by cellular senescence, organ dysfunction, and an increased risk for inflammatory‐mediated diseases (Li et al. 2023), chronological age is not synonymous with the biological age of an individual. Indeed, several biological clocks have been proposed to predict biological age and estimate age acceleration in humans (Belsky et al. 2015; Lohman et al. 2021), which, in turn, are more strongly associated with disease and health span. The most well‐known prediction models utilize DNA methylation to generate epigenetic clocks. For instance, GrimAge and PhenoAge outperform chronological age in predicting health status and co‐morbidities (Moqri et al. 2023). Epigenetic clocks can be used as a tool to evaluate the efficacy of age‐reversing interventions, as several pharmacological and environmental approaches influence epigenetic mechanisms related to biological aging (Duan et al. 2022; Galow and Peleg 2022).

Circulating proteins have been recently used as predictors of chronological age and comorbidities in cohorts of healthy individuals (Tanaka et al. 2020; Basisty et al. 2020), and they are of particular interest because reflect dynamic changes with aging and can be measured in samples of easy access, such as plasma. Several studies have been conducted to predict age in PLHIV using different biomarkers, suggesting that HIV infection enhances the inflammaging phenotype (Shiau et al. 2021; Esteban‐Cantos et al. 2022; de Armas et al. 2021). Given that many PLHIV, even when using ART, show persistent systemic inflammation and a high burden of non‐AIDS comorbidities, we hypothesized that PLHIV exhibit a premature aging phenotype reflected by age‐related changes in the plasma proteome. As part of this study, we aimed (1) to identify age‐associated plasma proteins in virally suppressed PLHIV and people without HIV, (2) to develop an age prediction model based on the plasma proteome, (3) to calculate inflammatory age advancement as a proxy of biological aging in both PLHIV and people without HIV, and (4) to investigate to which extent sociodemographic factors and HIV‐related parameters contribute to age advancement in PLHIV, and whether age advancement is associated with inflammation‐mediated comorbidities.

## 2 Materials and Methods

### 2.1 Study Design and Participants

The study investigated two independent cohorts of PLHIV, the 200HIV and 2000HIV cohorts, and one cohort of people without HIV the 200FG cohort, which are all part of the Human Functional Genomics Project (HFGP, http://www.humanfunctionalgenomics.org). The 200FG cohort comprises adults of Western European ancestry, older than 18 years old, enrolled in 2019. Table 1 provides the demographic characteristics of the volunteers from the three cohorts. For the proteomic profiling of the 2000HIV, we used measurements available in 641 participants out of 1895 virally suppressed PLHIV of the 2000HIV cohort, who had not been vaccinated against COVID‐19. The 2000HIV cohort comprises PLHIV of multiple ethnicities (European, African, Asian, Hispanic, Native American, and mixed ancestry) who were recruited in four HIV treatment centers (Radboudumc Nijmegen, Erasmus MC Rotterdam, and OLVG Amsterdam and Elisabeth‐TweeSteden Ziekenhuis Tilburg) between 2019 and 2022. Inclusion criteria were as follows: age > 18 years, HIV‐seropositive, receiving ART ≥ 6 months, and latest HIV‐RNA levels < 200 copies/mL. Exclusion criteria were absence of informed consent, detectable viral hepatitis B or C DNA by polymerase chain reaction (PCR), any current acute infection, and pregnancy. PLHIV from the 200HIV (*n* = 210) cohort were enrolled between December 2015 and February 2017 at Radboudumc and consisted of PLHIV > 18 years old who received ART for ≥ 6 months, had HIV‐RNA levels < 200 copies/mL, and were of European ancestry. Demographic and clinical characteristics and sample handling procedures were previously described elsewhere for the 200FG (Jaeger et al. 2019), 200 HIV (van der Heijden et al. 2021), and 2000HIV studies (Vos et al. 2022). The research was conducted following the principles of the Declaration of Helsinki and study protocols were approved by the Medical Ethical Review Committee Oost Nederland, Nijmegen, the Netherlands under the registration NL68056.091.81 [2000HIV], NL42561.091.12 [200HIV] and 2011‐399 [200FG]. All participants provided written informed consent. For the current study, we used samples collected from 100 volunteers of the 200FG cohort, 641 volunteers from the 2000HIV, and 210 volunteers from 200HIV cohort.

**TABLE 1:** Characteristics of study participants.

	2000HIV	200HIV	200FG	*p*	*N*
	*N* = 588	*N* = 205	*N* = 98		
Sex birth				< 0.001	891
Female	50 (8.50%)	16 (7.80%)	24 (24.5%)		
Male	538 (91.5%)	189 (92.2%)	74 (75.5%)		
Age	53.0 [45.0; 60.0]	52.5 [46.2; 59.4]	49.0 [36.9; 57.7]	0.003	891
BMI baseline (kg/m^2^)	25.0 [22.7; 27.4]	24.1 [22.0; 26.0]	—	0.001	790
Ethnicity					588
Unknown	1 (0.17%)	—	—		
Asian	21 (3.57%)	—	—		
Black	42 (7.14%)	—	—		
Hispanic	10 (1.70%)	—	—		
Mixed	25 (4.25%)	—	—		
Native American	2 (0.34%)	—	—		
White	487 (82.8%)	—	—		
HIV duration (years)	11.8 [7.01; 19.0]	8.47 [5.02; 14.1]	—	< 0.001	793
Current smoking				1.000	793
Non‐smoker	420 (71.4%)	147 (71.7%)	—		
Smoker	168 (28.6%)	58 (28.3%)	—		
CD4 nadir (10^9^ cells/mL)	0.26 [0.14; 0.38]	0.25 [0.14; 0.36]	—	0.441	784
Viral load zenith (copies/mL)	100,000 [36,121; 299,006]	100,000 [54,590; 400,000]	—	0.031	729
Art duration (years)	9.64 [5.73; 15.9]	6.60 [4.16; 11.7]	—	< 0.001	793
NRTI class				0.630	793
No	17 (2.89%)	8 (3.90%)	—		
Yes	571 (97.1%)	197 (96.1%)	—		
NNRTI class				0.013	793
No	351 (59.7%)	143 (69.8%)	—		
Yes	237 (40.3%)	62 (30.2%)	—		
PI class				0.043	793
No	536 (91.2%)	176 (85.9%)	—		
Yes	52 (8.84%)	29 (14.1%)	—		
INSTI class				0.008	793
No	262 (44.6%)	69 (33.7%)	—		
Yes	326 (55.4%)	136 (66.3%)	—		
CD4 latest (10^9^ cells/mL)	0.70 [0.52; 0.92]	0.66 [0.48; 0.80]	—	0.011	792
CD8 latest (10^9^ cells/mL)	0.96 (0.46)	—	—		474
Type 2 diabetes				0.333	793
No	565 (96.1%)	193 (94.1%)	—		
Yes	23 (3.91%)	12 (5.85%)	—		
Hypertension				0.465	793
No	460 (78.2%)	166 (81.0%)	—		
Yes	128 (21.8%)	39 (19.0%)	—		
Myocardial infarction				0.243	793
No	569 (96.8%)	194 (94.6%)	—		
Yes	19 (3.23%)	11 (5.37%)	—		
Stroke				1.000	793
No	573 (97.4%)	200 (97.6%)	—		
Yes	15 (2.55%)	5 (2.44%)	—		
Central nervous disease				0.493	793
No	531 (90.3%)	181 (88.3%)	—		
Yes	57 (9.69%)	24 (11.7%)	—		
Hepatitis C					588
No	545 (92.7%)	—	—		
Yes	43 (7.31%)	—	—		
Syphilis					588
No	295 (50.2%)	—	—		
Yes	293 (49.8%)	—	—		
IMT (intima‐media thickness)	0.69 (0.14)	—	—		523
Plaque unilateral					523
No	320 (61.2%)	—	—		
Yes	203 (38.8%)	—	—		
Plaque bilateral					523
No	456 (87.2%)	—	—		
Yes	67 (12.8%)	—	—		
Metabolic syndrome					588
No	442 (75.2%)	—	—		
Yes	146 (24.8%)	—	—		
LSM (liver stiffness measurement)	4.96 (1.89)	—	—		312
CAP (control attenuation parameter)	253 (53.5)	—	—		314
Liver steatosis					588
No data	274 (46.6%)	—	—		
S0	159 (27.0%)	—	—		
S1 or higher	155 (26.4%)	—	—		
Liver fibrosis					588
No data	276 (46.9%)	—	—		
F0–F1	284 (48.3%)	—	—		
F2 or higher	28 (4.76%)	—	—		
CMV IgG serology					586
Negative	26 (4.44%)	—	—		
Positive	560 (95.6%)	—	—		

*Note:* Data are shown as a median (IQR) unless stated otherwise. Data were analyzed using Wilcoxon unpaired test and Chi‐square (*χ* ^2^) test where applicable. CENTRAL_NERVOUS_DISEASE: included, multiple sclerosis, epilepsy, Alzheimer, Parkinson.

Abbreviations: CAP, control attenuation parameter; CMV, cytomegalovirus infection (IU/mL); IMT, intima medium thickness measurement (IMT); INSTI, integrase inhibitors; LSM, liver stiffness measurement: as proxy of liver steatosis; NNRTI, non‐nucleoside reverse transcriptase inhibitor; NRTI, nucleoside reverse transcriptase inhibitor; PI, protease inhibitors.

### 2.2 Proteomic Profiling of Circulating Plasma Proteins

Collection, processing, and storage of the cohorts' blood samples were performed according to study protocols from the HFGP (Netea et al. 2016). Venous whole‐blood samples were collected using EDTA tubes and centrifuged into plasma before being stored at −80°C. For the 2000HIV study participants, blood was collected during the baseline study visit and shipped to Laboratory of Experimental Internal Medicine, Radboudumc, Nijmegen, overnight at room temperature.

Proteomic profiling was conducted in plasma samples using a proximity extension assay coupled with next‐generation sequencing as a readout method by Olink Proteomics AB (Uppsala Sweden) (Assarsson et al. 2014). The relative abundance of plasma proteins was measured using the library Olink Explore 1536 platform consisting of 1472 proteins divided into four 384‐well multiplex panels focused on inflammation, oncology, cardiometabolic, and neurology protein. Protein measurements are presented as Normalized Protein Expression (NPX) values, which is Olink's relative protein quantification unit on log2 scale. Standard quality control (QC) per protein and sample was performed prior to statistical data analysis. In each of the four panels from the Olink Explore 1536 platform, IL‐6, TNF, and CXCL8 were measured as technical duplicates for quality control purposes. Strong correlations (Spearman rho correlation *r* > 0.9) were observed between the technical duplicates among panels, and therefore, we selected the measurements from the inflammatory panel. Next, we excluded proteins with LOD ≥ 25 of the samples, resulting in 1306 proteins in the 2000HIV and 200FG cohort, and 1293 in the 200HIV cohort for follow‐up analysis. During QC per sample, we performed principal component analysis (PCA) using the NPX values. Samples were defined as outliers when falling above or below four standard deviations (SD) from the mean of principal component one (PC1) and/or two (PC2). After removing outliers, 634, 98, and 205 samples remain in the 2000HIV, 200FG, and 200HIV cohorts, respectively. Moreover, to avoid confounding effect of COVID‐19 infection, 13 COVID‐19 positive individuals were removed from the 2000HIV. The overview of QC process is depicted in Figure S1.

### 2.3 Cytokine Production Capacity Upon Ex Vivo Stimulation of Peripheral Blood Mononuclear Cells (PBMCs)

PBMCs were seeded in round‐bottom 96‐well plates at 0.5 × 10^6^ cells/well in 0.2 mL of RPMI Dutch modified (Life Technologies) supplemented with Gentamycin 5 mg/mL (Centrafarm), Pyruvate 1 mM, and GlutaMAX 2 mM (Life Technologies). Cells were stimulated for either 24 h or 7 days at 37°C and 5% CO_2_ with different bacterial, fungal, and viral stimuli (Table S1). In 7 days culture, the medium was supplemented with 10% human pool serum. Supernatants were collected and stored at −80°C until used for ELISA measurements. Concentrations of IL‐1β, IL‐6, IL‐8, MCP1, and MIP1‐a were determined in the supernatants of the 24‐h PBMCs and IFNγ, IL‐5, IL‐10, IL‐17, and IL‐22 in the supernatants of the 7‐day PBMCs, using commercial ELISA kits (Duoset ELISA, R&D Systems, catalog numbers: Table S1). QC was performed as follows: for 24‐h cytokines, samples from 1814 participants were measured, of which 42 samples were excluded for being contaminated due to cytokine production in the RPMI well (negative control). RPMI‐contaminated well was defined as having concentrations of above 2× lower limit of detection (LLOD) in two out of the three cytokines: TNF, IL‐1β, or IL‐6. Moreover, based on PCA analysis, samples falling above or below four standard deviations (SD) from the mean of principal component one (PC1) and/or two (PC2) were removed as outliers. After QC, measurements from a total of 1760 participants remained with 35 cytokine‐stimulus measurements. For 7‐day cytokines, samples from 1816 participants were measured, and after exclusion of samples due to RPMI contamination and PCA outliers, 1754 remained with 30 cytokine‐stimulus measurements. For this study, we used 551 samples (24 h) and 550 samples (7 days), for which both ex vivo cytokine production and plasma proteomic data were available.

### 2.4 Association of Protein Relative Abundance With Chronological Age

The association of each NPX protein value with chronological age was determined using a linear regression model adjusted by sex in the 200FG (*n* = 98 samples) and 200HIV (*n* = 205 samples) cohorts. In the 2000HIV cohort, the linear model was adjusted by sex, ethnicity, inclusion center, and the time elapsed between patient's phlebotomy and blood sample processing in the laboratory. The top five principal components (PCs) extracted from the genotype of the individuals for which we had both genotype and plasma proteomic data (*n* = 588 samples) were included in the model for correction of ethnicity. To account for multiple testing, correction for the false discovery rate (FDR) was applied using the Benjamini‐Hochberg method and proteins with adjusted *p*‐value (FDR) < 0.05 were considered significantly associated with chronological age. A meta‐analysis using the summary statistics of the proteins associated with age in the three cohorts was performed using a random effect model to calculate the summary fold change over the three cohorts using a summary *p* value that represents the probability that the summary fold‐change is not different than zero as implemented in the MetavolcanoR package (Prada 2020). Overall, 77 proteins consistently associated with age in all the three cohorts in the same direction were included in the plasma proteome‐based prediction model (*p*_meta‐analysis_ < 0.05).

### 2.5 Plasma Proteomic‐Based Age Prediction Model

To construct a plasma proteomic‐based age prediction model, we initially examined three penalized regression models, Ridge, Lasso, and Elastic Net, using the NPX values of the 77 proteins associated with age from the 200FG cohort. The 200FG data were randomly split into a training set (80% for building a predictive model) and a test set (20% for evaluating the model). Hyperparameter tuning was performed for each model and RMSE (root mean square error) was calculated using the train and test dataset. Each hyperparameter combination (alpha, lambda) was evaluated using a 10‐fold cross‐validation to calculate the average performance of each model. Lasso regression was selected as the best model as it minimized the median RMSE compared to ridge and elastic net in the 10‐fold cross‐validation (median RMSE = 5.99 in Lasso versus 7.65 and 6.11 in Ridge and Elastic Net, respectively). When using Lasso, the following hyperparameters were set up on the training data set: alpha = 1; “lamda.min” as the shrinkage variable estimated after 10‐fold cross‐validation = 0.6086. We computed variable importance using varImp() to understand which proteins are the best predictors in the model. The varImp function tracks the changes in model statistics for each predictor and accumulates the reduction in the statistic when each predictor's feature is added to the model. This total reduction is used as the variable importance measure. Relative variable importance values range from 0% to 100%. The most important variable always has a relative importance of 100%. Finally, a predicted proteomic age was calculated for 205 and 588 PLHIV from the 200HIV and 2000HIV cohort, respectively, as a linear combination of the regression coefficients of 77 proteins from the Lasso regression model. For computing penalized linear regression models, glmnet package was used in R. Finally, model training and evaluation were performed using caret package in R (4.1.2).

### 2.6 Statistical Analysis

Age advancement, as a proxy of accelerated aging, was calculated as the difference between proteomic‐based predicted age and chronological age, as previously described (De Francesco et al. 2019). Age advancement in each cohort is reported as a median and evaluated for statistical significance using Wilcoxon signed‐rank tests. We calculated age advancement as a proxy of accelerated aging, with values above zero indicating a higher proteomic age compared to chronological age.

#### 2.6.1 Association of Age Advancement With Clinical Parameters

A multiple linear regression model adjusted for chronological age was performed using demographics, HIV‐related and clinical variables as predictors (Table S2), and age advancement as outcome. The following regression formulas were used:

(1) Ageadvancement~variable of interest+chronologicalage

(2) $$\begin{array}{c} \mathrm{Age}\;\text{advancement}\sim \text{variable of interest}\\ +\text{chronological}\;\mathrm{age}+\mathrm{CVD}\;\text{risk factors} \end{array}$$

#### 2.6.2 Association of Age Advancement With Years of Cumulative Exposure to Each ART Medication

Exposure to antiretroviral medication was assessed as years of cumulative exposure to each ART medication. Association between age advancement and cumulative drug exposure was calculated in PLHIV who ever received the medication. The cumulative exposure to each ART medication did not follow normal distribution (Figure S2A). Data was inverse rank transformed (Figure S2B). The following formula was used for the linear association analysis:

(3) Ageadvancement~years_cum_type ofART+chronologicalage

#### 2.6.3 Association of Age Advancement With Ex Vivo Cytokine Capacity

Combinations of cytokine‐stimuli with low detectability (< 50%) were excluded from the analysis as well as cytokines with bimodal distribution. Data was transformed using the rank‐based inverse method before analysis (Figures S3–S6). Only cytokines with normal distribution after transformation were used for the analysis. The following formula was used for the linear regression analysis:

(4) Ageadvancement~cytokine+chronologicalage+sexbirth

For all associations, correction for the false discovery rate (FDR) was applied using Benjamini‐Hochberg method, and a significant association with age advancement was defined when adjusted *p* value < 0.05. All statistical analyses and visualizations were performed using R studio.

### 2.7 Role of the Funding Source

The funders of the study had no role in study design, data collection, data analysis, data interpretation, or writing of the report.

## 3 Results

### 3.1 Baseline Clinical Characteristics of the Cohorts

In this cross‐sectional study, we assessed the plasma proteome of 588 participants from the 2000HIV cohort (median age: 53 years, [IQR 45–60]; 91.5% male [*n* = 538] 8.5% female [*n* = 50]), 205 participants of the 200HIV cohort (median age: 52.5 years, [IQR 46.2–59.4]; 92.2% male [*n* = 189] 7.8% female [*n* = 16]), and 98 participants without HIV from the 200FG cohort (median age: 49.5 years, [IQR 36.9–57.7]; 75.5% male [*n* = 74] 24.5% female [*n* = 24]). Participants from the 2000HIV cohort had a slightly higher BMI (median: 25, IQR [IQR 22.7–27.4]), HIV duration (median: 11.8 [IQR 7–19]) and ART duration (median: 9.64, [IQR 5.73–15.9]), CD4 nadir (median: 0.26, [IQR 0.14–0.38]), and CD4 latest (median: 0.70 [IQR 0.52–0.92]), compared to participants from the 200HIV cohort (*p* < 0.05). No significant differences were found for the presence of comorbidities between the two PLHIV cohorts. General participant characteristics are described in Table 1.

### 3.2 Identification of Plasma Proteins That Predicts Biological Age in People Without HIV and PLHIV

**FIGURE 1:**
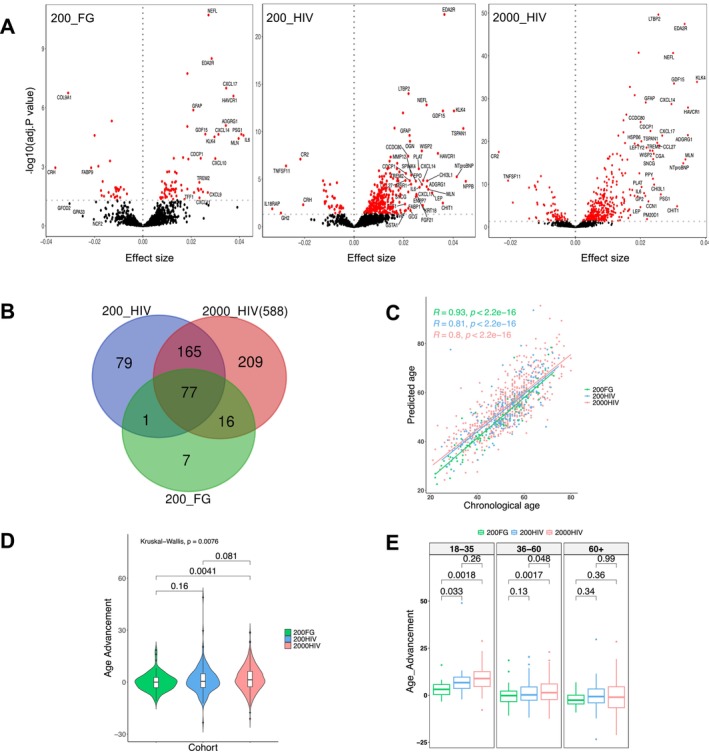
Proteomic age signature in people without HIV and PLHIV. (A) Volcano plot displaying the association of plasma proteins with chronological age in three independent cohorts: Left (200FG), middle: (200HIV), and right (2000HIV), respectively. (B) Venn diagram showing the comparison of the significant age‐associated proteins between the three cohorts and the signature of 77 proteins common to all cohorts. (C) Correlation of chronological age and predicted age (calculated using a lasso regression model) in the three cohorts evaluated. (D) Age advancement comparison (predicted age − chronological age) between the three cohorts. (E) Differences in age advancement across the three chronological age strata: Young (18–35 years old), middle age (36–60 years old), and older (60+). Statistical significance in (D) and (E) was estimated by Kruskal–Wallis and Wilcoxon unpaired test.

To investigate which plasma proteins were associated with chronological age and whether PLHIV exhibit a premature aging phenotype, we first assessed the association between the plasma protein concentrations with chronological age in the 200FG, 200HIV, and 2000HIV cohorts. Of the total proteins evaluated, chronological age was significantly associated with 101, 322, and 467 proteins (FDR < 0.05) in the 200FG, 200HIV, and 2000HIV cohorts, respectively (Figure 1A, Tables S3–S5). To identify proteins consistently linked to age, we next performed a meta‐analysis of the proteins significantly associated with age (FDR < 0.05) across all three cohorts. There were 77 proteins that reached statistical significance (*p* < 0.05) in the meta‐analysis (Figure 1B and Table S6) demonstrating their association with age. Out of the 77 proteins identified, nine proteins (TGFB1, SCARB2, NEFL, LTBP2, IGFBP4, GDF15, EFEMP1, DCN, and ColA1) were described as components of the Senescence Associated Secretory Phenotype (SASP) factors according to the database of the secretomes of senescence cells: SASP Atlas (Basisty et al. 2020).

To evaluate whether the plasma proteome could predict chronological age, a prediction model using lasso regression was developed using the common 77 protein‐signature associated with age. The predicted and chronological age showed a strong positive correlation (*r* = 0.93, *p* < 2.2e−16) in the 200FG cohort, indicating that the identified plasma proteins are a strong predictor of age.  This positive correlation was validated independently in the other two cohorts, with correlation coefficients between predicted and chronological age of 0.81 and 0.80 in the 200HIV and 2000HIV cohort, respectively (*p* < 2.2e−16 Figure 1C). In addition, lasso regression allowed to select the best predictor proteins based on their importance score (Figure S7 and Table S7). The following proteins were the top ten predictors with an importance score ranging between 40% and 100%: PODXL2, TNXB, COL1A1, GFAP, WNT9A, RET, SMOC1, BC, CTSV, and WISP2 (see Table 2). Among our top ten predictors for aging, PODXL2 was the most important predictor identified by the model.

**TABLE 2:** Top 10 important predictors of proteomic age in people without HIV and PLHIV.

Protein	Target full name	Uniprot	Association with chronological age	Expression	Function
PODXL2	Podocalyxin like 2	Q9NZ53	↓	T‐cells, B‐cells (memory and germinal), monocytes, endothelial cells, and CD34^+^ bone marrow cells. Highly expressed in brain	Cell adhesion. Mediates rapid rolling of leukocytes over vascular surfaces
TNXB	Tenascin xb	P22105	↓	Highly expressed in fetal adrenal, fetal testis, fetal smooth, striated, and cardiac muscle	Mediate interactions between cells and the extracellular matrix
COL1A1	Collagen alpha‐1(I) chain	P02452	↓	Fibrils of tendon, ligaments, and bones	SASP protein
GFAP	Glial fibrillary acidic protein	P14136	↑	Uniquely found in astrocytes in the CNS, non‐myelinating Schwann cells in the PNS, and enteric glial cells	Maintenance of activated astroglial cell status (astrogliosis) following nervous system injury. GFAP protein and its breakdown products are rapidly released into biofluids, making them strong candidate biomarkers for such neurological disorders
WNT9A	Wnt family member 9a	O14904	↑	Heart, endometrium	Ligand for members of the frizzled family of seven transmembrane receptors. Functions in the canonical Wnt/beta‐catenin signaling pathway. Required for normal chondrocyte maturation and for normal bone mineralization during embryonic bone development. Plays a redundant role in maintaining joint integrity
RET	Proto‐oncogene tyrosine‐protein kinase receptor Ret	P07949	↓	(monocytes and Natural Killer cells) and adaptive (B lymphocytes, CD4^+^ and CD8^+^ T cells)	Involved in numerous cellular mechanisms including cell proliferation, neuronal navigation, cell migration, and cell differentiation upon binding with glial cell‐derived neurotrophic factor family ligands
SMOC1	SPARC‐related modular calcium‐binding protein 1	Q9H4F8	↑	Cytoplasmic expression in testis, plasmacytoid DC	Plays essential roles in both eye and limb development. Probable regulator of osteoblast differentiation
BOC	Brother of CDO	Q9BWV1	↓	Skeletal muscle, heart, thymus, kidney, and small intestine	Promotes differentiation of myogenic cells
CTSV	Cathepsin L2	O60911	↓	Predominantly expressed in the thymus and testis	A lysosomal cysteine protease with endopeptidase activity
WISP2	CCN family member 5/Wnt‐1‐inducible signaling pathway protein	O76076	↑	Adipose tissue, fibroblast	CCN5/WISP2 is a matricellular protein, the expression of which is regulated by Wnt signaling and IGF‐1. CCN5 is a regulator of adipocyte proliferation and maturation, affecting lean/fat mass ratio and insulin sensitivity

Next, we calculated the age advancement, and we found that PLHIV from the 2000HIV cohort showed a significant increase in age advancement (median = 1.37, IQR = 8.91) compared to 200FG people without HIV (median = 0.045, IQR = 5.95, *p* = 0.0041). While the difference between the 200HIV and 200FG cohorts did not reach statistical significance (Figure 1D), this may be explained by the smaller sample size of the 200HIV compared to the 2000HIV cohort. In addition, differences in age advancement were investigated across the three cohorts following chronological age strata: young (18–35 years), middle age (36–60 years), and older (60+ years) individuals. Young PLHIV showed increased age advancement compared to people without HIV (2000HIV: median = 8.85, IQR = 7.94, *p* = 0.0041; 200HIV: median: 6.68, IQR = 6.02, *p* = 0.033). In PLHIV from 2000HIV cohort, a similar behavior was observed in the middle age group (2000HIV: median = 1.36, IQR = 8.27, *p* = 0.0017; 200HIV: median = 0.19, IQR = 7.05, *p* = 0.13). However, no significant differences were observed within the older age group (2000HIV: median = −1.02, IQR = 11.09, *p* = 0.36; 200HIV: median = −0.71, IQR = 7.38, *p* = 0.99) (Figure 1E).

### 3.3 Association of Age Advancement With Demographics, HIV‐ and ART‐Related Parameters and Comorbidities in PLHIV

**FIGURE 2:**
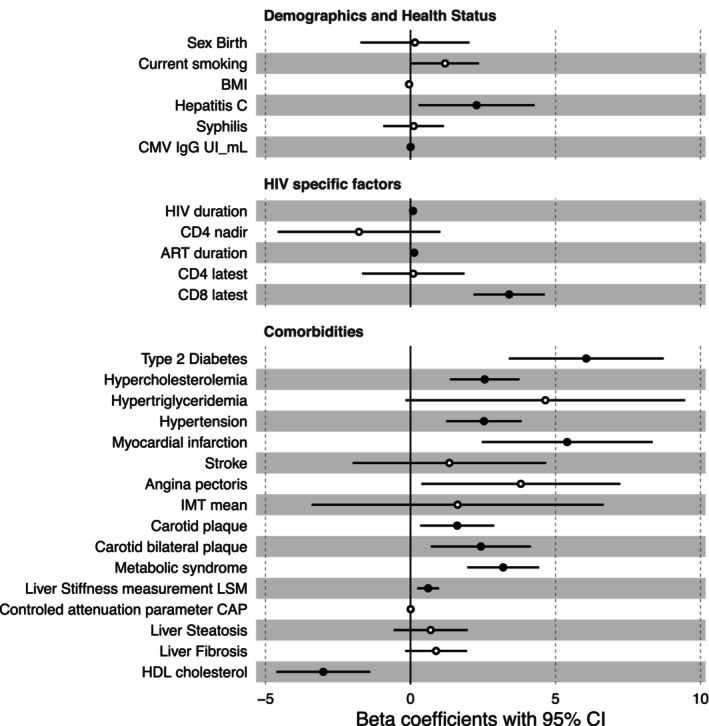
Forest plot displaying the associations between age advancement and demographics, HIV‐specific factors and comorbidities in PLHIV. For each of the associations between age advancement and clinical parameters, the standardized beta and the confidence interval derived from linear models were plotted. The significant association (FDR < 0.05) is shown with a black filled dot.

Next, to evaluate whether the calculated inflammatory age advancement is reflecting biological aging in PLHIV, we examined its association with various demographic, healthy status, and HIV‐specific factors, as well as age‐related comorbidities in the 2000HIV cohort. Age advancement was not correlated with sex, current smoking, BMI, co‐infection with syphilis, CD4 nadir, or last CD4 counts (Figure 2). Prior viral co‐infections, such as hepatitis C (HepC) and CMV (IgG titers), were positively correlated with age advancement, as well as HIV duration, ART duration, and the latest measurement of CD8 T‐cell count. Importantly, age advancement was positively correlated with cardiovascular and metabolic comorbidities including hypertension, myocardial infarction, presence of plaques, angina pectoris, hypertriglyceridemia, type 2 diabetes mellitus (T2DM), metabolic syndrome, and liver stiffness measurement (used as liver fibrosis proxy) (Figure 2). An accelerated age advancement was, on the other hand, negatively correlated with HDL cholesterol concentrations (FDR < 0.05) (Figure 2 and Table S8).

To test whether the associations between inflammatory age advancement and the above‐mentioned factors were confounded by traditional cardiovascular risk factors, we used a linear regression model using chronological age, sex, T2DM, hypercholesterolemia, hypertriglyceridemia, and hypertension as confounders. Age advancement remained significantly correlated with prior viral co‐infections (HepC and CMV), higher CD8‐T cell count, and lower HDL levels (FDR < 0.05) (Figure S8 and Table S9).

Since myocardial infarction and T2DM were the comorbidities that exhibited the strongest association with age advancement, we compared the relative concentrations of GFAP, SMOC1, WNT9A, and WISP2 (identified between the top ten predictors as the ones upregulated with chronological age) within participants from the 2000HIV cohort stratified based on the presence or absence of these comorbidities. The concentration of WNT9A and SMOC1 was significantly higher only in PLHIV with faster age advancement having at least one of the evaluated comorbidities. However, no significant differences were observed for these two proteins among participants with lower age advancement. Notably, this pattern was not observed for GFAP and WISP2 proteins (Figure S9).

**FIGURE 3:**
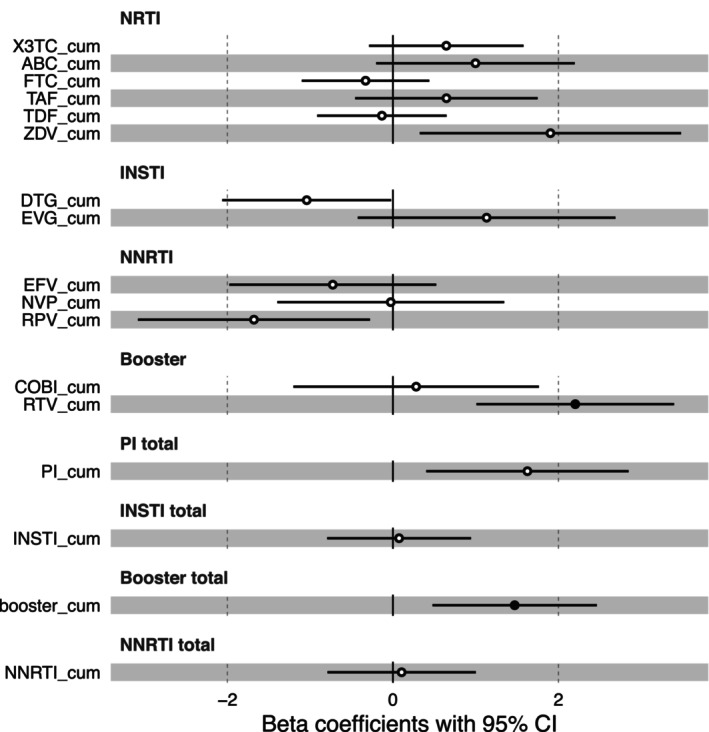
Forest plot of associations between age advancement and cumulative antiretroviral exposure in PLHIV. For each of the associations between age advancement and years of cumulative exposure to each ART medication, the standardized beta and the confidence interval derived from linear models were plotted. The model was corrected by age. The significant associations (FDR < 0.05) are shown with a black filled dot. 3TC, lamivudine; ABC, Abacavir; booster, booster drugs; COBI, Cobicistat; DTG, Dolutegravir; EFC, efavirenz; EVG, elvitegravir; FTC, Emtricitabine; INSTI, Integrase inhibitors; NNRTI, non‐nucleoside reverse transcriptase inhibitor; NVP, nevirapine; PI, protease inhibitors; RPV, rilpivirine; RTV, ritonavir; TAF, tenofovir alafenamide; TDF, tenofovir disproxil; ZDV, Zidovudine.

Besides comorbidities and co‐infections, ART usage can contribute to accelerated aging in PLHIV and, therefore, we investigated the association of age advancement with cumulative exposure to antiretroviral drugs. We found that cumulative exposure to the protease inhibitor ritonavir (RTV_cum) was positively correlated with an increased age advancement (FDR < 0.05). (Figure 3 and Table S10). Although not statistically significant after multiple testing correction, we observed a significant negative association (*p*‐value < 0.05) between age advancement and cumulative exposure to Dolutegravir and Rilpivirine. Additionally, a positive association was found with Zidovudine and total cumulative exposure to protease inhibitors.

### 3.4 Association of Age Advancement With Ex Vivo Cytokine Production

**FIGURE 4:**
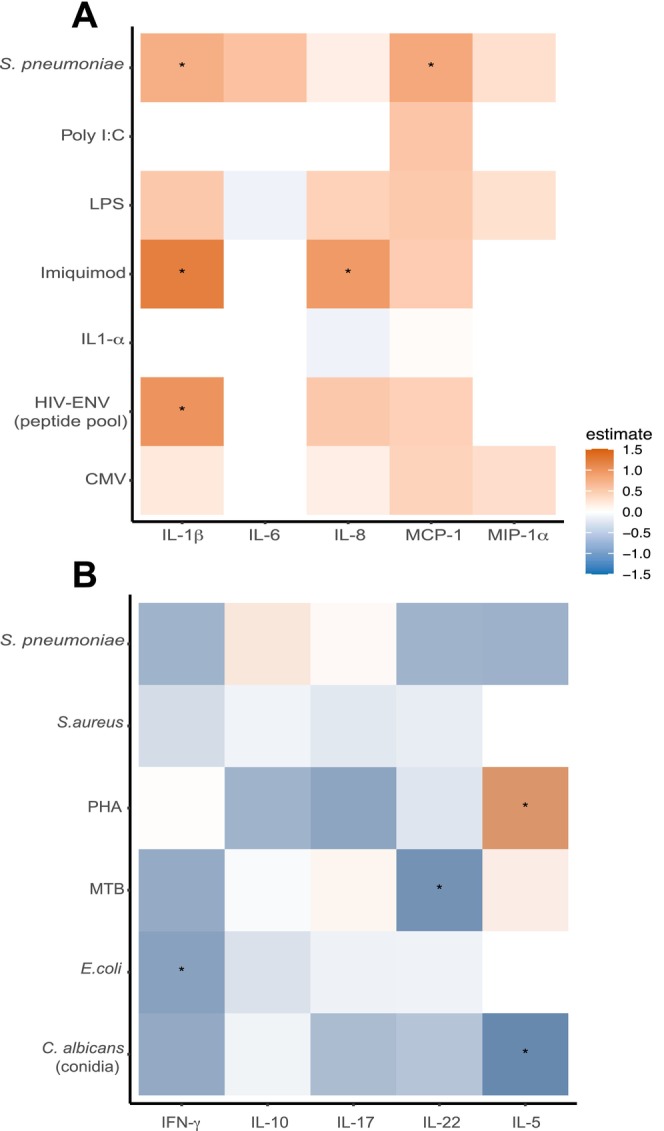
Heat map showing the association between (A) PBMC‐secreted SASP‐cytokines and (B) T cell cytokines with age advancement in PLHIV. (A) PBMCs from PLHIV of the 2000HIV cohort (*n* = 551) were stimulated with the TLR ligands Poly:IC, IMQ, LPS, the peptides HIV ENV pool and pp65 CMV, *S. pneumoniae* and rhIL‐1a for 24 h and cytokines (IL‐1β, IL‐6, IL‐8), and chemokines (MCP‐1, MIP‐1α) were measured by ELISA. (B) PBMCs from PLHIV of the 2000HIV cohort (*n* = 550) were stimulated for 7 days with *C. albicans* conidia, *E. coli* , *M. tuberculosis* , PHA, *S. aureus* , *S. pneumoniae* , and the cytokines (IFNγ, IL‐10, IL‐17, IL‐22, IL‐5) were measured by ELISA. Heatmaps represent the estimate from linear regression model adjusted by age and sex. Only significant associations (FDR < 0.05) are shown (*).

To investigate the influence of age advancement on the functionality of immune cells in PLHIV, we assessed the secreted SASP‐cytokines in the supernatant of PBMC after exposure to various microbial and (non)‐microbial stimuli. Overall, a positive correlation was found between age advancement and a higher production of IL‐1β in response to HIV‐envelope peptide pool (HIV‐ENV), Imiquimod (IMQ), and *Streptococcus pneumoniae* , as well as IL‐8 in response to IMQ, and MCP1 in response to *S. pneumoniae* . A negative correlation was found between age advancement and the following cytokines produced by T cells: IL‐5 in response to *Candida albicans* conidia, IFNγ in response to *E. coli* , and IL‐22 in response to *Mycobacterium tuberculosis* (Figure 4, Figure S10, Tables S11 and S12).

## 4 Discussion

In this study, we developed a prediction model of biological age based on 77 plasma proteins that were consistently associated with chronological age in two cohorts of PLHIV and one cohort of people without HIV. Many of these proteins are biomarkers of systemic inflammation, and we propose that this prediction model mirrors the impact of inflammation on biological age. We found that PLHIV exhibited an increased age advancement that was positively associated with prior infections, including hepatitis C and CMV, duration of HIV infection, and ART usage, as well as with non‐AIDS comorbidities. These findings indicated the presence of accelerated biological aging in PLHIV, of which inflammation plays a significant role. Of note, we did not observe any significant difference in age advancement when participants were stratified in groups based on HIV duration (Figure S11).

The presence of accelerated aging in PLHIV is consistent with previous studies that have shown an increased age acceleration in PLHIV based on epigenetic age clocks (Esteban‐Cantos et al. 2021) and immune cell composition (de Armas et al. 2021). These studies also concluded that PLHIV experiencing accelerated aging are predisposed to infections, cardiovascular diseases, neurodegenerative diseases, and cancer. We observed that the accelerated inflammatory aging in our PLHIV cohort was most clearly present in young individuals, while being less accentuated in older people. The precise mechanisms behind this difference are unknown, but it suggests that chronic HIV infection leads to a quicker kinetics toward an inflammatory status associated with older age. Conversely, the elderly population has already a proinflammatory status established, and the effects of HIV infection are less obvious. Previous studies using telomere length assays, as an aging proxy, have shown premature biological aging in children and young adults with HIV. This premature aging was characterized by heightened immune activation and associated with telomere shortening (Alcaraz et al. 2021; Dalzini et al. 2021, 2020).

An inflammatory‐proteomic‐based age model has one important advantage compared to the earlier developed epigenetic and immune cells aging models: while all these scores mirror a certain aspect of biological aging, the inflammatory aging models can also suggest therapeutic targets by identifying proteins and pathways important for aging. Our predictive model relied on an age‐associated signature of 77 plasma proteins, most of which have been previously reported as predictors of age in healthy cohorts. During aging, dysregulated function in senescent cells leads to the release of inflammatory mediators, known as the senescence‐associated secretory phenotype (SASP) (Schafer et al. 2020). Our study found that nine out of the 77 proteins identified are SASP mediators, confirming their role in age‐related processes across all cohorts. Among them, GDF15 has been identified as a potential biomarker of aging and is associated with the presence of multiple chronic diseases (Liu et al. 2021). Additionally, two proteins from our signature, CTSV and WISP2, were reported by Coenen et al. as part of their 15‐protein aging signature that accurately predicted individuals' age in four independent cohorts (Coenen et al. 2023). Proteins from our signature: ADGRG1, BOC, CDON, CTSV, PROK1, RET, and TNXB, which exhibited lower expression with age, play important roles in the remodeling of the extracellular matrix (Schuler et al. 2021). Consequently, this reduced expression might indicate a decline in tissue resilience and flexibility. Other biological age‐predictive models based on the plasma proteome and single‐cell RNA sequencing have captured alterations in the extracellular matrix related to early cognitive decline (Oh et al. 2023).

One of the most important age predictors we identified was podocalyxin‐like protein 2 (PODXL2), a ligand for vascular selectins, which mediates the rapid rolling of leukocytes over vascular surfaces (Kerr et al. 2008). A previous study demonstrated the critical role of Podxl2 in maintaining endothelial barrier integrity, as knockout mice exhibited increased vascular permeability and inflammation compared to the control group (Horrillo et al. 2016). Another study in humans investigated how DNA methylation patterns change with aging and identified CpG sites within the *PODXL2* among the 155 significantly associated with aging in an epigenome‐wide analysis. Specifically, certain CpG sites in the *PODXL2* region were newly discovered to have age‐associated DNA methylation patterns (Florath et al. 2014). Although PODXL2 has not been previously reported as an aging biomarker, our observation of reduced PODXL2 expression with increased biological aging, together with being an important predictor of age advancement, may suggest that this reduction reflects the deleterious vascular changes linked to aging.

In addition, the relative expression of four proteins (GFAP, WNT9A, SMOC1, and WISP2) positively correlated with age advancement. In our study, PLHIV with faster age advancement, and having either myocardial infarction or T2DM, showed higher levels of SMOC1 and WNT9A, indicating the involvement of these two proteins in age‐related processes in PLHIV. Interestingly, WNT9A and WISP2 are part of the Wnt‐signaling pathway, which is known to be involved in aging‐related biological processes and metabolic disorders (Gruber, Yee, and Tolwinski 2016). Recently, WNT9A and SMOC1 were proposed as biomarkers of dementia and Alzheimer's disease, respectively. For instance, higher expression of WNT9A has been negatively correlated with cognitive performance in patients with dementia (Ehtewish et al. 2023), and elevated SMOC1 concentrations were detected 30 years before the onset of Alzheimer's symptoms (Johnson et al. 2023). Likewise, a high concentration of WNT9A has been associated with renal fibrosis in human and mouse models, possibly by accelerating senescence in tubular epithelial cells (Luo et al. 2018). Altogether, these results suggest that both proteins might be explored as potential biomarkers for accelerated aging in PLHIV.

Our inflammatory‐proteomic‐based age model shows a strong association with several age‐related comorbidities. This demonstrates the relevance of the inflammatory age score for biological aging and the prediction of cardiometabolic complications, known to be one of the main causes of death in PLHIV (Croxford et al. 2017). In addition to the inflammatory effect of chronic HIV infection, accelerated aging in PLHIV has been proposed as a side effect of antiretroviral therapies. Our results demonstrated a positive correlation between age advancement and cumulative exposure to the protease inhibitor Ritonavir (RTV) and the NRTI zidovudine (ZDV). The negative impact of these two classes of ART on mitochondrial function has been extensively described (Smith et al. 2012). In vitro exposure to these medications leads to mitochondrial‐DNA damage, increased oxidative stress, accumulation of Prelamin A (a pro‐senescence protein), and cellular apoptosis (Caron et al. 2007; Kuehnemann et al. 2023). These dysfunctions are critical contributors to aging as they impair cellular metabolism and energy production, accelerating the aging pace in cells and tissues. In contrast to RTV and ZDV, we observed that exposure to Dolutegravir (DTG) and Rilpivirine (RPV) was negatively associated with age advancement. Previous studies have indicated a positive effect of switching to DTG/3TC or DTG/RPV regimens on immune cell composition, particularly with an increase in CD4‐T cell counts and a decrease in CD8‐T cell counts (Troya et al. 2023, 2022). Additionally, a case report has described that in an HIV‐positive participant, the exposure to Rilpivirine reversed T2D and improved lipid measurements along with body fat distribution (Ucciferri et al. 2019). These findings indicate that DTG and RPV might have a positive effect on the biological aging process in PLHIV. However, more studies are needed to address this hypothesis and decipher the underlying mechanisms.

Finally, the two main immune function changes associated with aging are inflammation and decreased responsiveness to stimulation, especially of lymphoid cells. In our study, increased age advancement in PLHIV was associated with both a higher production of proinflammatory cytokines and chemokines (IL‐1β, IL‐8, and MCP‐1) that drive low‐grade, age‐related inflammation (Rea et al. 2018; Mitchell et al. 2023) and a lower secretion of cytokines produced by T cells. Altogether, the findings of our predictive model capture primary characteristics of immunosenescence. Consequently, the inflammatory aging score we present here may serve as a valuable surrogate marker of immune aging, guiding interventions with senolytic or senomorphic compounds in PLHIV. While more mechanistic studies are needed to strengthen the arguments for specific therapies, a number of strategies could be envisioned. Modulation of the Wnt pathway through WNT9A, as well as SMOC1, two of the most important age predictors in our model, could represent a potential therapeutic target. The increase in biomarkers associated with an SASP phenotype also argues for an anti‐inflammatory approach to counteract the effects of immune aging.

There are also some limitations in the present study. First, this is a cross‐sectional study that by definition presents correlations, and is not able to demonstrate causation. The study did not assess age advancement within the same individuals through the time, and thus could not capture the changes in health span. Future longitudinal measurements of proteomic data would be more sensitive in capturing the pace of aging in PLHIV. Second, our cohorts are represented mainly by populations of European ancestry; therefore, our conclusions cannot be directly extrapolated to populations of non‐European ancestry. Consequently, it would be important to validate the age‐associated proteins identified here, in independent healthy and PLHIV cohorts from other ancestries. Third, the 2000HIV cohort is predominantly composed of males, in line with the demographics of PLHIV. While we did not find a significant association between age advancement and sex, we cannot entirely rule out the potential contribution of sex to premature aging in PLHIV. Thus, it is essential that future studies will extend sex‐stratified analyses in more PLHIV cohorts and people without HIV, to investigate sex‐biased differences in aging. Fourth, while we measured approximately 1300 plasma proteins, this is by no means a comprehensive list of all proteins in the plasma; thus, our results do not represent a complete aging proteome. Therefore, it would be interesting that future aging studies incorporate multiple biomarkers to track change, not only in plasma but also across different organ systems. Finally, because of the non‐linear dynamics of biological aging, findings in young people regarding biological age should be interpretated with caution, as previously discussed by Oh et al. (Oh et al. 2023). Therefore, the inclusion of a higher number of young participants would be necessary to study biological aging in younger populations.

Overall, we developed a proteomic‐based predictive model of age that showed a strong correlation with chronological age in cohorts of people without HIV and PLHIV. The association of inflammatory age advancement with age‐related diseases and aging drivers indicates the applicability of this proteomic clock to further identification of mechanisms of accelerated aging. In addition, it contributes to the exploration of therapeutic targets to ameliorate the high burden of non‐AIDS comorbidities in PLHIV. Finally, some of the protein predictors described in our study could potentially be explored as biomarkers of aging. However, further studies in a longitudinal setting are required for exploring the changes of aging over time.

## Author Contributions

Mihai G. Netea, Leo A. B. Joosten, André J. A. M. van der Ven, Vasiliki Matzaraki, and Adriana Navas contributed to study design. Louise E. van Eekeren, Marc J. T. Blaauw, Albert L. Groenendijk, Wilhelm A. J. W. Vos, Adriana Navas, Jéssica C. dos Santos, and Maartje Jacobs‐Cleophas performed the investigation. Adriana Navas and Vasiliki Matzaraki performed the formal analysis and drafted the manuscript. Mihai G. Netea, Leo A. B. Joosten, André J. A. M. van der Ven, Vasiliki Matzaraki, Adriana Navas, Louise E. van Eekeren, Marc J. T. Blaauw, Albert L. Groenendijk, Wilhelm A. J. W. Vos, Jéssica C. dos Santos, and Maartje Jacobs‐Cleophas revised and approved the manuscript. All authors had full access to all the data in the study, read and approved the final version of the manuscript, and had final responsibility for the decision to submit for publication.

## Conflicts of Interest

All authors are part of the 2000HIV collaboration, which is supported by ViiV Healthcare. MGN and LABJ are scientific founders of TTxD, Salvina, and Lemba. MGN is scientific founder of Biotrip.

## Supporting information


Data S1.

## Data Availability

Further information and request for data resources should be directed to 2000 HIV study principal investigators: Prof. Dr. André J. A. M. van der Ven and Prof. Dr. Mihai G. Netea.
