# Supplementary material for: Plasma Proteomic Signature as a Predictor of Age Advancement in People Living With HIV
Source: Aging Cell. 2025 Jan 15;24(5):e14468. doi: 10.1111/acel.14468 (PMC12073908; doi:10.1111/acel.14468)
Supplement: Supplementary file 1 — Data S1. [file ACEL-24-e14468-s001.docx]

# **Supplementary Figures**

## **Supplementary Figure 1. Overview of quality control of proteomics analyses, related to figure 1A**

Supplementary Figure 1: Overview of quality control of proteomics analyses, related to figure 1A. Standard quality control (QC) per sample and protein was performed prior to statistical data analysis (A). During QC per sample, we performed principal component analysis (PCA) using the NPX values. Samples were defined as outliers when falling above or below three standard deviations (SD) from the mean of principal component one (PC1) and/or two (PC2). After removing outliers, 634, 98, and 205 samples remain in the 2000HIV, 200FG and 200HIV cohorts, respectively. Moreover, to avoid confounding effect of COVID-19 infection, COVID-19 positive individuals were removed from the 2000HIV. All 2000HIV individuals were COVID-19 unvaccinated, resulting in 588 individuals. (B). Protein measurements are presented as Normalized Protein expression (NPX) values, which is Olink’s relative protein quantification unit on log2 scale. In each of the four panels from the Olink® Explore 1536 platform, IL-6, TNF, CXCL8 were measured as technical duplicates for quality control purposes. Strong correlations (spearman correlation r > 0·9) were observed between the technical duplicates among panels, and therefore, we selected the measurements from the inflammatory panel. Next, we excluded proteins with LOD >= 25 of the samples, resulting in 1306 proteins (2000HIV and 200FG), 1293 (200HIV) for follow-up analysis.

**Supplementary Figure 2. Data distribution of cumulative antiretroviral drugs exposure**

Supplementary Figure 2: Data distribution of cumulative antiretroviral drugs exposure. (A) raw data. (B) Inverse transform data.

## **Supplementary Figure 3. Raw data distribution of cytokines secreted at 24** **hours**

Supplementary Figure 3: Raw data distribution of cytokines secreted at 24 hours: IL-1β, IL-6, IL-8, MCP-1, MIP-1a.

## **Supplementary Figure 4. Inverse-rank transformed data distribution of cytokines secreted by PBCMs at 24 hours**

Supplementary Figure 4: Inverse-rank transformed data distribution of cytokines secreted by PBCMs at 24 hours: IL-1β, IL-6, IL-8, MCP-1, MIP-1a

## **Supplementary Figure 5. Raw Data distribution of cytokines produced by PBMCs at 7 days**

Supplementary Figure 5: Raw Data distribution of cytokines produced by PBMCs at 7 days: IL-5, IL-10, IL-17, IL-22 and IFNγ.

## **Supplementary Figure 6. Inverse-rank transformed data of cytokines produced by PBMCs at 7 days**

Supplementary Figure 6: Inverse-rank transformed data of cytokines produced by PBMCs at 7 days: IL-5, IL-10, IL-17, IL-22 and IFNγ.

## **Supplementary Figure 7. Visualization of the importance score of each one of the 77 age-predictors**

Supplementary Figure 7: Visualization of the importance score of each one of the 77 age-predictors calculated by lasso regression model.

## **Supplementary Figure 8. Forest plot of associations between age advancement and demographics, HIV-specific factors and comorbidities in PLHIV**


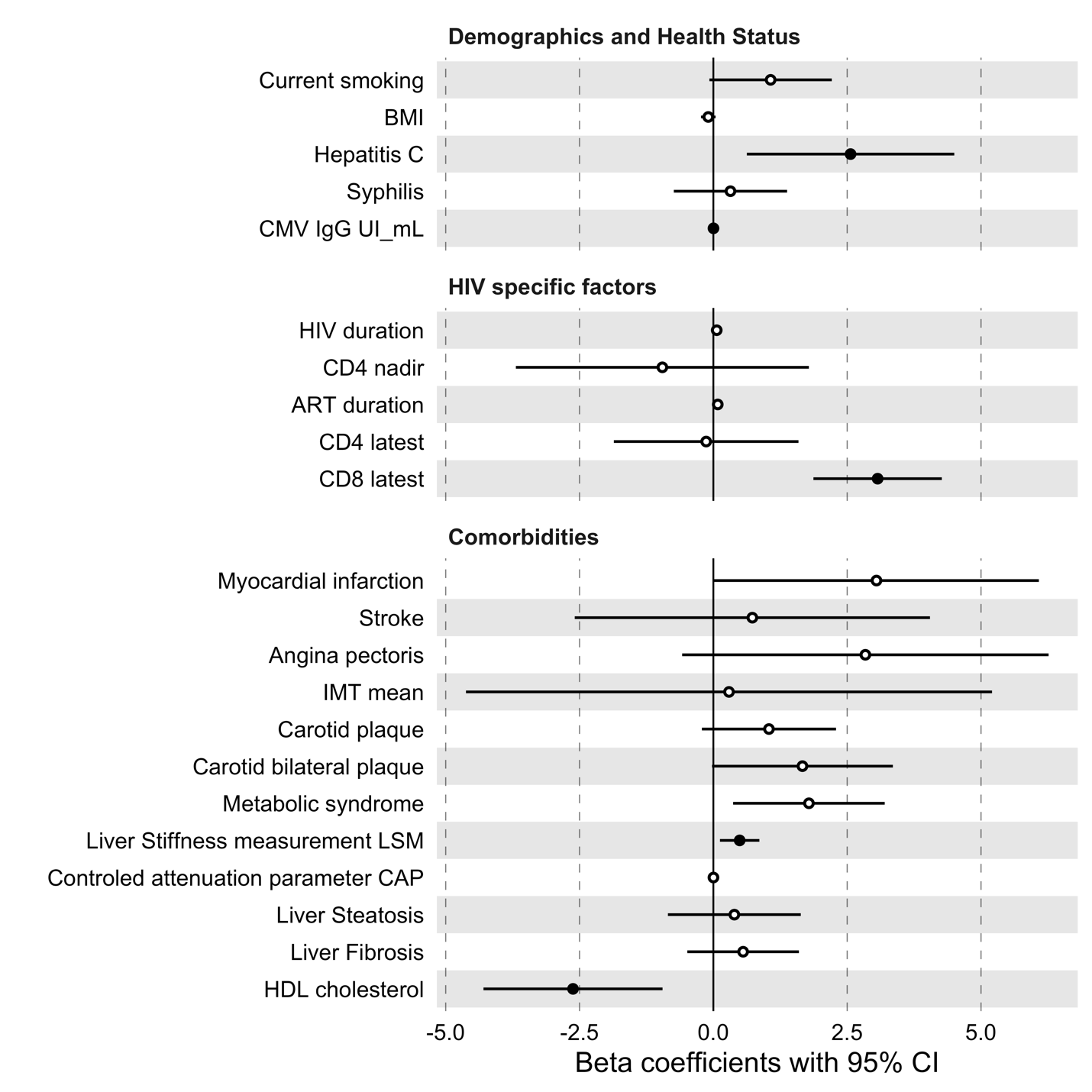


Supplementary Figure 8: Forest plot of associations between age advancement and demographics, HIV-specific factors and comorbidities in PLHIV. For each of the associations between age advancement and clinical parameters, the standardized beta and the confidence interval derived from linear models were plotted. The model was corrected by traditional cardiovascular risk factors such as: Type 2 diabetes, hypercholesterolemia, hypertriglyceridemia

## **Supplementary Figure 9. Correlation of predictors** **upregulated with chronological age (GFAP, WNT9A, SMOC1 and WISP2) and age advancement**

Supplementary Figure 9: (A) Correlation of predictors upregulated with chronological age (GFAP, WNT9A, SMOC1 and WISP2) and age advancement, P values are obtained from univariate linear regression model between age advancement and the protein of interest. (B) Comparison of NPX levels of GFAP, WNT9A, SMOC1 and WISP2 between participants with myocardial infarction and diabetes type 2. Participants were stratified according to faster (age advancement > 0) or lower age advancement (age advancement < 0). Statistical significance was estimated by Wilcoxon unpaired test.

## **Supplementary Figure 10. Correlation plots showing cytokines significant associated with age advancement (FDR<0·05) displayed in heat maps (from Figure 4)**

Supplementary Figure 10: Correlation plots showing cytokines significant associated with age advancement (FDR<0·05) displayed in heat maps (from Figure 3). (A) 24 hours (B) 7 days.

## **Supplementary Figure 11.**

## Supplementary Figure 11: Box plots comparing age advancement across different categories of HIV infection duration (in years). Statistical significance was assessed using the Wilcoxon test. None of the comparisons showed significant adjusted p-values, as follows: <5 years vs. 5–10 years: p.adj = 1; <5 years vs. 10–15 years: p.adj = 0.87; <5 years vs. >15 years: p.adj = 0.62; 5–10 years vs. 10–15 years: p.adj = 1; 5–10 years vs. >15 years: p.adj = 1; 10–15 years vs. >15 years: p.adj = 1.

# Supplementary Tables

## Supplementary Table 1. Stimulation scheme for evaluation of ex-vivo cytokine production

| PBMC 24-hour stimulation experiments | | | |
| --- | --- | --- | --- |
| Stimulus | Final Concentration | Manufacturer | Cat. no |
| 1. RPMI | NA | Life Technologies |  |
| 1. Poly I:C | 100 µg/mL | Invivogen | Tlrl-pic-5 |
| 1. LPS | 10 ng/mL | Sigma-Aldrich | L4005-100mg |
| 1. Imiquimod | 5 µg/mL | Invivogen | Tlr-imq |
| 1. Human recombinant IL-1α | 10 ng/mL | R&D Bio-techne | 200-LA-010 |
| 1. HIV-ENV (peptide pool) | 1 µg/mL | JPT Peptide Technologies | PM-HIV-ENV |
| 1. CMV (peptide pool) | 1 µg/mL | JPT Peptide Technologies | PM-PP65-2 |
| 1. S. pneumoniae | 5x10^6^/mL | In-house production | Strain: ATCC49619 |

| PBMC 7-day stimulation experiments | | | |
| --- | --- | --- | --- |
| Stimulus | Final Concentration | Manufacturer | Cat. no |
| 1. RPMI | NA | Gibco | 22409031 |
| 1. E. coli | 1x10^6^/mL | In-house production | Strain: ATCC35218 |
| 1. S· aureus | 1x10^6^/mL | In-house production | Strain: ATCC29213 |
| 1. S. Pneumoniae | 5x10^6^/mL | In-house production | Strain: ATCC49619 |
| 1. M. tuberculosis | 5 µg/mL | BEI Resources - H37Rv | NR-14822 |
| 1. C. albicans (conidia) | 1x10^6^/mL | In-house production | Strain: UC820 |
| 1. PHA | 10 µg/mL | Sigma-Aldrich | L9017-5MG |
| 1. C. albicans (hyphae) | 1x10^6^/mL | In-house production | Strain: UC820 |

## Supplementary Table 2. Variables tested for association with age advancement using a linear regression model

|  | Predictors |
| --- | --- |
| Demographics | Sex, smoking, hepatitis C (previous infection), syphilis (previous infection), cytomegalovirus (CMV) infection (IU/mL). |
| HIV-specific factors | HIV duration (years), ART duration (years), residual viremia (during the three years prior to baseline quantifiable viral load VL > 40 copies/ml), CD4 nadir (cells/mm3), CD4 and CD8 counts latest measurement (cells/mm3). |
| Comorbidities | At the moment of the inclusion, the presence of type 2 diabetes, hypercholesterolemia, hypertriglyceridemia, hypertension, myocardial infarction, stroke, angina pectoris, psychiatry disease, intima medium thickness measurement (IMT), carotid plaque, carotid bilateral plaque, metabolic syndrome, liver stiffness measurement (LSM) as proxy of liver steatosis, control attenuation parameter (CAP) as a proxy of liver fibrosis and plasma level of HDL cholesterol. |
| Exposure to antiretroviral medications | NNRTI: non-nucleoside reverse transcriptase inhibitor  NRTI: nucleoside reverse transcriptase inhibitor  INSTI: Integrase inhibitors  PI: Protease Inhibitors |

## Supplementary Table 3. Association results of 1306 plasma proteins with chronological age in healthy controls (200FG cohort)

| Protein | log FC | p-value | FDR | Beta |
| --- | --- | --- | --- | --- |
| NEFL | 0·02 | 1·5e-14 | 1·96e-11 | 22·08 |
| EDA2R | 0·02 | 4·83e-12 | 3·15e-09 | 16·34 |
| WNT9A | 0·01 | 4·21e-11 | 1·83e-08 | 14·19 |
| CXCL17 | 0·03 | 3·09e-10 | 1·01e-07 | 12·22 |
| COL9A1 | -0·03 | 6·8e-10 | 1·78e-07 | 11·44 |
| HAVCR1 | 0·03 | 1·16e-09 | 2·54e-07 | 10·90 |
| GFAP | 0·02 | 7·02e-09 | 1·31e-06 | 9·13 |
| FLT3 | -0·01 | 2·89e-08 | 4·71e-06 | 7·74 |
| ADGRG1 | 0·03 | 5·54e-08 | 8·03e-06 | 7·10 |
| LTBP2 | 0·01 | 6·85e-08 | 8·95e-06 | 6·89 |
| GDF15 | 0·02 | 1·84e-07 | 2·19e-05 | 5·92 |
| PSG1 | 0·04 | 2·04e-07 | 2·22e-05 | 5·83 |
| CXCL14 | 0·03 | 2·23e-07 | 2·24e-05 | 5·73 |
| IL6 | 0·04 | 2·77e-07 | 2·55e-05 | 5·52 |
| RET | -0·01 | 2·93e-07 | 2·55e-05 | 5·47 |
| KLK4 | 0·02 | 3·69e-07 | 3·01e-05 | 5·24 |
| MLN | 0·03 | 4·73e-07 | 3·64e-05 | 5·00 |
| CCL3 | 0·01 | 4·67e-06 | 0·00 | 2·77 |
| CDCP1 | 0·02 | 5·51e-06 | 0·00 | 2·61 |
| FGF5 | 0·01 | 5·65e-06 | 0·00 | 2·59 |
| CXCL10 | 0·02 | 6·11e-06 | 0·00 | 2·51 |
| VSIG4 | 0·01 | 6·82e-06 | 0·00 | 2·40 |
| CDON | -0·01 | 8·9e-06 | 0·00 | 2·15 |
| CTSV | -0·01 | 1·71e-05 | 0·00 | 1·51 |
| FABP9 | -0·02 | 2·18e-05 | 0·00 | 1·28 |
| CRH | -0·03 | 2·22e-05 | 0·00 | 1·26 |
| IGFBPL1 | 0·01 | 3·62e-05 | 0·00 | 0·79 |
| FLT3LG | 0·01 | 4·04e-05 | 0·00 | 0·69 |
| ADM | 0·01 | 4·06e-05 | 0·00 | 0·68 |
| TSPAN1 | 0·01 | 4·86e-05 | 0·00 | 0·51 |
| AMBP | 0·00 | 0·00 | 0·00 | -0·46 |
| DSG4 | -0·01 | 0·00 | 0·00 | -0·50 |
| OGN | 0·01 | 0·00 | 0·00 | -0·50 |
| SMOC1 | 0·01 | 0·00 | 0·00 | -0·61 |
| IGFBP4 | 0·01 | 0·00 | 0·00 | -0·65 |
| TREM2 | 0·02 | 0·00 | 0·00 | -0·68 |
| FAS | 0·00 | 0·00 | 0·00 | -0·82 |
| SCARF2 | 0·00 | 0·00 | 0·00 | -1·00 |
| WFDC2 | 0·01 | 0·00 | 0·00 | -1·06 |
| TNFRSF11B | 0·01 | 0·00 | 0·00 | -1·15 |
| TNXB | -0·00 | 0·00 | 0·01 | -1·35 |
| PODXL2 | -0·01 | 0·00 | 0·01 | -1·44 |
| DSG3 | -0·01 | 0·00 | 0·01 | -1·45 |
| ADAMTS16 | 0·00 | 0·00 | 0·01 | -1·46 |
| CCL13 | 0·02 | 0·00 | 0·01 | -1·61 |
| EFEMP1 | 0·01 | 0·00 | 0·01 | -1·64 |
| BCAN | -0·01 | 0·00 | 0·01 | -1·65 |
| WISP2 | 0·01 | 0·00 | 0·01 | -1·69 |
| TNFRSF11A | 0·01 | 0·00 | 0·01 | -1·73 |
| HSPB6 | 0·01 | 0·00 | 0·01 | -1·74 |
| SNCG | 0·02 | 0·00 | 0·01 | -1·78 |
| NFASC | 0·00 | 0·00 | 0·01 | -1·82 |
| TNFRSF1A | 0·00 | 0·00 | 0·01 | -1·86 |
| CD300E | 0·01 | 0·00 | 0·01 | -1·87 |
| COL1A1 | -0·00 | 0·00 | 0·01 | -1·87 |
| NCAM2 | -0·00 | 0·00 | 0·01 | -1·90 |
| MEPE | -0·01 | 0·00 | 0·01 | -1·91 |
| TGFB1 | 0·01 | 0·00 | 0·01 | -1·94 |
| LGALS9 | 0·01 | 0·00 | 0·01 | -1·98 |
| MSR1 | 0·01 | 0·00 | 0·01 | -2·07 |
| CGA | 0·02 | 0·00 | 0·01 | -2·09 |
| MOG | 0·01 | 0·00 | 0·01 | -2·09 |
| LAG3 | 0·01 | 0·00 | 0·01 | -2·12 |
| PROK1 | -0·01 | 0·00 | 0·01 | -2·13 |
| SCARB2 | 0·00 | 0·00 | 0·01 | -2·16 |
| MATN3 | -0·01 | 0·00 | 0·01 | -2·17 |
| LYPD3 | -0·01 | 0·00 | 0·01 | -2·26 |
| CCL2 | 0·01 | 0·00 | 0·01 | -2·35 |
| NTproBNP | 0·02 | 0·00 | 0·01 | -2·35 |
| CCL11 | 0·01 | 0·00 | 0·01 | -2·38 |
| MEGF10 | -0·01 | 0·00 | 0·01 | -2·43 |
| CXCL9 | 0·02 | 0·00 | 0·02 | -2·46 |
| TFF1 | 0·02 | 0·00 | 0·02 | -2·47 |
| PIK3IP1 | 0·00 | 0·00 | 0·02 | -2·48 |
| IL17D | 0·00 | 0·00 | 0·02 | -2·60 |
| FSTL3 | 0·01 | 0·00 | 0·02 | -2·67 |
| BOC | -0·00 | 0·00 | 0·02 | -2·67 |
| NCAN | -0·01 | 0·00 | 0·02 | -2·75 |
| TIMP4 | 0·00 | 0·00 | 0·02 | -2·88 |
| TNFSF11 | -0·01 | 0·00 | 0·02 | -2·88 |
| SPINK6 | 0·01 | 0·00 | 0·03 | -2·94 |
| LAIR1 | 0·01 | 0·00 | 0·03 | -2·96 |
| WIF1 | -0·01 | 0·00 | 0·03 | -2·99 |
| KLK13 | -0·01 | 0·00 | 0·03 | -3·01 |
| KRT5 | -0·01 | 0·00 | 0·03 | -3·03 |
| CRTAC1 | 0·00 | 0·00 | 0·03 | -3·08 |
| CD74 | 0·00 | 0·00 | 0·03 | -3·13 |
| LRRN1 | -0·01 | 0·00 | 0·03 | -3·13 |
| HGF | 0·00 | 0·00 | 0·03 | -3·14 |
| HAVCR2 | 0·00 | 0·00 | 0·03 | -3·21 |
| MSTN | -0·01 | 0·00 | 0·03 | -3·22 |
| EBI3_IL27 | 0·00 | 0·00 | 0·03 | -3·25 |
| EFNA1 | 0·00 | 0·00 | 0·03 | -3·25 |
| DCN | 0·00 | 0·00 | 0·03 | -3·26 |
| KIT | -0·00 | 0·00 | 0·03 | -3·26 |
| CXCL11 | 0·02 | 0·00 | 0·03 | -3·30 |
| PGF | 0·00 | 0·00 | 0·03 | -3·35 |
| RSPO3 | 0·00 | 0·00 | 0·04 | -3·42 |
| FUT3_FUT5 | 0·01 | 0·00 | 0·04 | -3·49 |
| IL5RA | 0·01 | 0·00 | 0·04 | -3·51 |
| DPP4 | -0·01 | 0·00 | 0·04 | -3·60 |
| REG4 | 0·01 | 0·00 | 0·05 | -3·64 |
| TNR | -0·01 | 0·00 | 0·05 | -3·73 |
| WISP1 | 0·00 | 0·00 | 0·05 | -3·77 |
| BTN3A2 | 0·01 | 0·00 | 0·05 | -3·82 |
| FABP4 | 0·01 | 0·00 | 0·05 | -3·82 |
| CCL27 | 0·01 | 0·00 | 0·05 | -3·83 |
| NDRG1 | -0·01 | 0·00 | 0·06 | -3·86 |
| NPPC | -0·01 | 0·00 | 0·06 | -3·90 |
| GALNT7 | -0·00 | 0·00 | 0·06 | -3·92 |
| LEFTY2 | 0·01 | 0·00 | 0·06 | -3·92 |
| TYRO3 | -0·00 | 0·00 | 0·06 | -3·97 |
| FURIN | 0·01 | 0·00 | 0·06 | -3·99 |
| SSC5D | 0·01 | 0·00 | 0·06 | -3·99 |
| KLRB1 | 0·00 | 0·00 | 0·06 | -4·02 |
| ENTPD5 | 0·00 | 0·00 | 0·07 | -4·07 |
| MMP12 | 0·01 | 0·00 | 0·07 | -4·11 |
| SOST | 0·01 | 0·00 | 0·07 | -4·12 |
| CCL8 | 0·01 | 0·00 | 0·07 | -4·13 |
| GFOD2 | -0·03 | 0·00 | 0·07 | -4·13 |
| IFNG | 0·02 | 0·00 | 0·07 | -4·15 |
| C4BPB | 0·01 | 0·00 | 0·08 | -4·25 |
| CDHR2 | 0·01 | 0·00 | 0·08 | -4·26 |
| PLXNB2 | 0·00 | 0·00 | 0·08 | -4·30 |
| MEP1B | 0·02 | 0·00 | 0·09 | -4·36 |
| CTSC | 0·01 | 0·00 | 0·09 | -4·36 |
| FCRLB | 0·01 | 0·00 | 0·09 | -4·36 |
| CNTN2 | 0·01 | 0·00 | 0·09 | -4·41 |
| CR2 | -0·01 | 0·00 | 0·09 | -4·41 |
| NELL2 | -0·00 | 0·00 | 0·09 | -4·44 |
| PILRA | 0·00 | 0·00 | 0·09 | -4·46 |
| CKAP4 | 0·00 | 0·00 | 0·09 | -4·46 |
| SLAMF7 | 0·01 | 0·00 | 0·09 | -4·47 |
| RNASET2 | 0·00 | 0·00 | 0·09 | -4·47 |
| LAMP3 | 0·01 | 0·00 | 0·09 | -4·48 |
| THOP1 | -0·00 | 0·01 | 0·09 | -4·49 |
| FASLG | -0·00 | 0·01 | 0·09 | -4·50 |
| ARSA | 0·01 | 0·01 | 0·09 | -4·53 |
| IL12RB1 | 0·00 | 0·01 | 0·09 | -4·54 |
| CHI3L1 | 0·01 | 0·01 | 0·10 | -4·57 |
| CHIT1 | 0·03 | 0·01 | 0·11 | -4·67 |
| PTPRS | -0·00 | 0·01 | 0·11 | -4·67 |
| SEZ6L2 | -0·00 | 0·01 | 0·11 | -4·68 |
| MUC13 | 0·01 | 0·01 | 0·11 | -4·71 |
| PLA2G2A | 0·01 | 0·01 | 0·11 | -4·72 |
| SERPINA11 | 0·00 | 0·01 | 0·11 | -4·72 |
| RBP2 | 0·01 | 0·01 | 0·11 | -4·75 |
| TNFRSF1B | 0·00 | 0·01 | 0·11 | -4·75 |
| CLMP | 0·00 | 0·01 | 0·12 | -4·77 |
| IL1RL2 | 0·00 | 0·01 | 0·12 | -4·78 |
| CCL7 | 0·01 | 0·01 | 0·12 | -4·78 |
| NPM1 | -0·01 | 0·01 | 0·12 | -4·81 |
| KLK8 | -0·00 | 0·01 | 0·12 | -4·84 |
| CD59 | 0·00 | 0·01 | 0·12 | -4·85 |
| MMP13 | -0·00 | 0·01 | 0·12 | -4·86 |
| ITGA5 | 0·00 | 0·01 | 0·12 | -4·87 |
| CCDC80 | 0·00 | 0·01 | 0·13 | -4·89 |
| MDGA1 | -0·01 | 0·01 | 0·13 | -4·93 |
| IL12B | 0·01 | 0·01 | 0·14 | -5·01 |
| CDHR1 | -0·00 | 0·01 | 0·14 | -5·02 |
| TNF | 0·00 | 0·01 | 0·14 | -5·03 |
| ANXA10 | 0·01 | 0·01 | 0·14 | -5·03 |
| EGFR | -0·00 | 0·01 | 0·15 | -5·05 |
| CSF1 | 0·00 | 0·01 | 0·15 | -5·06 |
| TGFBR2 | 0·00 | 0·01 | 0·15 | -5·07 |
| SPINK1 | 0·00 | 0·01 | 0·15 | -5·08 |
| MERTK | 0·00 | 0·01 | 0·15 | -5·08 |
| RARRES2 | 0·01 | 0·02 | 0·16 | -5·13 |
| TNFRSF13B | 0·01 | 0·02 | 0·16 | -5·14 |
| AHSP | -0·01 | 0·02 | 0·16 | -5·15 |
| SORCS2 | 0·00 | 0·02 | 0·16 | -5·16 |
| REG3A | 0·01 | 0·02 | 0·16 | -5·16 |
| ST6GAL1 | 0·00 | 0·02 | 0·16 | -5·17 |
| SPON2 | 0·00 | 0·02 | 0·16 | -5·18 |
| CA9 | 0·01 | 0·02 | 0·16 | -5·19 |
| IGF2R | 0·00 | 0·02 | 0·16 | -5·20 |
| OMD | -0·01 | 0·02 | 0·16 | -5·21 |
| CD1C | -0·00 | 0·02 | 0·16 | -5·21 |
| SPINK5 | -0·00 | 0·02 | 0·16 | -5·24 |
| DKK3 | 0·00 | 0·02 | 0·17 | -5·25 |
| KAZALD1 | -0·00 | 0·02 | 0·17 | -5·27 |
| PON3 | -0·00 | 0·02 | 0·17 | -5·29 |
| TNFRSF14 | 0·00 | 0·02 | 0·18 | -5·33 |
| CDH15 | 0·01 | 0·02 | 0·18 | -5·36 |
| GZMH | 0·01 | 0·02 | 0·18 | -5·36 |
| RTN4R | 0·00 | 0·02 | 0·18 | -5·37 |
| MFAP5 | 0·01 | 0·02 | 0·19 | -5·40 |
| LRIG1 | 0·00 | 0·02 | 0·19 | -5·41 |
| TNFSF13 | 0·00 | 0·02 | 0·20 | -5·44 |
| DSC2 | 0·00 | 0·03 | 0·20 | -5·47 |
| KLRD1 | 0·00 | 0·03 | 0·21 | -5·48 |
| DPEP1 | -0·00 | 0·03 | 0·21 | -5·49 |
| FST | 0·01 | 0·03 | 0·21 | -5·49 |
| SIGLEC1 | 0·01 | 0·03 | 0·21 | -5·49 |
| WFIKKN1 | -0·01 | 0·03 | 0·21 | -5·52 |
| C2 | 0·00 | 0·03 | 0·21 | -5·52 |
| SKAP1 | -0·00 | 0·03 | 0·21 | -5·52 |
| CST6 | -0·00 | 0·03 | 0·21 | -5·53 |
| CDH3 | -0·00 | 0·03 | 0·21 | -5·53 |
| CCL16 | 0·00 | 0·03 | 0·21 | -5·54 |
| COLEC12 | 0·00 | 0·03 | 0·21 | -5·55 |
| VWA1 | 0·00 | 0·03 | 0·21 | -5·55 |
| ISLR2 | -0·00 | 0·03 | 0·21 | -5·56 |
| CCL19 | 0·01 | 0·03 | 0·21 | -5·57 |
| FAM3C | 0·00 | 0·03 | 0·22 | -5·59 |
| XCL1 | 0·01 | 0·03 | 0·22 | -5·60 |
| NINJ1 | 0·00 | 0·03 | 0·23 | -5·63 |
| TXNDC5 | 0·01 | 0·03 | 0·23 | -5·64 |
| PRSS27 | -0·00 | 0·03 | 0·23 | -5·65 |
| SCGB1A1 | 0·00 | 0·03 | 0·23 | -5·65 |
| MNDA | -0·01 | 0·03 | 0·24 | -5·69 |
| CA6 | -0·01 | 0·04 | 0·25 | -5·73 |
| CD27 | 0·00 | 0·04 | 0·25 | -5·76 |
| IL4R | 0·00 | 0·04 | 0·26 | -5·76 |
| SPINT1 | -0·00 | 0·04 | 0·26 | -5·78 |
| ADGRG2 | -0·00 | 0·04 | 0·26 | -5·78 |
| TIMP1 | 0·00 | 0·04 | 0·26 | -5·78 |
| ARG1 | -0·01 | 0·04 | 0·26 | -5·78 |
| PLAT | 0·01 | 0·04 | 0·27 | -5·81 |
| SLAMF6 | 0·00 | 0·04 | 0·27 | -5·82 |
| MB | 0·00 | 0·04 | 0·27 | -5·82 |
| S100A16 | -0·01 | 0·04 | 0·27 | -5·85 |
| SCGB3A2 | -0·01 | 0·04 | 0·28 | -5·88 |
| GFRA1 | 0·00 | 0·04 | 0·28 | -5·89 |
| LAYN | 0·00 | 0·04 | 0·28 | -5·89 |
| PRCP | 0·00 | 0·05 | 0·29 | -5·90 |
| IL19 | -0·01 | 0·05 | 0·29 | -5·91 |
| COL6A3 | 0·00 | 0·05 | 0·29 | -5·92 |
| CST3 | 0·00 | 0·05 | 0·29 | -5·93 |
| NPDC1 | 0·00 | 0·05 | 0·29 | -5·93 |
| TNFRSF10B | 0·00 | 0·05 | 0·29 | -5·93 |
| PTK7 | -0·00 | 0·05 | 0·29 | -5·94 |
| CXCL8 | 0·01 | 0·05 | 0·29 | -5·95 |
| TNFRSF10A | 0·00 | 0·05 | 0·30 | -5·96 |
| ENG | -0·00 | 0·05 | 0·30 | -5·98 |
| IL17C | -0·01 | 0·05 | 0·30 | -5·98 |
| ADGRE2 | 0·00 | 0·05 | 0·30 | -5·99 |
| TNFSF14 | 0·00 | 0·05 | 0·30 | -5·99 |
| ANGPTL2 | 0·00 | 0·05 | 0·30 | -6·00 |
| TNFRSF4 | 0·00 | 0·05 | 0·30 | -6·01 |
| CARHSP1 | -0·01 | 0·05 | 0·30 | -6·01 |
| KLK10 | -0·00 | 0·05 | 0·30 | -6·02 |
| BLMH | -0·00 | 0·05 | 0·30 | -6·02 |
| IGFBP7 | 0·00 | 0·05 | 0·30 | -6·02 |
| DAG1 | 0·00 | 0·05 | 0·30 | -6·02 |
| SMAD5 | 0·00 | 0·05 | 0·31 | -6·05 |
| CCN1 | 0·01 | 0·06 | 0·31 | -6·05 |
| AXL | -0·00 | 0·06 | 0·31 | -6·05 |
| IL2RA | 0·00 | 0·06 | 0·31 | -6·06 |
| CTSH | 0·01 | 0·06 | 0·31 | -6·06 |
| GALNT3 | -0·00 | 0·06 | 0·31 | -6·06 |
| IL18R1 | 0·00 | 0·06 | 0·31 | -6·07 |
| MZB1 | 0·00 | 0·06 | 0·31 | -6·08 |
| ITIH3 | 0·00 | 0·06 | 0·31 | -6·08 |
| GPA33 | -0·02 | 0·06 | 0·31 | -6·08 |
| SEMA7A | 0·00 | 0·06 | 0·31 | -6·09 |
| IL18BP | 0·00 | 0·06 | 0·31 | -6·09 |
| TRIAP1 | 0·00 | 0·06 | 0·31 | -6·09 |
| TNC | -0·00 | 0·06 | 0·31 | -6·10 |
| CHRDL1 | 0·00 | 0·06 | 0·32 | -6·11 |
| CRNN | -0·01 | 0·06 | 0·32 | -6·11 |
| BPIFB1 | 0·01 | 0·06 | 0·32 | -6·11 |
| PPY | 0·01 | 0·06 | 0·32 | -6·13 |
| IL13RA1 | 0·00 | 0·06 | 0·32 | -6·13 |
| CD70 | 0·00 | 0·06 | 0·32 | -6·14 |
| ENPP7 | 0·01 | 0·06 | 0·32 | -6·14 |
| CD97 | 0·00 | 0·06 | 0·33 | -6·15 |
| WFIKKN2 | -0·00 | 0·06 | 0·33 | -6·16 |
| GALNT2 | -0·00 | 0·06 | 0·33 | -6·17 |
| GCNT1 | 0·00 | 0·06 | 0·33 | -6·17 |
| ABHD14B | 0·01 | 0·06 | 0·33 | -6·18 |
| RSPO1 | 0·00 | 0·07 | 0·33 | -6·18 |
| GPR37 | 0·01 | 0·07 | 0·33 | -6·19 |
| PRDX3 | 0·01 | 0·07 | 0·33 | -6·19 |
| CDH2 | 0·00 | 0·07 | 0·33 | -6·19 |
| ICOSLG | -0·00 | 0·07 | 0·33 | -6·19 |
| BTC | 0·01 | 0·07 | 0·33 | -6·20 |
| CES1 | 0·01 | 0·07 | 0·33 | -6·20 |
| SLAMF8 | 0·00 | 0·07 | 0·33 | -6·21 |
| CBLIF | 0·01 | 0·07 | 0·33 | -6·21 |
| CCL18 | 0·01 | 0·07 | 0·34 | -6·22 |
| SFRP1 | 0·00 | 0·07 | 0·34 | -6·23 |
| IL15 | 0·00 | 0·07 | 0·34 | -6·24 |
| ACP5 | 0·00 | 0·07 | 0·34 | -6·25 |
| PIGR | 0·00 | 0·07 | 0·35 | -6·26 |
| MAPK9 | 0·00 | 0·07 | 0·35 | -6·26 |
| FOLR2 | 0·00 | 0·07 | 0·35 | -6·26 |
| ADA | -0·00 | 0·07 | 0·35 | -6·27 |
| LY96 | 0·00 | 0·07 | 0·35 | -6·29 |
| TPP1 | 0·00 | 0·07 | 0·35 | -6·29 |
| SCARA5 | 0·00 | 0·08 | 0·35 | -6·29 |
| IL12A_IL12B | 0·00 | 0·08 | 0·36 | -6·30 |
| CCN3 | 0·00 | 0·08 | 0·36 | -6·30 |
| CD34 | -0·00 | 0·08 | 0·36 | -6·31 |
| ITGB5 | 0·00 | 0·08 | 0·36 | -6·31 |
| ALDH3A1 | -0·00 | 0·08 | 0·36 | -6·32 |
| EPCAM | -0·01 | 0·08 | 0·36 | -6·32 |
| COL18A1 | 0·00 | 0·08 | 0·36 | -6·32 |
| FCGR2B | -0·01 | 0·08 | 0·36 | -6·32 |
| REG1B | 0·00 | 0·08 | 0·36 | -6·32 |
| CNDP1 | -0·00 | 0·08 | 0·36 | -6·33 |
| AGRN | 0·00 | 0·08 | 0·36 | -6·33 |
| LEP | 0·01 | 0·08 | 0·36 | -6·33 |
| LRP1 | 0·00 | 0·08 | 0·36 | -6·34 |
| SCARB1 | -0·00 | 0·08 | 0·36 | -6·34 |
| GRN | 0·00 | 0·08 | 0·36 | -6·35 |
| HBQ1 | -0·00 | 0·08 | 0·36 | -6·35 |
| LILRB2 | 0·00 | 0·08 | 0·36 | -6·35 |
| TMPRSS15 | 0·01 | 0·08 | 0·36 | -6·35 |
| CCL24 | 0·01 | 0·08 | 0·36 | -6·35 |
| COL4A1 | -0·00 | 0·08 | 0·36 | -6·35 |
| KIRREL2 | -0·00 | 0·08 | 0·36 | -6·35 |
| CCL25 | 0·00 | 0·08 | 0·36 | -6·37 |
| PTPRF | 0·00 | 0·08 | 0·36 | -6·37 |
| TNFRSF19 | 0·00 | 0·08 | 0·37 | -6·39 |
| TGM2 | -0·01 | 0·09 | 0·37 | -6·39 |
| NOS1 | 0·00 | 0·09 | 0·37 | -6·39 |
| CSF3 | 0·00 | 0·09 | 0·37 | -6·40 |
| PRL | -0·00 | 0·09 | 0·37 | -6·41 |
| CHGB | 0·00 | 0·09 | 0·37 | -6·41 |
| MPI | 0·01 | 0·09 | 0·37 | -6·41 |
| ADAMTS15 | -0·01 | 0·09 | 0·37 | -6·42 |
| VNN2 | -0·00 | 0·09 | 0·37 | -6·42 |
| IQGAP2 | 0·00 | 0·09 | 0·38 | -6·43 |
| AGRP | -0·00 | 0·09 | 0·38 | -6·45 |
| FLT4 | 0·00 | 0·09 | 0·38 | -6·45 |
| HNMT | 0·00 | 0·09 | 0·38 | -6·46 |
| F2R | 0·00 | 0·09 | 0·38 | -6·46 |
| TNFRSF6B | 0·00 | 0·09 | 0·38 | -6·46 |
| DPY30 | 0·00 | 0·10 | 0·39 | -6·48 |
| PTH1R | -0·00 | 0·10 | 0·39 | -6·48 |
| PRELP | 0·00 | 0·10 | 0·39 | -6·48 |
| PCDH1 | 0·00 | 0·10 | 0·39 | -6·48 |
| CRTAM | 0·00 | 0·10 | 0·39 | -6·49 |
| PILRB | 0·00 | 0·10 | 0·39 | -6·49 |
| LTBR | 0·00 | 0·10 | 0·39 | -6·49 |
| C1QA | 0·00 | 0·10 | 0·39 | -6·50 |
| RGMA | -0·00 | 0·10 | 0·39 | -6·50 |
| AMIGO2 | -0·00 | 0·10 | 0·39 | -6·50 |
| CD79B | 0·00 | 0·10 | 0·39 | -6·50 |
| TCL1A | -0·01 | 0·10 | 0·40 | -6·51 |
| GAL | -0·00 | 0·10 | 0·40 | -6·51 |
| CD200 | 0·00 | 0·10 | 0·40 | -6·52 |
| CCL15 | 0·00 | 0·10 | 0·40 | -6·52 |
| NPTN | -0·01 | 0·10 | 0·40 | -6·52 |
| PAMR1 | 0·00 | 0·10 | 0·40 | -6·53 |
| CD302 | 0·00 | 0·10 | 0·40 | -6·54 |
| OMG | 0·01 | 0·10 | 0·40 | -6·55 |
| DKKL1 | 0·01 | 0·10 | 0·40 | -6·55 |
| CASC4 | 0·00 | 0·10 | 0·40 | -6·55 |
| FXYD5 | 0·00 | 0·11 | 0·41 | -6·56 |
| ITGB2 | 0·00 | 0·11 | 0·41 | -6·56 |
| LBP | 0·00 | 0·11 | 0·41 | -6·56 |
| SELP | 0·00 | 0·11 | 0·41 | -6·56 |
| CD274 | 0·00 | 0·11 | 0·41 | -6·56 |
| VEGFA | 0·00 | 0·11 | 0·41 | -6·57 |
| GAS6 | -0·00 | 0·11 | 0·41 | -6·57 |
| LGALS1 | 0·00 | 0·11 | 0·41 | -6·57 |
| FGF21 | 0·01 | 0·11 | 0·41 | -6·57 |
| LDLR | 0·00 | 0·11 | 0·41 | -6·57 |
| FCRL2 | 0·00 | 0·11 | 0·41 | -6·58 |
| SIAE | 0·00 | 0·11 | 0·41 | -6·58 |
| AFP | 0·00 | 0·11 | 0·41 | -6·58 |
| NUDC | 0·00 | 0·11 | 0·41 | -6·58 |
| ANGPTL3 | 0·00 | 0·11 | 0·41 | -6·59 |
| NTF3 | -0·00 | 0·11 | 0·41 | -6·59 |
| GZMA | 0·00 | 0·11 | 0·41 | -6·59 |
| TNFRSF10C | 0·00 | 0·11 | 0·41 | -6·60 |
| TDGF1 | 0·02 | 0·11 | 0·41 | -6·60 |
| SPON1 | 0·00 | 0·11 | 0·41 | -6·60 |
| LILRB4 | 0·00 | 0·11 | 0·41 | -6·61 |
| CD4 | -0·00 | 0·11 | 0·41 | -6·62 |
| GSAP | 0·00 | 0·12 | 0·42 | -6·63 |
| ESAM | 0·00 | 0·12 | 0·42 | -6·63 |
| ICAM3 | 0·00 | 0·12 | 0·42 | -6·64 |
| CLEC6A | 0·00 | 0·12 | 0·42 | -6·64 |
| IL1R2 | -0·00 | 0·12 | 0·42 | -6·64 |
| STC1 | 0·00 | 0·12 | 0·42 | -6·64 |
| GPC1 | -0·00 | 0·12 | 0·42 | -6·65 |
| LTA | -0·00 | 0·12 | 0·42 | -6·65 |
| DDR1 | -0·00 | 0·12 | 0·42 | -6·65 |
| GP6 | 0·01 | 0·12 | 0·42 | -6·65 |
| CSTB | 0·00 | 0·12 | 0·42 | -6·66 |
| IL15RA | 0·00 | 0·12 | 0·43 | -6·66 |
| PKLR | -0·00 | 0·12 | 0·43 | -6·67 |
| S100A4 | -0·00 | 0·12 | 0·43 | -6·67 |
| CLEC1A | 0·00 | 0·12 | 0·43 | -6·67 |
| PPME1 | -0·01 | 0·12 | 0·43 | -6·68 |
| DCTPP1 | 0·00 | 0·13 | 0·44 | -6·69 |
| ICAM2 | 0·00 | 0·13 | 0·44 | -6·69 |
| MMP7 | 0·01 | 0·13 | 0·44 | -6·70 |
| PSMG3 | -0·00 | 0·13 | 0·44 | -6·71 |
| EFNA4 | 0·00 | 0·13 | 0·44 | -6·71 |
| DLK1 | 0·00 | 0·13 | 0·44 | -6·72 |
| L1CAM | 0·00 | 0·13 | 0·44 | -6·72 |
| TNFRSF8 | 0·00 | 0·13 | 0·44 | -6·72 |
| GP2 | 0·00 | 0·13 | 0·44 | -6·72 |
| TMSB10 | 0·01 | 0·13 | 0·45 | -6·73 |
| ROBO1 | -0·00 | 0·13 | 0·45 | -6·74 |
| P4HB | 0·00 | 0·14 | 0·45 | -6·74 |
| AGER | -0·00 | 0·14 | 0·45 | -6·74 |
| KIR2DL3 | 0·00 | 0·14 | 0·45 | -6·74 |
| CCL23 | 0·00 | 0·14 | 0·45 | -6·75 |
| CNTN5 | 0·00 | 0·14 | 0·45 | -6·75 |
| FLI1 | -0·01 | 0·14 | 0·45 | -6·76 |
| SPINK4 | 0·00 | 0·14 | 0·45 | -6·76 |
| FUS | -0·00 | 0·14 | 0·45 | -6·76 |
| ICAM5 | -0·00 | 0·14 | 0·45 | -6·76 |
| MEGF9 | -0·00 | 0·14 | 0·46 | -6·76 |
| EPHB6 | -0·00 | 0·14 | 0·46 | -6·77 |
| ANPEP | -0·00 | 0·14 | 0·46 | -6·77 |
| CXCL5 | 0·01 | 0·14 | 0·46 | -6·78 |
| CTSS | 0·00 | 0·14 | 0·46 | -6·78 |
| IL1R1 | 0·00 | 0·14 | 0·46 | -6·79 |
| NBL1 | 0·00 | 0·14 | 0·46 | -6·79 |
| DAB2 | 0·01 | 0·14 | 0·46 | -6·79 |
| GDNF | 0·00 | 0·14 | 0·46 | -6·79 |
| HDGF | 0·00 | 0·15 | 0·46 | -6·80 |
| IFNGR2 | -0·00 | 0·15 | 0·46 | -6·80 |
| NCF2 | -0·02 | 0·15 | 0·46 | -6·80 |
| TNFRSF12A | 0·00 | 0·15 | 0·46 | -6·80 |
| DTX3 | 0·00 | 0·15 | 0·46 | -6·80 |
| F9 | 0·00 | 0·15 | 0·47 | -6·81 |
| PSMD9 | -0·00 | 0·15 | 0·47 | -6·81 |
| TFPI | 0·00 | 0·15 | 0·47 | -6·81 |
| IGFBP3 | -0·00 | 0·15 | 0·47 | -6·81 |
| NDUFS6 | 0·00 | 0·15 | 0·47 | -6·82 |
| ODAM | -0·00 | 0·15 | 0·47 | -6·82 |
| CES2 | 0·00 | 0·15 | 0·47 | -6·82 |
| LAP3 | 0·00 | 0·15 | 0·47 | -6·82 |
| OPTC | 0·00 | 0·15 | 0·47 | -6·83 |
| FABP1 | 0·01 | 0·15 | 0·47 | -6·83 |
| VWC2 | 0·00 | 0·15 | 0·47 | -6·83 |
| TPSAB1 | 0·00 | 0·15 | 0·47 | -6·84 |
| XG | 0·00 | 0·16 | 0·48 | -6·86 |
| SIGLEC6 | 0·00 | 0·16 | 0·48 | -6·86 |
| CFC1 | 0·00 | 0·16 | 0·48 | -6·86 |
| PRDX5 | 0·01 | 0·16 | 0·49 | -6·87 |
| KIFBP | 0·01 | 0·16 | 0·49 | -6·87 |
| MDK | 0·00 | 0·16 | 0·49 | -6·87 |
| SULT2A1 | 0·00 | 0·16 | 0·49 | -6·87 |
| F3 | 0·00 | 0·16 | 0·49 | -6·87 |
| PSIP1 | -0·00 | 0·16 | 0·49 | -6·88 |
| CRELD2 | 0·00 | 0·16 | 0·49 | -6·88 |
| IL1RAP | -0·00 | 0·16 | 0·49 | -6·88 |
| ENPP5 | -0·00 | 0·16 | 0·49 | -6·88 |
| EPHA2 | 0·00 | 0·16 | 0·49 | -6·88 |
| NME3 | -0·00 | 0·16 | 0·49 | -6·89 |
| EPHA1 | 0·00 | 0·16 | 0·49 | -6·89 |
| LRPAP1 | 0·00 | 0·16 | 0·49 | -6·89 |
| LILRA5 | 0·00 | 0·16 | 0·49 | -6·89 |
| MCAM | 0·00 | 0·17 | 0·49 | -6·89 |
| PLIN1 | 0·00 | 0·17 | 0·49 | -6·90 |
| NCR1 | 0·00 | 0·17 | 0·49 | -6·90 |
| STC2 | -0·00 | 0·17 | 0·49 | -6·90 |
| BMP4 | 0·00 | 0·17 | 0·49 | -6·90 |
| LHB | -0·01 | 0·17 | 0·49 | -6·90 |
| ADAM8 | -0·00 | 0·17 | 0·49 | -6·91 |
| IGSF8 | 0·00 | 0·17 | 0·49 | -6·91 |
| PQBP1 | -0·00 | 0·17 | 0·49 | -6·91 |
| PTGDS | 0·00 | 0·17 | 0·49 | -6·91 |
| CLEC4A | -0·00 | 0·17 | 0·49 | -6·91 |
| PRKAR1A | 0·01 | 0·17 | 0·49 | -6·92 |
| SERPINA9 | 0·00 | 0·17 | 0·49 | -6·92 |
| MMP3 | 0·00 | 0·17 | 0·49 | -6·93 |
| TFF2 | 0·00 | 0·17 | 0·49 | -6·93 |
| CTRC | -0·00 | 0·18 | 0·50 | -6·94 |
| DNAJA2 | 0·01 | 0·18 | 0·50 | -6·94 |
| JAM2 | 0·00 | 0·18 | 0·50 | -6·94 |
| TFPI2 | 0·00 | 0·18 | 0·50 | -6·95 |
| AHCY | 0·00 | 0·18 | 0·50 | -6·95 |
| S100A12 | -0·01 | 0·18 | 0·51 | -6·96 |
| NOTCH3 | 0·00 | 0·18 | 0·51 | -6·96 |
| CXCL16 | 0·00 | 0·18 | 0·51 | -6·96 |
| SOD2 | -0·00 | 0·18 | 0·51 | -6·96 |
| SEZ6L | -0·00 | 0·18 | 0·51 | -6·96 |
| CXADR | -0·00 | 0·18 | 0·51 | -6·97 |
| SORT1 | 0·00 | 0·18 | 0·51 | -6·97 |
| IGFBP2 | 0·00 | 0·18 | 0·51 | -6·97 |
| TYMP | 0·00 | 0·19 | 0·51 | -6·98 |
| GFRA2 | -0·00 | 0·19 | 0·51 | -6·98 |
| VSTM1 | -0·00 | 0·19 | 0·51 | -6·98 |
| FCRL6 | 0·00 | 0·19 | 0·51 | -6·98 |
| ST3GAL1 | 0·00 | 0·19 | 0·51 | -6·98 |
| ITGB6 | -0·00 | 0·19 | 0·51 | -6·98 |
| TACC3 | 0·01 | 0·19 | 0·51 | -6·99 |
| MGMT | 0·01 | 0·19 | 0·51 | -6·99 |
| DAPP1 | 0·01 | 0·19 | 0·51 | -6·99 |
| CLEC14A | 0·00 | 0·19 | 0·51 | -6·99 |
| CCL4 | 0·00 | 0·19 | 0·52 | -7·00 |
| GLO1 | -0·00 | 0·19 | 0·52 | -7·01 |
| CRHBP | 0·00 | 0·20 | 0·53 | -7·01 |
| MET | -0·00 | 0·20 | 0·53 | -7·02 |
| APOH | 0·00 | 0·20 | 0·54 | -7·03 |
| SMPD1 | 0·00 | 0·20 | 0·54 | -7·04 |
| CALB1 | 0·00 | 0·20 | 0·54 | -7·05 |
| CPM | 0·00 | 0·20 | 0·54 | -7·05 |
| PLXDC1 | -0·00 | 0·21 | 0·54 | -7·05 |
| CLIP2 | 0·01 | 0·21 | 0·54 | -7·05 |
| DCBLD2 | 0·00 | 0·21 | 0·54 | -7·05 |
| CA1 | -0·00 | 0·21 | 0·54 | -7·05 |
| MESD | 0·01 | 0·21 | 0·55 | -7·06 |
| CD84 | -0·00 | 0·21 | 0·56 | -7·07 |
| EIF4G1 | 0·01 | 0·21 | 0·56 | -7·07 |
| CTRB1 | 0·00 | 0·21 | 0·56 | -7·08 |
| SIGLEC7 | 0·00 | 0·22 | 0·56 | -7·08 |
| C2CD2L | 0·00 | 0·22 | 0·56 | -7·08 |
| APLP1 | -0·00 | 0·22 | 0·56 | -7·09 |
| CDC27 | -0·00 | 0·22 | 0·57 | -7·09 |
| PDCD1 | 0·00 | 0·22 | 0·57 | -7·09 |
| RELT | 0·00 | 0·22 | 0·57 | -7·10 |
| TMPRSS5 | -0·00 | 0·22 | 0·57 | -7·10 |
| BCAM | 0·00 | 0·22 | 0·57 | -7·10 |
| S100A11 | -0·01 | 0·22 | 0·57 | -7·10 |
| CES3 | 0·00 | 0·22 | 0·57 | -7·10 |
| CPVL | 0·00 | 0·22 | 0·57 | -7·11 |
| ANGPTL4 | 0·00 | 0·22 | 0·57 | -7·11 |
| CD58 | -0·00 | 0·22 | 0·57 | -7·11 |
| GLRX | -0·00 | 0·22 | 0·57 | -7·11 |
| IL17RA | -0·00 | 0·23 | 0·57 | -7·12 |
| ADAMTS8 | -0·00 | 0·23 | 0·58 | -7·12 |
| ADAM15 | -0·00 | 0·23 | 0·58 | -7·12 |
| CASP1 | -0·01 | 0·23 | 0·58 | -7·12 |
| CD48 | 0·00 | 0·23 | 0·58 | -7·12 |
| LRP11 | 0·00 | 0·23 | 0·58 | -7·13 |
| FXN | 0·00 | 0·23 | 0·59 | -7·14 |
| TFRC | -0·00 | 0·23 | 0·59 | -7·14 |
| CCL17 | 0·00 | 0·23 | 0·59 | -7·14 |
| LILRB5 | -0·00 | 0·23 | 0·59 | -7·14 |
| ENTPD2 | -0·00 | 0·24 | 0·59 | -7·15 |
| MME | -0·00 | 0·24 | 0·59 | -7·15 |
| RRM2B | 0·00 | 0·24 | 0·59 | -7·15 |
| SHMT1 | 0·00 | 0·24 | 0·59 | -7·16 |
| LXN | -0·00 | 0·24 | 0·59 | -7·16 |
| SCG2 | 0·00 | 0·24 | 0·59 | -7·16 |
| DSG2 | -0·00 | 0·24 | 0·59 | -7·16 |
| EPO | 0·00 | 0·24 | 0·60 | -7·17 |
| OBP2B | -0·00 | 0·24 | 0·60 | -7·17 |
| RBP5 | 0·00 | 0·24 | 0·60 | -7·17 |
| IL10RB | 0·00 | 0·24 | 0·60 | -7·17 |
| SH2B3 | 0·01 | 0·24 | 0·60 | -7·17 |
| CPE | 0·00 | 0·24 | 0·60 | -7·17 |
| AGXT | -0·00 | 0·25 | 0·60 | -7·18 |
| DCTN6 | 0·00 | 0·25 | 0·60 | -7·18 |
| PLA2G15 | 0·00 | 0·25 | 0·60 | -7·18 |
| CBLN4 | -0·00 | 0·25 | 0·60 | -7·18 |
| NOMO1 | 0·00 | 0·25 | 0·60 | -7·18 |
| LGALS4 | 0·00 | 0·25 | 0·61 | -7·20 |
| EZR | 0·00 | 0·25 | 0·61 | -7·20 |
| RASSF2 | -0·01 | 0·25 | 0·61 | -7·20 |
| COMP | 0·00 | 0·26 | 0·61 | -7·20 |
| DBI | 0·00 | 0·26 | 0·61 | -7·21 |
| TIE1 | 0·00 | 0·26 | 0·61 | -7·21 |
| CD40 | 0·00 | 0·26 | 0·61 | -7·21 |
| GPKOW | -0·00 | 0·26 | 0·61 | -7·21 |
| ASGR1 | 0·00 | 0·26 | 0·62 | -7·22 |
| IMPA1 | -0·00 | 0·27 | 0·63 | -7·23 |
| LTBP3 | 0·00 | 0·27 | 0·63 | -7·23 |
| NCS1 | 0·00 | 0·27 | 0·63 | -7·23 |
| SIRPB1 | 0·00 | 0·27 | 0·63 | -7·23 |
| CD163 | 0·00 | 0·27 | 0·63 | -7·23 |
| PRSS8 | 0·00 | 0·27 | 0·63 | -7·24 |
| THBS4 | 0·00 | 0·27 | 0·63 | -7·24 |
| MICB_MICA | 0·01 | 0·27 | 0·63 | -7·24 |
| HSD11B1 | -0·00 | 0·27 | 0·63 | -7·24 |
| ACP6 | -0·00 | 0·27 | 0·63 | -7·24 |
| PRTFDC1 | 0·01 | 0·27 | 0·63 | -7·25 |
| ANXA5 | 0·00 | 0·27 | 0·63 | -7·25 |
| SFTPD | 0·00 | 0·27 | 0·63 | -7·25 |
| PODXL | -0·00 | 0·27 | 0·64 | -7·25 |
| PDGFRB | -0·00 | 0·28 | 0·64 | -7·26 |
| MAD1L1 | 0·00 | 0·28 | 0·64 | -7·26 |
| AKR1B1 | -0·00 | 0·28 | 0·64 | -7·26 |
| CASP2 | 0·00 | 0·28 | 0·64 | -7·26 |
| ITGAV | 0·00 | 0·28 | 0·65 | -7·27 |
| MYO9B | 0·00 | 0·28 | 0·65 | -7·27 |
| CRLF1 | -0·00 | 0·28 | 0·65 | -7·27 |
| CGREF1 | 0·00 | 0·28 | 0·65 | -7·28 |
| NAAA | 0·00 | 0·29 | 0·65 | -7·28 |
| ING1 | -0·00 | 0·29 | 0·65 | -7·28 |
| CPB1 | 0·00 | 0·29 | 0·65 | -7·28 |
| ITGA6 | 0·00 | 0·29 | 0·65 | -7·28 |
| TACSTD2 | -0·00 | 0·29 | 0·65 | -7·28 |
| EIF4EBP1 | -0·00 | 0·29 | 0·65 | -7·28 |
| BST1 | -0·00 | 0·29 | 0·65 | -7·29 |
| BTN2A1 | 0·00 | 0·29 | 0·65 | -7·29 |
| FABP6 | 0·00 | 0·29 | 0·65 | -7·29 |
| GSTA3 | 0·00 | 0·29 | 0·65 | -7·29 |
| THY1 | 0·00 | 0·29 | 0·65 | -7·29 |
| F7 | 0·00 | 0·29 | 0·65 | -7·29 |
| GDF2 | 0·00 | 0·29 | 0·65 | -7·30 |
| CASP3 | 0·00 | 0·29 | 0·66 | -7·30 |
| OGFR | -0·00 | 0·30 | 0·66 | -7·30 |
| LAIR2 | 0·00 | 0·30 | 0·66 | -7·30 |
| BID | 0·01 | 0·30 | 0·66 | -7·30 |
| LY9 | 0·00 | 0·30 | 0·66 | -7·31 |
| FCRL1 | -0·00 | 0·30 | 0·66 | -7·31 |
| EGLN1 | -0·01 | 0·30 | 0·66 | -7·31 |
| KDR | -0·00 | 0·30 | 0·66 | -7·31 |
| APEX1 | -0·00 | 0·30 | 0·66 | -7·31 |
| OSMR | 0·00 | 0·30 | 0·66 | -7·32 |
| IL17A | 0·00 | 0·30 | 0·66 | -7·32 |
| SIGLEC10 | 0·00 | 0·30 | 0·66 | -7·32 |
| IGF1R | 0·00 | 0·30 | 0·66 | -7·32 |
| PEAR1 | -0·00 | 0·31 | 0·67 | -7·32 |
| OXT | 0·01 | 0·31 | 0·67 | -7·33 |
| TEK | -0·00 | 0·31 | 0·67 | -7·33 |
| SERPINE1 | 0·00 | 0·31 | 0·67 | -7·33 |
| ANG | -0·00 | 0·31 | 0·67 | -7·33 |
| CLSTN1 | -0·00 | 0·31 | 0·67 | -7·33 |
| DDC | -0·00 | 0·31 | 0·67 | -7·33 |
| CLSTN2 | 0·00 | 0·31 | 0·67 | -7·34 |
| TCN2 | 0·00 | 0·31 | 0·67 | -7·34 |
| MFGE8 | 0·00 | 0·31 | 0·67 | -7·34 |
| CNTN3 | 0·00 | 0·32 | 0·67 | -7·34 |
| CD207 | -0·00 | 0·32 | 0·67 | -7·34 |
| SPINT2 | 0·00 | 0·32 | 0·67 | -7·35 |
| IGFBP6 | 0·00 | 0·32 | 0·67 | -7·35 |
| SRP14 | -0·00 | 0·32 | 0·67 | -7·35 |
| ART3 | -0·00 | 0·32 | 0·67 | -7·35 |
| CEACAM21 | 0·00 | 0·32 | 0·67 | -7·35 |
| PPP1R9B | 0·00 | 0·32 | 0·67 | -7·35 |
| FEN1 | -0·00 | 0·32 | 0·68 | -7·35 |
| DPP10 | -0·00 | 0·32 | 0·68 | -7·36 |
| COX5B | 0·00 | 0·32 | 0·68 | -7·36 |
| CD300C | 0·00 | 0·32 | 0·68 | -7·36 |
| CNTN4 | -0·00 | 0·33 | 0·68 | -7·36 |
| CLPS | 0·00 | 0·33 | 0·68 | -7·36 |
| PMVK | 0·00 | 0·33 | 0·69 | -7·37 |
| ULBP2 | 0·00 | 0·33 | 0·69 | -7·37 |
| FOPNL | 0·00 | 0·33 | 0·69 | -7·37 |
| BAG3 | -0·00 | 0·33 | 0·69 | -7·38 |
| TRIM21 | 0·00 | 0·33 | 0·69 | -7·38 |
| PLTP | -0·00 | 0·33 | 0·69 | -7·38 |
| DDX58 | 0·00 | 0·33 | 0·69 | -7·38 |
| CTSF | 0·00 | 0·33 | 0·69 | -7·38 |
| KLB | -0·00 | 0·33 | 0·69 | -7·38 |
| NCAM1 | -0·00 | 0·33 | 0·69 | -7·38 |
| FABP2 | 0·00 | 0·34 | 0·69 | -7·38 |
| PVALB | 0·00 | 0·34 | 0·69 | -7·38 |
| CCS | -0·00 | 0·34 | 0·69 | -7·38 |
| HYOU1 | 0·00 | 0·34 | 0·69 | -7·39 |
| SPARCL1 | -0·00 | 0·34 | 0·69 | -7·39 |
| MGLL | 0·00 | 0·34 | 0·69 | -7·39 |
| LSP1 | -0·00 | 0·34 | 0·69 | -7·39 |
| CST7 | 0·00 | 0·34 | 0·69 | -7·39 |
| CA4 | -0·00 | 0·34 | 0·69 | -7·39 |
| ELOA | -0·00 | 0·34 | 0·69 | -7·39 |
| AGR3 | -0·00 | 0·34 | 0·69 | -7·40 |
| ERBB3 | -0·00 | 0·34 | 0·69 | -7·40 |
| ITGA11 | -0·00 | 0·34 | 0·69 | -7·40 |
| ADAMTS13 | -0·00 | 0·35 | 0·70 | -7·40 |
| SCG3 | -0·00 | 0·35 | 0·70 | -7·41 |
| THBS2 | -0·00 | 0·35 | 0·70 | -7·41 |
| ATOX1 | 0·00 | 0·35 | 0·71 | -7·41 |
| CD99L2 | 0·00 | 0·35 | 0·71 | -7·42 |
| PLAUR | 0·00 | 0·35 | 0·71 | -7·42 |
| BLVRB | -0·00 | 0·35 | 0·71 | -7·42 |
| HARS | 0·00 | 0·36 | 0·71 | -7·42 |
| PPCDC | -0·00 | 0·36 | 0·71 | -7·42 |
| APOM | -0·00 | 0·36 | 0·71 | -7·43 |
| PRTN3 | 0·00 | 0·36 | 0·71 | -7·43 |
| LPO | -0·00 | 0·36 | 0·71 | -7·43 |
| EPHB4 | 0·00 | 0·36 | 0·71 | -7·43 |
| ICAM1 | 0·00 | 0·36 | 0·71 | -7·43 |
| WFDC12 | 0·00 | 0·36 | 0·71 | -7·43 |
| PRKAB1 | 0·00 | 0·37 | 0·72 | -7·44 |
| ARHGAP25 | -0·00 | 0·37 | 0·72 | -7·44 |
| RHOC | 0·00 | 0·37 | 0·72 | -7·44 |
| CLC | -0·00 | 0·37 | 0·72 | -7·44 |
| FBP1 | 0·00 | 0·37 | 0·72 | -7·45 |
| CD28 | -0·00 | 0·37 | 0·72 | -7·45 |
| CXCL12 | 0·00 | 0·37 | 0·72 | -7·45 |
| DCTN1 | 0·00 | 0·37 | 0·72 | -7·45 |
| FETUB | -0·00 | 0·37 | 0·72 | -7·45 |
| NECTIN4 | -0·00 | 0·37 | 0·72 | -7·45 |
| PDLIM7 | 0·01 | 0·37 | 0·72 | -7·45 |
| VPS37A | 0·00 | 0·37 | 0·72 | -7·45 |
| ATG4A | -0·00 | 0·37 | 0·72 | -7·45 |
| USO1 | 0·00 | 0·37 | 0·72 | -7·45 |
| CLUL1 | -0·00 | 0·38 | 0·73 | -7·46 |
| PAM | -0·00 | 0·38 | 0·73 | -7·46 |
| IL1B | -0·00 | 0·38 | 0·73 | -7·46 |
| SIGLEC9 | 0·00 | 0·38 | 0·73 | -7·46 |
| CD38 | 0·00 | 0·38 | 0·73 | -7·46 |
| CLEC1B | 0·00 | 0·38 | 0·73 | -7·46 |
| NECTIN2 | 0·00 | 0·38 | 0·73 | -7·47 |
| CD40LG | 0·00 | 0·38 | 0·73 | -7·47 |
| CRKL | 0·00 | 0·38 | 0·73 | -7·47 |
| CA13 | 0·00 | 0·38 | 0·73 | -7·47 |
| LRRC25 | -0·00 | 0·39 | 0·73 | -7·47 |
| FCGR2A | -0·00 | 0·39 | 0·73 | -7·47 |
| ITGAM | 0·00 | 0·39 | 0·73 | -7·47 |
| ESM1 | -0·00 | 0·39 | 0·73 | -7·47 |
| B4GALT1 | 0·00 | 0·39 | 0·73 | -7·47 |
| CTSL | 0·00 | 0·39 | 0·73 | -7·48 |
| NUDT5 | 0·00 | 0·39 | 0·73 | -7·48 |
| CD99 | 0·00 | 0·39 | 0·74 | -7·48 |
| CTSB | 0·00 | 0·39 | 0·74 | -7·48 |
| PDCD6 | 0·00 | 0·39 | 0·74 | -7·48 |
| CDKN2D | 0·00 | 0·39 | 0·74 | -7·48 |
| CA11 | -0·00 | 0·39 | 0·74 | -7·48 |
| LGALS8 | 0·00 | 0·39 | 0·74 | -7·49 |
| ACE2 | 0·00 | 0·39 | 0·74 | -7·49 |
| PTS | -0·00 | 0·40 | 0·74 | -7·49 |
| REG1A | 0·00 | 0·40 | 0·74 | -7·49 |
| GLB1 | 0·00 | 0·40 | 0·74 | -7·49 |
| SEMA4C | 0·00 | 0·40 | 0·74 | -7·49 |
| CD55 | 0·00 | 0·40 | 0·74 | -7·50 |
| FOLR1 | 0·00 | 0·40 | 0·74 | -7·50 |
| CD300LF | 0·00 | 0·40 | 0·74 | -7·50 |
| WWP2 | -0·00 | 0·40 | 0·75 | -7·50 |
| DNAJB1 | -0·00 | 0·41 | 0·75 | -7·50 |
| PARP1 | -0·00 | 0·41 | 0·75 | -7·50 |
| SSC4D | 0·00 | 0·41 | 0·75 | -7·50 |
| THPO | 0·00 | 0·41 | 0·75 | -7·51 |
| SNX9 | 0·00 | 0·41 | 0·75 | -7·51 |
| CRIP2 | 0·00 | 0·41 | 0·75 | -7·51 |
| CD177 | -0·00 | 0·41 | 0·75 | -7·51 |
| CLEC4D | 0·00 | 0·41 | 0·75 | -7·51 |
| SCLY | -0·00 | 0·41 | 0·75 | -7·51 |
| TRIM5 | 0·00 | 0·41 | 0·75 | -7·52 |
| CLEC7A | 0·00 | 0·42 | 0·76 | -7·52 |
| GUSB | 0·00 | 0·42 | 0·76 | -7·52 |
| MYOC | -0·00 | 0·42 | 0·76 | -7·52 |
| CALCOCO1 | 0·00 | 0·42 | 0·76 | -7·52 |
| TXLNA | 0·00 | 0·42 | 0·76 | -7·52 |
| CCL14 | 0·00 | 0·42 | 0·76 | -7·53 |
| CD160 | 0·00 | 0·42 | 0·76 | -7·53 |
| AKT1S1 | -0·00 | 0·43 | 0·76 | -7·53 |
| IGFBP1 | 0·00 | 0·43 | 0·76 | -7·53 |
| AZU1 | -0·00 | 0·43 | 0·76 | -7·53 |
| SAMD9L | 0·00 | 0·43 | 0·76 | -7·53 |
| LAMA4 | -0·00 | 0·43 | 0·76 | -7·53 |
| PADI2 | 0·00 | 0·43 | 0·76 | -7·53 |
| CCL26 | 0·00 | 0·43 | 0·77 | -7·54 |
| DPP6 | 0·00 | 0·43 | 0·77 | -7·54 |
| FRZB | -0·00 | 0·43 | 0·77 | -7·54 |
| NUCB2 | 0·00 | 0·43 | 0·77 | -7·54 |
| SELE | 0·00 | 0·44 | 0·77 | -7·54 |
| NFKBIE | -0·00 | 0·44 | 0·77 | -7·54 |
| GP1BA | -0·00 | 0·44 | 0·77 | -7·55 |
| NOTCH1 | -0·00 | 0·44 | 0·78 | -7·55 |
| FGR | -0·00 | 0·44 | 0·78 | -7·55 |
| GPNMB | -0·00 | 0·44 | 0·78 | -7·55 |
| GRAP2 | 0·00 | 0·44 | 0·78 | -7·55 |
| IL10RA | -0·00 | 0·44 | 0·78 | -7·55 |
| DEFA1_DEFA1B | 0·00 | 0·44 | 0·78 | -7·55 |
| PIK3AP1 | -0·00 | 0·44 | 0·78 | -7·56 |
| DNPH1 | -0·00 | 0·44 | 0·78 | -7·56 |
| NAMPT | -0·00 | 0·45 | 0·78 | -7·56 |
| ATXN10 | 0·00 | 0·45 | 0·78 | -7·56 |
| NT5C3A | 0·00 | 0·45 | 0·78 | -7·56 |
| MILR1 | 0·00 | 0·45 | 0·78 | -7·56 |
| BACH1 | -0·00 | 0·45 | 0·78 | -7·56 |
| NTRK2 | -0·00 | 0·45 | 0·78 | -7·56 |
| NTRK3 | -0·00 | 0·45 | 0·78 | -7·56 |
| TXNDC15 | 0·00 | 0·45 | 0·78 | -7·56 |
| CTSO | -0·00 | 0·45 | 0·78 | -7·56 |
| CREG1 | 0·00 | 0·45 | 0·78 | -7·56 |
| MANF | 0·00 | 0·45 | 0·78 | -7·57 |
| RGMB | 0·00 | 0·45 | 0·78 | -7·57 |
| PDGFC | -0·00 | 0·45 | 0·78 | -7·57 |
| CD46 | 0·00 | 0·46 | 0·78 | -7·57 |
| SUSD2 | 0·00 | 0·46 | 0·78 | -7·57 |
| ENAH | 0·00 | 0·46 | 0·78 | -7·57 |
| PXN | -0·00 | 0·46 | 0·78 | -7·57 |
| TANK | 0·00 | 0·46 | 0·78 | -7·57 |
| CST5 | 0·00 | 0·46 | 0·79 | -7·58 |
| PLA2G7 | 0·00 | 0·46 | 0·79 | -7·58 |
| EIF4B | -0·00 | 0·47 | 0·79 | -7·58 |
| CIAPIN1 | 0·00 | 0·47 | 0·79 | -7·58 |
| MSMB | 0·00 | 0·47 | 0·79 | -7·58 |
| METAP1D | 0·00 | 0·47 | 0·79 | -7·58 |
| CTSZ | 0·00 | 0·47 | 0·79 | -7·59 |
| CDHR5 | 0·00 | 0·47 | 0·79 | -7·59 |
| LAT | 0·00 | 0·47 | 0·79 | -7·59 |
| TST | 0·00 | 0·47 | 0·79 | -7·59 |
| FKBP4 | -0·00 | 0·47 | 0·79 | -7·59 |
| UBAC1 | -0·00 | 0·47 | 0·79 | -7·59 |
| CNTN1 | -0·00 | 0·47 | 0·79 | -7·59 |
| ANGPT2 | 0·00 | 0·47 | 0·79 | -7·59 |
| OSM | 0·00 | 0·47 | 0·79 | -7·59 |
| PECAM1 | 0·00 | 0·47 | 0·79 | -7·59 |
| EDAR | 0·00 | 0·47 | 0·79 | -7·59 |
| GGT1 | 0·00 | 0·47 | 0·79 | -7·59 |
| TNFSF13B | 0·00 | 0·47 | 0·79 | -7·59 |
| ENO2 | 0·00 | 0·48 | 0·79 | -7·59 |
| LGALS7_LGALS7B | 0·00 | 0·48 | 0·79 | -7·59 |
| SNAP29 | 0·00 | 0·48 | 0·79 | -7·59 |
| HYAL1 | 0·00 | 0·48 | 0·79 | -7·59 |
| HLA-E | 0·00 | 0·48 | 0·79 | -7·60 |
| FCRL5 | 0·00 | 0·48 | 0·79 | -7·60 |
| FCER2 | 0·00 | 0·48 | 0·79 | -7·60 |
| NBN | -0·00 | 0·48 | 0·79 | -7·60 |
| TINAGL1 | 0·00 | 0·48 | 0·79 | -7·60 |
| KLK1 | -0·00 | 0·48 | 0·79 | -7·60 |
| CHL1 | -0·00 | 0·48 | 0·79 | -7·60 |
| TBC1D23 | 0·00 | 0·48 | 0·79 | -7·60 |
| CALCA | 0·00 | 0·48 | 0·79 | -7·60 |
| OLR1 | -0·00 | 0·49 | 0·80 | -7·61 |
| LILRB1 | 0·00 | 0·49 | 0·80 | -7·61 |
| EGFL7 | 0·00 | 0·49 | 0·80 | -7·61 |
| MMP10 | 0·00 | 0·49 | 0·80 | -7·61 |
| ERBB4 | 0·00 | 0·49 | 0·80 | -7·61 |
| ABL1 | 0·00 | 0·49 | 0·80 | -7·61 |
| ILKAP | -0·00 | 0·49 | 0·80 | -7·61 |
| DPT | 0·00 | 0·49 | 0·80 | -7·61 |
| BAIAP2 | 0·00 | 0·49 | 0·80 | -7·61 |
| SRC | 0·00 | 0·50 | 0·80 | -7·62 |
| ENTPD6 | -0·00 | 0·50 | 0·80 | -7·62 |
| DKK1 | 0·00 | 0·50 | 0·80 | -7·62 |
| SH2D1A | -0·00 | 0·50 | 0·80 | -7·62 |
| SUSD1 | 0·00 | 0·50 | 0·80 | -7·62 |
| CCL21 | -0·00 | 0·50 | 0·80 | -7·62 |
| ACAN | -0·00 | 0·50 | 0·80 | -7·62 |
| IDS | -0·00 | 0·50 | 0·80 | -7·62 |
| QPCT | -0·00 | 0·50 | 0·80 | -7·62 |
| LBR | 0·00 | 0·50 | 0·80 | -7·62 |
| HSPG2 | 0·00 | 0·50 | 0·80 | -7·62 |
| SERPINB1 | -0·00 | 0·50 | 0·80 | -7·62 |
| CASP10 | -0·00 | 0·50 | 0·80 | -7·62 |
| RUVBL1 | -0·00 | 0·50 | 0·80 | -7·62 |
| EGF | 0·00 | 0·51 | 0·80 | -7·63 |
| GRPEL1 | 0·00 | 0·51 | 0·80 | -7·63 |
| ATP5IF1 | 0·00 | 0·51 | 0·80 | -7·63 |
| MPO | 0·00 | 0·51 | 0·80 | -7·63 |
| SERPINB9 | -0·00 | 0·51 | 0·81 | -7·63 |
| IDUA | 0·00 | 0·51 | 0·81 | -7·63 |
| HMBS | -0·00 | 0·51 | 0·81 | -7·63 |
| MIA | -0·00 | 0·51 | 0·81 | -7·63 |
| HNRNPK | -0·00 | 0·52 | 0·81 | -7·64 |
| PLXNA4 | 0·00 | 0·52 | 0·81 | -7·64 |
| EDIL3 | -0·00 | 0·52 | 0·81 | -7·64 |
| MANSC1 | -0·00 | 0·52 | 0·82 | -7·64 |
| ALDH1A1 | -0·00 | 0·52 | 0·82 | -7·64 |
| SELPLG | -0·00 | 0·52 | 0·82 | -7·64 |
| MSLN | 0·00 | 0·52 | 0·82 | -7·64 |
| SOD1 | -0·00 | 0·53 | 0·82 | -7·65 |
| NELL1 | -0·00 | 0·53 | 0·82 | -7·65 |
| ITM2A | 0·00 | 0·53 | 0·82 | -7·65 |
| HEXIM1 | 0·00 | 0·53 | 0·82 | -7·65 |
| AMN | 0·00 | 0·53 | 0·82 | -7·65 |
| DOK2 | 0·00 | 0·53 | 0·82 | -7·65 |
| LILRA2 | 0·00 | 0·53 | 0·82 | -7·65 |
| TNFSF10 | 0·00 | 0·53 | 0·82 | -7·65 |
| RBKS | -0·00 | 0·53 | 0·82 | -7·65 |
| CD14 | 0·00 | 0·53 | 0·82 | -7·65 |
| CCN2 | 0·00 | 0·53 | 0·82 | -7·65 |
| C19orf12 | 0·00 | 0·53 | 0·82 | -7·65 |
| CNPY4 | 0·00 | 0·53 | 0·82 | -7·65 |
| BCL2L11 | 0·00 | 0·53 | 0·82 | -7·65 |
| BST2 | 0·00 | 0·53 | 0·82 | -7·65 |
| PLA2G10 | 0·00 | 0·53 | 0·82 | -7·65 |
| TBCB | 0·00 | 0·54 | 0·82 | -7·66 |
| VCAM1 | 0·00 | 0·54 | 0·82 | -7·66 |
| PPP3R1 | 0·00 | 0·54 | 0·82 | -7·66 |
| CD2AP | -0·00 | 0·54 | 0·82 | -7·66 |
| PPIB | 0·00 | 0·54 | 0·82 | -7·66 |
| DLL1 | 0·00 | 0·54 | 0·82 | -7·66 |
| RNF41 | -0·00 | 0·54 | 0·82 | -7·66 |
| SIT1 | -0·00 | 0·54 | 0·82 | -7·66 |
| SPP1 | -0·00 | 0·54 | 0·82 | -7·66 |
| FYB1 | 0·00 | 0·54 | 0·82 | -7·66 |
| HSPB1 | 0·00 | 0·54 | 0·82 | -7·66 |
| RP2 | -0·00 | 0·54 | 0·82 | -7·66 |
| TJAP1 | 0·00 | 0·54 | 0·82 | -7·66 |
| BMP6 | 0·00 | 0·55 | 0·83 | -7·67 |
| CLEC4C | -0·00 | 0·55 | 0·83 | -7·67 |
| S100P | -0·00 | 0·55 | 0·83 | -7·67 |
| STX6 | 0·00 | 0·55 | 0·83 | -7·67 |
| RWDD1 | 0·00 | 0·55 | 0·83 | -7·67 |
| B4GAT1 | -0·00 | 0·55 | 0·83 | -7·67 |
| ITGB7 | -0·00 | 0·56 | 0·84 | -7·68 |
| IPCEF1 | -0·00 | 0·56 | 0·84 | -7·68 |
| PLA2G4A | 0·00 | 0·56 | 0·84 | -7·68 |
| IL1RL1 | -0·00 | 0·56 | 0·84 | -7·68 |
| RNASE3 | -0·00 | 0·57 | 0·84 | -7·68 |
| FCGR3B | -0·00 | 0·57 | 0·84 | -7·68 |
| ERBIN | 0·00 | 0·57 | 0·85 | -7·69 |
| IFNGR1 | 0·00 | 0·57 | 0·85 | -7·69 |
| GSTA1 | 0·00 | 0·57 | 0·85 | -7·69 |
| CTSD | 0·00 | 0·57 | 0·85 | -7·69 |
| INHBC | 0·00 | 0·57 | 0·85 | -7·69 |
| JUN | -0·00 | 0·57 | 0·85 | -7·69 |
| CD69 | 0·00 | 0·57 | 0·85 | -7·69 |
| ANXA4 | -0·00 | 0·57 | 0·85 | -7·69 |
| PAEP | -0·00 | 0·58 | 0·85 | -7·69 |
| PLPBP | -0·00 | 0·58 | 0·85 | -7·69 |
| SLC39A14 | 0·00 | 0·58 | 0·85 | -7·69 |
| IVD | 0·00 | 0·58 | 0·85 | -7·69 |
| TIA1 | 0·00 | 0·58 | 0·85 | -7·69 |
| CD93 | -0·00 | 0·58 | 0·85 | -7·69 |
| CXCL6 | 0·00 | 0·58 | 0·85 | -7·69 |
| FCN2 | -0·00 | 0·58 | 0·85 | -7·69 |
| MVK | -0·00 | 0·58 | 0·85 | -7·70 |
| MCFD2 | 0·00 | 0·58 | 0·85 | -7·70 |
| PLIN3 | 0·00 | 0·58 | 0·85 | -7·70 |
| PRTG | -0·00 | 0·58 | 0·85 | -7·70 |
| TLR3 | -0·00 | 0·59 | 0·85 | -7·70 |
| PDCD1LG2 | 0·00 | 0·59 | 0·85 | -7·70 |
| TIMD4 | -0·00 | 0·59 | 0·85 | -7·70 |
| PPP1R12A | 0·00 | 0·59 | 0·85 | -7·70 |
| CPXM1 | -0·00 | 0·59 | 0·85 | -7·70 |
| CNTNAP2 | 0·00 | 0·59 | 0·86 | -7·70 |
| CCL20 | -0·00 | 0·59 | 0·86 | -7·70 |
| PCSK9 | 0·00 | 0·59 | 0·86 | -7·71 |
| TNFRSF21 | -0·00 | 0·59 | 0·86 | -7·71 |
| PTPRN2 | -0·00 | 0·59 | 0·86 | -7·71 |
| LY6D | 0·00 | 0·60 | 0·86 | -7·71 |
| HAGH | -0·00 | 0·60 | 0·86 | -7·71 |
| NOS3 | -0·00 | 0·60 | 0·86 | -7·71 |
| ITGB1BP2 | 0·00 | 0·60 | 0·86 | -7·71 |
| NPTX1 | -0·00 | 0·60 | 0·86 | -7·71 |
| NRP2 | 0·00 | 0·60 | 0·86 | -7·71 |
| SERPINB6 | 0·00 | 0·60 | 0·86 | -7·71 |
| TRAF2 | -0·00 | 0·60 | 0·86 | -7·71 |
| TIMP3 | -0·00 | 0·60 | 0·86 | -7·71 |
| SLIT2 | -0·00 | 0·60 | 0·86 | -7·71 |
| FOXO1 | -0·00 | 0·61 | 0·86 | -7·71 |
| SEMA4D | -0·00 | 0·61 | 0·86 | -7·71 |
| NCK2 | 0·00 | 0·61 | 0·86 | -7·71 |
| FUCA1 | 0·00 | 0·61 | 0·86 | -7·72 |
| STK11 | 0·00 | 0·61 | 0·86 | -7·72 |
| MAP4K5 | 0·00 | 0·61 | 0·87 | -7·72 |
| GPC5 | -0·00 | 0·61 | 0·87 | -7·72 |
| DBNL | 0·00 | 0·61 | 0·87 | -7·72 |
| CDKN1A | 0·00 | 0·62 | 0·87 | -7·72 |
| CD209 | 0·00 | 0·62 | 0·87 | -7·72 |
| DIABLO | 0·00 | 0·62 | 0·87 | -7·72 |
| CDH17 | 0·00 | 0·62 | 0·87 | -7·72 |
| LPL | -0·00 | 0·62 | 0·87 | -7·72 |
| PRSS2 | 0·00 | 0·62 | 0·87 | -7·72 |
| CAMKK1 | -0·00 | 0·62 | 0·87 | -7·73 |
| SIGLEC15 | -0·00 | 0·62 | 0·87 | -7·73 |
| TPMT | 0·00 | 0·62 | 0·87 | -7·73 |
| GHRL | 0·00 | 0·63 | 0·87 | -7·73 |
| SETMAR | 0·00 | 0·63 | 0·87 | -7·73 |
| SMPDL3A | 0·00 | 0·63 | 0·87 | -7·73 |
| SCAMP3 | 0·00 | 0·63 | 0·87 | -7·73 |
| AREG | 0·00 | 0·63 | 0·87 | -7·73 |
| KRT19 | 0·00 | 0·63 | 0·87 | -7·73 |
| TNFRSF9 | 0·00 | 0·63 | 0·87 | -7·73 |
| PFKFB2 | -0·00 | 0·63 | 0·87 | -7·73 |
| FCAR | 0·00 | 0·63 | 0·87 | -7·73 |
| RABGAP1L | -0·00 | 0·63 | 0·87 | -7·73 |
| SMOC2 | 0·00 | 0·63 | 0·87 | -7·73 |
| CDC37 | 0·00 | 0·63 | 0·87 | -7·73 |
| ANXA3 | -0·00 | 0·63 | 0·87 | -7·73 |
| DNER | -0·00 | 0·63 | 0·87 | -7·73 |
| CPPED1 | -0·00 | 0·63 | 0·87 | -7·73 |
| BCR | 0·00 | 0·63 | 0·87 | -7·73 |
| CD244 | 0·00 | 0·63 | 0·87 | -7·73 |
| CEACAM5 | -0·00 | 0·64 | 0·87 | -7·73 |
| MPIG6B | 0·00 | 0·64 | 0·87 | -7·73 |
| FADD | 0·00 | 0·64 | 0·87 | -7·73 |
| GCG | 0·00 | 0·64 | 0·87 | -7·74 |
| GUCA2A | 0·00 | 0·64 | 0·87 | -7·74 |
| ASAH2 | -0·00 | 0·64 | 0·87 | -7·74 |
| CD164 | -0·00 | 0·64 | 0·87 | -7·74 |
| IL16 | 0·00 | 0·64 | 0·87 | -7·74 |
| DPP7 | 0·00 | 0·64 | 0·87 | -7·74 |
| PSME1 | -0·00 | 0·64 | 0·87 | -7·74 |
| IL20 | -0·00 | 0·64 | 0·87 | -7·74 |
| AIFM1 | 0·00 | 0·64 | 0·87 | -7·74 |
| VCAN | 0·00 | 0·65 | 0·87 | -7·74 |
| PVR | 0·00 | 0·65 | 0·87 | -7·74 |
| SEMA3F | 0·00 | 0·65 | 0·87 | -7·74 |
| CDH1 | 0·00 | 0·65 | 0·87 | -7·74 |
| OSCAR | 0·00 | 0·65 | 0·87 | -7·74 |
| ANKRD54 | -0·00 | 0·65 | 0·87 | -7·74 |
| ERBB2 | -0·00 | 0·65 | 0·87 | -7·74 |
| LAMP2 | -0·00 | 0·65 | 0·87 | -7·74 |
| HGS | -0·00 | 0·65 | 0·87 | -7·74 |
| KLK11 | -0·00 | 0·65 | 0·88 | -7·75 |
| SLITRK6 | -0·00 | 0·65 | 0·88 | -7·75 |
| CD63 | 0·00 | 0·65 | 0·88 | -7·75 |
| PDCD5 | 0·00 | 0·65 | 0·88 | -7·75 |
| CD5 | 0·00 | 0·66 | 0·88 | -7·75 |
| FOLR3 | 0·00 | 0·66 | 0·88 | -7·75 |
| ROR1 | -0·00 | 0·66 | 0·88 | -7·75 |
| ALPP | 0·00 | 0·66 | 0·88 | -7·75 |
| GFER | -0·00 | 0·67 | 0·88 | -7·75 |
| FGF23 | 0·00 | 0·67 | 0·88 | -7·75 |
| HBEGF | 0·00 | 0·67 | 0·88 | -7·75 |
| MUC16 | -0·00 | 0·67 | 0·88 | -7·75 |
| IRAK1 | 0·00 | 0·67 | 0·88 | -7·75 |
| CA14 | -0·00 | 0·67 | 0·88 | -7·76 |
| FCRL3 | -0·00 | 0·67 | 0·88 | -7·76 |
| CD109 | -0·00 | 0·67 | 0·88 | -7·76 |
| MAX | 0·00 | 0·67 | 0·88 | -7·76 |
| TNFSF12 | -0·00 | 0·68 | 0·89 | -7·76 |
| SIRPA | 0·00 | 0·68 | 0·89 | -7·76 |
| IRAK4 | 0·00 | 0·68 | 0·89 | -7·76 |
| TFF3 | 0·00 | 0·68 | 0·89 | -7·76 |
| PARK7 | 0·00 | 0·68 | 0·89 | -7·76 |
| ZBTB16 | 0·00 | 0·68 | 0·89 | -7·76 |
| F11R | 0·00 | 0·68 | 0·89 | -7·76 |
| DARS | 0·00 | 0·68 | 0·89 | -7·76 |
| GGH | 0·00 | 0·69 | 0·90 | -7·77 |
| CLPP | 0·00 | 0·69 | 0·90 | -7·77 |
| REN | 0·00 | 0·69 | 0·90 | -7·77 |
| PPP1R2 | 0·00 | 0·69 | 0·90 | -7·77 |
| FAM3B | -0·00 | 0·69 | 0·90 | -7·77 |
| BRK1 | 0·00 | 0·69 | 0·90 | -7·77 |
| IL20RA | -0·00 | 0·69 | 0·90 | -7·77 |
| PDGFRA | -0·00 | 0·69 | 0·90 | -7·77 |
| XPNPEP2 | -0·00 | 0·70 | 0·90 | -7·77 |
| TXNRD1 | 0·00 | 0·70 | 0·90 | -7·77 |
| TBL1X | 0·00 | 0·70 | 0·90 | -7·77 |
| YES1 | 0·00 | 0·70 | 0·90 | -7·77 |
| ARSB | 0·00 | 0·70 | 0·90 | -7·77 |
| ADAM22 | 0·00 | 0·70 | 0·90 | -7·77 |
| CNST | -0·00 | 0·70 | 0·90 | -7·77 |
| BAX | -0·00 | 0·70 | 0·90 | -7·77 |
| GKN1 | -0·00 | 0·70 | 0·90 | -7·77 |
| MATN2 | -0·00 | 0·70 | 0·90 | -7·77 |
| CRIM1 | 0·00 | 0·70 | 0·90 | -7·77 |
| NADK | 0·00 | 0·70 | 0·90 | -7·77 |
| VEGFD | 0·00 | 0·70 | 0·90 | -7·77 |
| VPS53 | 0·00 | 0·70 | 0·90 | -7·77 |
| CD22 | -0·00 | 0·70 | 0·90 | -7·78 |
| FUT8 | -0·00 | 0·71 | 0·90 | -7·78 |
| BSG | 0·00 | 0·71 | 0·90 | -7·78 |
| WARS | 0·00 | 0·71 | 0·91 | -7·78 |
| CCL22 | -0·00 | 0·71 | 0·91 | -7·78 |
| CPA1 | 0·00 | 0·71 | 0·91 | -7·78 |
| PNLIPRP2 | -0·01 | 0·71 | 0·91 | -7·78 |
| IL7 | 0·00 | 0·71 | 0·91 | -7·78 |
| PI3 | -0·00 | 0·72 | 0·91 | -7·78 |
| ADH4 | 0·00 | 0·72 | 0·91 | -7·78 |
| RILP | 0·00 | 0·72 | 0·91 | -7·78 |
| CKMT1A_CKMT1B | -0·00 | 0·72 | 0·91 | -7·78 |
| BANK1 | -0·00 | 0·72 | 0·91 | -7·78 |
| TBC1D5 | 0·00 | 0·72 | 0·91 | -7·78 |
| CA12 | -0·00 | 0·72 | 0·91 | -7·78 |
| APBB1IP | -0·00 | 0·72 | 0·91 | -7·78 |
| VASN | -0·00 | 0·72 | 0·91 | -7·78 |
| TNFRSF13C | 0·00 | 0·73 | 0·91 | -7·79 |
| AOC3 | 0·00 | 0·73 | 0·91 | -7·79 |
| UMOD | -0·00 | 0·73 | 0·91 | -7·79 |
| NID2 | 0·00 | 0·73 | 0·91 | -7·79 |
| SKAP2 | 0·00 | 0·74 | 0·92 | -7·79 |
| LACTB2 | 0·00 | 0·74 | 0·92 | -7·79 |
| SLC16A1 | -0·00 | 0·74 | 0·92 | -7·79 |
| IL32 | 0·00 | 0·74 | 0·92 | -7·79 |
| PCOLCE | 0·00 | 0·74 | 0·92 | -7·79 |
| DKK4 | -0·00 | 0·74 | 0·92 | -7·79 |
| STX8 | -0·00 | 0·74 | 0·92 | -7·79 |
| CXCL1 | -0·00 | 0·74 | 0·92 | -7·79 |
| GNLY | 0·00 | 0·74 | 0·92 | -7·79 |
| SLC39A5 | 0·00 | 0·74 | 0·92 | -7·79 |
| AKR1C4 | -0·00 | 0·75 | 0·92 | -7·79 |
| MAP2K6 | 0·00 | 0·75 | 0·92 | -7·79 |
| SPRY2 | 0·00 | 0·75 | 0·92 | -7·79 |
| GOPC | 0·00 | 0·75 | 0·92 | -7·79 |
| HMOX2 | -0·00 | 0·75 | 0·92 | -7·79 |
| CCL5 | -0·00 | 0·75 | 0·92 | -7·79 |
| RTBDN | 0·00 | 0·75 | 0·92 | -7·80 |
| CHAC2 | 0·00 | 0·75 | 0·92 | -7·80 |
| SDC4 | -0·00 | 0·75 | 0·92 | -7·80 |
| DNMBP | 0·00 | 0·75 | 0·92 | -7·80 |
| CLEC11A | 0·00 | 0·75 | 0·92 | -7·80 |
| MIF | -0·00 | 0·75 | 0·92 | -7·80 |
| MARCO | 0·00 | 0·75 | 0·92 | -7·80 |
| TGFBI | -0·00 | 0·75 | 0·92 | -7·80 |
| ENO1 | -0·00 | 0·75 | 0·92 | -7·80 |
| CXCL13 | -0·00 | 0·75 | 0·92 | -7·80 |
| ADA2 | 0·00 | 0·76 | 0·92 | -7·80 |
| ISM1 | -0·00 | 0·76 | 0·92 | -7·80 |
| PRDX6 | 0·00 | 0·76 | 0·92 | -7·80 |
| PTX3 | 0·00 | 0·76 | 0·92 | -7·80 |
| IL34 | 0·00 | 0·76 | 0·92 | -7·80 |
| AIF1 | 0·00 | 0·76 | 0·92 | -7·80 |
| STAMBP | -0·00 | 0·76 | 0·93 | -7·80 |
| CPA2 | 0·00 | 0·76 | 0·93 | -7·80 |
| PCDH17 | -0·00 | 0·76 | 0·93 | -7·80 |
| STAT5B | 0·00 | 0·76 | 0·93 | -7·80 |
| LGMN | 0·00 | 0·77 | 0·93 | -7·80 |
| CD8A | -0·00 | 0·77 | 0·93 | -7·80 |
| NRCAM | -0·00 | 0·77 | 0·93 | -7·80 |
| DPEP2 | -0·00 | 0·77 | 0·93 | -7·80 |
| ANGPTL1 | -0·00 | 0·77 | 0·94 | -7·81 |
| FOXO3 | -0·00 | 0·78 | 0·94 | -7·81 |
| TPPP3 | -0·00 | 0·78 | 0·94 | -7·81 |
| HMOX1 | 0·00 | 0·78 | 0·94 | -7·81 |
| THBD | 0·00 | 0·78 | 0·94 | -7·81 |
| AMBN | -0·00 | 0·78 | 0·94 | -7·81 |
| JCHAIN | 0·00 | 0·78 | 0·94 | -7·81 |
| ADAM23 | -0·00 | 0·78 | 0·94 | -7·81 |
| ACY1 | 0·00 | 0·78 | 0·94 | -7·81 |
| KYNU | -0·00 | 0·79 | 0·94 | -7·81 |
| IL1RN | 0·00 | 0·79 | 0·94 | -7·81 |
| ENPP2 | 0·00 | 0·79 | 0·94 | -7·81 |
| ERP44 | 0·00 | 0·79 | 0·94 | -7·81 |
| SUMF2 | 0·00 | 0·79 | 0·94 | -7·81 |
| SFTPA2 | -0·00 | 0·79 | 0·94 | -7·81 |
| APRT | 0·00 | 0·79 | 0·94 | -7·81 |
| IFNLR1 | -0·00 | 0·79 | 0·94 | -7·81 |
| VEGFC | 0·00 | 0·79 | 0·94 | -7·81 |
| NUB1 | -0·00 | 0·79 | 0·94 | -7·81 |
| FIS1 | -0·00 | 0·79 | 0·94 | -7·81 |
| HSPA1A | 0·00 | 0·79 | 0·94 | -7·81 |
| SNAP23 | 0·00 | 0·80 | 0·94 | -7·81 |
| FLRT2 | -0·00 | 0·80 | 0·95 | -7·81 |
| CXCL3 | 0·00 | 0·80 | 0·95 | -7·81 |
| EBAG9 | 0·00 | 0·80 | 0·95 | -7·82 |
| ARHGEF12 | 0·00 | 0·81 | 0·95 | -7·82 |
| NT5E | 0·00 | 0·81 | 0·95 | -7·82 |
| DCTN2 | -0·00 | 0·81 | 0·95 | -7·82 |
| CLEC5A | 0·00 | 0·81 | 0·95 | -7·82 |
| BGN | -0·00 | 0·81 | 0·95 | -7·82 |
| RETN | -0·00 | 0·81 | 0·95 | -7·82 |
| RAD23B | -0·00 | 0·81 | 0·95 | -7·82 |
| NFATC1 | -0·00 | 0·81 | 0·95 | -7·82 |
| CCL28 | 0·00 | 0·82 | 0·95 | -7·82 |
| AARSD1 | 0·00 | 0·82 | 0·95 | -7·82 |
| CEACAM1 | -0·00 | 0·82 | 0·95 | -7·82 |
| FHIT | 0·00 | 0·82 | 0·95 | -7·82 |
| KEL | -0·00 | 0·82 | 0·95 | -7·82 |
| FGF19 | 0·00 | 0·82 | 0·95 | -7·82 |
| SDC1 | -0·00 | 0·82 | 0·95 | -7·82 |
| SCARF1 | 0·00 | 0·82 | 0·95 | -7·82 |
| PRKRA | 0·00 | 0·82 | 0·95 | -7·82 |
| RAB6A | 0·00 | 0·82 | 0·95 | -7·82 |
| DRG2 | 0·00 | 0·82 | 0·95 | -7·82 |
| CD200R1 | -0·00 | 0·82 | 0·95 | -7·82 |
| BAG6 | 0·00 | 0·82 | 0·95 | -7·82 |
| GYS1 | 0·00 | 0·82 | 0·95 | -7·82 |
| STIP1 | 0·00 | 0·83 | 0·95 | -7·82 |
| NXPH1 | -0·00 | 0·83 | 0·95 | -7·82 |
| SULT1A1 | -0·00 | 0·83 | 0·95 | -7·82 |
| LEPR | 0·00 | 0·83 | 0·95 | -7·82 |
| NRP1 | -0·00 | 0·83 | 0·95 | -7·82 |
| VWF | 0·00 | 0·83 | 0·95 | -7·82 |
| GLOD4 | -0·00 | 0·83 | 0·95 | -7·82 |
| FGFBP1 | -0·00 | 0·83 | 0·95 | -7·82 |
| HTRA2 | 0·00 | 0·83 | 0·95 | -7·82 |
| CDH5 | -0·00 | 0·83 | 0·95 | -7·82 |
| SLITRK2 | 0·00 | 0·83 | 0·95 | -7·82 |
| TBCC | 0·00 | 0·84 | 0·96 | -7·83 |
| CC2D1A | 0·00 | 0·84 | 0·96 | -7·83 |
| ROBO2 | -0·00 | 0·84 | 0·96 | -7·83 |
| PTPRM | -0·00 | 0·84 | 0·96 | -7·83 |
| IL7R | -0·00 | 0·84 | 0·96 | -7·83 |
| SCGN | 0·00 | 0·84 | 0·96 | -7·83 |
| KITLG | 0·00 | 0·84 | 0·96 | -7·83 |
| ICA1 | 0·00 | 0·84 | 0·96 | -7·83 |
| TGFBR3 | -0·00 | 0·84 | 0·96 | -7·83 |
| CD6 | -0·00 | 0·84 | 0·96 | -7·83 |
| ALCAM | 0·00 | 0·84 | 0·96 | -7·83 |
| NSFL1C | 0·00 | 0·84 | 0·96 | -7·83 |
| EPS8L2 | 0·00 | 0·85 | 0·96 | -7·83 |
| CELA3A | 0·00 | 0·85 | 0·96 | -7·83 |
| LGALS3 | 0·00 | 0·85 | 0·96 | -7·83 |
| PLAU | 0·00 | 0·85 | 0·96 | -7·83 |
| GH1 | 0·00 | 0·85 | 0·96 | -7·83 |
| IGSF3 | 0·00 | 0·86 | 0·96 | -7·83 |
| STK4 | 0·00 | 0·86 | 0·96 | -7·83 |
| SORD | -0·00 | 0·86 | 0·96 | -7·83 |
| ANXA11 | 0·00 | 0·86 | 0·96 | -7·83 |
| INPP1 | -0·00 | 0·86 | 0·96 | -7·83 |
| PHOSPHO1 | 0·00 | 0·86 | 0·96 | -7·83 |
| KLK14 | 0·00 | 0·86 | 0·96 | -7·83 |
| FABP5 | -0·00 | 0·86 | 0·96 | -7·83 |
| PGLYRP1 | -0·00 | 0·86 | 0·96 | -7·83 |
| METAP2 | 0·00 | 0·86 | 0·96 | -7·83 |
| KIR3DL1 | 0·00 | 0·86 | 0·96 | -7·83 |
| PSPN | -0·00 | 0·86 | 0·96 | -7·83 |
| IL18RAP | -0·00 | 0·87 | 0·96 | -7·83 |
| PEBP1 | 0·00 | 0·87 | 0·96 | -7·83 |
| PON2 | 0·00 | 0·87 | 0·96 | -7·83 |
| LHPP | -0·00 | 0·87 | 0·96 | -7·83 |
| YTHDF3 | -0·00 | 0·87 | 0·96 | -7·83 |
| IL17RB | 0·00 | 0·87 | 0·96 | -7·83 |
| MSRA | -0·00 | 0·87 | 0·96 | -7·83 |
| PRDX1 | -0·00 | 0·87 | 0·96 | -7·83 |
| SPOCK1 | 0·00 | 0·87 | 0·96 | -7·83 |
| RRM2 | -0·00 | 0·87 | 0·96 | -7·83 |
| DCXR | 0·00 | 0·87 | 0·96 | -7·83 |
| HCLS1 | -0·00 | 0·87 | 0·96 | -7·83 |
| CD276 | -0·00 | 0·87 | 0·96 | -7·83 |
| CHRDL2 | -0·00 | 0·87 | 0·96 | -7·83 |
| SF3B4 | 0·00 | 0·87 | 0·96 | -7·83 |
| NPY | -0·00 | 0·88 | 0·96 | -7·83 |
| CLEC4G | -0·00 | 0·88 | 0·96 | -7·83 |
| SCRN1 | -0·00 | 0·88 | 0·96 | -7·83 |
| TGFA | -0·00 | 0·88 | 0·97 | -7·83 |
| VSIR | -0·00 | 0·88 | 0·97 | -7·83 |
| C1QTNF1 | -0·00 | 0·88 | 0·97 | -7·83 |
| HPGDS | -0·00 | 0·88 | 0·97 | -7·84 |
| QDPR | -0·00 | 0·88 | 0·97 | -7·84 |
| NPTXR | -0·00 | 0·88 | 0·97 | -7·84 |
| CDSN | -0·00 | 0·88 | 0·97 | -7·84 |
| CDNF | 0·00 | 0·88 | 0·97 | -7·84 |
| MAVS | 0·00 | 0·89 | 0·97 | -7·84 |
| PTPN1 | 0·00 | 0·89 | 0·97 | -7·84 |
| STX4 | -0·00 | 0·89 | 0·97 | -7·84 |
| SIGLEC5 | 0·00 | 0·89 | 0·97 | -7·84 |
| IL6R | 0·00 | 0·89 | 0·97 | -7·84 |
| PSME2 | -0·00 | 0·89 | 0·97 | -7·84 |
| VMO1 | 0·00 | 0·89 | 0·97 | -7·84 |
| POLR2F | 0·00 | 0·89 | 0·97 | -7·84 |
| EREG | 0·00 | 0·89 | 0·97 | -7·84 |
| ANGPTL7 | 0·00 | 0·89 | 0·97 | -7·84 |
| KRT18 | 0·00 | 0·90 | 0·97 | -7·84 |
| CA5A | 0·00 | 0·90 | 0·97 | -7·84 |
| ACVRL1 | 0·00 | 0·90 | 0·97 | -7·84 |
| GGT5 | 0·00 | 0·90 | 0·97 | -7·84 |
| LRMP | -0·00 | 0·90 | 0·97 | -7·84 |
| CRISP2 | 0·00 | 0·90 | 0·97 | -7·84 |
| AMY2A | 0·00 | 0·90 | 0·97 | -7·84 |
| HPCAL1 | 0·00 | 0·90 | 0·97 | -7·84 |
| LIFR | 0·00 | 0·90 | 0·97 | -7·84 |
| MMP1 | 0·00 | 0·90 | 0·97 | -7·84 |
| KYAT1 | -0·00 | 0·90 | 0·97 | -7·84 |
| SUGT1 | -0·00 | 0·90 | 0·97 | -7·84 |
| CHMP1A | 0·00 | 0·90 | 0·97 | -7·84 |
| DEFB4A_DEFB4B | -0·00 | 0·90 | 0·97 | -7·84 |
| CD83 | -0·00 | 0·90 | 0·97 | -7·84 |
| IKBKG | 0·00 | 0·91 | 0·97 | -7·84 |
| CORO1A | 0·00 | 0·91 | 0·97 | -7·84 |
| IL10 | -0·00 | 0·91 | 0·97 | -7·84 |
| PDGFA | 0·00 | 0·91 | 0·97 | -7·84 |
| CEACAM8 | 0·00 | 0·91 | 0·97 | -7·84 |
| LY75 | -0·00 | 0·91 | 0·97 | -7·84 |
| ICAM4 | 0·00 | 0·91 | 0·97 | -7·84 |
| DDAH1 | 0·00 | 0·91 | 0·97 | -7·84 |
| CSF2RA | -0·00 | 0·91 | 0·97 | -7·84 |
| PM20D1 | 0·00 | 0·92 | 0·97 | -7·84 |
| VTA1 | 0·00 | 0·92 | 0·97 | -7·84 |
| AXIN1 | -0·00 | 0·92 | 0·97 | -7·84 |
| ARHGAP1 | -0·00 | 0·92 | 0·97 | -7·84 |
| VAT1 | 0·00 | 0·92 | 0·97 | -7·84 |
| CRADD | 0·00 | 0·92 | 0·97 | -7·84 |
| TARBP2 | 0·00 | 0·92 | 0·97 | -7·84 |
| PDGFB | -0·00 | 0·92 | 0·97 | -7·84 |
| PLXNB3 | -0·00 | 0·92 | 0·97 | -7·84 |
| PAG1 | -0·00 | 0·92 | 0·97 | -7·84 |
| CASP8 | 0·00 | 0·92 | 0·97 | -7·84 |
| MASP1 | 0·00 | 0·92 | 0·97 | -7·84 |
| SMAD1 | -0·00 | 0·92 | 0·97 | -7·84 |
| INPPL1 | -0·00 | 0·92 | 0·97 | -7·84 |
| AMY2B | 0·00 | 0·92 | 0·97 | -7·84 |
| IL6ST | 0·00 | 0·92 | 0·97 | -7·84 |
| HS3ST3B1 | -0·00 | 0·92 | 0·97 | -7·84 |
| PTPN6 | 0·00 | 0·93 | 0·97 | -7·84 |
| PLA2G1B | -0·00 | 0·93 | 0·97 | -7·84 |
| GMPR | 0·00 | 0·93 | 0·97 | -7·84 |
| ADCYAP1R1 | -0·00 | 0·93 | 0·97 | -7·84 |
| SERPINA12 | -0·00 | 0·93 | 0·97 | -7·84 |
| CD300LG | -0·00 | 0·93 | 0·97 | -7·84 |
| LAT2 | 0·00 | 0·94 | 0·97 | -7·84 |
| HAO1 | -0·00 | 0·94 | 0·97 | -7·84 |
| ITGB1 | -0·00 | 0·94 | 0·97 | -7·84 |
| CANT1 | -0·00 | 0·94 | 0·97 | -7·84 |
| PBLD | 0·00 | 0·94 | 0·97 | -7·84 |
| FGFR2 | -0·00 | 0·94 | 0·98 | -7·84 |
| TBC1D17 | 0·00 | 0·94 | 0·98 | -7·84 |
| GZMB | -0·00 | 0·94 | 0·98 | -7·84 |
| MED18 | -0·00 | 0·94 | 0·98 | -7·84 |
| MITD1 | -0·00 | 0·95 | 0·98 | -7·84 |
| COMT | -0·00 | 0·95 | 0·98 | -7·84 |
| CLEC10A | 0·00 | 0·95 | 0·98 | -7·84 |
| CAPG | 0·00 | 0·95 | 0·98 | -7·84 |
| SPARC | 0·00 | 0·95 | 0·98 | -7·84 |
| SIRT2 | 0·00 | 0·95 | 0·98 | -7·84 |
| NID1 | 0·00 | 0·96 | 0·99 | -7·84 |
| ADGRB3 | 0·00 | 0·96 | 0·99 | -7·84 |
| ECE1 | 0·00 | 0·96 | 0·99 | -7·84 |
| TSHB | -0·00 | 0·96 | 0·99 | -7·84 |
| FAP | 0·00 | 0·96 | 0·99 | -7·84 |
| LYPD8 | -0·00 | 0·96 | 0·99 | -7·84 |
| HS6ST1 | 0·00 | 0·96 | 0·99 | -7·84 |
| FLT1 | -9·63e-05 | 0·96 | 0·99 | -7·84 |
| CX3CL1 | 0·00 | 0·96 | 0·99 | -7·84 |
| TREML2 | 0·00 | 0·96 | 0·99 | -7·84 |
| SERPINB5 | -0·00 | 0·96 | 0·99 | -7·84 |
| CDH6 | -0·00 | 0·96 | 0·99 | -7·84 |
| UXS1 | 0·00 | 0·97 | 0·99 | -7·84 |
| CD33 | 0·00 | 0·97 | 0·99 | -7·84 |
| DRAXIN | -0·00 | 0·97 | 0·99 | -7·84 |
| CRACR2A | 0·00 | 0·97 | 0·99 | -7·84 |
| DFFA | -0·00 | 0·97 | 0·99 | -7·84 |
| PROC | 8·87e-05 | 0·97 | 0·99 | -7·84 |
| ZBTB17 | 7·57e-05 | 0·97 | 0·99 | -7·84 |
| FMNL1 | -0·00 | 0·97 | 0·99 | -7·84 |
| MMP8 | 0·00 | 0·97 | 0·99 | -7·84 |
| ANGPT1 | -0·00 | 0·97 | 0·99 | -7·84 |
| CA2 | 0·00 | 0·97 | 0·99 | -7·84 |
| IL18 | -0·00 | 0·98 | 0·99 | -7·84 |
| FKBP5 | -0·00 | 0·98 | 0·99 | -7·84 |
| LYN | -0·00 | 0·98 | 0·99 | -7·85 |
| AK1 | -0·00 | 0·98 | 0·99 | -7·85 |
| NMNAT1 | 7·18e-05 | 0·98 | 0·99 | -7·85 |
| CA3 | 8·2e-05 | 0·98 | 0·99 | -7·85 |
| GALNT10 | 3·59e-05 | 0·98 | 0·99 | -7·85 |
| SERPINB8 | 9·83e-05 | 0·98 | 0·99 | -7·85 |
| DECR1 | -7·86e-05 | 0·99 | 0·99 | -7·85 |
| MMP9 | -4·72e-05 | 0·99 | 0·99 | -7·85 |
| GFRA3 | 2·05e-05 | 0·99 | 0·99 | -7·85 |
| TAFA5 | -2·48e-05 | 0·99 | 0·99 | -7·85 |
| KLK6 | -2·34e-05 | 0·99 | 0·99 | -7·85 |
| TDRKH | 4·4e-05 | 0·99 | 0·99 | -7·85 |
| USP8 | 2·21e-05 | 0·99 | 0·99 | -7·85 |
| LCN2 | 9·32e-06 | 0·99 | 0·99 | -7·85 |

## Supplementary Table 4. Association results of 1293 plasma proteins with chronological age in the 200HIV cohort

| Protein | log FC | p-value | FDR | Beta |
| --- | --- | --- | --- | --- |
| EDA2R | 0·03 | 3·68e-26 | 4·75e-23 | 48·18 |
| LTBP2 | 0·02 | 1·58e-17 | 1·02e-14 | 28·40 |
| NEFL | 0·02 | 3·65e-16 | 1·57e-13 | 25·28 |
| GDF15 | 0·03 | 2·35e-15 | 6·7e-13 | 23·44 |
| KLK4 | 0·04 | 2·59e-15 | 6·7e-13 | 23·34 |
| WNT9A | 0·01 | 5·07e-15 | 1·09e-12 | 22·68 |
| SCARF2 | 0·01 | 2·33e-13 | 4·3e-11 | 18·89 |
| TSPAN1 | 0·04 | 2·89e-13 | 4·67e-11 | 18·68 |
| GFAP | 0·02 | 1·76e-12 | 2·53e-10 | 16·89 |
| OGN | 0·02 | 8·15e-12 | 1·05e-09 | 15·38 |
| WISP2 | 0·02 | 9·25e-11 | 1·09e-08 | 12·99 |
| HAVCR1 | 0·03 | 1·84e-10 | 1·98e-08 | 12·31 |
| CCDC80 | 0·02 | 3·91e-10 | 3·89e-08 | 11·57 |
| RSPO3 | 0·01 | 5·14e-10 | 4·75e-08 | 11·30 |
| CR2 | -0·02 | 9·24e-10 | 7·96e-08 | 10·73 |
| ADM | 0·01 | 1·61e-09 | 1·3e-07 | 10·18 |
| TIMP4 | 0·01 | 2·87e-09 | 2·18e-07 | 9·61 |
| TNFSF11 | -0·02 | 5·72e-09 | 4·11e-07 | 8·94 |
| EFEMP1 | 0·01 | 9·31e-09 | 6·34e-07 | 8·46 |
| PLAT | 0·02 | 1·32e-08 | 8·21e-07 | 8·12 |
| IGFBP4 | 0·01 | 1·33e-08 | 8·21e-07 | 8·11 |
| TNFRSF11B | 0·01 | 1·41e-08 | 8·31e-07 | 8·05 |
| PGF | 0·01 | 2·35e-08 | 1·32e-06 | 7·56 |
| HSPB6 | 0·01 | 3·25e-08 | 1·75e-06 | 7·24 |
| CDCP1 | 0·02 | 4·98e-08 | 2·53e-06 | 6·82 |
| TNFRSF11A | 0·01 | 5·09e-08 | 2·53e-06 | 6·80 |
| IGFBPL1 | 0·01 | 5·49e-08 | 2·63e-06 | 6·73 |
| MMP12 | 0·02 | 8·46e-08 | 3·91e-06 | 6·31 |
| NFASC | 0·01 | 1·1e-07 | 4·93e-06 | 6·05 |
| SCARB2 | 0·01 | 1·22e-07 | 5·25e-06 | 5·95 |
| HGF | 0·01 | 1·37e-07 | 5·58e-06 | 5·84 |
| PIK3IP1 | 0·01 | 1·38e-07 | 5·58e-06 | 5·83 |
| NTproBNP | 0·04 | 1·44e-07 | 5·64e-06 | 5·79 |
| RSPO1 | 0·01 | 2·21e-07 | 8·42e-06 | 5·37 |
| ADAMTS16 | 0·01 | 2·42e-07 | 8·95e-06 | 5·28 |
| SCARA5 | 0·01 | 2·81e-07 | 1·01e-05 | 5·14 |
| CCL27 | 0·02 | 3·97e-07 | 1·39e-05 | 4·80 |
| CXCL14 | 0·02 | 4·09e-07 | 1·39e-05 | 4·77 |
| TREM2 | 0·02 | 4·41e-07 | 1·46e-05 | 4·70 |
| KIT | -0·01 | 4·63e-07 | 1·5e-05 | 4·65 |
| CHI3L1 | 0·02 | 4·81e-07 | 1·52e-05 | 4·62 |
| PTN | 0·01 | 5·11e-07 | 1·56e-05 | 4·56 |
| MMP7 | 0·01 | 5·22e-07 | 1·56e-05 | 4·54 |
| SPON2 | 0·01 | 5·3e-07 | 1·56e-05 | 4·52 |
| SPINK4 | 0·02 | 5·74e-07 | 1·65e-05 | 4·45 |
| NPPB | 0·04 | 6·04e-07 | 1·7e-05 | 4·40 |
| THY1 | 0·01 | 7·88e-07 | 2·17e-05 | 4·14 |
| DPT | 0·01 | 8·43e-07 | 2·27e-05 | 4·07 |
| EGFR | -0·00 | 9·12e-07 | 2·41e-05 | 4·00 |
| FLT3LG | 0·01 | 1·32e-06 | 3·42e-05 | 3·64 |
| MSR1 | 0·02 | 1·36e-06 | 3·44e-05 | 3·61 |
| EPO | 0·02 | 1·45e-06 | 3·6e-05 | 3·55 |
| CD1C | -0·00 | 1·48e-06 | 3·61e-05 | 3·53 |
| MOG | 0·01 | 1·61e-06 | 3·85e-05 | 3·45 |
| ADGRG2 | -0·01 | 1·66e-06 | 3·88e-05 | 3·42 |
| CLMP | 0·00 | 1·68e-06 | 3·88e-05 | 3·40 |
| TIMP1 | 0·00 | 2·06e-06 | 4·68e-05 | 3·21 |
| CCL11 | 0·01 | 2·26e-06 | 5·04e-05 | 3·12 |
| RARRES2 | 0·01 | 2·48e-06 | 5·42e-05 | 3·03 |
| ADGRG1 | 0·02 | 2·87e-06 | 6·19e-05 | 2·89 |
| JAM2 | 0·00 | 3·05e-06 | 6·46e-05 | 2·83 |
| IL6 | 0·02 | 3·47e-06 | 7·23e-05 | 2·70 |
| TNFRSF10A | 0·01 | 4·32e-06 | 8·81e-05 | 2·49 |
| FGF5 | 0·01 | 4·37e-06 | 8·81e-05 | 2·48 |
| TMSB10 | 0·01 | 4·43e-06 | 8·81e-05 | 2·47 |
| SIAE | 0·01 | 4·69e-06 | 9·19e-05 | 2·41 |
| FSTL3 | 0·01 | 4·91e-06 | 9·48e-05 | 2·37 |
| CTSZ | 0·01 | 5·67e-06 | 0·00 | 2·23 |
| SORCS2 | 0·01 | 6·31e-06 | 0·00 | 2·13 |
| TNFRSF10B | 0·01 | 6·33e-06 | 0·00 | 2·12 |
| MLN | 0·02 | 6·62e-06 | 0·00 | 2·08 |
| GDNF | 0·01 | 6·99e-06 | 0·00 | 2·03 |
| PTGDS | 0·01 | 7·39e-06 | 0·00 | 1·98 |
| PI3 | 0·01 | 8·61e-06 | 0·00 | 1·83 |
| IGFBP3 | -0·01 | 8·86e-06 | 0·00 | 1·80 |
| VSIG4 | 0·01 | 9·6e-06 | 0·00 | 1·72 |
| VEGFA | 0·01 | 9·76e-06 | 0·00 | 1·71 |
| NCS1 | 0·01 | 1·04e-05 | 0·00 | 1·65 |
| TGFB1 | 0·01 | 1·05e-05 | 0·00 | 1·63 |
| TXNDC5 | 0·01 | 1·11e-05 | 0·00 | 1·59 |
| CDON | -0·01 | 1·16e-05 | 0·00 | 1·54 |
| TGFBR2 | 0·01 | 1·23e-05 | 0·00 | 1·49 |
| CCL13 | 0·01 | 1·3e-05 | 0·00 | 1·43 |
| CD59 | 0·00 | 1·31e-05 | 0·00 | 1·42 |
| SNCG | 0·02 | 1·49e-05 | 0·00 | 1·30 |
| IGFBP6 | 0·01 | 1·6e-05 | 0·00 | 1·23 |
| HAVCR2 | 0·01 | 1·7e-05 | 0·00 | 1·17 |
| LEP | 0·03 | 1·9e-05 | 0·00 | 1·07 |
| CST3 | 0·00 | 2·1e-05 | 0·00 | 0·97 |
| TNFSF13 | 0·00 | 2·48e-05 | 0·00 | 0·81 |
| CDH15 | 0·01 | 2·72e-05 | 0·00 | 0·72 |
| ITGB5 | 0·01 | 2·74e-05 | 0·00 | 0·72 |
| CXCL8 | 0·01 | 2·89e-05 | 0·00 | 0·67 |
| CXCL17 | 0·02 | 2·9e-05 | 0·00 | 0·67 |
| INHBC | 0·01 | 3·21e-05 | 0·00 | 0·57 |
| AMBP | 0·00 | 3·23e-05 | 0·00 | 0·56 |
| KRT18 | 0·02 | 3·26e-05 | 0·00 | 0·55 |
| ARSA | 0·01 | 3·26e-05 | 0·00 | 0·55 |
| COLEC12 | 0·00 | 3·6e-05 | 0·00 | 0·46 |
| CCL3 | 0·01 | 3·64e-05 | 0·00 | 0·45 |
| FABP4 | 0·01 | 3·65e-05 | 0·00 | 0·44 |
| SSC5D | 0·01 | 3·85e-05 | 0·00 | 0·39 |
| COL6A3 | 0·01 | 4·27e-05 | 0·00 | 0·29 |
| MAD1L1 | 0·01 | 4·49e-05 | 0·00 | 0·25 |
| LGALS9 | 0·00 | 4·58e-05 | 0·00 | 0·23 |
| CSTB | 0·01 | 4·94e-05 | 0·00 | 0·16 |
| CGA | 0·01 | 5·04e-05 | 0·00 | 0·14 |
| PODXL2 | -0·00 | 5·44e-05 | 0·00 | 0·06 |
| TNFRSF1A | 0·00 | 5·53e-05 | 0·00 | 0·05 |
| TNFRSF19 | 0·00 | 5·68e-05 | 0·00 | 0·02 |
| ENPP5 | -0·01 | 6·1e-05 | 0·00 | -0·04 |
| ENPP7 | 0·02 | 6·12e-05 | 0·00 | -0·04 |
| COL18A1 | 0·00 | 6·17e-05 | 0·00 | -0·05 |
| IDUA | 0·01 | 6·41e-05 | 0·00 | -0·08 |
| PLA2G15 | 0·00 | 6·45e-05 | 0·00 | -0·09 |
| GFRA1 | 0·00 | 7·07e-05 | 0·00 | -0·18 |
| WFDC2 | 0·01 | 7·68e-05 | 0·00 | -0·26 |
| CREG1 | 0·01 | 8·36e-05 | 0·00 | -0·34 |
| DKK3 | 0·00 | 8·47e-05 | 0·00 | -0·35 |
| MDK | 0·01 | 8·48e-05 | 0·00 | -0·35 |
| CTSF | 0·01 | 8·51e-05 | 0·00 | -0·35 |
| NBL1 | 0·00 | 8·69e-05 | 0·00 | -0·37 |
| EGFL7 | 0·00 | 8·95e-05 | 0·00 | -0·40 |
| LILRB4 | 0·01 | 9·66e-05 | 0·00 | -0·47 |
| ANPEP | -0·00 | 9·71e-05 | 0·00 | -0·48 |
| TNXB | -0·00 | 0·00 | 0·00 | -0·51 |
| NPDC1 | 0·00 | 0·00 | 0·00 | -0·52 |
| IGFBP7 | 0·00 | 0·00 | 0·00 | -0·54 |
| CD302 | 0·00 | 0·00 | 0·00 | -0·55 |
| IL17D | 0·01 | 0·00 | 0·00 | -0·57 |
| CCL2 | 0·00 | 0·00 | 0·00 | -0·70 |
| FOLR2 | 0·00 | 0·00 | 0·00 | -0·84 |
| NOTCH3 | 0·00 | 0·00 | 0·00 | -0·86 |
| DKK1 | 0·01 | 0·00 | 0·00 | -0·86 |
| XG | 0·00 | 0·00 | 0·00 | -0·98 |
| AGRN | 0·00 | 0·00 | 0·00 | -1·02 |
| PHOSPHO1 | 0·00 | 0·00 | 0·00 | -1·05 |
| CPM | 0·01 | 0·00 | 0·00 | -1·06 |
| CKAP4 | 0·00 | 0·00 | 0·00 | -1·08 |
| HSPG2 | 0·00 | 0·00 | 0·00 | -1·10 |
| SFRP1 | 0·01 | 0·00 | 0·00 | -1·20 |
| VWC2 | 0·01 | 0·00 | 0·00 | -1·32 |
| GGH | 0·00 | 0·00 | 0·00 | -1·33 |
| PROK1 | -0·01 | 0·00 | 0·00 | -1·38 |
| BCAM | 0·00 | 0·00 | 0·00 | -1·43 |
| MB | 0·01 | 0·00 | 0·00 | -1·43 |
| SPON1 | 0·00 | 0·00 | 0·00 | -1·44 |
| FUT3_FUT5 | 0·01 | 0·00 | 0·00 | -1·46 |
| PRSS8 | 0·01 | 0·00 | 0·00 | -1·47 |
| CTSV | -0·01 | 0·00 | 0·00 | -1·47 |
| IGF2R | 0·00 | 0·00 | 0·00 | -1·51 |
| BAX | 0·01 | 0·00 | 0·00 | -1·55 |
| SCGB1A1 | 0·01 | 0·00 | 0·00 | -1·56 |
| SPINK1 | 0·01 | 0·00 | 0·00 | -1·57 |
| CXCL9 | 0·01 | 0·00 | 0·00 | -1·62 |
| EBI3_IL27 | 0·00 | 0·00 | 0·00 | -1·64 |
| AREG | 0·01 | 0·00 | 0·00 | -1·78 |
| S100P | 0·01 | 0·00 | 0·00 | -1·79 |
| LGALS4 | 0·01 | 0·00 | 0·00 | -1·81 |
| CHIT1 | 0·03 | 0·00 | 0·00 | -1·83 |
| CD74 | 0·00 | 0·00 | 0·00 | -1·84 |
| EFNA1 | 0·00 | 0·00 | 0·00 | -1·91 |
| CD300E | 0·01 | 0·00 | 0·00 | -1·91 |
| FAM3C | 0·00 | 0·00 | 0·00 | -1·92 |
| CDH2 | 0·01 | 0·00 | 0·00 | -1·98 |
| RET | -0·01 | 0·00 | 0·00 | -1·98 |
| APOH | 0·00 | 0·00 | 0·00 | -2·01 |
| RTN4R | 0·00 | 0·00 | 0·00 | -2·06 |
| TFF1 | 0·02 | 0·00 | 0·00 | -2·12 |
| NADK | 0·01 | 0·00 | 0·00 | -2·13 |
| CCN2 | 0·01 | 0·00 | 0·00 | -2·15 |
| WISP1 | 0·00 | 0·00 | 0·00 | -2·16 |
| TRIAP1 | 0·00 | 0·00 | 0·00 | -2·18 |
| DBI | 0·01 | 0·00 | 0·00 | -2·19 |
| SOST | 0·01 | 0·00 | 0·00 | -2·19 |
| CTSC | 0·01 | 0·00 | 0·00 | -2·25 |
| CD63 | 0·01 | 0·00 | 0·00 | -2·25 |
| MFAP5 | 0·01 | 0·00 | 0·00 | -2·27 |
| CRH | -0·02 | 0·00 | 0·00 | -2·33 |
| DTX3 | 0·00 | 0·00 | 0·00 | -2·41 |
| CTSD | 0·00 | 0·00 | 0·00 | -2·42 |
| TNFSF10 | -0·00 | 0·00 | 0·00 | -2·48 |
| FGF21 | 0·02 | 0·00 | 0·00 | -2·51 |
| TFPI2 | 0·00 | 0·00 | 0·00 | -2·51 |
| GFER | 0·01 | 0·00 | 0·00 | -2·63 |
| CA14 | -0·00 | 0·00 | 0·00 | -2·63 |
| SMPD1 | 0·01 | 0·00 | 0·00 | -2·63 |
| LAYN | 0·00 | 0·00 | 0·00 | -2·65 |
| ERBB3 | -0·00 | 0·00 | 0·00 | -2·65 |
| DCN | 0·00 | 0·00 | 0·00 | -2·66 |
| RBP5 | 0·01 | 0·00 | 0·00 | -2·75 |
| BOC | -0·00 | 0·00 | 0·00 | -2·75 |
| C1QA | 0·00 | 0·00 | 0·00 | -2·75 |
| CA9 | 0·01 | 0·00 | 0·00 | -2·76 |
| FAS | 0·00 | 0·00 | 0·00 | -2·77 |
| CCL14 | 0·00 | 0·00 | 0·00 | -2·78 |
| RNASET2 | 0·00 | 0·00 | 0·00 | -2·80 |
| FCGR3B | -0·01 | 0·00 | 0·00 | -2·82 |
| ITGA11 | -0·00 | 0·00 | 0·00 | -2·85 |
| CDH5 | -0·00 | 0·00 | 0·00 | -2·85 |
| ACE2 | 0·01 | 0·00 | 0·00 | -2·87 |
| LAIR1 | 0·00 | 0·00 | 0·00 | -2·88 |
| SERPINE1 | 0·01 | 0·00 | 0·00 | -2·91 |
| PRDX3 | 0·01 | 0·00 | 0·00 | -2·98 |
| ATOX1 | 0·01 | 0·00 | 0·00 | -2·99 |
| ADA2 | 0·01 | 0·00 | 0·00 | -3·00 |
| EFNA4 | 0·00 | 0·00 | 0·00 | -3·03 |
| SMOC1 | 0·00 | 0·00 | 0·00 | -3·06 |
| CSF1 | 0·00 | 0·00 | 0·00 | -3·07 |
| NUCB2 | 0·00 | 0·00 | 0·00 | -3·08 |
| CD300C | 0·00 | 0·00 | 0·00 | -3·10 |
| CPXM1 | 0·01 | 0·00 | 0·00 | -3·11 |
| PRSS2 | 0·01 | 0·00 | 0·00 | -3·12 |
| CCL28 | 0·01 | 0·00 | 0·01 | -3·16 |
| CPB1 | 0·01 | 0·00 | 0·01 | -3·18 |
| TNFRSF14 | 0·00 | 0·00 | 0·01 | -3·18 |
| DAG1 | 0·00 | 0·00 | 0·01 | -3·20 |
| FURIN | 0·00 | 0·00 | 0·01 | -3·21 |
| CHRDL1 | 0·01 | 0·00 | 0·01 | -3·22 |
| FASLG | -0·00 | 0·00 | 0·01 | -3·24 |
| CCN3 | 0·00 | 0·00 | 0·01 | -3·26 |
| ENG | -0·00 | 0·00 | 0·01 | -3·27 |
| KDR | -0·00 | 0·00 | 0·01 | -3·27 |
| GUSB | 0·01 | 0·00 | 0·01 | -3·28 |
| CHGB | 0·00 | 0·00 | 0·01 | -3·31 |
| TINAGL1 | 0·00 | 0·00 | 0·01 | -3·32 |
| MVK | 0·01 | 0·00 | 0·01 | -3·33 |
| SERPINA11 | 0·01 | 0·00 | 0·01 | -3·33 |
| CLC | 0·01 | 0·00 | 0·01 | -3·34 |
| CD79B | 0·01 | 0·00 | 0·01 | -3·35 |
| IL1R1 | 0·00 | 0·00 | 0·01 | -3·38 |
| FABP1 | 0·02 | 0·00 | 0·01 | -3·39 |
| MET | -0·00 | 0·00 | 0·01 | -3·39 |
| DLK1 | 0·01 | 0·00 | 0·01 | -3·42 |
| CDHR2 | 0·01 | 0·00 | 0·01 | -3·43 |
| F11R | 0·00 | 0·00 | 0·01 | -3·44 |
| STC1 | 0·01 | 0·00 | 0·01 | -3·45 |
| DPP7 | 0·01 | 0·00 | 0·01 | -3·46 |
| ITIH3 | 0·00 | 0·00 | 0·01 | -3·47 |
| HYAL1 | 0·00 | 0·00 | 0·01 | -3·47 |
| CFC1 | 0·00 | 0·00 | 0·01 | -3·50 |
| CXCL16 | 0·00 | 0·00 | 0·01 | -3·51 |
| VEGFC | 0·00 | 0·00 | 0·01 | -3·51 |
| CD40 | 0·00 | 0·00 | 0·01 | -3·53 |
| IL18RAP | -0·03 | 0·00 | 0·01 | -3·54 |
| TFF3 | 0·00 | 0·00 | 0·01 | -3·56 |
| DCBLD2 | 0·00 | 0·00 | 0·01 | -3·61 |
| FGFR2 | 0·00 | 0·00 | 0·01 | -3·63 |
| TNFRSF1B | 0·00 | 0·00 | 0·01 | -3·65 |
| CLEC4A | -0·00 | 0·00 | 0·01 | -3·67 |
| GRPEL1 | 0·01 | 0·00 | 0·01 | -3·68 |
| CCL18 | 0·01 | 0·00 | 0·01 | -3·74 |
| CRIP2 | 0·00 | 0·00 | 0·01 | -3·77 |
| DSC2 | 0·00 | 0·00 | 0·01 | -3·81 |
| TIMP3 | 0·01 | 0·00 | 0·01 | -3·88 |
| NID2 | 0·01 | 0·00 | 0·01 | -3·92 |
| DDAH1 | 0·00 | 0·00 | 0·01 | -3·92 |
| GSTA1 | 0·02 | 0·00 | 0·01 | -3·92 |
| GCG | 0·02 | 0·00 | 0·01 | -3·94 |
| FST | 0·00 | 0·00 | 0·02 | -3·97 |
| DPY30 | 0·00 | 0·00 | 0·02 | -4·01 |
| IGSF8 | 0·00 | 0·00 | 0·02 | -4·03 |
| CCL15 | 0·00 | 0·00 | 0·02 | -4·03 |
| POLR2F | 0·00 | 0·00 | 0·02 | -4·04 |
| VWF | 0·02 | 0·00 | 0·02 | -4·05 |
| ANGPTL2 | 0·01 | 0·00 | 0·02 | -4·05 |
| PLXNB2 | 0·00 | 0·00 | 0·02 | -4·08 |
| SLAMF1 | 0·00 | 0·00 | 0·02 | -4·09 |
| NOS1 | 0·01 | 0·00 | 0·02 | -4·10 |
| VWA1 | 0·00 | 0·00 | 0·02 | -4·12 |
| LGALS1 | 0·00 | 0·00 | 0·02 | -4·13 |
| ADH4 | 0·01 | 0·00 | 0·02 | -4·18 |
| IGF1R | 0·00 | 0·00 | 0·02 | -4·20 |
| NAAA | 0·01 | 0·00 | 0·02 | -4·22 |
| FGF23 | 0·01 | 0·00 | 0·02 | -4·24 |
| CTSH | 0·01 | 0·00 | 0·02 | -4·25 |
| FXN | 0·01 | 0·00 | 0·02 | -4·26 |
| APOM | -0·00 | 0·00 | 0·02 | -4·26 |
| LBR | 0·01 | 0·00 | 0·02 | -4·29 |
| KEL | 0·00 | 0·00 | 0·02 | -4·32 |
| METAP1D | 0·01 | 0·00 | 0·02 | -4·38 |
| FOLR1 | 0·00 | 0·00 | 0·02 | -4·39 |
| CLPP | 0·01 | 0·00 | 0·02 | -4·40 |
| CNST | 0·01 | 0·00 | 0·03 | -4·42 |
| HNMT | 0·01 | 0·00 | 0·03 | -4·50 |
| FCER2 | 0·01 | 0·00 | 0·03 | -4·50 |
| PCDH1 | 0·00 | 0·00 | 0·03 | -4·50 |
| COL1A1 | -0·00 | 0·00 | 0·03 | -4·51 |
| ROBO2 | 0·00 | 0·00 | 0·03 | -4·52 |
| OMG | 0·01 | 0·00 | 0·03 | -4·53 |
| CA3 | 0·01 | 0·00 | 0·03 | -4·53 |
| C1QTNF1 | 0·01 | 0·00 | 0·03 | -4·54 |
| LTBR | 0·00 | 0·00 | 0·03 | -4·54 |
| PPP1R12A | 0·01 | 0·00 | 0·03 | -4·54 |
| CD97 | 0·00 | 0·00 | 0·03 | -4·56 |
| ADGRE2 | 0·00 | 0·00 | 0·03 | -4·57 |
| BRK1 | 0·00 | 0·00 | 0·03 | -4·58 |
| ADAM22 | 0·00 | 0·00 | 0·03 | -4·58 |
| AGR2 | 0·01 | 0·00 | 0·03 | -4·59 |
| SLAMF8 | 0·01 | 0·00 | 0·03 | -4·60 |
| CPA1 | 0·01 | 0·00 | 0·03 | -4·60 |
| CTSL | 0·00 | 0·00 | 0·03 | -4·61 |
| SORD | 0·01 | 0·00 | 0·03 | -4·62 |
| CXCL10 | 0·01 | 0·00 | 0·03 | -4·65 |
| ATP5IF1 | 0·01 | 0·00 | 0·03 | -4·65 |
| DRAXIN | 0·01 | 0·00 | 0·03 | -4·67 |
| GH2 | -0·02 | 0·00 | 0·03 | -4·67 |
| F3 | 0·00 | 0·00 | 0·03 | -4·68 |
| GSTA3 | 0·01 | 0·00 | 0·03 | -4·70 |
| PPP1R9B | 0·01 | 0·00 | 0·03 | -4·73 |
| MEGF10 | -0·00 | 0·00 | 0·03 | -4·74 |
| CA5A | 0·01 | 0·01 | 0·04 | -4·80 |
| CD163 | 0·00 | 0·01 | 0·04 | -4·81 |
| IL12B | 0·01 | 0·01 | 0·04 | -4·83 |
| ACP6 | 0·01 | 0·01 | 0·04 | -4·84 |
| KLK11 | 0·00 | 0·01 | 0·04 | -4·85 |
| AIFM1 | 0·01 | 0·01 | 0·04 | -4·86 |
| TAFA5 | 0·00 | 0·01 | 0·04 | -4·86 |
| SIGLEC1 | 0·00 | 0·01 | 0·04 | -4·88 |
| SFTPD | 0·01 | 0·01 | 0·04 | -4·91 |
| PTS | 0·01 | 0·01 | 0·04 | -4·96 |
| F2R | 0·00 | 0·01 | 0·04 | -4·97 |
| SLC16A1 | 0·00 | 0·01 | 0·05 | -4·99 |
| MERTK | 0·00 | 0·01 | 0·05 | -5·00 |
| GALNT10 | 0·00 | 0·01 | 0·05 | -5·01 |
| CCL7 | 0·00 | 0·01 | 0·05 | -5·02 |
| IL10RB | 0·00 | 0·01 | 0·05 | -5·02 |
| SMOC2 | 0·00 | 0·01 | 0·05 | -5·03 |
| GLO1 | 0·00 | 0·01 | 0·05 | -5·06 |
| LRPAP1 | 0·00 | 0·01 | 0·05 | -5·06 |
| TNF | 0·00 | 0·01 | 0·05 | -5·08 |
| PEBP1 | 0·00 | 0·01 | 0·05 | -5·12 |
| PILRA | 0·00 | 0·01 | 0·05 | -5·12 |
| IL1R2 | -0·00 | 0·01 | 0·05 | -5·13 |
| PAMR1 | 0·00 | 0·01 | 0·05 | -5·13 |
| EPHA1 | 0·00 | 0·01 | 0·05 | -5·14 |
| CTSS | 0·00 | 0·01 | 0·05 | -5·14 |
| ALDH1A1 | 0·01 | 0·01 | 0·05 | -5·14 |
| KLK6 | 0·00 | 0·01 | 0·05 | -5·14 |
| TYRO3 | -0·00 | 0·01 | 0·05 | -5·16 |
| ANGPT1 | 0·01 | 0·01 | 0·05 | -5·18 |
| DCTPP1 | 0·00 | 0·01 | 0·05 | -5·19 |
| PRELP | 0·00 | 0·01 | 0·05 | -5·19 |
| APEX1 | 0·01 | 0·01 | 0·05 | -5·20 |
| IL12A_IL12B | 0·01 | 0·01 | 0·06 | -5·20 |
| ANGPTL4 | 0·00 | 0·01 | 0·06 | -5·22 |
| AGRP | -0·00 | 0·01 | 0·06 | -5·23 |
| SKAP1 | -0·00 | 0·01 | 0·06 | -5·23 |
| LEFTY2 | 0·00 | 0·01 | 0·06 | -5·24 |
| CXCL11 | 0·01 | 0·01 | 0·06 | -5·24 |
| IL19 | 0·01 | 0·01 | 0·06 | -5·25 |
| GGT1 | 0·01 | 0·01 | 0·06 | -5·25 |
| CD38 | 0·00 | 0·01 | 0·06 | -5·25 |
| IL7R | -0·00 | 0·01 | 0·06 | -5·27 |
| EPHA2 | 0·00 | 0·01 | 0·06 | -5·27 |
| LYPD3 | -0·00 | 0·01 | 0·06 | -5·27 |
| WIF1 | -0·00 | 0·01 | 0·06 | -5·28 |
| CCN1 | 0·01 | 0·01 | 0·06 | -5·29 |
| PCOLCE | 0·00 | 0·01 | 0·06 | -5·30 |
| PVR | 0·00 | 0·01 | 0·06 | -5·30 |
| ABL1 | 0·00 | 0·01 | 0·06 | -5·30 |
| MMP1 | 0·01 | 0·01 | 0·06 | -5·32 |
| ISLR2 | -0·00 | 0·01 | 0·06 | -5·34 |
| CDNF | 0·00 | 0·01 | 0·06 | -5·35 |
| GUCA2A | 0·01 | 0·01 | 0·06 | -5·37 |
| THPO | 0·00 | 0·01 | 0·06 | -5·37 |
| CDHR1 | -0·00 | 0·01 | 0·06 | -5·37 |
| GALNT7 | -0·00 | 0·01 | 0·06 | -5·38 |
| CD55 | 0·00 | 0·01 | 0·06 | -5·38 |
| DPP6 | 0·00 | 0·01 | 0·06 | -5·38 |
| CNTNAP2 | 0·00 | 0·01 | 0·06 | -5·39 |
| APBB1IP | 0·00 | 0·01 | 0·06 | -5·39 |
| SHMT1 | 0·01 | 0·01 | 0·06 | -5·40 |
| IL5RA | 0·00 | 0·02 | 0·07 | -5·41 |
| MUC13 | 0·00 | 0·02 | 0·07 | -5·44 |
| ESAM | 0·00 | 0·02 | 0·07 | -5·45 |
| CD34 | -0·00 | 0·02 | 0·07 | -5·46 |
| IL1RN | 0·00 | 0·02 | 0·07 | -5·47 |
| VSIR | 0·00 | 0·02 | 0·07 | -5·47 |
| COX5B | 0·01 | 0·02 | 0·07 | -5·49 |
| TNFRSF10C | 0·00 | 0·02 | 0·07 | -5·49 |
| CLEC1B | 0·01 | 0·02 | 0·07 | -5·50 |
| APLP1 | -0·00 | 0·02 | 0·07 | -5·50 |
| ACVRL1 | 0·00 | 0·02 | 0·07 | -5·50 |
| GP2 | 0·01 | 0·02 | 0·07 | -5·51 |
| IL12RB1 | 0·00 | 0·02 | 0·07 | -5·51 |
| COMP | 0·00 | 0·02 | 0·07 | -5·51 |
| TXNRD1 | 0·00 | 0·02 | 0·07 | -5·51 |
| RELT | 0·00 | 0·02 | 0·07 | -5·51 |
| C19orf12 | 0·01 | 0·02 | 0·07 | -5·52 |
| ISM1 | 0·00 | 0·02 | 0·07 | -5·53 |
| PRCP | 0·00 | 0·02 | 0·07 | -5·53 |
| CD27 | 0·00 | 0·02 | 0·07 | -5·54 |
| RGMB | 0·00 | 0·02 | 0·07 | -5·54 |
| CPPED1 | 0·00 | 0·02 | 0·07 | -5·55 |
| SORT1 | 0·00 | 0·02 | 0·08 | -5·58 |
| CTRB1 | 0·00 | 0·02 | 0·08 | -5·59 |
| PEAR1 | -0·00 | 0·02 | 0·08 | -5·60 |
| GPR37 | 0·01 | 0·02 | 0·08 | -5·61 |
| BAG6 | 0·00 | 0·02 | 0·08 | -5·61 |
| DECR1 | 0·01 | 0·02 | 0·08 | -5·61 |
| IL15RA | 0·00 | 0·02 | 0·08 | -5·62 |
| SCAMP3 | 0·01 | 0·02 | 0·08 | -5·66 |
| MANF | 0·01 | 0·02 | 0·08 | -5·66 |
| PLA2G7 | -0·00 | 0·02 | 0·08 | -5·67 |
| CNTN3 | 0·00 | 0·02 | 0·08 | -5·68 |
| LYN | 0·00 | 0·02 | 0·08 | -5·68 |
| VSTM2L | 0·00 | 0·02 | 0·08 | -5·68 |
| SPRY2 | 0·01 | 0·02 | 0·08 | -5·69 |
| MYOC | 0·00 | 0·02 | 0·08 | -5·69 |
| PRKAR1A | 0·01 | 0·02 | 0·08 | -5·70 |
| NOS3 | 0·00 | 0·02 | 0·08 | -5·70 |
| CIAPIN1 | 0·00 | 0·02 | 0·08 | -5·71 |
| LY96 | 0·00 | 0·02 | 0·08 | -5·71 |
| CNTN5 | 0·00 | 0·02 | 0·09 | -5·72 |
| EPS8L2 | 0·00 | 0·02 | 0·09 | -5·73 |
| LAT | 0·01 | 0·02 | 0·09 | -5·73 |
| LRIG1 | 0·00 | 0·02 | 0·09 | -5·73 |
| RRM2B | 0·00 | 0·02 | 0·09 | -5·73 |
| CALB1 | 0·00 | 0·02 | 0·09 | -5·74 |
| FLT3 | -0·00 | 0·02 | 0·09 | -5·75 |
| CRIM1 | 0·00 | 0·02 | 0·09 | -5·75 |
| DSG4 | -0·00 | 0·02 | 0·09 | -5·75 |
| PON3 | -0·00 | 0·03 | 0·09 | -5·79 |
| IL17C | 0·01 | 0·03 | 0·09 | -5·81 |
| NOMO1 | 0·00 | 0·03 | 0·09 | -5·81 |
| SERPINB6 | 0·00 | 0·03 | 0·09 | -5·83 |
| SNX9 | 0·00 | 0·03 | 0·10 | -5·84 |
| FABP9 | -0·00 | 0·03 | 0·10 | -5·84 |
| CD70 | 0·00 | 0·03 | 0·10 | -5·85 |
| PTPN6 | 0·01 | 0·03 | 0·10 | -5·86 |
| CCL16 | 0·00 | 0·03 | 0·10 | -5·87 |
| SLAMF7 | 0·00 | 0·03 | 0·10 | -5·88 |
| EGLN1 | 0·00 | 0·03 | 0·10 | -5·89 |
| APP | 0·00 | 0·03 | 0·10 | -5·89 |
| PDGFA | 0·01 | 0·03 | 0·10 | -5·90 |
| AXL | -0·00 | 0·03 | 0·10 | -5·90 |
| CD2AP | 0·00 | 0·03 | 0·10 | -5·91 |
| PPY | 0·01 | 0·03 | 0·10 | -5·92 |
| ALCAM | 0·00 | 0·03 | 0·10 | -5·93 |
| ANGPTL7 | 0·00 | 0·03 | 0·10 | -5·94 |
| WFIKKN1 | -0·00 | 0·03 | 0·11 | -5·95 |
| ROR1 | 0·00 | 0·03 | 0·11 | -5·95 |
| ENTPD5 | 0·00 | 0·03 | 0·11 | -5·96 |
| SCARF1 | 0·00 | 0·03 | 0·11 | -5·98 |
| REG1A | 0·00 | 0·03 | 0·11 | -5·98 |
| NRP2 | 0·00 | 0·03 | 0·11 | -5·99 |
| HSPB1 | 0·01 | 0·03 | 0·11 | -5·99 |
| LAIR2 | 0·01 | 0·04 | 0·11 | -6·02 |
| ANGPT2 | 0·00 | 0·04 | 0·11 | -6·03 |
| P4HB | 0·00 | 0·04 | 0·11 | -6·03 |
| CD14 | 0·00 | 0·04 | 0·11 | -6·03 |
| BTN2A1 | 0·00 | 0·04 | 0·11 | -6·04 |
| RASSF2 | 0·00 | 0·04 | 0·12 | -6·05 |
| BTN3A2 | 0·00 | 0·04 | 0·12 | -6·05 |
| CA6 | -0·01 | 0·04 | 0·12 | -6·05 |
| DPP10 | 0·00 | 0·04 | 0·12 | -6·06 |
| HEXIM1 | 0·00 | 0·04 | 0·12 | -6·07 |
| CXCL5 | 0·01 | 0·04 | 0·12 | -6·08 |
| CLEC7A | 0·00 | 0·04 | 0·12 | -6·09 |
| STK11 | 0·00 | 0·04 | 0·12 | -6·09 |
| LGALS3 | 0·00 | 0·04 | 0·12 | -6·09 |
| SPINK6 | 0·00 | 0·04 | 0·12 | -6·11 |
| LTBP3 | 0·00 | 0·04 | 0·12 | -6·12 |
| FIS1 | 0·00 | 0·04 | 0·12 | -6·12 |
| ARSB | 0·00 | 0·04 | 0·12 | -6·13 |
| NECTIN2 | 0·00 | 0·04 | 0·13 | -6·14 |
| MPIG6B | 0·01 | 0·04 | 0·13 | -6·14 |
| NUDT5 | 0·00 | 0·04 | 0·13 | -6·15 |
| REN | 0·01 | 0·04 | 0·13 | -6·15 |
| MEGF9 | -0·00 | 0·04 | 0·13 | -6·16 |
| CLEC4C | -0·00 | 0·04 | 0·13 | -6·17 |
| EGF | 0·01 | 0·04 | 0·13 | -6·17 |
| ANXA10 | 0·01 | 0·04 | 0·13 | -6·17 |
| CNTN2 | 0·00 | 0·04 | 0·13 | -6·17 |
| HAO1 | 0·01 | 0·04 | 0·13 | -6·18 |
| GOPC | 0·01 | 0·04 | 0·13 | -6·18 |
| KRT19 | 0·00 | 0·04 | 0·13 | -6·18 |
| REG3A | 0·01 | 0·05 | 0·13 | -6·19 |
| CCL23 | 0·00 | 0·05 | 0·13 | -6·20 |
| DIABLO | 0·01 | 0·05 | 0·13 | -6·20 |
| CD200R1 | -0·00 | 0·05 | 0·13 | -6·21 |
| TFF2 | 0·00 | 0·05 | 0·13 | -6·21 |
| CCL17 | 0·01 | 0·05 | 0·13 | -6·22 |
| AKR1C4 | 0·00 | 0·05 | 0·13 | -6·22 |
| CXCL6 | 0·00 | 0·05 | 0·14 | -6·25 |
| VTA1 | 0·00 | 0·05 | 0·14 | -6·25 |
| LRP11 | 0·00 | 0·05 | 0·14 | -6·26 |
| PSG1 | 0·01 | 0·05 | 0·14 | -6·26 |
| PLIN1 | 0·00 | 0·05 | 0·14 | -6·27 |
| TFRC | 0·00 | 0·05 | 0·14 | -6·27 |
| IL7 | 0·00 | 0·05 | 0·14 | -6·28 |
| NT5E | 0·00 | 0·05 | 0·14 | -6·28 |
| SMAD5 | 0·00 | 0·05 | 0·14 | -6·28 |
| ING1 | -0·00 | 0·05 | 0·14 | -6·29 |
| CXCL3 | 0·01 | 0·05 | 0·14 | -6·29 |
| AHCY | 0·00 | 0·05 | 0·14 | -6·30 |
| ASAH2 | 0·00 | 0·05 | 0·14 | -6·31 |
| PLAUR | 0·00 | 0·05 | 0·14 | -6·31 |
| CBLIF | 0·00 | 0·05 | 0·15 | -6·32 |
| PDCD5 | 0·00 | 0·05 | 0·15 | -6·32 |
| GRAP2 | 0·01 | 0·05 | 0·15 | -6·32 |
| HCLS1 | 0·00 | 0·05 | 0·15 | -6·33 |
| RTBDN | 0·00 | 0·06 | 0·15 | -6·35 |
| SUSD1 | 0·00 | 0·06 | 0·15 | -6·35 |
| LGALS7_LGALS7B | 0·00 | 0·06 | 0·15 | -6·37 |
| DNAJA2 | 0·01 | 0·06 | 0·15 | -6·38 |
| SELP | 0·00 | 0·06 | 0·15 | -6·38 |
| DCTN1 | 0·00 | 0·06 | 0·16 | -6·39 |
| UMOD | -0·00 | 0·06 | 0·16 | -6·40 |
| CLEC4G | -0·00 | 0·06 | 0·16 | -6·40 |
| LACTB2 | 0·00 | 0·06 | 0·16 | -6·41 |
| IRAK1 | 0·00 | 0·06 | 0·16 | -6·41 |
| CD99 | 0·00 | 0·06 | 0·16 | -6·42 |
| HPCAL1 | 0·00 | 0·06 | 0·16 | -6·42 |
| FABP5 | 0·00 | 0·06 | 0·16 | -6·43 |
| ENO1 | 0·00 | 0·06 | 0·16 | -6·43 |
| FGFBP1 | -0·00 | 0·06 | 0·16 | -6·44 |
| IL18BP | 0·00 | 0·06 | 0·16 | -6·44 |
| CEACAM8 | 0·00 | 0·06 | 0·16 | -6·44 |
| BAIAP2 | 0·00 | 0·06 | 0·16 | -6·45 |
| MZB1 | 0·00 | 0·07 | 0·17 | -6·48 |
| DAB2 | 0·00 | 0·07 | 0·17 | -6·48 |
| DCXR | 0·00 | 0·07 | 0·17 | -6·50 |
| MGMT | 0·01 | 0·07 | 0·17 | -6·50 |
| GPA33 | -0·01 | 0·07 | 0·17 | -6·50 |
| EIF4G1 | 0·01 | 0·07 | 0·17 | -6·50 |
| FYB1 | 0·01 | 0·07 | 0·17 | -6·51 |
| BST1 | -0·00 | 0·07 | 0·18 | -6·51 |
| MEP1B | 0·01 | 0·07 | 0·18 | -6·52 |
| CD46 | 0·00 | 0·07 | 0·18 | -6·53 |
| DPP4 | -0·00 | 0·07 | 0·18 | -6·54 |
| TNFRSF4 | 0·00 | 0·07 | 0·18 | -6·55 |
| CRTAC1 | 0·00 | 0·07 | 0·18 | -6·56 |
| ENO2 | 0·00 | 0·07 | 0·18 | -6·56 |
| SLC39A5 | 0·00 | 0·07 | 0·18 | -6·56 |
| IL6R | -0·00 | 0·07 | 0·18 | -6·57 |
| AFP | 0·00 | 0·07 | 0·18 | -6·57 |
| THOP1 | -0·00 | 0·07 | 0·18 | -6·57 |
| DNER | -0·00 | 0·08 | 0·19 | -6·58 |
| SIGLEC10 | 0·00 | 0·08 | 0·19 | -6·59 |
| GFRA3 | 0·00 | 0·08 | 0·19 | -6·59 |
| LY6D | 0·00 | 0·08 | 0·19 | -6·60 |
| CTSO | 0·00 | 0·08 | 0·19 | -6·60 |
| KIR3DL1 | -0·01 | 0·08 | 0·19 | -6·60 |
| PPP1R2 | 0·00 | 0·08 | 0·19 | -6·61 |
| RNF41 | 0·00 | 0·08 | 0·19 | -6·62 |
| THBS4 | 0·00 | 0·08 | 0·19 | -6·62 |
| DOK2 | 0·01 | 0·08 | 0·20 | -6·63 |
| GP6 | 0·00 | 0·08 | 0·20 | -6·64 |
| CD69 | 0·00 | 0·08 | 0·20 | -6·65 |
| MSRA | 0·00 | 0·08 | 0·20 | -6·65 |
| ACY1 | 0·00 | 0·08 | 0·20 | -6·65 |
| NXPH1 | 0·00 | 0·08 | 0·20 | -6·66 |
| EPCAM | -0·01 | 0·08 | 0·20 | -6·67 |
| PDGFB | 0·01 | 0·08 | 0·20 | -6·67 |
| FGR | 0·00 | 0·09 | 0·20 | -6·68 |
| QDPR | 0·00 | 0·09 | 0·20 | -6·68 |
| TPSAB1 | 0·00 | 0·09 | 0·21 | -6·69 |
| KITLG | 0·00 | 0·09 | 0·21 | -6·70 |
| CD276 | 0·00 | 0·09 | 0·21 | -6·71 |
| NTF3 | -0·00 | 0·09 | 0·22 | -6·73 |
| REG4 | 0·00 | 0·09 | 0·22 | -6·73 |
| IGSF3 | 0·00 | 0·09 | 0·22 | -6·73 |
| MCFD2 | 0·00 | 0·09 | 0·22 | -6·74 |
| MCAM | 0·00 | 0·09 | 0·22 | -6·74 |
| CASC4 | 0·00 | 0·09 | 0·22 | -6·74 |
| AMIGO2 | -0·00 | 0·09 | 0·22 | -6·74 |
| MUC16 | 0·00 | 0·09 | 0·22 | -6·76 |
| NID1 | 0·00 | 0·09 | 0·22 | -6·76 |
| CPVL | 0·00 | 0·10 | 0·23 | -6·78 |
| CCL25 | 0·00 | 0·10 | 0·23 | -6·78 |
| KYNU | 0·00 | 0·10 | 0·23 | -6·78 |
| REG1B | 0·00 | 0·10 | 0·23 | -6·78 |
| VCAN | 0·00 | 0·10 | 0·23 | -6·79 |
| ANXA11 | 0·00 | 0·10 | 0·23 | -6·79 |
| PIGR | 0·00 | 0·10 | 0·23 | -6·79 |
| CXCL13 | 0·00 | 0·10 | 0·23 | -6·79 |
| DKKL1 | 0·00 | 0·10 | 0·23 | -6·79 |
| SNAP23 | 0·00 | 0·10 | 0·23 | -6·80 |
| SEMA3F | 0·00 | 0·10 | 0·23 | -6·80 |
| USP8 | 0·00 | 0·10 | 0·23 | -6·81 |
| PAG1 | 0·00 | 0·10 | 0·23 | -6·81 |
| CLEC11A | 0·00 | 0·10 | 0·23 | -6·81 |
| MEPE | -0·00 | 0·10 | 0·23 | -6·81 |
| COL9A1 | -0·00 | 0·10 | 0·23 | -6·81 |
| CGREF1 | 0·00 | 0·10 | 0·23 | -6·82 |
| PCSK9 | 0·00 | 0·10 | 0·23 | -6·82 |
| ENTPD2 | -0·00 | 0·10 | 0·23 | -6·82 |
| GPC5 | -0·00 | 0·10 | 0·23 | -6·83 |
| PARK7 | 0·00 | 0·10 | 0·23 | -6·83 |
| NUDC | 0·00 | 0·10 | 0·23 | -6·83 |
| IQGAP2 | 0·00 | 0·11 | 0·24 | -6·86 |
| PILRB | 0·00 | 0·11 | 0·24 | -6·86 |
| TCN2 | 0·00 | 0·11 | 0·24 | -6·86 |
| TNFRSF21 | 0·00 | 0·11 | 0·24 | -6·86 |
| PODXL | -0·00 | 0·11 | 0·24 | -6·87 |
| LILRA5 | 0·00 | 0·11 | 0·24 | -6·87 |
| SEMA4D | -0·00 | 0·11 | 0·24 | -6·88 |
| CLEC5A | 0·00 | 0·11 | 0·24 | -6·88 |
| TBCB | 0·00 | 0·11 | 0·24 | -6·88 |
| KLK1 | 0·01 | 0·11 | 0·25 | -6·89 |
| CCL4 | 0·00 | 0·11 | 0·25 | -6·89 |
| CDH17 | -0·00 | 0·11 | 0·25 | -6·89 |
| BCAN | -0·00 | 0·11 | 0·25 | -6·89 |
| BGN | 0·00 | 0·11 | 0·25 | -6·90 |
| HBEGF | 0·00 | 0·11 | 0·25 | -6·90 |
| ZBTB16 | 0·00 | 0·12 | 0·25 | -6·91 |
| PPIB | 0·00 | 0·12 | 0·25 | -6·91 |
| CCL8 | 0·00 | 0·12 | 0·25 | -6·91 |
| BSG | 0·00 | 0·12 | 0·25 | -6·92 |
| RWDD1 | 0·00 | 0·12 | 0·25 | -6·92 |
| ASGR1 | 0·00 | 0·12 | 0·25 | -6·92 |
| HARS | 0·00 | 0·12 | 0·25 | -6·92 |
| ANXA3 | 0·00 | 0·12 | 0·25 | -6·93 |
| JCHAIN | 0·00 | 0·12 | 0·26 | -6·94 |
| CNTN1 | 0·00 | 0·12 | 0·26 | -6·94 |
| NDRG1 | 0·00 | 0·12 | 0·26 | -6·95 |
| MAVS | 0·00 | 0·12 | 0·26 | -6·95 |
| EZR | 0·00 | 0·12 | 0·26 | -6·96 |
| ANGPTL1 | 0·00 | 0·12 | 0·26 | -6·97 |
| CA13 | 0·00 | 0·12 | 0·26 | -6·97 |
| CD274 | 0·00 | 0·13 | 0·27 | -6·97 |
| SPARC | 0·00 | 0·13 | 0·27 | -6·98 |
| ULBP2 | 0·00 | 0·13 | 0·27 | -6·98 |
| MESD | 0·00 | 0·13 | 0·27 | -6·98 |
| TPMT | -0·00 | 0·13 | 0·27 | -6·99 |
| LPO | -0·00 | 0·13 | 0·27 | -6·99 |
| ST6GAL1 | 0·00 | 0·13 | 0·27 | -6·99 |
| TXLNA | 0·00 | 0·13 | 0·27 | -6·99 |
| ENTPD6 | 0·00 | 0·13 | 0·27 | -6·99 |
| HSD11B1 | -0·00 | 0·13 | 0·27 | -6·99 |
| NPTX1 | 0·00 | 0·13 | 0·27 | -6·99 |
| VMO1 | 0·00 | 0·13 | 0·27 | -7·00 |
| GCNT1 | 0·00 | 0·13 | 0·27 | -7·00 |
| SCGB3A2 | -0·00 | 0·13 | 0·27 | -7·00 |
| ADAM8 | -0·00 | 0·13 | 0·27 | -7·01 |
| CA12 | 0·00 | 0·13 | 0·28 | -7·02 |
| MED18 | 0·00 | 0·13 | 0·28 | -7·02 |
| FUT8 | 0·00 | 0·13 | 0·28 | -7·02 |
| FBP1 | 0·01 | 0·13 | 0·28 | -7·02 |
| MGLL | 0·00 | 0·13 | 0·28 | -7·02 |
| ELOA | 0·00 | 0·14 | 0·28 | -7·03 |
| MILR1 | 0·00 | 0·14 | 0·28 | -7·03 |
| ROBO1 | -0·00 | 0·14 | 0·28 | -7·03 |
| LAP3 | 0·00 | 0·14 | 0·28 | -7·04 |
| SCGN | 0·00 | 0·14 | 0·28 | -7·04 |
| F9 | 0·00 | 0·14 | 0·28 | -7·04 |
| PFKFB2 | 0·00 | 0·14 | 0·28 | -7·05 |
| CA4 | -0·00 | 0·14 | 0·28 | -7·05 |
| DDR1 | 0·00 | 0·14 | 0·28 | -7·05 |
| TGFA | 0·00 | 0·14 | 0·28 | -7·06 |
| PLAU | 0·00 | 0·14 | 0·29 | -7·06 |
| PRSS27 | -0·00 | 0·14 | 0·29 | -7·07 |
| PTPRS | -0·00 | 0·14 | 0·29 | -7·07 |
| CST5 | 0·00 | 0·14 | 0·29 | -7·07 |
| BID | 0·00 | 0·14 | 0·29 | -7·07 |
| CNDP1 | -0·00 | 0·14 | 0·29 | -7·08 |
| VSTM1 | 0·00 | 0·14 | 0·29 | -7·08 |
| HSPA1A | 0·00 | 0·14 | 0·29 | -7·08 |
| RBKS | 0·00 | 0·15 | 0·29 | -7·09 |
| GLB1 | 0·00 | 0·15 | 0·29 | -7·09 |
| BCR | 0·00 | 0·15 | 0·29 | -7·09 |
| SFTPA2 | -0·00 | 0·15 | 0·30 | -7·10 |
| GYS1 | 0·01 | 0·15 | 0·30 | -7·11 |
| LAG3 | 0·00 | 0·15 | 0·30 | -7·11 |
| B4GALT1 | 0·00 | 0·15 | 0·30 | -7·11 |
| PVALB | 0·00 | 0·15 | 0·30 | -7·12 |
| TGM2 | 0·00 | 0·15 | 0·30 | -7·12 |
| TJAP1 | 0·00 | 0·15 | 0·30 | -7·12 |
| CLIP2 | 0·00 | 0·15 | 0·30 | -7·12 |
| PLXNA4 | 0·00 | 0·15 | 0·30 | -7·13 |
| AGXT | 0·00 | 0·16 | 0·30 | -7·13 |
| SCG2 | 0·00 | 0·16 | 0·30 | -7·13 |
| PRTN3 | -0·00 | 0·16 | 0·30 | -7·14 |
| EIF4EBP1 | 0·00 | 0·16 | 0·31 | -7·14 |
| SNAP29 | 0·00 | 0·16 | 0·31 | -7·14 |
| SPINT2 | 0·00 | 0·16 | 0·31 | -7·15 |
| PMVK | 0·01 | 0·16 | 0·31 | -7·15 |
| SLITRK2 | 0·00 | 0·16 | 0·31 | -7·15 |
| LILRB2 | 0·00 | 0·16 | 0·31 | -7·15 |
| CKMT1A_CKMT1B | 0·00 | 0·16 | 0·31 | -7·16 |
| CD207 | -0·00 | 0·16 | 0·31 | -7·16 |
| COMT | 0·00 | 0·16 | 0·31 | -7·16 |
| PSME2 | 0·00 | 0·16 | 0·31 | -7·17 |
| CST6 | -0·00 | 0·16 | 0·31 | -7·17 |
| LGMN | 0·00 | 0·16 | 0·31 | -7·17 |
| RILP | 0·00 | 0·16 | 0·31 | -7·17 |
| ITGA5 | 0·00 | 0·17 | 0·31 | -7·18 |
| IL2RA | -0·00 | 0·17 | 0·31 | -7·18 |
| DNMBP | 0·00 | 0·17 | 0·31 | -7·18 |
| DLL1 | 0·00 | 0·17 | 0·32 | -7·18 |
| CD164 | 0·00 | 0·17 | 0·32 | -7·19 |
| KIR2DL3 | -0·00 | 0·17 | 0·32 | -7·19 |
| LRRN1 | -0·00 | 0·17 | 0·32 | -7·19 |
| CES3 | -0·01 | 0·17 | 0·32 | -7·20 |
| ICA1 | 0·00 | 0·17 | 0·32 | -7·20 |
| PLA2G4A | 0·00 | 0·17 | 0·32 | -7·20 |
| TARBP2 | 0·00 | 0·17 | 0·32 | -7·21 |
| IL4R | 0·00 | 0·17 | 0·33 | -7·21 |
| ADAM23 | 0·00 | 0·17 | 0·33 | -7·22 |
| CBLN4 | 0·00 | 0·18 | 0·33 | -7·23 |
| CEACAM1 | -0·00 | 0·18 | 0·33 | -7·23 |
| CD109 | 0·00 | 0·18 | 0·33 | -7·24 |
| CDH6 | 0·00 | 0·18 | 0·34 | -7·24 |
| SULT2A1 | 0·00 | 0·18 | 0·34 | -7·24 |
| CD40LG | 0·00 | 0·18 | 0·34 | -7·24 |
| BACH1 | 0·00 | 0·18 | 0·34 | -7·25 |
| METAP2 | 0·00 | 0·18 | 0·34 | -7·25 |
| PRDX5 | 0·00 | 0·18 | 0·34 | -7·25 |
| CDSN | 0·00 | 0·18 | 0·34 | -7·25 |
| IFNLR1 | -0·00 | 0·19 | 0·34 | -7·26 |
| CRKL | 0·00 | 0·19 | 0·34 | -7·26 |
| DAPP1 | 0·01 | 0·19 | 0·34 | -7·27 |
| OSCAR | 0·00 | 0·19 | 0·34 | -7·27 |
| FLT4 | 0·00 | 0·19 | 0·34 | -7·27 |
| STX4 | 0·00 | 0·19 | 0·34 | -7·27 |
| CD58 | -0·00 | 0·19 | 0·35 | -7·28 |
| NMNAT1 | 0·00 | 0·20 | 0·36 | -7·30 |
| CAPG | 0·00 | 0·20 | 0·36 | -7·30 |
| CD48 | -0·00 | 0·20 | 0·36 | -7·31 |
| CASP2 | 0·00 | 0·20 | 0·36 | -7·31 |
| HDGF | 0·00 | 0·20 | 0·36 | -7·31 |
| TNFSF13B | 0·00 | 0·20 | 0·36 | -7·31 |
| ARHGEF12 | 0·00 | 0·20 | 0·36 | -7·31 |
| CCL20 | 0·00 | 0·20 | 0·36 | -7·32 |
| HBQ1 | -0·00 | 0·20 | 0·36 | -7·32 |
| F7 | 0·00 | 0·20 | 0·36 | -7·32 |
| HMOX2 | 0·00 | 0·20 | 0·36 | -7·32 |
| IPCEF1 | 0·00 | 0·20 | 0·37 | -7·33 |
| PADI2 | 0·00 | 0·21 | 0·37 | -7·34 |
| IGFBP1 | -0·01 | 0·21 | 0·37 | -7·35 |
| LXN | -0·00 | 0·21 | 0·38 | -7·36 |
| PARP1 | 0·00 | 0·21 | 0·38 | -7·36 |
| PLA2G2A | 0·00 | 0·21 | 0·38 | -7·36 |
| OXT | 0·01 | 0·21 | 0·38 | -7·36 |
| SIGLEC7 | 0·00 | 0·22 | 0·38 | -7·37 |
| CRLF1 | -0·00 | 0·22 | 0·38 | -7·37 |
| MITD1 | 0·00 | 0·22 | 0·39 | -7·38 |
| STAT5B | 0·00 | 0·22 | 0·39 | -7·39 |
| CALCOCO1 | 0·00 | 0·22 | 0·39 | -7·39 |
| TRIM21 | 0·00 | 0·22 | 0·39 | -7·39 |
| CDH1 | 0·00 | 0·22 | 0·39 | -7·39 |
| HTRA2 | 0·00 | 0·23 | 0·39 | -7·40 |
| PON2 | 0·00 | 0·23 | 0·39 | -7·40 |
| DKK4 | 0·00 | 0·23 | 0·39 | -7·40 |
| TLR3 | 0·00 | 0·23 | 0·39 | -7·40 |
| DDC | -0·00 | 0·23 | 0·39 | -7·40 |
| RARRES1 | 0·00 | 0·23 | 0·39 | -7·40 |
| EBAG9 | 0·00 | 0·23 | 0·40 | -7·41 |
| SKAP2 | 0·00 | 0·23 | 0·40 | -7·41 |
| QPCT | 0·00 | 0·23 | 0·40 | -7·41 |
| TIA1 | 0·00 | 0·23 | 0·40 | -7·41 |
| LHB | -0·00 | 0·23 | 0·40 | -7·41 |
| XCL1 | 0·00 | 0·23 | 0·40 | -7·41 |
| PECAM1 | 0·00 | 0·23 | 0·40 | -7·41 |
| RABGAP1L | -0·00 | 0·23 | 0·40 | -7·42 |
| PPCDC | 0·00 | 0·23 | 0·40 | -7·42 |
| PRDX6 | -0·00 | 0·23 | 0·40 | -7·42 |
| PLA2G10 | 0·00 | 0·23 | 0·40 | -7·43 |
| TNR | -0·00 | 0·23 | 0·40 | -7·43 |
| NCAM2 | -0·00 | 0·24 | 0·41 | -7·44 |
| BCL2L11 | 0·00 | 0·24 | 0·41 | -7·44 |
| TNFRSF8 | -0·00 | 0·24 | 0·41 | -7·44 |
| MIF | 0·00 | 0·24 | 0·41 | -7·44 |
| PDLIM7 | 0·00 | 0·24 | 0·41 | -7·44 |
| TACSTD2 | 0·00 | 0·24 | 0·41 | -7·45 |
| CLEC14A | 0·00 | 0·24 | 0·41 | -7·45 |
| CALCA | 0·00 | 0·24 | 0·41 | -7·45 |
| CASP8 | 0·00 | 0·25 | 0·42 | -7·46 |
| AOC3 | 0·00 | 0·25 | 0·42 | -7·46 |
| SUGT1 | 0·00 | 0·25 | 0·42 | -7·46 |
| ACAA1 | 0·00 | 0·25 | 0·42 | -7·47 |
| GAL | -0·00 | 0·25 | 0·42 | -7·47 |
| KLB | 0·00 | 0·25 | 0·42 | -7·47 |
| CELA3A | 0·00 | 0·25 | 0·42 | -7·47 |
| DBNL | 0·00 | 0·25 | 0·42 | -7·47 |
| TCL1A | 0·00 | 0·25 | 0·43 | -7·48 |
| ZBTB17 | 0·00 | 0·26 | 0·43 | -7·49 |
| CCL5 | 0·00 | 0·26 | 0·43 | -7·49 |
| LY75 | -0·00 | 0·26 | 0·43 | -7·49 |
| PDGFRB | 0·00 | 0·26 | 0·43 | -7·49 |
| TNFSF14 | 0·00 | 0·26 | 0·43 | -7·49 |
| IL18 | 0·00 | 0·26 | 0·43 | -7·50 |
| CDKN2D | 0·00 | 0·26 | 0·43 | -7·50 |
| RHOC | 0·00 | 0·26 | 0·43 | -7·50 |
| AGR3 | 0·00 | 0·26 | 0·43 | -7·50 |
| NCK2 | 0·00 | 0·26 | 0·43 | -7·50 |
| SEZ6L2 | 0·00 | 0·26 | 0·43 | -7·50 |
| MIA | 0·00 | 0·26 | 0·43 | -7·50 |
| C2 | 0·00 | 0·26 | 0·43 | -7·50 |
| CHL1 | -0·00 | 0·26 | 0·43 | -7·51 |
| SDC1 | -0·00 | 0·26 | 0·43 | -7·51 |
| SERPINB1 | 0·00 | 0·26 | 0·43 | -7·51 |
| SUSD2 | 0·00 | 0·26 | 0·43 | -7·51 |
| IL1RL2 | 0·00 | 0·26 | 0·43 | -7·51 |
| NINJ1 | 0·00 | 0·26 | 0·43 | -7·51 |
| CNTN4 | 0·00 | 0·27 | 0·44 | -7·52 |
| IL6ST | 0·00 | 0·27 | 0·44 | -7·52 |
| CA11 | 0·00 | 0·27 | 0·44 | -7·52 |
| ERBB2 | 0·00 | 0·27 | 0·44 | -7·52 |
| NCR1 | 0·00 | 0·27 | 0·44 | -7·52 |
| TBL1X | 0·00 | 0·27 | 0·44 | -7·53 |
| MDGA1 | -0·00 | 0·27 | 0·44 | -7·53 |
| LBP | 0·00 | 0·27 | 0·44 | -7·53 |
| AMN | 0·00 | 0·27 | 0·44 | -7·53 |
| LHPP | 0·00 | 0·27 | 0·44 | -7·53 |
| CDC37 | 0·00 | 0·27 | 0·44 | -7·53 |
| ART3 | 0·00 | 0·28 | 0·44 | -7·54 |
| ICAM3 | -0·00 | 0·28 | 0·45 | -7·55 |
| ALPP | -0·01 | 0·28 | 0·45 | -7·55 |
| NME3 | -0·00 | 0·28 | 0·45 | -7·55 |
| ACAN | 0·00 | 0·28 | 0·45 | -7·55 |
| CPE | 0·00 | 0·28 | 0·45 | -7·55 |
| AIF1 | 0·00 | 0·28 | 0·45 | -7·55 |
| FOPNL | 0·00 | 0·28 | 0·45 | -7·55 |
| IKBKG | 0·00 | 0·28 | 0·45 | -7·55 |
| PAM | 0·00 | 0·28 | 0·45 | -7·56 |
| ERP44 | -0·00 | 0·28 | 0·45 | -7·56 |
| ITGB7 | -0·00 | 0·28 | 0·45 | -7·56 |
| NPPC | -0·00 | 0·29 | 0·45 | -7·56 |
| RNASE3 | 0·00 | 0·29 | 0·45 | -7·56 |
| ANGPTL3 | 0·00 | 0·29 | 0·46 | -7·57 |
| FAM3B | 0·00 | 0·29 | 0·46 | -7·57 |
| NTRK3 | 0·00 | 0·29 | 0·46 | -7·57 |
| CNPY4 | 0·00 | 0·29 | 0·46 | -7·57 |
| PLXDC1 | 0·00 | 0·29 | 0·46 | -7·58 |
| SIRT2 | 0·00 | 0·30 | 0·46 | -7·58 |
| PDGFRA | 0·00 | 0·30 | 0·46 | -7·59 |
| CHAC2 | 0·00 | 0·30 | 0·46 | -7·59 |
| MAP4K5 | 0·00 | 0·30 | 0·47 | -7·59 |
| CDHR5 | 0·00 | 0·30 | 0·47 | -7·59 |
| SULT1A1 | 0·00 | 0·30 | 0·47 | -7·59 |
| CRTAM | -0·00 | 0·30 | 0·47 | -7·59 |
| ADAMTS13 | -0·00 | 0·30 | 0·47 | -7·60 |
| NCAN | -0·00 | 0·30 | 0·47 | -7·60 |
| ITGB1BP2 | 0·00 | 0·30 | 0·47 | -7·60 |
| TNFRSF13C | -0·00 | 0·30 | 0·47 | -7·60 |
| LY9 | -0·00 | 0·30 | 0·47 | -7·60 |
| PRTFDC1 | 0·00 | 0·30 | 0·47 | -7·60 |
| AMY2A | 0·00 | 0·31 | 0·47 | -7·60 |
| AZU1 | -0·00 | 0·31 | 0·47 | -7·60 |
| AKT1S1 | 0·00 | 0·31 | 0·48 | -7·61 |
| KRT5 | 0·00 | 0·31 | 0·48 | -7·62 |
| FCN2 | -0·00 | 0·31 | 0·48 | -7·62 |
| SH2D1A | -0·00 | 0·31 | 0·48 | -7·62 |
| CASP3 | 0·00 | 0·32 | 0·48 | -7·63 |
| LSP1 | 0·00 | 0·32 | 0·49 | -7·63 |
| USO1 | 0·00 | 0·32 | 0·49 | -7·64 |
| IL15 | 0·00 | 0·32 | 0·49 | -7·64 |
| CD5 | -0·00 | 0·32 | 0·49 | -7·64 |
| TNC | -0·00 | 0·33 | 0·50 | -7·65 |
| CASP1 | 0·00 | 0·33 | 0·50 | -7·65 |
| SIRPB1 | 0·00 | 0·33 | 0·50 | -7·65 |
| SRC | 0·00 | 0·33 | 0·50 | -7·65 |
| AARSD1 | 0·00 | 0·33 | 0·50 | -7·66 |
| TPP1 | 0·00 | 0·33 | 0·50 | -7·66 |
| CD6 | -0·00 | 0·33 | 0·50 | -7·66 |
| GHRL | -0·00 | 0·33 | 0·50 | -7·66 |
| OBP2B | -0·00 | 0·33 | 0·51 | -7·66 |
| KLK8 | -0·00 | 0·34 | 0·51 | -7·67 |
| STX8 | 0·00 | 0·34 | 0·51 | -7·67 |
| IRAK4 | 0·00 | 0·34 | 0·51 | -7·67 |
| MASP1 | -0·00 | 0·34 | 0·51 | -7·67 |
| EIF5A | 0·00 | 0·34 | 0·51 | -7·67 |
| CD28 | 0·00 | 0·34 | 0·51 | -7·68 |
| C2CD2L | 0·00 | 0·34 | 0·51 | -7·68 |
| S100A11 | 0·00 | 0·34 | 0·51 | -7·68 |
| CRADD | 0·00 | 0·34 | 0·51 | -7·68 |
| LILRB1 | 0·00 | 0·34 | 0·52 | -7·68 |
| CDC27 | 0·00 | 0·35 | 0·52 | -7·68 |
| CLSTN2 | 0·00 | 0·35 | 0·52 | -7·69 |
| KLRB1 | 0·00 | 0·35 | 0·52 | -7·69 |
| BIN2 | 0·00 | 0·35 | 0·52 | -7·69 |
| GP1BA | -0·00 | 0·35 | 0·52 | -7·69 |
| NRP1 | 0·00 | 0·35 | 0·52 | -7·70 |
| PPP3R1 | 0·00 | 0·35 | 0·52 | -7·70 |
| AGER | -0·00 | 0·36 | 0·53 | -7·70 |
| LRRC25 | 0·00 | 0·36 | 0·53 | -7·70 |
| CD84 | -0·00 | 0·36 | 0·53 | -7·71 |
| LRP1 | 0·00 | 0·36 | 0·53 | -7·71 |
| CD4 | 0·00 | 0·36 | 0·53 | -7·71 |
| KLK13 | -0·00 | 0·36 | 0·53 | -7·71 |
| CD177 | -0·00 | 0·36 | 0·54 | -7·72 |
| NFKBIE | 0·00 | 0·37 | 0·54 | -7·72 |
| BAG3 | -0·00 | 0·37 | 0·54 | -7·72 |
| CCL19 | 0·00 | 0·37 | 0·54 | -7·72 |
| CES2 | 0·00 | 0·37 | 0·54 | -7·73 |
| TNFRSF6B | 0·00 | 0·37 | 0·54 | -7·73 |
| SCARB1 | 0·00 | 0·37 | 0·55 | -7·73 |
| KLK10 | 0·00 | 0·38 | 0·55 | -7·74 |
| DFFA | 0·00 | 0·38 | 0·55 | -7·74 |
| LAT2 | 0·00 | 0·38 | 0·55 | -7·74 |
| TNFRSF12A | 0·00 | 0·38 | 0·55 | -7·74 |
| FGF19 | -0·00 | 0·38 | 0·55 | -7·74 |
| AMY2B | 0·00 | 0·38 | 0·55 | -7·74 |
| FEN1 | -0·00 | 0·38 | 0·55 | -7·74 |
| VASN | -0·00 | 0·38 | 0·55 | -7·74 |
| YES1 | 0·00 | 0·38 | 0·55 | -7·75 |
| IDS | -0·00 | 0·38 | 0·55 | -7·75 |
| MAP2K6 | 0·00 | 0·39 | 0·56 | -7·75 |
| CD93 | -0·00 | 0·39 | 0·56 | -7·76 |
| ANG | 0·00 | 0·39 | 0·56 | -7·76 |
| LTA | -0·00 | 0·39 | 0·56 | -7·76 |
| MFGE8 | 0·00 | 0·39 | 0·56 | -7·76 |
| CST7 | 0·00 | 0·39 | 0·56 | -7·76 |
| GZMA | -0·00 | 0·39 | 0·56 | -7·77 |
| CA2 | 0·00 | 0·40 | 0·57 | -7·77 |
| GLOD4 | 0·00 | 0·40 | 0·57 | -7·77 |
| NECTIN4 | 0·00 | 0·40 | 0·57 | -7·77 |
| GNE | 0·00 | 0·40 | 0·57 | -7·77 |
| NTRK2 | -0·00 | 0·40 | 0·57 | -7·77 |
| TMPRSS15 | 0·00 | 0·40 | 0·57 | -7·78 |
| IL13RA1 | 0·00 | 0·41 | 0·58 | -7·78 |
| TBCC | 0·00 | 0·41 | 0·58 | -7·79 |
| CD300LF | 0·00 | 0·41 | 0·58 | -7·79 |
| WFIKKN2 | -0·00 | 0·41 | 0·58 | -7·79 |
| NOTCH1 | -0·00 | 0·41 | 0·58 | -7·79 |
| PRKAB1 | 0·00 | 0·42 | 0·59 | -7·80 |
| CLUL1 | -0·00 | 0·42 | 0·59 | -7·80 |
| AMBN | 0·00 | 0·42 | 0·59 | -7·80 |
| PDGFC | -0·00 | 0·42 | 0·59 | -7·80 |
| ADA | -0·00 | 0·42 | 0·60 | -7·81 |
| KIRREL2 | -0·00 | 0·42 | 0·60 | -7·81 |
| AHSP | 0·00 | 0·42 | 0·60 | -7·81 |
| FCGR2A | 0·00 | 0·43 | 0·60 | -7·81 |
| IL34 | 0·00 | 0·43 | 0·60 | -7·81 |
| TNFSF12 | -0·00 | 0·43 | 0·60 | -7·81 |
| ECE1 | 0·00 | 0·43 | 0·60 | -7·82 |
| LILRA2 | 0·00 | 0·43 | 0·60 | -7·82 |
| IGFBP2 | 0·00 | 0·43 | 0·60 | -7·82 |
| BST2 | 0·00 | 0·43 | 0·60 | -7·82 |
| PLTP | -0·00 | 0·43 | 0·60 | -7·82 |
| BMP4 | -0·00 | 0·43 | 0·60 | -7·82 |
| LCN2 | 0·00 | 0·44 | 0·60 | -7·82 |
| ANKRD54 | -0·00 | 0·44 | 0·61 | -7·83 |
| CLEC6A | 0·00 | 0·44 | 0·61 | -7·83 |
| GRN | 0·00 | 0·44 | 0·61 | -7·83 |
| WFDC12 | 0·00 | 0·44 | 0·61 | -7·84 |
| TDGF1 | -0·00 | 0·44 | 0·61 | -7·84 |
| TBC1D5 | 0·00 | 0·45 | 0·62 | -7·84 |
| EPHB4 | 0·00 | 0·45 | 0·62 | -7·84 |
| TACC3 | 0·00 | 0·45 | 0·62 | -7·84 |
| TYMP | 0·00 | 0·45 | 0·62 | -7·84 |
| SLIT2 | 0·00 | 0·45 | 0·62 | -7·84 |
| PSPN | 0·00 | 0·45 | 0·62 | -7·85 |
| MSMB | 0·00 | 0·45 | 0·62 | -7·85 |
| IL20 | -0·00 | 0·46 | 0·62 | -7·85 |
| LTA4H | -0·00 | 0·46 | 0·62 | -7·85 |
| CES1 | 0·00 | 0·46 | 0·63 | -7·85 |
| KIFBP | 0·00 | 0·46 | 0·63 | -7·85 |
| CRACR2A | 0·00 | 0·46 | 0·63 | -7·86 |
| SIGLEC15 | -0·00 | 0·46 | 0·63 | -7·86 |
| SF3B4 | 0·00 | 0·47 | 0·64 | -7·86 |
| PAEP | 0·00 | 0·47 | 0·64 | -7·86 |
| MMP3 | 0·00 | 0·47 | 0·64 | -7·87 |
| ERBIN | 0·00 | 0·47 | 0·64 | -7·87 |
| VPS53 | 0·00 | 0·47 | 0·64 | -7·87 |
| SCG3 | -0·00 | 0·47 | 0·64 | -7·87 |
| MANSC1 | -0·00 | 0·47 | 0·64 | -7·87 |
| OGFR | 0·00 | 0·47 | 0·64 | -7·87 |
| RAD23B | 0·00 | 0·48 | 0·64 | -7·87 |
| FCRL1 | -0·00 | 0·48 | 0·64 | -7·87 |
| KYAT1 | 0·00 | 0·48 | 0·65 | -7·88 |
| FABP6 | 0·00 | 0·48 | 0·65 | -7·88 |
| SMPDL3A | 0·00 | 0·48 | 0·65 | -7·88 |
| IFNGR1 | 0·00 | 0·48 | 0·65 | -7·88 |
| MSTN | 0·00 | 0·48 | 0·65 | -7·88 |
| CXADR | -0·00 | 0·49 | 0·65 | -7·89 |
| CD83 | 0·00 | 0·49 | 0·65 | -7·89 |
| STAMBP | 0·00 | 0·49 | 0·65 | -7·89 |
| ANXA4 | 0·00 | 0·49 | 0·66 | -7·89 |
| FCRLB | 0·00 | 0·49 | 0·66 | -7·89 |
| ACP5 | 0·00 | 0·49 | 0·66 | -7·89 |
| ITGB1 | -0·00 | 0·49 | 0·66 | -7·89 |
| MICB_MICA | 0·00 | 0·49 | 0·66 | -7·89 |
| S100A12 | 0·00 | 0·50 | 0·66 | -7·89 |
| DNPH1 | 0·00 | 0·50 | 0·66 | -7·90 |
| TREML2 | 0·00 | 0·50 | 0·66 | -7·90 |
| MAPK9 | 0·00 | 0·50 | 0·66 | -7·90 |
| FCRL2 | 0·00 | 0·50 | 0·66 | -7·90 |
| THBS2 | 0·00 | 0·50 | 0·66 | -7·90 |
| FCAR | -0·00 | 0·50 | 0·66 | -7·90 |
| FMNL1 | 0·00 | 0·50 | 0·66 | -7·90 |
| EDAR | -0·00 | 0·50 | 0·66 | -7·90 |
| PTX3 | 0·00 | 0·50 | 0·66 | -7·90 |
| PDCD6 | -0·00 | 0·50 | 0·66 | -7·90 |
| INPPL1 | 0·00 | 0·50 | 0·66 | -7·90 |
| UXS1 | 0·00 | 0·51 | 0·66 | -7·90 |
| TSLP | -0·00 | 0·51 | 0·66 | -7·90 |
| PRTG | 0·00 | 0·51 | 0·66 | -7·91 |
| GSAP | 0·00 | 0·51 | 0·67 | -7·91 |
| IL17A | -0·00 | 0·51 | 0·67 | -7·91 |
| CDH3 | 0·00 | 0·51 | 0·67 | -7·91 |
| NCF2 | 0·00 | 0·51 | 0·67 | -7·91 |
| ICAM5 | 0·00 | 0·51 | 0·67 | -7·91 |
| ATG4A | -0·00 | 0·51 | 0·67 | -7·91 |
| PLIN3 | 0·00 | 0·52 | 0·67 | -7·91 |
| CORO1A | 0·00 | 0·52 | 0·67 | -7·92 |
| TIE1 | 0·00 | 0·52 | 0·67 | -7·92 |
| SELPLG | -0·00 | 0·52 | 0·67 | -7·92 |
| FKBP4 | 0·00 | 0·52 | 0·67 | -7·92 |
| BANK1 | 0·00 | 0·52 | 0·67 | -7·92 |
| TFPI | 0·00 | 0·52 | 0·67 | -7·92 |
| CARHSP1 | -0·00 | 0·52 | 0·67 | -7·92 |
| MATN3 | 0·00 | 0·52 | 0·67 | -7·92 |
| LIFR | -0·00 | 0·52 | 0·67 | -7·92 |
| MME | -0·00 | 0·53 | 0·68 | -7·93 |
| OPTC | 0·00 | 0·53 | 0·68 | -7·93 |
| PLXNB3 | 0·00 | 0·53 | 0·68 | -7·93 |
| TXNDC15 | 0·00 | 0·53 | 0·68 | -7·93 |
| PBLD | 0·00 | 0·53 | 0·68 | -7·93 |
| ICOSLG | -0·00 | 0·53 | 0·68 | -7·93 |
| CD99L2 | 0·00 | 0·54 | 0·68 | -7·93 |
| TEK | -0·00 | 0·54 | 0·69 | -7·94 |
| GZMH | -0·00 | 0·54 | 0·69 | -7·94 |
| IL1B | -0·00 | 0·54 | 0·69 | -7·94 |
| IL10RA | 0·00 | 0·55 | 0·69 | -7·94 |
| CCS | -0·00 | 0·55 | 0·69 | -7·94 |
| SIT1 | -0·00 | 0·55 | 0·69 | -7·94 |
| NELL1 | 0·00 | 0·55 | 0·70 | -7·95 |
| PTPN1 | 0·00 | 0·55 | 0·70 | -7·95 |
| NSFL1C | 0·00 | 0·55 | 0·70 | -7·95 |
| CX3CL1 | 0·00 | 0·56 | 0·70 | -7·95 |
| SEMA7A | 0·00 | 0·56 | 0·70 | -7·95 |
| CRNN | 0·00 | 0·56 | 0·70 | -7·95 |
| SELE | -0·00 | 0·56 | 0·71 | -7·96 |
| IVD | -0·00 | 0·56 | 0·71 | -7·96 |
| NUB1 | 0·00 | 0·56 | 0·71 | -7·96 |
| FETUB | 0·00 | 0·56 | 0·71 | -7·96 |
| ERBB4 | 0·00 | 0·56 | 0·71 | -7·96 |
| PPME1 | -0·00 | 0·57 | 0·71 | -7·96 |
| PKLR | 0·00 | 0·57 | 0·71 | -7·96 |
| PRL | 0·00 | 0·57 | 0·71 | -7·96 |
| IL1RL1 | -0·00 | 0·57 | 0·72 | -7·97 |
| ADAMTS8 | 0·00 | 0·57 | 0·72 | -7·97 |
| ARHGAP1 | 0·00 | 0·58 | 0·72 | -7·97 |
| PCDH17 | -0·00 | 0·58 | 0·72 | -7·97 |
| PROC | -0·00 | 0·58 | 0·72 | -7·97 |
| RGMA | 0·00 | 0·58 | 0·72 | -7·97 |
| RETN | 0·00 | 0·58 | 0·72 | -7·98 |
| LILRB5 | 0·00 | 0·58 | 0·72 | -7·98 |
| SOD2 | 0·00 | 0·58 | 0·73 | -7·98 |
| GNLY | 0·00 | 0·59 | 0·73 | -7·98 |
| EDIL3 | 0·00 | 0·59 | 0·73 | -7·98 |
| JUN | 0·00 | 0·59 | 0·73 | -7·98 |
| CD200 | 0·00 | 0·59 | 0·73 | -7·98 |
| CSF3 | 0·00 | 0·59 | 0·73 | -7·98 |
| SPP1 | 0·00 | 0·59 | 0·73 | -7·98 |
| MSLN | 0·00 | 0·59 | 0·73 | -7·98 |
| PTPRM | -0·00 | 0·59 | 0·73 | -7·98 |
| GFRA2 | -0·00 | 0·59 | 0·73 | -7·98 |
| PIK3AP1 | 0·00 | 0·59 | 0·73 | -7·98 |
| SDC4 | -0·00 | 0·59 | 0·73 | -7·98 |
| MMP10 | -0·00 | 0·59 | 0·73 | -7·98 |
| CTRC | -0·00 | 0·59 | 0·73 | -7·98 |
| CHMP1A | 0·00 | 0·60 | 0·73 | -7·98 |
| AXIN1 | 0·00 | 0·60 | 0·73 | -7·99 |
| LEPR | -0·00 | 0·60 | 0·73 | -7·99 |
| DDX58 | 0·00 | 0·60 | 0·73 | -7·99 |
| NRCAM | 0·00 | 0·60 | 0·73 | -7·99 |
| DPEP2 | -0·00 | 0·60 | 0·74 | -7·99 |
| PNLIPRP2 | -0·01 | 0·60 | 0·74 | -7·99 |
| VCAM1 | 0·00 | 0·61 | 0·74 | -7·99 |
| NPY | 0·00 | 0·61 | 0·74 | -8·00 |
| CDKN1A | 0·00 | 0·61 | 0·74 | -8·00 |
| PM20D1 | -0·00 | 0·62 | 0·75 | -8·00 |
| FLRT2 | 0·00 | 0·62 | 0·75 | -8·00 |
| SEMA4C | -0·00 | 0·62 | 0·75 | -8·00 |
| PGLYRP1 | 0·00 | 0·62 | 0·75 | -8·00 |
| SRP14 | 0·00 | 0·62 | 0·75 | -8·01 |
| CRELD2 | -0·00 | 0·63 | 0·75 | -8·01 |
| ST3GAL1 | -0·00 | 0·63 | 0·75 | -8·01 |
| PTPRN2 | 0·00 | 0·63 | 0·75 | -8·01 |
| HMBS | 0·00 | 0·63 | 0·76 | -8·01 |
| IMPA1 | -0·00 | 0·63 | 0·76 | -8·01 |
| DSG2 | 0·00 | 0·63 | 0·76 | -8·01 |
| EIF4B | 0·00 | 0·63 | 0·76 | -8·01 |
| ENAH | -0·00 | 0·64 | 0·77 | -8·01 |
| CEACAM21 | 0·00 | 0·64 | 0·77 | -8·02 |
| COL4A1 | -0·00 | 0·64 | 0·77 | -8·02 |
| ALDH3A1 | -0·00 | 0·64 | 0·77 | -8·02 |
| LRMP | 0·00 | 0·64 | 0·77 | -8·02 |
| PSIP1 | -0·00 | 0·65 | 0·77 | -8·02 |
| IL32 | 0·00 | 0·65 | 0·77 | -8·02 |
| SERPINB9 | 0·00 | 0·65 | 0·77 | -8·02 |
| CXCL12 | -0·00 | 0·65 | 0·77 | -8·02 |
| CRHR1 | -0·00 | 0·65 | 0·77 | -8·02 |
| KAZALD1 | 0·00 | 0·65 | 0·78 | -8·02 |
| CD209 | -0·00 | 0·65 | 0·78 | -8·02 |
| NDUFS6 | 0·00 | 0·65 | 0·78 | -8·02 |
| IL18R1 | 0·00 | 0·66 | 0·78 | -8·03 |
| ADAM15 | -0·00 | 0·66 | 0·78 | -8·03 |
| EPHB6 | -0·00 | 0·66 | 0·78 | -8·03 |
| CRHBP | -0·00 | 0·66 | 0·78 | -8·03 |
| ODAM | -0·00 | 0·66 | 0·78 | -8·03 |
| SSC4D | 0·00 | 0·66 | 0·78 | -8·03 |
| CD160 | -0·00 | 0·67 | 0·78 | -8·03 |
| LPL | 0·00 | 0·67 | 0·79 | -8·03 |
| CA1 | 0·00 | 0·67 | 0·79 | -8·03 |
| SEZ6L | 0·00 | 0·67 | 0·79 | -8·03 |
| FCRL5 | 0·00 | 0·67 | 0·79 | -8·04 |
| HAGH | -0·00 | 0·68 | 0·79 | -8·04 |
| KLK14 | 0·00 | 0·68 | 0·79 | -8·04 |
| CEACAM5 | 0·00 | 0·68 | 0·79 | -8·04 |
| LGALS8 | 0·00 | 0·68 | 0·80 | -8·04 |
| SERPINA12 | -0·00 | 0·68 | 0·80 | -8·04 |
| B4GAT1 | 0·00 | 0·68 | 0·80 | -8·04 |
| GZMB | 0·00 | 0·68 | 0·80 | -8·04 |
| SLITRK6 | 0·00 | 0·69 | 0·80 | -8·04 |
| RP2 | -0·00 | 0·69 | 0·81 | -8·05 |
| VNN2 | 0·00 | 0·70 | 0·81 | -8·05 |
| CANT1 | -0·00 | 0·70 | 0·81 | -8·05 |
| L1CAM | 0·00 | 0·70 | 0·81 | -8·05 |
| ICAM4 | -0·00 | 0·70 | 0·81 | -8·05 |
| STIP1 | 0·00 | 0·70 | 0·81 | -8·05 |
| CLSTN1 | 0·00 | 0·71 | 0·82 | -8·05 |
| CCL24 | -0·00 | 0·71 | 0·82 | -8·05 |
| IFNGR2 | -0·00 | 0·71 | 0·82 | -8·06 |
| IL17RB | 0·00 | 0·71 | 0·82 | -8·06 |
| GGT5 | -0·00 | 0·71 | 0·82 | -8·06 |
| HLA-E | -0·00 | 0·71 | 0·82 | -8·06 |
| ITGA6 | -0·00 | 0·72 | 0·82 | -8·06 |
| SOD1 | -0·00 | 0·72 | 0·82 | -8·06 |
| SPINT1 | -0·00 | 0·72 | 0·82 | -8·06 |
| GH1 | 0·00 | 0·72 | 0·82 | -8·06 |
| TGFBR3 | 0·00 | 0·72 | 0·82 | -8·06 |
| CLEC10A | -0·00 | 0·72 | 0·82 | -8·06 |
| ITGB6 | 0·00 | 0·72 | 0·82 | -8·06 |
| ITM2A | 0·00 | 0·72 | 0·83 | -8·06 |
| FOLR3 | 0·00 | 0·72 | 0·83 | -8·06 |
| ARG1 | -0·00 | 0·72 | 0·83 | -8·06 |
| SAMD9L | 0·00 | 0·72 | 0·83 | -8·06 |
| TGFBI | -0·00 | 0·73 | 0·83 | -8·06 |
| CD22 | -0·00 | 0·73 | 0·83 | -8·06 |
| WARS | 0·00 | 0·73 | 0·83 | -8·07 |
| GKN1 | 0·00 | 0·73 | 0·83 | -8·07 |
| SETMAR | 0·00 | 0·74 | 0·84 | -8·07 |
| CXCL1 | 0·00 | 0·74 | 0·84 | -8·07 |
| HMOX1 | -0·00 | 0·74 | 0·84 | -8·07 |
| CD244 | 0·00 | 0·74 | 0·84 | -8·07 |
| DEFA1_DEFA1B | 0·00 | 0·74 | 0·84 | -8·07 |
| LIF | 0·00 | 0·74 | 0·84 | -8·07 |
| CC2D1A | 0·00 | 0·74 | 0·84 | -8·07 |
| PSME1 | 0·00 | 0·75 | 0·84 | -8·07 |
| GPNMB | -0·00 | 0·75 | 0·84 | -8·07 |
| PDCD1LG2 | 0·00 | 0·75 | 0·84 | -8·07 |
| LDLR | 0·00 | 0·75 | 0·85 | -8·07 |
| LAMA4 | -0·00 | 0·75 | 0·85 | -8·07 |
| ESM1 | 0·00 | 0·75 | 0·85 | -8·07 |
| SERPINB8 | 0·00 | 0·75 | 0·85 | -8·08 |
| DCTN2 | 0·00 | 0·76 | 0·85 | -8·08 |
| GMPR | 0·00 | 0·76 | 0·85 | -8·08 |
| CD300LG | 0·00 | 0·76 | 0·85 | -8·08 |
| FLI1 | 0·00 | 0·76 | 0·85 | -8·08 |
| MMP8 | 0·00 | 0·76 | 0·85 | -8·08 |
| DPEP1 | -0·00 | 0·76 | 0·85 | -8·08 |
| IL10 | 0·00 | 0·77 | 0·86 | -8·08 |
| MARCO | 0·00 | 0·77 | 0·86 | -8·08 |
| LAMP3 | 0·00 | 0·77 | 0·86 | -8·08 |
| OMD | -0·00 | 0·77 | 0·86 | -8·08 |
| SLC39A14 | -0·00 | 0·78 | 0·86 | -8·08 |
| RBP2 | 0·00 | 0·78 | 0·86 | -8·08 |
| FRZB | -0·00 | 0·78 | 0·87 | -8·08 |
| BMP6 | 0·00 | 0·78 | 0·87 | -8·08 |
| NPTXR | -0·00 | 0·78 | 0·87 | -8·09 |
| THBD | -0·00 | 0·78 | 0·87 | -8·09 |
| OSM | 0·00 | 0·78 | 0·87 | -8·09 |
| DNAJB1 | 0·00 | 0·79 | 0·87 | -8·09 |
| PTH1R | -0·00 | 0·79 | 0·87 | -8·09 |
| PXN | 0·00 | 0·79 | 0·87 | -8·09 |
| IFNG | 0·00 | 0·79 | 0·87 | -8·09 |
| FLT1 | 0·00 | 0·79 | 0·87 | -8·09 |
| GLRX | -0·00 | 0·79 | 0·87 | -8·09 |
| FABP2 | -0·00 | 0·79 | 0·87 | -8·09 |
| GPC1 | 0·00 | 0·80 | 0·88 | -8·09 |
| OSMR | 0·00 | 0·80 | 0·88 | -8·09 |
| FADD | 0·00 | 0·80 | 0·88 | -8·09 |
| FKBP5 | 0·00 | 0·81 | 0·88 | -8·09 |
| SERPINA9 | -0·00 | 0·81 | 0·88 | -8·09 |
| NBN | 0·00 | 0·81 | 0·88 | -8·09 |
| C4BPB | 0·00 | 0·81 | 0·88 | -8·09 |
| VPS37A | -0·00 | 0·81 | 0·89 | -8·10 |
| PLA2G1B | 0·00 | 0·81 | 0·89 | -8·10 |
| ICAM2 | 0·00 | 0·82 | 0·89 | -8·10 |
| KLRD1 | 0·00 | 0·82 | 0·89 | -8·10 |
| UBAC1 | -0·00 | 0·82 | 0·89 | -8·10 |
| IL20RA | 0·00 | 0·82 | 0·89 | -8·10 |
| EREG | 0·00 | 0·82 | 0·89 | -8·10 |
| TPPP3 | -0·00 | 0·83 | 0·90 | -8·10 |
| CHRDL2 | -0·00 | 0·83 | 0·90 | -8·10 |
| S100A16 | -0·00 | 0·83 | 0·90 | -8·10 |
| TSHB | 0·00 | 0·83 | 0·90 | -8·10 |
| CTSB | 0·00 | 0·83 | 0·90 | -8·10 |
| XPNPEP2 | 0·00 | 0·83 | 0·90 | -8·10 |
| NCAM1 | 0·00 | 0·84 | 0·90 | -8·10 |
| IL22RA1 | -0·00 | 0·84 | 0·90 | -8·10 |
| GALNT3 | -0·00 | 0·84 | 0·90 | -8·10 |
| ADCYAP1R1 | -0·00 | 0·84 | 0·90 | -8·10 |
| FCGR2B | 0·00 | 0·84 | 0·90 | -8·10 |
| FUCA1 | -0·00 | 0·84 | 0·90 | -8·10 |
| CLEC4D | 0·00 | 0·84 | 0·90 | -8·10 |
| VAT1 | -0·00 | 0·84 | 0·90 | -8·10 |
| SIRPA | -0·00 | 0·84 | 0·91 | -8·10 |
| PSMG3 | -0·00 | 0·84 | 0·91 | -8·10 |
| SPOCK1 | 0·00 | 0·85 | 0·91 | -8·10 |
| SH2B3 | -0·00 | 0·85 | 0·91 | -8·10 |
| CCL26 | -0·00 | 0·85 | 0·91 | -8·11 |
| MNDA | -0·00 | 0·85 | 0·91 | -8·11 |
| FOXO1 | 0·00 | 0·85 | 0·91 | -8·11 |
| DEFB4A_DEFB4B | 0·00 | 0·85 | 0·91 | -8·11 |
| RAB6A | -0·00 | 0·85 | 0·91 | -8·11 |
| ITGB2 | 0·00 | 0·85 | 0·91 | -8·11 |
| HPGDS | -0·00 | 0·85 | 0·91 | -8·11 |
| FAP | -0·00 | 0·85 | 0·91 | -8·11 |
| APRT | 0·00 | 0·85 | 0·91 | -8·11 |
| SUMF2 | -0·00 | 0·86 | 0·91 | -8·11 |
| DARS | -0·00 | 0·86 | 0·91 | -8·11 |
| SIGLEC5 | -0·00 | 0·86 | 0·91 | -8·11 |
| FHIT | 0·00 | 0·86 | 0·91 | -8·11 |
| TIMD4 | 0·00 | 0·87 | 0·92 | -8·11 |
| CLPS | 0·00 | 0·87 | 0·92 | -8·11 |
| CCL21 | 0·00 | 0·87 | 0·92 | -8·11 |
| LAMP2 | 0·00 | 0·87 | 0·92 | -8·11 |
| MPI | 0·00 | 0·88 | 0·93 | -8·11 |
| GDF2 | 0·00 | 0·88 | 0·93 | -8·11 |
| NFATC1 | 0·00 | 0·88 | 0·93 | -8·11 |
| ITGAM | -0·00 | 0·88 | 0·93 | -8·11 |
| HS6ST1 | -0·00 | 0·88 | 0·93 | -8·11 |
| ITGAV | 0·00 | 0·89 | 0·93 | -8·11 |
| FCRL3 | -0·00 | 0·89 | 0·93 | -8·11 |
| CD33 | -0·00 | 0·89 | 0·93 | -8·11 |
| NT5C3A | 0·00 | 0·89 | 0·93 | -8·11 |
| SIGLEC6 | 0·00 | 0·89 | 0·93 | -8·11 |
| ICAM1 | 0·00 | 0·89 | 0·93 | -8·11 |
| AKR1B1 | 0·00 | 0·89 | 0·93 | -8·11 |
| PDCD1 | -0·00 | 0·89 | 0·93 | -8·11 |
| STC2 | 0·00 | 0·89 | 0·93 | -8·11 |
| IL16 | 0·00 | 0·89 | 0·93 | -8·11 |
| CCL22 | 0·00 | 0·90 | 0·93 | -8·11 |
| ABHD14B | -0·00 | 0·90 | 0·93 | -8·12 |
| SCP2 | -0·00 | 0·90 | 0·94 | -8·12 |
| GAS6 | -0·00 | 0·90 | 0·94 | -8·12 |
| PLPBP | 0·00 | 0·90 | 0·94 | -8·12 |
| BPIFB1 | -0·00 | 0·90 | 0·94 | -8·12 |
| SCRN1 | -0·00 | 0·90 | 0·94 | -8·12 |
| PTPRF | 0·00 | 0·90 | 0·94 | -8·12 |
| HS3ST3B1 | -0·00 | 0·91 | 0·94 | -8·12 |
| MMP9 | 0·00 | 0·91 | 0·95 | -8·12 |
| RUVBL1 | -0·00 | 0·92 | 0·95 | -8·12 |
| AK1 | -0·00 | 0·93 | 0·96 | -8·12 |
| OLR1 | 0·00 | 0·93 | 0·96 | -8·12 |
| VEGFD | 0·00 | 0·93 | 0·96 | -8·12 |
| BLMH | 0·00 | 0·94 | 0·96 | -8·12 |
| NPTN | 0·00 | 0·94 | 0·97 | -8·12 |
| IL17RA | -0·00 | 0·94 | 0·97 | -8·12 |
| CPA2 | -0·00 | 0·94 | 0·97 | -8·12 |
| NELL2 | 0·00 | 0·94 | 0·97 | -8·12 |
| TNFRSF13B | -0·00 | 0·94 | 0·97 | -8·12 |
| SCLY | -0·00 | 0·94 | 0·97 | -8·12 |
| SPINK5 | -0·00 | 0·94 | 0·97 | -8·12 |
| GSTP1 | -0·00 | 0·94 | 0·97 | -8·12 |
| SIGLEC9 | -0·00 | 0·94 | 0·97 | -8·12 |
| S100A4 | 0·00 | 0·95 | 0·97 | -8·12 |
| PSMD9 | 0·00 | 0·95 | 0·97 | -8·12 |
| CSF2RA | 0·00 | 0·95 | 0·97 | -8·12 |
| DSG3 | -0·00 | 0·96 | 0·98 | -8·12 |
| TNFRSF9 | 0·00 | 0·96 | 0·98 | -8·12 |
| MATN2 | 9·83e-05 | 0·96 | 0·98 | -8·12 |
| GALNT2 | -7·84e-05 | 0·96 | 0·98 | -8·12 |
| SLAMF6 | -8·51e-05 | 0·97 | 0·98 | -8·12 |
| WWP2 | -9·95e-05 | 0·97 | 0·98 | -8·12 |
| NPM1 | -0·00 | 0·97 | 0·98 | -8·12 |
| MPO | -9·02e-05 | 0·97 | 0·98 | -8·12 |
| HGS | -0·00 | 0·97 | 0·98 | -8·12 |
| TRAF2 | -0·00 | 0·97 | 0·98 | -8·12 |
| SPARCL1 | -7·09e-05 | 0·97 | 0·98 | -8·12 |
| CRISP2 | 0·00 | 0·97 | 0·98 | -8·12 |
| IL1RAP | -8·59e-05 | 0·97 | 0·98 | -8·12 |
| TMPRSS5 | -8·7e-05 | 0·97 | 0·98 | -8·12 |
| ADGRB3 | -6·15e-05 | 0·97 | 0·98 | -8·12 |
| PRDX1 | 7·85e-05 | 0·98 | 0·98 | -8·12 |
| BTC | 9·29e-05 | 0·98 | 0·98 | -8·12 |
| HYOU1 | -3·61e-05 | 0·98 | 0·99 | -8·12 |
| BLVRB | 6·88e-05 | 0·98 | 0·99 | -8·12 |
| CD8A | -6·57e-05 | 0·98 | 0·99 | -8·12 |
| FCRL6 | 6·21e-05 | 0·98 | 0·99 | -8·12 |
| ADAMTS15 | 6·63e-05 | 0·99 | 0·99 | -8·12 |
| PAPPA | 3·38e-05 | 0·99 | 0·99 | -8·12 |
| ENPP2 | -1·54e-05 | 0·99 | 0·99 | -8·12 |
| INPP1 | -1·76e-05 | 0·99 | 0·99 | -8·12 |
| RRM2 | 8·29e-06 | 0·99 | 0·99 | -8·12 |
| CLEC1A | 3·91e-06 | 0·99 | 0·99 | -8·12 |

## Supplementary Table 5. Association results of 1306 plasma proteins with chronological age in the 2000HIV cohort

| Protein | log FC | p-value | FDR | Beta |
| --- | --- | --- | --- | --- |
| LTBP2 | 0·02 | 1·76e-53 | 2·30e-50 | 110·17 |
| EDA2R | 0·03 | 5·48e-51 | 3·58e-48 | 104·44 |
| WNT9A | 0·01 | 4·29e-44 | 1·86e-41 | 88·61 |
| NEFL | 0·03 | 6·74e-44 | 2·20e-41 | 88·16 |
| KLK4 | 0·03 | 4·78e-37 | 1·25e-34 | 72·44 |
| GDF15 | 0·03 | 1·38e-36 | 3·00e-34 | 71·38 |
| SCARF2 | 0·01 | 9·91e-36 | 1·84e-33 | 69·42 |
| IL17D | 0·01 | 9·91e-34 | 1·61e-31 | 64·84 |
| GFAP | 0·02 | 5·49e-32 | 7·98e-30 | 60·84 |
| CXCL14 | 0·02 | 1·42e-31 | 1·85e-29 | 59·89 |
| HAVCR1 | 0·03 | 1·04e-30 | 1·24e-28 | 57·91 |
| IGFBPL1 | 0·01 | 5·06e-29 | 5·51e-27 | 54·05 |
| RSPO3 | 0·01 | 1·16e-27 | 1·17e-25 | 50·93 |
| CCDC80 | 0·02 | 3·23e-27 | 3·01e-25 | 49·92 |
| CDCP1 | 0·02 | 4·29e-25 | 3·73e-23 | 45·06 |
| RSPO1 | 0·01 | 9·32e-25 | 7·60e-23 | 44·29 |
| ADGRG1 | 0·03 | 4·48e-24 | 3·44e-22 | 42·73 |
| CXCL17 | 0·02 | 6·33e-24 | 4·49e-22 | 42·39 |
| ADAMTS16 | 0·01 | 6·54e-24 | 4·49e-22 | 42·36 |
| HSPB6 | 0·02 | 1·48e-22 | 9·69e-21 | 39·26 |
| TSPAN1 | 0·02 | 3·37e-22 | 2·09e-20 | 38·45 |
| TNFRSF11B | 0·01 | 4·14e-22 | 2·46e-20 | 38·24 |
| SFRP1 | 0·01 | 6·08e-22 | 3·45e-20 | 37·86 |
| MSR1 | 0·01 | 1·23e-21 | 6·70e-20 | 37·16 |
| CCL27 | 0·02 | 1·88e-21 | 9·82e-20 | 36·74 |
| LEFTY2 | 0·02 | 5·79e-21 | 2·90e-19 | 35·63 |
| WISP2 | 0·02 | 2·88e-20 | 1·39e-18 | 34·03 |
| TREM2 | 0·02 | 3·21e-20 | 1·49e-18 | 33·93 |
| CR2 | -0·02 | 6·64e-20 | 2·99e-18 | 33·21 |
| CGA | 0·02 | 1·76e-19 | 7·68e-18 | 32·24 |
| NCS1 | 0·01 | 1·91e-19 | 8·08e-18 | 32·16 |
| SCARB2 | 0·01 | 2·45e-19 | 1·00e-17 | 31·91 |
| FLT3LG | 0·01 | 5·48e-19 | 2·17e-17 | 31·12 |
| ADM | 0·01 | 8·84e-19 | 3·39e-17 | 30·65 |
| DTX3 | 0·00 | 2·74e-18 | 1·02e-16 | 29·53 |
| PRELP | 0·01 | 2·96e-18 | 1·07e-16 | 29·45 |
| SNCG | 0·02 | 4·06e-18 | 1·43e-16 | 29·14 |
| MLN | 0·03 | 4·79e-18 | 1·64e-16 | 28·97 |
| OGN | 0·01 | 5·49e-18 | 1·84e-16 | 28·84 |
| TIMP4 | 0·01 | 6·13e-18 | 2·00e-16 | 28·73 |
| NTproBNP | 0·03 | 4·13e-17 | 1·31e-15 | 26·84 |
| SMOC1 | 0·01 | 1·26e-16 | 3·93e-15 | 25·74 |
| PGF | 0·00 | 5·71e-16 | 1·73e-14 | 24·25 |
| DCN | 0·00 | 1·23e-15 | 3·67e-14 | 23·48 |
| IGFBP4 | 0·01 | 1·27e-15 | 3·71e-14 | 23·45 |
| GDNF | 0·01 | 5·05e-15 | 1·43e-13 | 22·09 |
| WFDC2 | 0·01 | 9·93e-15 | 2·75e-13 | 21·43 |
| CHRDL1 | 0·01 | 1·01e-14 | 2·76e-13 | 21·41 |
| MMP12 | 0·01 | 1·08e-14 | 2·90e-13 | 21·34 |
| DKK3 | 0·01 | 1·55e-14 | 4·05e-13 | 20·99 |
| SOST | 0·01 | 3·70e-14 | 9·49e-13 | 20·13 |
| CDON | -0·01 | 5·22e-14 | 1·31e-12 | 19·79 |
| NFASC | 0·00 | 7·64e-14 | 1·88e-12 | 19·42 |
| FGF5 | 0·01 | 9·99e-14 | 2·41e-12 | 19·15 |
| AMBP | 0·00 | 1·70e-13 | 4·03e-12 | 18·63 |
| PPY | 0·02 | 2·32e-13 | 5·42e-12 | 18·32 |
| DPT | 0·01 | 3·37e-13 | 7·74e-12 | 17·95 |
| TNFSF11 | -0·02 | 6·58e-13 | 1·48e-11 | 17·30 |
| CCN3 | 0·01 | 7·57e-13 | 1·67e-11 | 17·16 |
| NOTCH3 | 0·01 | 1·10e-12 | 2·41e-11 | 16·78 |
| CTSV | -0·01 | 1·27e-12 | 2·70e-11 | 16·65 |
| SCARA5 | 0·00 | 1·28e-12 | 2·70e-11 | 16·64 |
| FSTL3 | 0·01 | 2·08e-12 | 4·32e-11 | 16·16 |
| CRTAC1 | 0·00 | 2·32e-12 | 4·74e-11 | 16·06 |
| CHI3L1 | 0·02 | 6·35e-12 | 1·26e-10 | 15·07 |
| CPM | 0·01 | 6·38e-12 | 1·26e-10 | 15·06 |
| RET | -0·01 | 1·03e-11 | 2·01e-10 | 14·59 |
| CCL11 | 0·00 | 1·16e-11 | 2·23e-10 | 14·48 |
| KIT | -0·01 | 1·69e-11 | 3·20e-10 | 14·11 |
| VSIG4 | 0·01 | 2·28e-11 | 4·27e-10 | 13·81 |
| PLAT | 0·02 | 2·49e-11 | 4·58e-10 | 13·73 |
| IGFBP3 | -0·01 | 2·67e-11 | 4·85e-10 | 13·66 |
| CD1C | -0·00 | 3·26e-11 | 5·84e-10 | 13·46 |
| DSG4 | -0·01 | 3·62e-11 | 6·40e-10 | 13·36 |
| IGF2R | 0·00 | 4·25e-11 | 7·40e-10 | 13·20 |
| EBI3_IL27 | 0·00 | 7·75e-11 | 1·33e-09 | 12·62 |
| ACE2 | 0·01 | 1·01e-10 | 1·71e-09 | 12·35 |
| FASLG | -0·01 | 1·11e-10 | 1·87e-09 | 12·26 |
| PHOSPHO1 | 0·00 | 1·14e-10 | 1·89e-09 | 12·23 |
| CCL13 | 0·01 | 1·36e-10 | 2·23e-09 | 12·06 |
| TNFSF13 | 0·00 | 1·64e-10 | 2·65e-09 | 11·88 |
| COLEC12 | 0·00 | 1·79e-10 | 2·82e-09 | 11·79 |
| LRP1 | 0·00 | 1·79e-10 | 2·82e-09 | 11·79 |
| VWC2 | 0·01 | 4·34e-10 | 6·73e-09 | 10·93 |
| CD59 | 0·00 | 4·38e-10 | 6·73e-09 | 10·92 |
| COL1A1 | -0·00 | 4·77e-10 | 7·25e-09 | 10·83 |
| IL5RA | 0·01 | 5·31e-10 | 7·97e-09 | 10·73 |
| CLMP | 0·00 | 8·09e-10 | 1·20e-08 | 10·32 |
| IL6 | 0·02 | 8·69e-10 | 1·27e-08 | 10·25 |
| TNFRSF10A | 0·00 | 1·11e-09 | 1·61e-08 | 10·01 |
| TGFBR2 | 0·00 | 1·48e-09 | 2·12e-08 | 9·73 |
| PIK3IP1 | 0·00 | 1·65e-09 | 2·34e-08 | 9·62 |
| MERTK | 0·00 | 1·68e-09 | 2·36e-08 | 9·60 |
| PSG1 | 0·02 | 1·76e-09 | 2·44e-08 | 9·56 |
| SORCS2 | 0·00 | 2·64e-09 | 3·63e-08 | 9·16 |
| SPINK4 | 0·01 | 2·75e-09 | 3·74e-08 | 9·12 |
| WISP1 | 0·00 | 3·27e-09 | 4·40e-08 | 8·95 |
| IGFBP7 | 0·00 | 3·41e-09 | 4·54e-08 | 8·91 |
| CDHR2 | 0·01 | 4·12e-09 | 5·44e-08 | 8·73 |
| GP2 | 0·02 | 4·30e-09 | 5·62e-08 | 8·69 |
| XG | 0·00 | 4·98e-09 | 6·45e-08 | 8·54 |
| SUSD2 | 0·00 | 5·27e-09 | 6·75e-08 | 8·49 |
| CRIP2 | 0·00 | 5·88e-09 | 7·46e-08 | 8·38 |
| CXCL9 | 0·01 | 5·94e-09 | 7·46e-08 | 8·37 |
| CST5 | 0·01 | 6·18e-09 | 7·69e-08 | 8·33 |
| TNXB | -0·00 | 8·80e-09 | 1·08e-07 | 7·99 |
| LAYN | 0·00 | 1·09e-08 | 1·34e-07 | 7·77 |
| EGFL7 | 0·00 | 1·11e-08 | 1·35e-07 | 7·76 |
| MFAP5 | 0·01 | 1·13e-08 | 1·36e-07 | 7·74 |
| FABP4 | 0·01 | 1·16e-08 | 1·38e-07 | 7·72 |
| FLT3 | -0·00 | 1·18e-08 | 1·39e-07 | 7·70 |
| EFEMP1 | 0·01 | 1·24e-08 | 1·45e-07 | 7·65 |
| TNFRSF10B | 0·01 | 1·29e-08 | 1·49e-07 | 7·61 |
| SPON1 | 0·00 | 1·38e-08 | 1·58e-07 | 7·55 |
| CD302 | 0·00 | 1·61e-08 | 1·83e-07 | 7·40 |
| NBL1 | 0·00 | 2·09e-08 | 2·36e-07 | 7·14 |
| COL6A3 | 0·00 | 2·97e-08 | 3·32e-07 | 6·80 |
| GGT1 | 0·01 | 3·08e-08 | 3·39e-07 | 6·77 |
| ITGB5 | 0·01 | 3·09e-08 | 3·39e-07 | 6·77 |
| ACVRL1 | 0·00 | 5·37e-08 | 5·85e-07 | 6·23 |
| CD300E | 0·00 | 6·51e-08 | 7·03e-07 | 6·04 |
| SMAD5 | 0·00 | 7·04e-08 | 7·49e-07 | 5·97 |
| TNFRSF12A | 0·00 | 7·06e-08 | 7·49e-07 | 5·96 |
| MMP7 | 0·01 | 8·55e-08 | 9·01e-07 | 5·78 |
| FOLR1 | 0·00 | 1·00e-07 | 1·04e-06 | 5·62 |
| CCN1 | 0·02 | 1·02e-07 | 1·06e-06 | 5·60 |
| GUCA2A | 0·00 | 1·07e-07 | 1·10e-06 | 5·55 |
| NTRK3 | 0·00 | 1·26e-07 | 1·28e-06 | 5·40 |
| MOG | 0·00 | 1·35e-07 | 1·37e-06 | 5·33 |
| STC1 | 0·01 | 1·36e-07 | 1·37e-06 | 5·32 |
| COL18A1 | 0·00 | 1·42e-07 | 1·42e-06 | 5·28 |
| CRH | -0·01 | 2·02e-07 | 1·99e-06 | 4·95 |
| ISM1 | 0·00 | 2·13e-07 | 2·09e-06 | 4·89 |
| FST | 0·01 | 2·16e-07 | 2·09e-06 | 4·88 |
| PROK1 | -0·01 | 2·17e-07 | 2·09e-06 | 4·88 |
| MB | 0·01 | 2·61e-07 | 2·50e-06 | 4·70 |
| GALNT10 | 0·00 | 2·64e-07 | 2·52e-06 | 4·68 |
| CD97 | 0·00 | 2·68e-07 | 2·54e-06 | 4·67 |
| HAVCR2 | 0·00 | 2·72e-07 | 2·55e-06 | 4·66 |
| CXCL10 | 0·01 | 2·80e-07 | 2·61e-06 | 4·63 |
| PCDH1 | 0·00 | 2·85e-07 | 2·63e-06 | 4·61 |
| TNFRSF1A | 0·00 | 2·87e-07 | 2·63e-06 | 4·60 |
| TNFRSF19 | 0·00 | 2·88e-07 | 2·63e-06 | 4·60 |
| LY96 | 0·00 | 3·12e-07 | 2·83e-06 | 4·52 |
| CDH2 | 0·00 | 3·57e-07 | 3·21e-06 | 4·39 |
| CD70 | 0·00 | 4·15e-07 | 3·71e-06 | 4·25 |
| CXCL16 | 0·00 | 4·28e-07 | 3·79e-06 | 4·22 |
| PODXL2 | -0·00 | 4·30e-07 | 3·79e-06 | 4·21 |
| ITGAV | 0·00 | 4·75e-07 | 4·16e-06 | 4·12 |
| CCL15 | 0·00 | 5·71e-07 | 4·97e-06 | 3·94 |
| MEPE | -0·00 | 5·78e-07 | 4·99e-06 | 3·93 |
| FABP9 | -0·01 | 6·26e-07 | 5·38e-06 | 3·85 |
| ENPP5 | -0·00 | 6·71e-07 | 5·72e-06 | 3·78 |
| FCRLB | 0·01 | 7·09e-07 | 6·01e-06 | 3·73 |
| NOS1 | 0·01 | 7·59e-07 | 6·39e-06 | 3·67 |
| CA9 | 0·01 | 7·68e-07 | 6·43e-06 | 3·65 |
| DCBLD2 | 0·00 | 8·01e-07 | 6·67e-06 | 3·61 |
| BCAM | 0·00 | 8·64e-07 | 7·14e-06 | 3·54 |
| TNFRSF11A | 0·00 | 1·02e-06 | 8·40e-06 | 3·38 |
| CTSC | 0·00 | 1·09e-06 | 8·96e-06 | 3·31 |
| FCRL1 | -0·00 | 1·13e-06 | 9·22e-06 | 3·27 |
| LGALS9 | 0·00 | 1·22e-06 | 9·83e-06 | 3·21 |
| CFC1 | 0·00 | 1·29e-06 | 1·03e-05 | 3·15 |
| WIF1 | -0·00 | 1·39e-06 | 1·10e-05 | 3·08 |
| C1QA | 0·00 | 1·43e-06 | 1·13e-05 | 3·05 |
| PTS | 0·01 | 1·46e-06 | 1·15e-05 | 3·03 |
| IGF1R | 0·00 | 1·48e-06 | 1·16e-05 | 3·02 |
| JCHAIN | 0·01 | 1·69e-06 | 1·32e-05 | 2·89 |
| LRPAP1 | 0·01 | 1·79e-06 | 1·38e-05 | 2·83 |
| CHIT1 | 0·03 | 1·85e-06 | 1·42e-05 | 2·80 |
| TINAGL1 | 0·00 | 2·01e-06 | 1·53e-05 | 2·73 |
| GFRA1 | 0·00 | 2·86e-06 | 2·17e-05 | 2·39 |
| LEP | 0·02 | 3·15e-06 | 2·38e-05 | 2·29 |
| CA3 | 0·01 | 3·66e-06 | 2·75e-05 | 2·15 |
| MAD1L1 | 0·00 | 4·17e-06 | 3·11e-05 | 2·02 |
| LILRB4 | 0·00 | 4·89e-06 | 3·62e-05 | 1·87 |
| PRSS8 | 0·00 | 5·29e-06 | 3·90e-05 | 1·80 |
| CTSF | 0·00 | 6·59e-06 | 4·83e-05 | 1·58 |
| NRP2 | 0·00 | 7·18e-06 | 5·24e-05 | 1·50 |
| FUT3_FUT5 | 0·00 | 7·66e-06 | 5·55e-05 | 1·44 |
| CD74 | 0·00 | 8·25e-06 | 5·95e-05 | 1·37 |
| PLXNB2 | 0·00 | 8·35e-06 | 5·99e-05 | 1·36 |
| CDH15 | 0·01 | 8·99e-06 | 6·42e-05 | 1·29 |
| HNMT | 0·01 | 9·66e-06 | 6·86e-05 | 1·22 |
| KRT18 | 0·01 | 1·01e-05 | 7·14e-05 | 1·17 |
| ADGRE2 | 0·00 | 1·06e-05 | 7·50e-05 | 1·12 |
| VWF | 0·01 | 1·09e-05 | 7·66e-05 | 1·10 |
| SMPD1 | 0·00 | 1·27e-05 | 8·83e-05 | 0·96 |
| FAS | 0·00 | 1·28e-05 | 8·90e-05 | 0·94 |
| ADGRG2 | -0·00 | 1·50e-05 | 0·00 | 0·79 |
| CKAP4 | 0·00 | 1·55e-05 | 0·00 | 0·76 |
| GSTA1 | 0·01 | 1·57e-05 | 0·00 | 0·75 |
| MUC13 | 0·00 | 1·85e-05 | 0·00 | 0·60 |
| ANGPTL4 | 0·00 | 1·90e-05 | 0·00 | 0·57 |
| ERBB4 | 0·00 | 1·92e-05 | 0·00 | 0·56 |
| LAIR1 | 0·00 | 1·92e-05 | 0·00 | 0·56 |
| KDR | -0·00 | 1·94e-05 | 0·00 | 0·55 |
| VCAN | 0·00 | 1·99e-05 | 0·00 | 0·52 |
| DCXR | 0·01 | 2·79e-05 | 0·00 | 0·20 |
| ENPP7 | 0·01 | 2·92e-05 | 0·00 | 0·16 |
| BCAN | -0·00 | 2·95e-05 | 0·00 | 0·15 |
| DCTPP1 | 0·00 | 3·26e-05 | 0·00 | 0·06 |
| AGRN | 0·00 | 3·36e-05 | 0·00 | 0·03 |
| SSC5D | 0·00 | 3·39e-05 | 0·00 | 0·02 |
| TGFB1 | 0·00 | 3·39e-05 | 0·00 | 0·02 |
| DKKL1 | 0·01 | 3·43e-05 | 0·00 | 0·01 |
| NT5E | 0·00 | 3·49e-05 | 0·00 | -0·00 |
| TNFSF13B | 0·00 | 3·50e-05 | 0·00 | -0·00 |
| CEACAM5 | 0·01 | 3·54e-05 | 0·00 | -0·01 |
| F3 | 0·00 | 3·54e-05 | 0·00 | -0·01 |
| PLA2G10 | 0·01 | 3·77e-05 | 0·00 | -0·07 |
| HGF | 0·00 | 3·93e-05 | 0·00 | -0·11 |
| FLT1 | 0·00 | 4·20e-05 | 0·00 | -0·17 |
| AFP | 0·01 | 4·25e-05 | 0·00 | -0·19 |
| ANXA5 | 0·00 | 4·79e-05 | 0·00 | -0·30 |
| MDK | 0·00 | 5·12e-05 | 0·00 | -0·36 |
| CRIM1 | 0·00 | 5·16e-05 | 0·00 | -0·37 |
| CA14 | -0·00 | 5·39e-05 | 0·00 | -0·41 |
| AREG | 0·00 | 5·63e-05 | 0·00 | -0·45 |
| NPDC1 | 0·00 | 5·77e-05 | 0·00 | -0·48 |
| EPHA2 | 0·00 | 5·87e-05 | 0·00 | -0·49 |
| CCL18 | 0·01 | 5·90e-05 | 0·00 | -0·50 |
| PLIN1 | 0·00 | 6·66e-05 | 0·00 | -0·61 |
| REG4 | 0·00 | 6·67e-05 | 0·00 | -0·61 |
| CCL7 | 0·00 | 6·86e-05 | 0·00 | -0·64 |
| COL9A1 | -0·00 | 7·36e-05 | 0·00 | -0·71 |
| PILRA | 0·00 | 7·56e-05 | 0·00 | -0·73 |
| CXCL13 | 0·01 | 8·47e-05 | 0·00 | -0·84 |
| IL17A | -0·00 | 8·97e-05 | 0·00 | -0·89 |
| SPOCK1 | 0·00 | 9·05e-05 | 0·00 | -0·90 |
| THBS4 | 0·01 | 9·41e-05 | 0·00 | -0·94 |
| GSTA3 | 0·01 | 9·57e-05 | 0·00 | -0·96 |
| SIAE | 0·00 | 9·60e-05 | 0·00 | -0·96 |
| GFRA3 | 0·00 | 0·00 | 0·00 | -1·06 |
| BOC | -0·00 | 0·00 | 0·00 | -1·13 |
| ODAM | -0·00 | 0·00 | 0·00 | -1·19 |
| EGFR | -0·00 | 0·00 | 0·00 | -1·28 |
| BRK1 | 0·00 | 0·00 | 0·00 | -1·36 |
| SPON2 | 0·00 | 0·00 | 0·00 | -1·39 |
| LYPD3 | -0·00 | 0·00 | 0·00 | -1·44 |
| IL19 | 0·01 | 0·00 | 0·00 | -1·48 |
| FCN2 | -0·00 | 0·00 | 0·00 | -1·51 |
| LGALS7_LGALS7B | 0·00 | 0·00 | 0·00 | -1·61 |
| GPR37 | 0·01 | 0·00 | 0·00 | -1·63 |
| LAG3 | 0·00 | 0·00 | 0·00 | -1·70 |
| KRT19 | 0·01 | 0·00 | 0·00 | -1·71 |
| MMP13 | -0·00 | 0·00 | 0·00 | -1·79 |
| FOLR2 | 0·00 | 0·00 | 0·00 | -1·83 |
| CD276 | 0·00 | 0·00 | 0·00 | -1·88 |
| RELT | 0·00 | 0·00 | 0·00 | -1·89 |
| SERPINA11 | 0·00 | 0·00 | 0·00 | -1·94 |
| FGF19 | 0·01 | 0·00 | 0·00 | -1·98 |
| LGALS1 | 0·00 | 0·00 | 0·00 | -1·99 |
| CSTB | 0·00 | 0·00 | 0·00 | -2·01 |
| IGFBP2 | 0·00 | 0·00 | 0·00 | -2·02 |
| DPP4 | -0·00 | 0·00 | 0·00 | -2·02 |
| DSG3 | -0·00 | 0·00 | 0·00 | -2·03 |
| CD79B | 0·00 | 0·00 | 0·00 | -2·08 |
| IDUA | 0·00 | 0·00 | 0·00 | -2·08 |
| THBS2 | 0·00 | 0·00 | 0·00 | -2·10 |
| CA4 | -0·00 | 0·00 | 0·00 | -2·10 |
| PDGFRB | 0·00 | 0·00 | 0·00 | -2·14 |
| WFIKKN1 | -0·00 | 0·00 | 0·00 | -2·15 |
| CCL16 | 0·00 | 0·00 | 0·00 | -2·17 |
| FBP1 | 0·01 | 0·00 | 0·00 | -2·18 |
| FAM3C | 0·00 | 0·00 | 0·00 | -2·20 |
| JAM2 | 0·00 | 0·00 | 0·00 | -2·23 |
| LAMP3 | 0·00 | 0·00 | 0·00 | -2·24 |
| CNTN3 | 0·00 | 0·00 | 0·00 | -2·26 |
| NPTX1 | 0·00 | 0·00 | 0·00 | -2·26 |
| VMO1 | 0·00 | 0·00 | 0·00 | -2·26 |
| LGALS4 | 0·00 | 0·00 | 0·00 | -2·27 |
| LGALS3 | 0·00 | 0·00 | 0·00 | -2·34 |
| GRPEL1 | 0·00 | 0·00 | 0·00 | -2·37 |
| CD99 | 0·00 | 0·00 | 0·00 | -2·40 |
| DSC2 | 0·00 | 0·00 | 0·00 | -2·43 |
| SIGLEC1 | 0·00 | 0·00 | 0·00 | -2·43 |
| PLTP | 0·00 | 0·00 | 0·00 | -2·47 |
| AGER | -0·00 | 0·00 | 0·00 | -2·52 |
| ARSA | 0·00 | 0·00 | 0·00 | -2·56 |
| EFNA1 | 0·00 | 0·00 | 0·00 | -2·60 |
| COMP | 0·00 | 0·00 | 0·00 | -2·61 |
| CDNF | 0·00 | 0·00 | 0·00 | -2·64 |
| KEL | 0·00 | 0·00 | 0·00 | -2·67 |
| ANGPT2 | 0·00 | 0·00 | 0·00 | -2·68 |
| APOH | 0·00 | 0·00 | 0·00 | -2·69 |
| ISLR2 | -0·00 | 0·00 | 0·00 | -2·69 |
| ALCAM | 0·00 | 0·00 | 0·00 | -2·76 |
| L1CAM | 0·00 | 0·00 | 0·00 | -2·78 |
| DRAXIN | 0·00 | 0·00 | 0·00 | -2·78 |
| PI3 | 0·00 | 0·00 | 0·00 | -2·79 |
| LILRA2 | 0·00 | 0·00 | 0·00 | -2·81 |
| TFPI2 | 0·00 | 0·00 | 0·00 | -2·81 |
| AXL | -0·00 | 0·00 | 0·00 | -2·84 |
| PTPRS | -0·00 | 0·00 | 0·00 | -2·85 |
| CTSL | 0·00 | 0·00 | 0·00 | -2·85 |
| CDHR1 | -0·00 | 0·00 | 0·00 | -2·86 |
| ADA2 | 0·00 | 0·00 | 0·00 | -2·86 |
| CHRDL2 | 0·00 | 0·00 | 0·00 | -2·89 |
| TACSTD2 | 0·00 | 0·00 | 0·00 | -2·90 |
| HLA-E | 0·00 | 0·00 | 0·00 | -2·91 |
| SLC39A5 | 0·00 | 0·00 | 0·00 | -2·94 |
| SETMAR | 0·00 | 0·00 | 0·00 | -2·96 |
| TFF1 | 0·01 | 0·00 | 0·00 | -2·96 |
| ANGPTL7 | 0·00 | 0·00 | 0·00 | -2·96 |
| SCGB1A1 | 0·00 | 0·00 | 0·00 | -2·99 |
| ADH4 | 0·01 | 0·00 | 0·00 | -3·02 |
| TFF2 | 0·00 | 0·00 | 0·00 | -3·08 |
| TNFRSF8 | -0·00 | 0·00 | 0·00 | -3·09 |
| EFNA4 | 0·00 | 0·00 | 0·00 | -3·09 |
| KLK8 | -0·00 | 0·00 | 0·00 | -3·12 |
| CLSTN2 | 0·00 | 0·00 | 0·00 | -3·20 |
| ANGPTL1 | 0·00 | 0·00 | 0·00 | -3·20 |
| FGFR2 | 0·00 | 0·00 | 0·00 | -3·20 |
| TNFRSF13C | -0·00 | 0·00 | 0·00 | -3·23 |
| SULT2A1 | 0·00 | 0·00 | 0·00 | -3·25 |
| TYRO3 | -0·00 | 0·00 | 0·00 | -3·28 |
| FABP1 | 0·01 | 0·00 | 0·00 | -3·28 |
| GCG | 0·01 | 0·00 | 0·00 | -3·32 |
| CALCA | 0·00 | 0·00 | 0·00 | -3·34 |
| SEMA4D | -0·00 | 0·00 | 0·00 | -3·38 |
| PPP3R1 | 0·00 | 0·00 | 0·00 | -3·42 |
| CD109 | 0·00 | 0·00 | 0·00 | -3·45 |
| SORT1 | 0·00 | 0·00 | 0·00 | -3·45 |
| SLC16A1 | 0·00 | 0·00 | 0·00 | -3·45 |
| IL10 | -0·01 | 0·00 | 0·00 | -3·48 |
| ANPEP | -0·00 | 0·00 | 0·00 | -3·48 |
| CTSH | 0·00 | 0·00 | 0·00 | -3·51 |
| VEGFA | 0·00 | 0·00 | 0·00 | -3·51 |
| IGSF3 | 0·00 | 0·00 | 0·00 | -3·52 |
| ENAH | 0·00 | 0·00 | 0·00 | -3·55 |
| CD5 | -0·00 | 0·00 | 0·00 | -3·56 |
| CCL3 | 0·00 | 0·00 | 0·00 | -3·56 |
| RTN4R | 0·00 | 0·00 | 0·00 | -3·58 |
| CPA1 | 0·00 | 0·00 | 0·00 | -3·62 |
| PON3 | -0·00 | 0·00 | 0·00 | -3·64 |
| CLEC14A | 0·00 | 0·00 | 0·00 | -3·65 |
| NAAA | 0·00 | 0·00 | 0·00 | -3·73 |
| ASGR1 | 0·00 | 0·00 | 0·00 | -3·75 |
| ITGA5 | 0·00 | 0·00 | 0·00 | -3·79 |
| SMPDL3A | 0·00 | 0·00 | 0·00 | -3·83 |
| ACAN | 0·00 | 0·00 | 0·00 | -3·84 |
| KLK1 | 0·01 | 0·00 | 0·00 | -3·85 |
| PAMR1 | 0·00 | 0·00 | 0·00 | -3·85 |
| AGXT | 0·00 | 0·00 | 0·00 | -3·85 |
| AZU1 | -0·01 | 0·00 | 0·00 | -3·86 |
| EPO | 0·00 | 0·00 | 0·00 | -3·90 |
| PLA2G15 | 0·00 | 0·00 | 0·00 | -3·92 |
| MYOC | 0·00 | 0·00 | 0·00 | -3·93 |
| CNTN5 | 0·00 | 0·00 | 0·00 | -4·01 |
| CBLIF | 0·00 | 0·00 | 0·01 | -4·11 |
| NCF2 | -0·01 | 0·00 | 0·01 | -4·14 |
| PDGFRA | 0·00 | 0·00 | 0·01 | -4·15 |
| CD22 | -0·00 | 0·00 | 0·01 | -4·15 |
| INHBC | 0·00 | 0·00 | 0·01 | -4·15 |
| RBP5 | 0·00 | 0·00 | 0·01 | -4·21 |
| APOM | -0·00 | 0·00 | 0·01 | -4·23 |
| MAPK9 | 0·00 | 0·00 | 0·01 | -4·24 |
| SEMA7A | 0·00 | 0·00 | 0·01 | -4·24 |
| CLPS | 0·00 | 0·00 | 0·01 | -4·25 |
| NTF3 | -0·00 | 0·00 | 0·01 | -4·25 |
| CPB1 | 0·00 | 0·00 | 0·01 | -4·28 |
| OPTC | 0·00 | 0·00 | 0·01 | -4·29 |
| TNFSF10 | -0·00 | 0·00 | 0·01 | -4·30 |
| MARCO | 0·00 | 0·00 | 0·01 | -4·31 |
| SFTPD | 0·00 | 0·00 | 0·01 | -4·34 |
| IGSF8 | 0·00 | 0·00 | 0·01 | -4·35 |
| NINJ1 | 0·00 | 0·00 | 0·01 | -4·38 |
| ADAM22 | 0·00 | 0·00 | 0·01 | -4·38 |
| IL15RA | 0·00 | 0·00 | 0·01 | -4·40 |
| CES1 | 0·00 | 0·00 | 0·01 | -4·40 |
| CHGB | 0·00 | 0·00 | 0·01 | -4·45 |
| DPP6 | 0·00 | 0·00 | 0·01 | -4·45 |
| CD300C | 0·00 | 0·00 | 0·01 | -4·47 |
| LY6D | 0·00 | 0·00 | 0·01 | -4·48 |
| TNFRSF1B | 0·00 | 0·00 | 0·01 | -4·49 |
| GPKOW | 0·00 | 0·00 | 0·01 | -4·50 |
| PM20D1 | 0·02 | 0·00 | 0·01 | -4·50 |
| KLK13 | -0·00 | 0·00 | 0·01 | -4·51 |
| NPPC | -0·00 | 0·00 | 0·01 | -4·55 |
| GAL | -0·00 | 0·00 | 0·01 | -4·55 |
| CST6 | -0·00 | 0·00 | 0·01 | -4·57 |
| IL10RB | 0·00 | 0·00 | 0·01 | -4·58 |
| ADGRB3 | 0·00 | 0·00 | 0·01 | -4·59 |
| ENG | -0·00 | 0·00 | 0·01 | -4·61 |
| SKAP1 | -0·00 | 0·00 | 0·01 | -4·65 |
| IL6R | -0·00 | 0·00 | 0·01 | -4·67 |
| SLAMF8 | 0·00 | 0·00 | 0·01 | -4·67 |
| CALB1 | 0·00 | 0·00 | 0·01 | -4·69 |
| MSLN | 0·00 | 0·00 | 0·01 | -4·69 |
| TREML2 | -0·00 | 0·00 | 0·01 | -4·70 |
| SMOC2 | 0·00 | 0·00 | 0·01 | -4·74 |
| LRP11 | 0·00 | 0·00 | 0·01 | -4·76 |
| LRRN1 | -0·00 | 0·00 | 0·01 | -4·77 |
| BAIAP2 | 0·00 | 0·00 | 0·01 | -4·77 |
| DPP7 | 0·00 | 0·00 | 0·02 | -4·82 |
| CDH6 | 0·00 | 0·00 | 0·02 | -4·85 |
| GGT5 | 0·00 | 0·00 | 0·02 | -4·86 |
| MCAM | 0·00 | 0·00 | 0·02 | -4·88 |
| DPY30 | 0·00 | 0·00 | 0·02 | -4·90 |
| PLAU | 0·00 | 0·00 | 0·02 | -4·91 |
| ART3 | -0·00 | 0·00 | 0·02 | -4·92 |
| EPS8L2 | 0·00 | 0·00 | 0·02 | -4·92 |
| CCL2 | 0·00 | 0·00 | 0·02 | -4·94 |
| PCSK9 | 0·00 | 0·00 | 0·02 | -4·95 |
| NOS3 | 0·00 | 0·00 | 0·02 | -4·95 |
| CDH3 | -0·00 | 0·00 | 0·02 | -4·96 |
| ANXA3 | -0·00 | 0·00 | 0·02 | -4·97 |
| MILR1 | 0·00 | 0·00 | 0·02 | -4·99 |
| GP1BA | -0·00 | 0·00 | 0·02 | -5·02 |
| SIT1 | -0·00 | 0·00 | 0·02 | -5·04 |
| TNFRSF14 | 0·00 | 0·00 | 0·02 | -5·06 |
| KIRREL2 | -0·00 | 0·00 | 0·02 | -5·06 |
| CA5A | 0·00 | 0·00 | 0·02 | -5·08 |
| GPNMB | 0·00 | 0·00 | 0·02 | -5·09 |
| FGF21 | 0·01 | 0·00 | 0·02 | -5·11 |
| PLA2G2A | 0·00 | 0·00 | 0·02 | -5·11 |
| GHRL | 0·00 | 0·00 | 0·02 | -5·13 |
| AMY2A | 0·00 | 0·00 | 0·02 | -5·15 |
| DDR1 | 0·00 | 0·00 | 0·02 | -5·18 |
| IL15 | 0·00 | 0·00 | 0·02 | -5·20 |
| SPINK1 | 0·00 | 0·00 | 0·02 | -5·20 |
| BTN3A2 | 0·00 | 0·00 | 0·02 | -5·21 |
| CD27 | 0·00 | 0·00 | 0·02 | -5·21 |
| ANGPTL2 | 0·00 | 0·00 | 0·02 | -5·21 |
| CA6 | -0·00 | 0·00 | 0·02 | -5·22 |
| PLAUR | 0·00 | 0·00 | 0·02 | -5·24 |
| FLRT2 | 0·00 | 0·00 | 0·03 | -5·24 |
| PDCD1 | -0·00 | 0·00 | 0·03 | -5·25 |
| CNTN2 | 0·00 | 0·01 | 0·03 | -5·26 |
| PIGR | 0·00 | 0·01 | 0·03 | -5·28 |
| CNTNAP2 | 0·00 | 0·01 | 0·03 | -5·29 |
| ANXA10 | 0·00 | 0·01 | 0·03 | -5·33 |
| ADAMTS13 | -0·00 | 0·01 | 0·03 | -5·34 |
| CX3CL1 | 0·00 | 0·01 | 0·03 | -5·35 |
| SORD | 0·00 | 0·01 | 0·03 | -5·36 |
| TXNDC5 | 0·00 | 0·01 | 0·03 | -5·36 |
| SIGLEC7 | 0·00 | 0·01 | 0·03 | -5·36 |
| VASN | -0·00 | 0·01 | 0·03 | -5·37 |
| HAO1 | 0·01 | 0·01 | 0·03 | -5·37 |
| CPE | 0·00 | 0·01 | 0·03 | -5·41 |
| IL12RB1 | 0·00 | 0·01 | 0·03 | -5·42 |
| CD93 | -0·00 | 0·01 | 0·03 | -5·43 |
| ADAMTS8 | 0·00 | 0·01 | 0·03 | -5·45 |
| ALDH1A1 | 0·00 | 0·01 | 0·03 | -5·46 |
| LTA | -0·00 | 0·01 | 0·03 | -5·49 |
| CA12 | 0·00 | 0·01 | 0·03 | -5·52 |
| IL1RL1 | 0·00 | 0·01 | 0·04 | -5·56 |
| C19orf12 | 0·00 | 0·01 | 0·04 | -5·56 |
| CDHR5 | -0·00 | 0·01 | 0·04 | -5·58 |
| DLK1 | 0·00 | 0·01 | 0·04 | -5·59 |
| RARRES2 | 0·00 | 0·01 | 0·04 | -5·59 |
| IL1B | -0·01 | 0·01 | 0·04 | -5·59 |
| RNASET2 | 0·00 | 0·01 | 0·04 | -5·60 |
| BMP6 | 0·00 | 0·01 | 0·04 | -5·62 |
| PILRB | 0·00 | 0·01 | 0·04 | -5·64 |
| THOP1 | -0·00 | 0·01 | 0·04 | -5·65 |
| CCL8 | 0·00 | 0·01 | 0·04 | -5·66 |
| PLXDC1 | -0·00 | 0·01 | 0·04 | -5·66 |
| RGMA | -0·00 | 0·01 | 0·04 | -5·70 |
| ENTPD5 | 0·00 | 0·01 | 0·04 | -5·70 |
| LILRB2 | 0·00 | 0·01 | 0·04 | -5·71 |
| BGN | 0·01 | 0·01 | 0·04 | -5·71 |
| CCL17 | 0·00 | 0·01 | 0·04 | -5·76 |
| ACP5 | 0·00 | 0·01 | 0·04 | -5·76 |
| CSF1 | 0·00 | 0·01 | 0·04 | -5·76 |
| ROBO1 | -0·00 | 0·01 | 0·04 | -5·76 |
| NPY | 0·00 | 0·01 | 0·05 | -5·78 |
| S100A4 | -0·00 | 0·01 | 0·05 | -5·79 |
| NECTIN2 | 0·00 | 0·01 | 0·05 | -5·80 |
| IL1RAP | -0·00 | 0·01 | 0·05 | -5·80 |
| ERBB3 | -0·00 | 0·01 | 0·05 | -5·81 |
| AMY2B | 0·00 | 0·01 | 0·05 | -5·83 |
| SCGN | 0·00 | 0·01 | 0·05 | -5·84 |
| HCLS1 | -0·00 | 0·01 | 0·05 | -5·86 |
| MSTN | -0·00 | 0·01 | 0·05 | -5·86 |
| CST3 | 0·00 | 0·02 | 0·05 | -5·87 |
| CD84 | -0·00 | 0·02 | 0·05 | -5·88 |
| REN | 0·00 | 0·02 | 0·05 | -5·90 |
| TNFRSF6B | 0·00 | 0·02 | 0·05 | -5·90 |
| NMNAT1 | 0·00 | 0·02 | 0·05 | -5·91 |
| CD34 | -0·00 | 0·02 | 0·05 | -5·92 |
| VSTM1 | -0·00 | 0·02 | 0·05 | -5·93 |
| CNDP1 | -0·00 | 0·02 | 0·06 | -5·97 |
| IL32 | 0·00 | 0·02 | 0·06 | -6·00 |
| TAFA5 | 0·00 | 0·02 | 0·06 | -6·01 |
| NPTXR | -0·00 | 0·02 | 0·06 | -6·02 |
| FURIN | 0·00 | 0·02 | 0·06 | -6·04 |
| MZB1 | 0·00 | 0·02 | 0·06 | -6·04 |
| TSHB | 0·00 | 0·02 | 0·06 | -6·04 |
| IL7R | -0·00 | 0·02 | 0·06 | -6·07 |
| CCL28 | 0·00 | 0·02 | 0·06 | -6·08 |
| MFGE8 | 0·00 | 0·02 | 0·06 | -6·08 |
| NCAN | -0·00 | 0·02 | 0·06 | -6·08 |
| DKK1 | 0·00 | 0·02 | 0·07 | -6·12 |
| CLEC7A | 0·00 | 0·02 | 0·07 | -6·13 |
| CLEC4C | -0·00 | 0·02 | 0·07 | -6·14 |
| FRZB | 0·00 | 0·02 | 0·07 | -6·15 |
| ADAM8 | -0·00 | 0·02 | 0·07 | -6·16 |
| SIGLEC10 | 0·00 | 0·02 | 0·07 | -6·18 |
| S100A11 | -0·00 | 0·02 | 0·07 | -6·20 |
| LIFR | 0·00 | 0·03 | 0·07 | -6·23 |
| PRTN3 | -0·00 | 0·03 | 0·07 | -6·24 |
| HGS | -0·00 | 0·03 | 0·08 | -6·26 |
| NUCB2 | 0·00 | 0·03 | 0·08 | -6·26 |
| BCL2L11 | 0·00 | 0·03 | 0·08 | -6·27 |
| FGF23 | 0·00 | 0·03 | 0·08 | -6·28 |
| FETUB | -0·00 | 0·03 | 0·08 | -6·28 |
| NCAM2 | -0·00 | 0·03 | 0·08 | -6·29 |
| CD99L2 | 0·00 | 0·03 | 0·08 | -6·30 |
| KYNU | 0·00 | 0·03 | 0·08 | -6·31 |
| MNDA | -0·00 | 0·03 | 0·08 | -6·31 |
| P4HB | 0·00 | 0·03 | 0·08 | -6·31 |
| LILRB5 | 0·00 | 0·03 | 0·08 | -6·34 |
| WARS | 0·00 | 0·03 | 0·08 | -6·35 |
| EPCAM | -0·00 | 0·03 | 0·08 | -6·35 |
| CLEC4A | -0·00 | 0·03 | 0·08 | -6·35 |
| ST6GAL1 | 0·00 | 0·03 | 0·08 | -6·37 |
| ESAM | 0·00 | 0·03 | 0·08 | -6·37 |
| LTBR | 0·00 | 0·03 | 0·09 | -6·39 |
| TBC1D17 | 0·00 | 0·03 | 0·09 | -6·40 |
| CPXM1 | 0·00 | 0·03 | 0·09 | -6·40 |
| CRNN | -0·00 | 0·03 | 0·09 | -6·42 |
| PECAM1 | 0·00 | 0·03 | 0·09 | -6·43 |
| PVR | 0·00 | 0·03 | 0·09 | -6·43 |
| CD40 | 0·00 | 0·03 | 0·09 | -6·43 |
| RTBDN | 0·00 | 0·03 | 0·09 | -6·44 |
| CD6 | -0·00 | 0·03 | 0·09 | -6·44 |
| CREG1 | 0·00 | 0·04 | 0·09 | -6·47 |
| TNFRSF10C | 0·00 | 0·04 | 0·09 | -6·47 |
| IL1R1 | 0·00 | 0·04 | 0·09 | -6·47 |
| ST3GAL1 | 0·00 | 0·04 | 0·09 | -6·48 |
| MMP1 | 0·00 | 0·04 | 0·09 | -6·48 |
| MATN3 | 0·00 | 0·04 | 0·09 | -6·48 |
| CDH5 | -0·00 | 0·04 | 0·10 | -6·50 |
| COL4A1 | 0·00 | 0·04 | 0·10 | -6·50 |
| ACY1 | 0·00 | 0·04 | 0·10 | -6·52 |
| CXCL11 | 0·00 | 0·04 | 0·10 | -6·52 |
| TIMP3 | 0·01 | 0·04 | 0·10 | -6·53 |
| SEMA3F | 0·00 | 0·04 | 0·10 | -6·53 |
| IQGAP2 | 0·00 | 0·04 | 0·10 | -6·55 |
| TIMP1 | 0·00 | 0·04 | 0·10 | -6·56 |
| GALNT7 | -0·00 | 0·04 | 0·10 | -6·57 |
| ARG1 | -0·00 | 0·04 | 0·10 | -6·57 |
| LRRC25 | 0·00 | 0·04 | 0·10 | -6·58 |
| ITGB6 | 0·00 | 0·04 | 0·11 | -6·59 |
| SERPINB9 | -0·00 | 0·04 | 0·11 | -6·61 |
| PRCP | 0·00 | 0·04 | 0·11 | -6·61 |
| KIR3DL1 | -0·01 | 0·04 | 0·11 | -6·62 |
| IL4R | 0·00 | 0·04 | 0·11 | -6·64 |
| MEGF10 | -0·00 | 0·04 | 0·11 | -6·64 |
| SH2B3 | -0·00 | 0·04 | 0·11 | -6·65 |
| CD200R1 | -0·00 | 0·05 | 0·12 | -6·67 |
| ITIH3 | 0·00 | 0·05 | 0·12 | -6·68 |
| LDLR | 0·00 | 0·05 | 0·12 | -6·68 |
| VTA1 | -0·00 | 0·05 | 0·12 | -6·69 |
| HMOX2 | 0·00 | 0·05 | 0·12 | -6·70 |
| HS3ST3B1 | 0·00 | 0·05 | 0·12 | -6·71 |
| KLRD1 | 0·00 | 0·05 | 0·12 | -6·72 |
| CLPP | 0·00 | 0·05 | 0·12 | -6·73 |
| ABHD14B | 0·00 | 0·05 | 0·13 | -6·76 |
| LILRB1 | 0·00 | 0·05 | 0·13 | -6·77 |
| MANSC1 | -0·00 | 0·05 | 0·13 | -6·77 |
| OBP2B | -0·00 | 0·05 | 0·13 | -6·77 |
| APLP1 | -0·00 | 0·05 | 0·13 | -6·79 |
| PRSS2 | 0·00 | 0·05 | 0·13 | -6·79 |
| UMOD | -0·00 | 0·05 | 0·13 | -6·79 |
| CD164 | 0·00 | 0·05 | 0·13 | -6·79 |
| HMOX1 | 0·00 | 0·06 | 0·14 | -6·83 |
| MET | -0·00 | 0·06 | 0·14 | -6·83 |
| ITGA6 | -0·00 | 0·06 | 0·14 | -6·83 |
| CGREF1 | 0·00 | 0·06 | 0·14 | -6·83 |
| CCL14 | 0·00 | 0·06 | 0·14 | -6·84 |
| SCG2 | 0·00 | 0·06 | 0·14 | -6·86 |
| SERPINA9 | -0·00 | 0·06 | 0·14 | -6·86 |
| BTN2A1 | 0·00 | 0·06 | 0·14 | -6·87 |
| TMPRSS15 | 0·00 | 0·06 | 0·14 | -6·88 |
| IL1RL2 | 0·00 | 0·06 | 0·14 | -6·88 |
| PAEP | 0·00 | 0·06 | 0·14 | -6·88 |
| CCL25 | 0·00 | 0·06 | 0·14 | -6·88 |
| CCL23 | 0·00 | 0·06 | 0·15 | -6·90 |
| ROR1 | 0·00 | 0·06 | 0·15 | -6·91 |
| FMNL1 | -0·00 | 0·06 | 0·15 | -6·92 |
| DDAH1 | 0·00 | 0·06 | 0·15 | -6·93 |
| LTBP3 | 0·00 | 0·06 | 0·15 | -6·94 |
| REG1B | 0·00 | 0·06 | 0·15 | -6·94 |
| GNLY | 0·00 | 0·07 | 0·15 | -6·94 |
| CD163 | 0·00 | 0·07 | 0·15 | -6·95 |
| SPINT2 | 0·00 | 0·07 | 0·15 | -6·95 |
| C4BPB | 0·00 | 0·07 | 0·15 | -6·96 |
| NTRK2 | 0·00 | 0·07 | 0·15 | -6·96 |
| GPA33 | -0·00 | 0·07 | 0·15 | -6·96 |
| LPO | -0·00 | 0·07 | 0·16 | -6·97 |
| IL6ST | 0·00 | 0·07 | 0·16 | -6·97 |
| DEFB4A_DEFB4B | 0·01 | 0·07 | 0·16 | -6·97 |
| MVK | 0·00 | 0·07 | 0·16 | -6·98 |
| MDGA1 | 0·00 | 0·07 | 0·16 | -6·98 |
| DPP10 | 0·00 | 0·07 | 0·16 | -6·98 |
| CHAC2 | 0·00 | 0·07 | 0·16 | -6·98 |
| PAM | 0·00 | 0·07 | 0·16 | -7·00 |
| CST7 | -0·00 | 0·07 | 0·16 | -7·00 |
| TFF3 | 0·00 | 0·07 | 0·16 | -7·00 |
| ZBTB17 | 0·00 | 0·07 | 0·16 | -7·01 |
| TCN2 | 0·00 | 0·07 | 0·16 | -7·01 |
| CLC | -0·00 | 0·07 | 0·16 | -7·02 |
| KLK11 | 0·00 | 0·07 | 0·16 | -7·02 |
| CLEC11A | 0·00 | 0·07 | 0·16 | -7·02 |
| LGMN | -0·00 | 0·07 | 0·16 | -7·03 |
| LILRA5 | 0·00 | 0·08 | 0·17 | -7·05 |
| CLUL1 | 0·00 | 0·08 | 0·17 | -7·06 |
| NME3 | -0·00 | 0·08 | 0·17 | -7·07 |
| CASP1 | -0·00 | 0·08 | 0·17 | -7·08 |
| LRIG1 | 0·00 | 0·08 | 0·17 | -7·08 |
| HSPG2 | 0·00 | 0·08 | 0·17 | -7·09 |
| CTSD | 0·00 | 0·08 | 0·18 | -7·10 |
| ROBO2 | 0·00 | 0·08 | 0·18 | -7·10 |
| TXNRD1 | 0·00 | 0·08 | 0·18 | -7·12 |
| FCGR3B | -0·00 | 0·08 | 0·18 | -7·12 |
| PRSS27 | -0·00 | 0·08 | 0·18 | -7·12 |
| IFNGR1 | 0·00 | 0·08 | 0·18 | -7·12 |
| PRKAB1 | 0·00 | 0·08 | 0·18 | -7·12 |
| CES2 | 0·00 | 0·09 | 0·18 | -7·14 |
| NCAM1 | -0·00 | 0·09 | 0·19 | -7·15 |
| MME | 0·00 | 0·09 | 0·19 | -7·15 |
| EDIL3 | 0·00 | 0·09 | 0·19 | -7·16 |
| TNC | -0·00 | 0·09 | 0·19 | -7·17 |
| TNF | 0·00 | 0·09 | 0·19 | -7·17 |
| S100A12 | -0·00 | 0·09 | 0·19 | -7·18 |
| ENTPD2 | -0·00 | 0·09 | 0·20 | -7·20 |
| CTRB1 | 0·00 | 0·09 | 0·20 | -7·20 |
| DNER | -0·00 | 0·09 | 0·20 | -7·20 |
| FOXO1 | -0·00 | 0·09 | 0·20 | -7·22 |
| TGFBR3 | 0·00 | 0·10 | 0·20 | -7·23 |
| TPPP3 | 0·00 | 0·10 | 0·20 | -7·23 |
| CTSZ | 0·00 | 0·10 | 0·20 | -7·24 |
| SCAMP3 | 0·00 | 0·10 | 0·21 | -7·25 |
| DKK4 | 0·00 | 0·10 | 0·21 | -7·25 |
| THY1 | 0·00 | 0·10 | 0·21 | -7·25 |
| ING1 | 0·00 | 0·10 | 0·21 | -7·26 |
| LYPD8 | 0·00 | 0·10 | 0·21 | -7·26 |
| HBEGF | -0·00 | 0·10 | 0·21 | -7·26 |
| GPC1 | -0·00 | 0·10 | 0·21 | -7·26 |
| SPINK5 | -0·00 | 0·10 | 0·21 | -7·26 |
| SDC4 | -0·00 | 0·10 | 0·21 | -7·27 |
| CTSB | 0·00 | 0·10 | 0·21 | -7·28 |
| FAP | 0·00 | 0·10 | 0·21 | -7·28 |
| KLB | 0·00 | 0·10 | 0·21 | -7·29 |
| NOTCH1 | -0·00 | 0·10 | 0·22 | -7·30 |
| GUSB | 0·00 | 0·11 | 0·22 | -7·30 |
| CXCL8 | 0·00 | 0·11 | 0·22 | -7·31 |
| PTK7 | 0·00 | 0·11 | 0·22 | -7·32 |
| RBP2 | 0·00 | 0·11 | 0·22 | -7·33 |
| PSME2 | 0·00 | 0·11 | 0·22 | -7·33 |
| AARSD1 | 0·00 | 0·11 | 0·22 | -7·34 |
| SIGLEC6 | -0·00 | 0·11 | 0·22 | -7·34 |
| AMBN | -0·00 | 0·11 | 0·23 | -7·34 |
| OGFR | 0·00 | 0·11 | 0·23 | -7·35 |
| EDAR | -0·00 | 0·11 | 0·23 | -7·37 |
| KITLG | 0·00 | 0·12 | 0·24 | -7·38 |
| FUS | -0·00 | 0·12 | 0·24 | -7·38 |
| SCRN1 | 0·00 | 0·12 | 0·24 | -7·40 |
| MPO | -0·00 | 0·12 | 0·24 | -7·40 |
| CPPED1 | -0·00 | 0·12 | 0·24 | -7·40 |
| NOMO1 | 0·00 | 0·12 | 0·24 | -7·41 |
| EPHA1 | 0·00 | 0·12 | 0·25 | -7·42 |
| CCN2 | 0·00 | 0·12 | 0·25 | -7·43 |
| MED18 | -0·00 | 0·13 | 0·25 | -7·44 |
| CD207 | -0·00 | 0·13 | 0·25 | -7·45 |
| BSG | 0·00 | 0·13 | 0·26 | -7·45 |
| ADA | -0·00 | 0·13 | 0·26 | -7·46 |
| GALNT3 | -0·00 | 0·13 | 0·26 | -7·46 |
| TIMD4 | -0·00 | 0·14 | 0·27 | -7·49 |
| ANXA4 | -0·00 | 0·14 | 0·27 | -7·49 |
| PTPN6 | -0·00 | 0·14 | 0·27 | -7·49 |
| MMP8 | -0·00 | 0·14 | 0·27 | -7·49 |
| HPCAL1 | -0·00 | 0·14 | 0·27 | -7·49 |
| CD274 | 0·00 | 0·14 | 0·27 | -7·50 |
| CDH17 | -0·00 | 0·14 | 0·27 | -7·50 |
| ULBP2 | 0·00 | 0·14 | 0·27 | -7·51 |
| PROC | -0·00 | 0·14 | 0·27 | -7·51 |
| GZMH | 0·00 | 0·14 | 0·27 | -7·51 |
| SFTPA2 | 0·00 | 0·14 | 0·27 | -7·52 |
| ICAM2 | 0·00 | 0·14 | 0·27 | -7·52 |
| ENO1 | -0·00 | 0·14 | 0·28 | -7·53 |
| IL12B | 0·00 | 0·14 | 0·28 | -7·53 |
| VWA1 | 0·00 | 0·15 | 0·28 | -7·54 |
| MUC16 | -0·00 | 0·15 | 0·28 | -7·55 |
| RRM2 | -0·00 | 0·15 | 0·28 | -7·56 |
| IFNGR2 | 0·00 | 0·15 | 0·29 | -7·56 |
| CEACAM8 | -0·00 | 0·15 | 0·29 | -7·56 |
| AKR1C4 | 0·00 | 0·15 | 0·29 | -7·57 |
| OLR1 | -0·00 | 0·15 | 0·29 | -7·58 |
| FGFBP1 | -0·00 | 0·15 | 0·29 | -7·59 |
| SCARF1 | 0·00 | 0·15 | 0·29 | -7·59 |
| C1QTNF1 | 0·00 | 0·15 | 0·29 | -7·59 |
| IL12A_IL12B | 0·00 | 0·16 | 0·30 | -7·60 |
| SCG3 | -0·00 | 0·16 | 0·30 | -7·60 |
| CNTN1 | 0·00 | 0·16 | 0·30 | -7·60 |
| PTH1R | -0·00 | 0·16 | 0·30 | -7·60 |
| MEGF9 | -0·00 | 0·16 | 0·30 | -7·61 |
| AMN | 0·00 | 0·16 | 0·30 | -7·61 |
| IL18R1 | 0·00 | 0·16 | 0·30 | -7·62 |
| CELA3A | 0·00 | 0·16 | 0·30 | -7·62 |
| BST1 | 0·00 | 0·16 | 0·30 | -7·62 |
| SIGLEC9 | 0·00 | 0·16 | 0·30 | -7·63 |
| SEZ6L2 | 0·00 | 0·16 | 0·31 | -7·63 |
| SULT1A1 | -0·00 | 0·16 | 0·31 | -7·63 |
| ECE1 | 0·00 | 0·17 | 0·31 | -7·65 |
| PRL | -0·00 | 0·17 | 0·31 | -7·65 |
| BMP4 | 0·00 | 0·17 | 0·32 | -7·66 |
| MCFD2 | 0·00 | 0·17 | 0·32 | -7·67 |
| IGFBP6 | 0·00 | 0·17 | 0·32 | -7·67 |
| TNFRSF4 | 0·00 | 0·17 | 0·32 | -7·68 |
| FHIT | -0·00 | 0·18 | 0·33 | -7·70 |
| SUMF2 | 0·00 | 0·18 | 0·33 | -7·70 |
| GH1 | 0·01 | 0·18 | 0·33 | -7·70 |
| ICAM1 | -0·00 | 0·18 | 0·33 | -7·70 |
| SLITRK2 | 0·00 | 0·18 | 0·33 | -7·71 |
| SLITRK6 | -0·00 | 0·18 | 0·34 | -7·72 |
| CD300LG | 0·00 | 0·19 | 0·34 | -7·73 |
| GLRX | -0·00 | 0·19 | 0·34 | -7·73 |
| SIGLEC5 | 0·00 | 0·19 | 0·34 | -7·73 |
| CD63 | 0·00 | 0·19 | 0·35 | -7·74 |
| CRLF1 | -0·00 | 0·19 | 0·35 | -7·74 |
| GDF2 | 0·00 | 0·19 | 0·35 | -7·74 |
| DEFA1_DEFA1B | -0·00 | 0·19 | 0·35 | -7·75 |
| GZMB | 0·00 | 0·19 | 0·35 | -7·76 |
| F11R | 0·00 | 0·20 | 0·35 | -7·76 |
| NXPH1 | 0·00 | 0·20 | 0·35 | -7·76 |
| ARHGAP25 | -0·00 | 0·20 | 0·36 | -7·78 |
| CA2 | 0·00 | 0·20 | 0·36 | -7·78 |
| IL34 | 0·00 | 0·20 | 0·36 | -7·78 |
| SF3B4 | -0·00 | 0·20 | 0·36 | -7·78 |
| ASAH2 | 0·00 | 0·20 | 0·36 | -7·78 |
| PRKAR1A | 0·00 | 0·20 | 0·37 | -7·79 |
| CDKN2D | -0·00 | 0·21 | 0·37 | -7·80 |
| FIS1 | 0·00 | 0·21 | 0·37 | -7·80 |
| TGFBI | -0·00 | 0·21 | 0·37 | -7·80 |
| GFRA2 | 0·00 | 0·21 | 0·38 | -7·82 |
| GRN | -0·00 | 0·21 | 0·38 | -7·82 |
| NID2 | 0·00 | 0·21 | 0·38 | -7·82 |
| IPCEF1 | -0·00 | 0·21 | 0·38 | -7·82 |
| SSC4D | -0·00 | 0·22 | 0·39 | -7·84 |
| CD48 | -0·00 | 0·22 | 0·39 | -7·84 |
| LYN | -0·00 | 0·22 | 0·39 | -7·84 |
| FAM3B | 0·00 | 0·22 | 0·39 | -7·85 |
| CCL4 | 0·00 | 0·22 | 0·40 | -7·86 |
| VEGFC | 0·00 | 0·22 | 0·40 | -7·86 |
| SMAD1 | -0·00 | 0·23 | 0·40 | -7·86 |
| GLB1 | 0·00 | 0·23 | 0·40 | -7·87 |
| TPP1 | 0·00 | 0·23 | 0·40 | -7·88 |
| NBN | 0·00 | 0·23 | 0·40 | -7·88 |
| SOD1 | -0·00 | 0·23 | 0·40 | -7·88 |
| SIRT2 | -0·00 | 0·23 | 0·41 | -7·88 |
| SUGT1 | -0·00 | 0·23 | 0·41 | -7·88 |
| FOXO3 | -0·00 | 0·23 | 0·41 | -7·89 |
| IL2RA | -0·00 | 0·24 | 0·41 | -7·89 |
| CNTN4 | 0·00 | 0·24 | 0·42 | -7·90 |
| APBB1IP | -0·00 | 0·24 | 0·42 | -7·92 |
| PTPRN2 | -0·00 | 0·25 | 0·43 | -7·92 |
| AIFM1 | 0·00 | 0·25 | 0·43 | -7·92 |
| LBR | 0·00 | 0·25 | 0·43 | -7·92 |
| ANXA11 | -0·00 | 0·25 | 0·43 | -7·92 |
| PRTG | 0·00 | 0·25 | 0·43 | -7·93 |
| SLIT2 | 0·00 | 0·25 | 0·43 | -7·93 |
| YES1 | -0·00 | 0·25 | 0·43 | -7·93 |
| DCTN1 | 0·00 | 0·25 | 0·43 | -7·93 |
| TPSAB1 | 0·00 | 0·25 | 0·43 | -7·94 |
| SELE | -0·00 | 0·25 | 0·43 | -7·94 |
| CASC4 | 0·00 | 0·25 | 0·43 | -7·94 |
| ADAM23 | 0·00 | 0·25 | 0·43 | -7·94 |
| BANK1 | -0·00 | 0·26 | 0·44 | -7·95 |
| HAGH | 0·00 | 0·26 | 0·44 | -7·95 |
| HYOU1 | 0·00 | 0·26 | 0·44 | -7·95 |
| CC2D1A | 0·00 | 0·26 | 0·44 | -7·95 |
| APEX1 | -0·00 | 0·26 | 0·44 | -7·96 |
| TLR3 | 0·00 | 0·26 | 0·44 | -7·96 |
| CD244 | -0·00 | 0·26 | 0·44 | -7·96 |
| ANKRD54 | -0·00 | 0·26 | 0·44 | -7·96 |
| DNMBP | 0·00 | 0·26 | 0·44 | -7·96 |
| DAPP1 | -0·00 | 0·26 | 0·44 | -7·97 |
| RAB6A | 0·00 | 0·26 | 0·45 | -7·97 |
| FLT4 | 0·00 | 0·27 | 0·45 | -7·97 |
| PSME1 | 0·00 | 0·27 | 0·45 | -7·97 |
| PRDX5 | -0·00 | 0·27 | 0·45 | -7·98 |
| BPIFB1 | 0·00 | 0·27 | 0·46 | -7·99 |
| ESM1 | 0·00 | 0·27 | 0·46 | -7·99 |
| LAT2 | -0·00 | 0·28 | 0·46 | -8·00 |
| DFFA | -0·00 | 0·28 | 0·46 | -8·00 |
| STX4 | 0·00 | 0·28 | 0·46 | -8·00 |
| LAP3 | 0·00 | 0·28 | 0·46 | -8·00 |
| VSIR | 0·00 | 0·28 | 0·46 | -8·00 |
| PCOLCE | 0·00 | 0·28 | 0·46 | -8·01 |
| PSIP1 | 0·00 | 0·28 | 0·46 | -8·01 |
| CXADR | 0·00 | 0·28 | 0·46 | -8·01 |
| CD8A | -0·00 | 0·28 | 0·46 | -8·01 |
| EPHB4 | 0·00 | 0·28 | 0·47 | -8·02 |
| FCRL3 | 0·00 | 0·29 | 0·47 | -8·02 |
| KIFBP | -0·00 | 0·29 | 0·48 | -8·03 |
| PSPN | 0·00 | 0·29 | 0·48 | -8·03 |
| TMPRSS5 | 0·00 | 0·29 | 0·48 | -8·04 |
| ERBB2 | 0·00 | 0·30 | 0·49 | -8·05 |
| MAP2K6 | -0·00 | 0·30 | 0·49 | -8·05 |
| CD28 | 0·00 | 0·30 | 0·49 | -8·05 |
| NCR1 | 0·00 | 0·30 | 0·49 | -8·05 |
| SLC39A14 | 0·00 | 0·30 | 0·49 | -8·06 |
| TMSB10 | 0·00 | 0·30 | 0·49 | -8·06 |
| LAMP2 | 0·00 | 0·30 | 0·49 | -8·06 |
| CCL21 | -0·00 | 0·31 | 0·50 | -8·07 |
| TBL1X | -0·00 | 0·31 | 0·51 | -8·08 |
| GGH | -0·00 | 0·31 | 0·51 | -8·08 |
| POLR2F | 0·00 | 0·31 | 0·51 | -8·08 |
| ARHGAP1 | -0·00 | 0·32 | 0·51 | -8·09 |
| FEN1 | -0·00 | 0·32 | 0·51 | -8·09 |
| VEGFD | 0·00 | 0·32 | 0·51 | -8·09 |
| BID | -0·00 | 0·32 | 0·51 | -8·09 |
| RGMB | 0·00 | 0·32 | 0·51 | -8·10 |
| DBI | 0·00 | 0·32 | 0·51 | -8·10 |
| TNFRSF21 | 0·00 | 0·32 | 0·52 | -8·10 |
| AGRP | -0·00 | 0·32 | 0·52 | -8·10 |
| FUCA1 | 0·00 | 0·32 | 0·52 | -8·10 |
| SLAMF7 | 0·00 | 0·32 | 0·52 | -8·10 |
| HEXIM1 | 0·00 | 0·33 | 0·52 | -8·11 |
| ITGAM | -0·00 | 0·33 | 0·52 | -8·11 |
| PGLYRP1 | 0·00 | 0·33 | 0·52 | -8·11 |
| ICOSLG | -0·00 | 0·33 | 0·52 | -8·12 |
| SH2D1A | 0·00 | 0·33 | 0·52 | -8·12 |
| DDX58 | -0·00 | 0·33 | 0·52 | -8·12 |
| ABL1 | 0·00 | 0·33 | 0·52 | -8·12 |
| SERPINB1 | -0·00 | 0·33 | 0·53 | -8·12 |
| CCL24 | 0·00 | 0·33 | 0·53 | -8·12 |
| LCN2 | -0·00 | 0·33 | 0·53 | -8·12 |
| GCNT1 | 0·00 | 0·34 | 0·53 | -8·13 |
| ERBIN | -0·00 | 0·34 | 0·53 | -8·13 |
| GZMA | -0·00 | 0·34 | 0·53 | -8·13 |
| BAG6 | 0·00 | 0·34 | 0·53 | -8·13 |
| CD38 | 0·00 | 0·34 | 0·53 | -8·13 |
| CDSN | 0·00 | 0·34 | 0·54 | -8·14 |
| IFNLR1 | -0·00 | 0·34 | 0·54 | -8·14 |
| ADAMTS15 | -0·00 | 0·34 | 0·54 | -8·14 |
| STAT5B | -0·00 | 0·35 | 0·54 | -8·14 |
| IL20 | 0·00 | 0·35 | 0·54 | -8·15 |
| NUDT5 | 0·00 | 0·35 | 0·55 | -8·16 |
| FKBP4 | -0·00 | 0·35 | 0·55 | -8·16 |
| NELL1 | 0·00 | 0·35 | 0·55 | -8·16 |
| MSRA | -0·00 | 0·35 | 0·55 | -8·16 |
| TRIAP1 | 0·00 | 0·36 | 0·55 | -8·17 |
| VAT1 | 0·00 | 0·36 | 0·55 | -8·17 |
| CD4 | 0·00 | 0·36 | 0·56 | -8·17 |
| STK11 | 0·00 | 0·36 | 0·56 | -8·17 |
| CCL5 | -0·00 | 0·37 | 0·56 | -8·18 |
| TEK | -0·00 | 0·37 | 0·57 | -8·19 |
| TANK | 0·00 | 0·37 | 0·57 | -8·19 |
| IL20RA | -0·00 | 0·37 | 0·57 | -8·19 |
| C2 | -0·00 | 0·37 | 0·57 | -8·19 |
| CLEC10A | 0·00 | 0·38 | 0·58 | -8·20 |
| LXN | 0·00 | 0·38 | 0·58 | -8·20 |
| COX5B | 0·00 | 0·38 | 0·58 | -8·20 |
| FGR | -0·00 | 0·38 | 0·58 | -8·20 |
| UBAC1 | 0·00 | 0·38 | 0·58 | -8·20 |
| QPCT | 0·00 | 0·38 | 0·58 | -8·20 |
| ALDH3A1 | 0·00 | 0·38 | 0·58 | -8·21 |
| REG3A | 0·00 | 0·38 | 0·58 | -8·21 |
| ICAM3 | 0·00 | 0·38 | 0·58 | -8·21 |
| FUT8 | -0·00 | 0·38 | 0·58 | -8·21 |
| CD200 | 0·00 | 0·39 | 0·59 | -8·21 |
| AIF1 | -0·00 | 0·39 | 0·59 | -8·22 |
| MEP1B | 0·00 | 0·39 | 0·59 | -8·22 |
| CHL1 | -0·00 | 0·39 | 0·59 | -8·22 |
| CASP10 | -0·00 | 0·39 | 0·59 | -8·22 |
| BAX | 0·00 | 0·39 | 0·59 | -8·22 |
| GP6 | -0·00 | 0·39 | 0·59 | -8·22 |
| DCTN2 | 0·00 | 0·39 | 0·59 | -8·22 |
| CEACAM1 | 0·00 | 0·39 | 0·59 | -8·23 |
| FLI1 | -0·00 | 0·40 | 0·59 | -8·23 |
| CANT1 | -0·00 | 0·40 | 0·60 | -8·23 |
| WWP2 | -0·00 | 0·40 | 0·60 | -8·23 |
| IRAK4 | -0·00 | 0·40 | 0·60 | -8·23 |
| CD160 | -0·00 | 0·40 | 0·60 | -8·24 |
| PAG1 | -0·00 | 0·40 | 0·60 | -8·24 |
| PRTFDC1 | -0·00 | 0·40 | 0·60 | -8·24 |
| SRP14 | -0·00 | 0·41 | 0·60 | -8·24 |
| CASP3 | -0·00 | 0·41 | 0·60 | -8·24 |
| AOC3 | 0·00 | 0·41 | 0·60 | -8·24 |
| CD14 | -0·00 | 0·41 | 0·60 | -8·25 |
| PDGFC | -0·00 | 0·41 | 0·60 | -8·25 |
| CHMP1A | -0·00 | 0·41 | 0·61 | -8·25 |
| MICB_MICA | 0·00 | 0·41 | 0·61 | -8·25 |
| PRKRA | -0·00 | 0·41 | 0·61 | -8·25 |
| PEBP1 | 0·00 | 0·41 | 0·61 | -8·25 |
| SHMT1 | 0·00 | 0·41 | 0·61 | -8·25 |
| TDRKH | 0·00 | 0·41 | 0·61 | -8·26 |
| LEPR | -0·00 | 0·42 | 0·61 | -8·26 |
| CLEC4D | -0·00 | 0·42 | 0·61 | -8·26 |
| RP2 | -0·00 | 0·42 | 0·61 | -8·26 |
| SEMA4C | 0·00 | 0·42 | 0·61 | -8·26 |
| RASSF2 | -0·00 | 0·42 | 0·61 | -8·26 |
| GOPC | -0·00 | 0·42 | 0·61 | -8·26 |
| OSMR | -0·00 | 0·42 | 0·62 | -8·27 |
| SIGLEC15 | 0·00 | 0·43 | 0·62 | -8·27 |
| S100A16 | 0·00 | 0·43 | 0·62 | -8·27 |
| ATG4A | -0·00 | 0·43 | 0·62 | -8·27 |
| SPARCL1 | -0·00 | 0·43 | 0·62 | -8·27 |
| TNFRSF13B | 0·00 | 0·43 | 0·62 | -8·27 |
| MIF | -0·00 | 0·43 | 0·62 | -8·27 |
| PQBP1 | 0·00 | 0·43 | 0·62 | -8·28 |
| KAZALD1 | -0·00 | 0·43 | 0·62 | -8·28 |
| PLPBP | -0·00 | 0·43 | 0·63 | -8·28 |
| ITGA11 | -0·00 | 0·43 | 0·63 | -8·28 |
| DAG1 | 0·00 | 0·44 | 0·63 | -8·28 |
| NUB1 | -0·00 | 0·44 | 0·63 | -8·29 |
| PTGDS | 0·00 | 0·44 | 0·64 | -8·29 |
| ALPP | -0·00 | 0·44 | 0·64 | -8·30 |
| CD2AP | 0·00 | 0·45 | 0·64 | -8·30 |
| PSMG3 | -0·00 | 0·45 | 0·65 | -8·31 |
| CTSS | -0·00 | 0·46 | 0·65 | -8·31 |
| IL16 | 0·00 | 0·46 | 0·65 | -8·31 |
| AXIN1 | -0·00 | 0·46 | 0·65 | -8·31 |
| MSMB | 0·00 | 0·46 | 0·66 | -8·32 |
| CD46 | -0·00 | 0·46 | 0·66 | -8·32 |
| NCK2 | -0·00 | 0·46 | 0·66 | -8·32 |
| MASP1 | 0·00 | 0·46 | 0·66 | -8·32 |
| GYS1 | -0·00 | 0·47 | 0·66 | -8·32 |
| TNR | -0·00 | 0·47 | 0·67 | -8·33 |
| FCGR2A | 0·00 | 0·47 | 0·67 | -8·33 |
| ANGPT1 | 0·00 | 0·47 | 0·67 | -8·33 |
| MMP3 | 0·00 | 0·47 | 0·67 | -8·33 |
| TIE1 | 0·00 | 0·47 | 0·67 | -8·33 |
| NDRG1 | 0·00 | 0·47 | 0·67 | -8·33 |
| SPINK6 | 0·00 | 0·48 | 0·67 | -8·33 |
| B4GALT1 | 0·00 | 0·48 | 0·67 | -8·33 |
| ATP5IF1 | 0·00 | 0·48 | 0·67 | -8·33 |
| HPGDS | -0·00 | 0·48 | 0·67 | -8·34 |
| HTRA2 | 0·00 | 0·48 | 0·67 | -8·34 |
| IL18RAP | -0·00 | 0·48 | 0·68 | -8·34 |
| FOLR3 | 0·00 | 0·48 | 0·68 | -8·34 |
| SDC1 | -0·00 | 0·48 | 0·68 | -8·34 |
| PIK3AP1 | -0·00 | 0·49 | 0·68 | -8·35 |
| BST2 | 0·00 | 0·49 | 0·68 | -8·35 |
| EZR | 0·00 | 0·49 | 0·68 | -8·35 |
| TRIM5 | -0·00 | 0·49 | 0·68 | -8·35 |
| MAP4K5 | -0·00 | 0·49 | 0·68 | -8·35 |
| ERP44 | 0·00 | 0·49 | 0·68 | -8·35 |
| FKBP5 | -0·00 | 0·49 | 0·68 | -8·35 |
| CCL26 | -0·00 | 0·49 | 0·68 | -8·35 |
| ADCYAP1R1 | -0·00 | 0·49 | 0·68 | -8·35 |
| AHSP | -0·00 | 0·49 | 0·68 | -8·35 |
| CPVL | 0·00 | 0·50 | 0·69 | -8·36 |
| UXS1 | 0·00 | 0·50 | 0·69 | -8·36 |
| QDPR | 0·00 | 0·50 | 0·69 | -8·36 |
| CPA2 | 0·00 | 0·50 | 0·69 | -8·36 |
| LGALS8 | -0·00 | 0·50 | 0·69 | -8·36 |
| CCL20 | -0·00 | 0·51 | 0·69 | -8·36 |
| ACP6 | -0·00 | 0·51 | 0·69 | -8·37 |
| PXN | -0·00 | 0·51 | 0·70 | -8·37 |
| PODXL | -0·00 | 0·51 | 0·70 | -8·37 |
| IL1R2 | -0·00 | 0·51 | 0·70 | -8·37 |
| TBCC | -0·00 | 0·51 | 0·70 | -8·37 |
| PBLD | -0·00 | 0·52 | 0·70 | -8·38 |
| AGR3 | 0·00 | 0·52 | 0·70 | -8·38 |
| DCTN6 | 0·00 | 0·52 | 0·71 | -8·38 |
| RHOC | 0·00 | 0·52 | 0·71 | -8·38 |
| RBKS | 0·00 | 0·52 | 0·71 | -8·38 |
| CRTAM | 0·00 | 0·52 | 0·71 | -8·38 |
| TJAP1 | 0·00 | 0·52 | 0·71 | -8·38 |
| HMBS | 0·00 | 0·53 | 0·71 | -8·39 |
| SRC | -0·00 | 0·53 | 0·72 | -8·39 |
| RNASE3 | -0·00 | 0·53 | 0·72 | -8·39 |
| XCL1 | 0·00 | 0·54 | 0·73 | -8·40 |
| TXNDC15 | -0·00 | 0·54 | 0·73 | -8·40 |
| LAMA4 | -0·00 | 0·54 | 0·73 | -8·40 |
| PPME1 | -0·00 | 0·54 | 0·73 | -8·40 |
| F9 | 0·00 | 0·54 | 0·73 | -8·40 |
| LACTB2 | 0·00 | 0·54 | 0·73 | -8·40 |
| CTSO | -0·00 | 0·54 | 0·73 | -8·40 |
| RETN | 0·00 | 0·55 | 0·73 | -8·40 |
| PFKFB2 | -0·00 | 0·55 | 0·73 | -8·40 |
| IL18 | -0·00 | 0·55 | 0·73 | -8·40 |
| STC2 | -0·00 | 0·55 | 0·73 | -8·40 |
| OMD | -0·00 | 0·55 | 0·73 | -8·40 |
| FCER2 | 0·00 | 0·55 | 0·73 | -8·41 |
| PMVK | -0·00 | 0·55 | 0·73 | -8·41 |
| DPEP1 | 0·00 | 0·55 | 0·73 | -8·41 |
| FCRL6 | -0·00 | 0·55 | 0·73 | -8·41 |
| PPP1R12A | 0·00 | 0·55 | 0·73 | -8·41 |
| TFRC | 0·00 | 0·55 | 0·73 | -8·41 |
| PARP1 | -0·00 | 0·55 | 0·73 | -8·41 |
| CAPG | -0·00 | 0·55 | 0·73 | -8·41 |
| CD40LG | -0·00 | 0·56 | 0·74 | -8·41 |
| HYAL1 | 0·00 | 0·56 | 0·74 | -8·42 |
| ELOA | -0·00 | 0·56 | 0·74 | -8·42 |
| LPL | 0·00 | 0·56 | 0·74 | -8·42 |
| CEACAM21 | -0·00 | 0·57 | 0·74 | -8·42 |
| IL7 | -0·00 | 0·57 | 0·75 | -8·42 |
| PADI2 | -0·00 | 0·57 | 0·75 | -8·42 |
| FABP6 | 0·00 | 0·57 | 0·75 | -8·42 |
| TNFSF12 | -0·00 | 0·57 | 0·75 | -8·43 |
| RRM2B | 0·00 | 0·57 | 0·75 | -8·43 |
| GALNT2 | -0·00 | 0·57 | 0·75 | -8·43 |
| OXT | 0·00 | 0·58 | 0·75 | -8·43 |
| PTPRM | 0·00 | 0·58 | 0·75 | -8·43 |
| CSF2RA | 0·00 | 0·58 | 0·76 | -8·43 |
| FCAR | 0·00 | 0·58 | 0·76 | -8·43 |
| ANG | 0·00 | 0·58 | 0·76 | -8·43 |
| CRKL | -0·00 | 0·58 | 0·76 | -8·43 |
| SCGB3A2 | -0·00 | 0·58 | 0·76 | -8·44 |
| PDGFB | 0·00 | 0·58 | 0·76 | -8·44 |
| LY9 | 0·00 | 0·58 | 0·76 | -8·44 |
| MGLL | -0·00 | 0·59 | 0·76 | -8·44 |
| IL10RA | -0·00 | 0·59 | 0·77 | -8·44 |
| ITGB2 | 0·00 | 0·59 | 0·77 | -8·44 |
| USP8 | -0·00 | 0·59 | 0·77 | -8·44 |
| PRDX1 | 0·00 | 0·59 | 0·77 | -8·44 |
| TNFSF14 | -0·00 | 0·59 | 0·77 | -8·44 |
| SIRPB1 | 0·00 | 0·60 | 0·77 | -8·44 |
| IL17RB | -0·00 | 0·60 | 0·77 | -8·45 |
| IKBKG | 0·00 | 0·60 | 0·77 | -8·45 |
| DNAJB1 | 0·00 | 0·60 | 0·77 | -8·45 |
| EIF4G1 | 0·00 | 0·60 | 0·77 | -8·45 |
| DLL1 | 0·00 | 0·60 | 0·77 | -8·45 |
| WFDC12 | -0·00 | 0·60 | 0·77 | -8·45 |
| HBQ1 | -0·00 | 0·60 | 0·77 | -8·45 |
| PEAR1 | -0·00 | 0·60 | 0·77 | -8·45 |
| MIA | 0·00 | 0·60 | 0·77 | -8·45 |
| PSMD9 | -0·00 | 0·60 | 0·77 | -8·45 |
| INPPL1 | -0·00 | 0·60 | 0·77 | -8·45 |
| ITM2A | 0·00 | 0·61 | 0·77 | -8·45 |
| CLEC6A | 0·00 | 0·61 | 0·77 | -8·45 |
| SELP | -0·00 | 0·61 | 0·77 | -8·45 |
| PKLR | 0·00 | 0·61 | 0·77 | -8·45 |
| CKMT1A_CKMT1B | -0·00 | 0·61 | 0·77 | -8·45 |
| FXN | 0·00 | 0·61 | 0·78 | -8·46 |
| STK4 | -0·00 | 0·61 | 0·78 | -8·46 |
| TACC3 | -0·00 | 0·62 | 0·78 | -8·46 |
| CD58 | -0·00 | 0·62 | 0·78 | -8·46 |
| PTPRF | 0·00 | 0·62 | 0·78 | -8·46 |
| NUDC | -0·00 | 0·62 | 0·78 | -8·46 |
| IGFBP1 | 0·00 | 0·62 | 0·78 | -8·46 |
| EREG | 0·00 | 0·62 | 0·78 | -8·46 |
| IL1RN | -0·00 | 0·62 | 0·78 | -8·46 |
| NAMPT | -0·00 | 0·62 | 0·78 | -8·46 |
| LAT | -0·00 | 0·62 | 0·78 | -8·46 |
| MITD1 | -0·00 | 0·62 | 0·78 | -8·47 |
| USO1 | -0·00 | 0·63 | 0·78 | -8·47 |
| CNST | 0·00 | 0·63 | 0·78 | -8·47 |
| DIABLO | 0·00 | 0·63 | 0·78 | -8·47 |
| RNF41 | -0·00 | 0·63 | 0·78 | -8·47 |
| ITGB1BP2 | -0·00 | 0·63 | 0·78 | -8·47 |
| KLK6 | 0·00 | 0·63 | 0·79 | -8·47 |
| IMPA1 | -0·00 | 0·63 | 0·79 | -8·47 |
| IFNG | 0·00 | 0·63 | 0·79 | -8·47 |
| MPI | 0·00 | 0·64 | 0·79 | -8·47 |
| PDLIM7 | 0·00 | 0·64 | 0·79 | -8·47 |
| OSM | -0·00 | 0·64 | 0·79 | -8·47 |
| CCL22 | 0·00 | 0·64 | 0·79 | -8·48 |
| IVD | 0·00 | 0·64 | 0·79 | -8·48 |
| BLMH | -0·00 | 0·64 | 0·80 | -8·48 |
| MGMT | -0·00 | 0·65 | 0·80 | -8·48 |
| ENTPD6 | 0·00 | 0·65 | 0·80 | -8·48 |
| PTX3 | 0·00 | 0·65 | 0·80 | -8·48 |
| GAS6 | -0·00 | 0·65 | 0·80 | -8·48 |
| CRHBP | 0·00 | 0·65 | 0·80 | -8·48 |
| SERPINB5 | -0·00 | 0·65 | 0·80 | -8·48 |
| THPO | -0·00 | 0·65 | 0·80 | -8·48 |
| TXLNA | 0·00 | 0·65 | 0·80 | -8·48 |
| F2R | 0·00 | 0·65 | 0·80 | -8·48 |
| LHPP | 0·00 | 0·65 | 0·80 | -8·48 |
| NPTN | -0·00 | 0·66 | 0·80 | -8·48 |
| RAD23B | -0·00 | 0·66 | 0·80 | -8·49 |
| CD83 | -0·00 | 0·66 | 0·80 | -8·49 |
| TBCB | -0·00 | 0·66 | 0·80 | -8·49 |
| TCL1A | 0·00 | 0·66 | 0·80 | -8·49 |
| SCARB1 | 0·00 | 0·66 | 0·80 | -8·49 |
| NPM1 | -0·00 | 0·66 | 0·80 | -8·49 |
| CIAPIN1 | 0·00 | 0·66 | 0·80 | -8·49 |
| TRIM21 | 0·00 | 0·66 | 0·81 | -8·49 |
| NRCAM | 0·00 | 0·67 | 0·81 | -8·49 |
| CXCL3 | -0·00 | 0·67 | 0·81 | -8·49 |
| RUVBL1 | -0·00 | 0·67 | 0·82 | -8·49 |
| REG1A | 0·00 | 0·67 | 0·82 | -8·50 |
| TFPI | -0·00 | 0·68 | 0·82 | -8·50 |
| PTPN1 | 0·00 | 0·68 | 0·82 | -8·50 |
| FADD | -0·00 | 0·68 | 0·82 | -8·50 |
| CDH1 | -0·00 | 0·68 | 0·82 | -8·50 |
| MPIG6B | -0·00 | 0·68 | 0·82 | -8·50 |
| OMG | 0·00 | 0·68 | 0·82 | -8·50 |
| CXCL6 | -0·00 | 0·68 | 0·82 | -8·50 |
| STX6 | 0·00 | 0·69 | 0·82 | -8·50 |
| CASP8 | 0·00 | 0·69 | 0·83 | -8·50 |
| EIF4EBP1 | 0·00 | 0·69 | 0·83 | -8·50 |
| PPP1R2 | 0·00 | 0·69 | 0·83 | -8·51 |
| BLVRB | 0·00 | 0·69 | 0·83 | -8·51 |
| MAVS | -0·00 | 0·70 | 0·83 | -8·51 |
| NFKBIE | -0·00 | 0·70 | 0·83 | -8·51 |
| CD55 | 0·00 | 0·70 | 0·83 | -8·51 |
| PPIB | 0·00 | 0·70 | 0·84 | -8·51 |
| ENO2 | 0·00 | 0·70 | 0·84 | -8·51 |
| GLO1 | -0·00 | 0·71 | 0·84 | -8·51 |
| SNAP23 | -0·00 | 0·71 | 0·84 | -8·51 |
| BACH1 | -0·00 | 0·71 | 0·84 | -8·51 |
| CLEC4G | -0·00 | 0·71 | 0·84 | -8·51 |
| MMP10 | -0·00 | 0·71 | 0·84 | -8·52 |
| GFOD2 | -0·00 | 0·71 | 0·84 | -8·52 |
| STX8 | 0·00 | 0·72 | 0·86 | -8·52 |
| INPP1 | -0·00 | 0·72 | 0·86 | -8·52 |
| CBLN4 | -0·00 | 0·73 | 0·86 | -8·52 |
| CA11 | 0·00 | 0·73 | 0·86 | -8·52 |
| C2CD2L | 0·00 | 0·73 | 0·86 | -8·52 |
| CAMKK1 | 0·00 | 0·73 | 0·86 | -8·52 |
| NFATC1 | -0·00 | 0·73 | 0·86 | -8·52 |
| TIA1 | -0·00 | 0·73 | 0·86 | -8·53 |
| CD209 | 0·00 | 0·73 | 0·86 | -8·53 |
| MANF | 0·00 | 0·73 | 0·86 | -8·53 |
| ENPP2 | 0·00 | 0·73 | 0·86 | -8·53 |
| CLSTN1 | 0·00 | 0·74 | 0·86 | -8·53 |
| GPC5 | -0·00 | 0·74 | 0·86 | -8·53 |
| AHCY | 0·00 | 0·74 | 0·86 | -8·53 |
| GRAP2 | -0·00 | 0·74 | 0·86 | -8·53 |
| THBD | -0·00 | 0·74 | 0·86 | -8·53 |
| KYAT1 | -0·00 | 0·74 | 0·86 | -8·53 |
| DSG2 | -0·00 | 0·74 | 0·86 | -8·53 |
| ANGPTL3 | 0·00 | 0·74 | 0·86 | -8·53 |
| AKT1S1 | 0·00 | 0·74 | 0·86 | -8·53 |
| ADAM15 | 0·00 | 0·74 | 0·86 | -8·53 |
| HS6ST1 | -0·00 | 0·74 | 0·86 | -8·53 |
| LY75 | -0·00 | 0·74 | 0·86 | -8·53 |
| WFIKKN2 | 0·00 | 0·75 | 0·86 | -8·53 |
| TGFA | 0·00 | 0·75 | 0·87 | -8·53 |
| CA1 | -0·00 | 0·75 | 0·87 | -8·53 |
| KLK10 | -0·00 | 0·75 | 0·87 | -8·53 |
| PLA2G1B | -0·00 | 0·75 | 0·87 | -8·53 |
| ARHGEF12 | 0·00 | 0·75 | 0·87 | -8·53 |
| PDGFA | 0·00 | 0·76 | 0·87 | -8·54 |
| PPCDC | 0·00 | 0·76 | 0·88 | -8·54 |
| PDCD1LG2 | -0·00 | 0·76 | 0·88 | -8·54 |
| CLIP2 | -0·00 | 0·76 | 0·88 | -8·54 |
| DDC | -0·00 | 0·76 | 0·88 | -8·54 |
| FCRL2 | -0·00 | 0·76 | 0·88 | -8·54 |
| NELL2 | -0·00 | 0·76 | 0·88 | -8·54 |
| ZBTB16 | -0·00 | 0·77 | 0·88 | -8·54 |
| LRMP | 0·00 | 0·77 | 0·88 | -8·54 |
| PON2 | -0·00 | 0·77 | 0·88 | -8·54 |
| TGM2 | 0·00 | 0·77 | 0·88 | -8·54 |
| ICAM4 | 0·00 | 0·77 | 0·88 | -8·54 |
| SERPINA12 | -0·00 | 0·77 | 0·88 | -8·54 |
| PPP1R9B | 0·00 | 0·78 | 0·88 | -8·54 |
| HSPB1 | 0·00 | 0·78 | 0·89 | -8·54 |
| GFER | -0·00 | 0·78 | 0·89 | -8·54 |
| SCLY | 0·00 | 0·78 | 0·89 | -8·55 |
| PNLIPRP2 | 0·00 | 0·78 | 0·89 | -8·55 |
| SOD2 | -0·00 | 0·79 | 0·89 | -8·55 |
| PLA2G7 | -0·00 | 0·79 | 0·89 | -8·55 |
| TBC1D5 | 0·00 | 0·79 | 0·90 | -8·55 |
| NID1 | -0·00 | 0·79 | 0·90 | -8·55 |
| KRT5 | 0·00 | 0·80 | 0·90 | -8·55 |
| NRP1 | -0·00 | 0·80 | 0·90 | -8·55 |
| AKR1B1 | 0·00 | 0·80 | 0·91 | -8·55 |
| CLEC1A | -0·00 | 0·80 | 0·91 | -8·55 |
| CDC37 | -0·00 | 0·80 | 0·91 | -8·55 |
| MATN2 | 0·00 | 0·80 | 0·91 | -8·55 |
| GMPR | -0·00 | 0·80 | 0·91 | -8·55 |
| METAP2 | -0·00 | 0·81 | 0·91 | -8·55 |
| F7 | 0·00 | 0·81 | 0·91 | -8·55 |
| ATXN10 | 0·00 | 0·81 | 0·91 | -8·55 |
| CSF3 | 0·00 | 0·81 | 0·91 | -8·56 |
| EGF | -0·00 | 0·82 | 0·91 | -8·56 |
| MMP9 | 0·00 | 0·82 | 0·91 | -8·56 |
| SUSD1 | -0·00 | 0·82 | 0·91 | -8·56 |
| TARBP2 | 0·00 | 0·82 | 0·91 | -8·56 |
| CA13 | -0·00 | 0·82 | 0·91 | -8·56 |
| SIRPA | 0·00 | 0·82 | 0·91 | -8·56 |
| OSCAR | -0·00 | 0·82 | 0·92 | -8·56 |
| KLK14 | 0·00 | 0·82 | 0·92 | -8·56 |
| CCS | -0·00 | 0·83 | 0·92 | -8·56 |
| APRT | 0·00 | 0·83 | 0·92 | -8·56 |
| FABP5 | -0·00 | 0·83 | 0·92 | -8·56 |
| CXCL12 | -0·00 | 0·83 | 0·92 | -8·56 |
| PDCD5 | 0·00 | 0·83 | 0·92 | -8·56 |
| ITGB1 | -0·00 | 0·83 | 0·92 | -8·56 |
| PLA2G4A | -0·00 | 0·83 | 0·92 | -8·56 |
| ILKAP | 0·00 | 0·83 | 0·92 | -8·56 |
| VPS37A | -0·00 | 0·83 | 0·92 | -8·56 |
| CD177 | 0·00 | 0·84 | 0·92 | -8·56 |
| TBC1D23 | 0·00 | 0·84 | 0·92 | -8·56 |
| ICA1 | -0·00 | 0·84 | 0·93 | -8·56 |
| SELPLG | -0·00 | 0·84 | 0·93 | -8·56 |
| DNAJA2 | -0·00 | 0·84 | 0·93 | -8·56 |
| RWDD1 | 0·00 | 0·84 | 0·93 | -8·56 |
| SKAP2 | -0·00 | 0·85 | 0·93 | -8·56 |
| AK1 | 0·00 | 0·85 | 0·93 | -8·56 |
| BTC | -0·00 | 0·85 | 0·93 | -8·57 |
| CALCOCO1 | -0·00 | 0·85 | 0·93 | -8·57 |
| EGLN1 | -0·00 | 0·85 | 0·93 | -8·57 |
| PRDX6 | 0·00 | 0·85 | 0·93 | -8·57 |
| LBP | -0·00 | 0·85 | 0·93 | -8·57 |
| CORO1A | 0·00 | 0·85 | 0·93 | -8·57 |
| ITGB7 | -0·00 | 0·85 | 0·93 | -8·57 |
| TYMP | 0·00 | 0·86 | 0·93 | -8·57 |
| CTRC | 0·00 | 0·86 | 0·93 | -8·57 |
| LAIR2 | -0·00 | 0·86 | 0·94 | -8·57 |
| IL13RA1 | -0·00 | 0·86 | 0·94 | -8·57 |
| PVALB | 0·00 | 0·86 | 0·94 | -8·57 |
| S100P | 0·00 | 0·86 | 0·94 | -8·57 |
| TPMT | -0·00 | 0·86 | 0·94 | -8·57 |
| DAB2 | -0·00 | 0·86 | 0·94 | -8·57 |
| GLOD4 | 0·00 | 0·86 | 0·94 | -8·57 |
| CNPY4 | 0·00 | 0·86 | 0·94 | -8·57 |
| SPARC | 0·00 | 0·87 | 0·94 | -8·57 |
| CD300LF | 0·00 | 0·87 | 0·94 | -8·57 |
| PLXNB3 | -0·00 | 0·87 | 0·94 | -8·57 |
| FOPNL | 0·00 | 0·87 | 0·94 | -8·57 |
| EPHB6 | -0·00 | 0·87 | 0·94 | -8·57 |
| CDC27 | 0·00 | 0·87 | 0·94 | -8·57 |
| LHB | -0·00 | 0·87 | 0·94 | -8·57 |
| ARSB | 0·00 | 0·87 | 0·94 | -8·57 |
| NSFL1C | 0·00 | 0·88 | 0·94 | -8·57 |
| CASP2 | -0·00 | 0·88 | 0·94 | -8·57 |
| CXCL1 | 0·00 | 0·88 | 0·94 | -8·57 |
| HARS | -0·00 | 0·88 | 0·94 | -8·57 |
| CLEC1B | 0·00 | 0·88 | 0·94 | -8·57 |
| VCAM1 | -0·00 | 0·88 | 0·94 | -8·57 |
| DNPH1 | -0·00 | 0·88 | 0·94 | -8·57 |
| GSAP | -0·00 | 0·88 | 0·94 | -8·57 |
| BCR | -0·00 | 0·89 | 0·95 | -8·57 |
| STAMBP | -0·00 | 0·89 | 0·95 | -8·57 |
| KLRB1 | 0·00 | 0·89 | 0·95 | -8·57 |
| PRDX3 | 0·00 | 0·89 | 0·95 | -8·57 |
| TDGF1 | -0·00 | 0·89 | 0·95 | -8·57 |
| PLXNA4 | -0·00 | 0·89 | 0·95 | -8·57 |
| CARHSP1 | 0·00 | 0·90 | 0·95 | -8·57 |
| METAP1D | -0·00 | 0·90 | 0·95 | -8·57 |
| SERPINB6 | 0·00 | 0·90 | 0·95 | -8·57 |
| GKN1 | -0·00 | 0·90 | 0·95 | -8·57 |
| IL17RA | 0·00 | 0·90 | 0·96 | -8·57 |
| ICAM5 | 0·00 | 0·90 | 0·96 | -8·57 |
| NDUFS6 | 0·00 | 0·90 | 0·96 | -8·57 |
| EIF4B | -0·00 | 0·91 | 0·96 | -8·58 |
| DBNL | -0·00 | 0·91 | 0·96 | -8·58 |
| FCRL5 | -0·00 | 0·91 | 0·96 | -8·58 |
| COMT | -0·00 | 0·92 | 0·96 | -8·58 |
| MAX | 0·00 | 0·92 | 0·96 | -8·58 |
| CES3 | -0·00 | 0·92 | 0·96 | -8·58 |
| IRAK1 | 0·00 | 0·92 | 0·97 | -8·58 |
| HSPA1A | -0·00 | 0·92 | 0·97 | -8·58 |
| FCGR2B | -0·00 | 0·92 | 0·97 | -8·58 |
| PARK7 | 0·00 | 0·92 | 0·97 | -8·58 |
| EBAG9 | 0·00 | 0·93 | 0·97 | -8·58 |
| XPNPEP2 | -0·00 | 0·93 | 0·97 | -8·58 |
| BAG3 | -0·00 | 0·93 | 0·97 | -8·58 |
| DRG2 | -0·00 | 0·93 | 0·97 | -8·58 |
| FYB1 | -0·00 | 0·93 | 0·97 | -8·58 |
| ATOX1 | -0·00 | 0·93 | 0·97 | -8·58 |
| RABGAP1L | 0·00 | 0·93 | 0·97 | -8·58 |
| CLEC5A | -0·00 | 0·93 | 0·97 | -8·58 |
| YTHDF3 | -0·00 | 0·93 | 0·97 | -8·58 |
| SERPINB8 | 0·00 | 0·94 | 0·97 | -8·58 |
| FABP2 | 0·00 | 0·94 | 0·97 | -8·58 |
| MESD | -0·00 | 0·94 | 0·98 | -8·58 |
| SNX9 | -0·00 | 0·94 | 0·98 | -8·58 |
| SLAMF6 | -8·78e-05 | 0·94 | 0·98 | -8·58 |
| SPP1 | 0·00 | 0·94 | 0·98 | -8·58 |
| MYO9B | 0·00 | 0·94 | 0·98 | -8·58 |
| CRELD2 | 0·00 | 0·95 | 0·98 | -8·58 |
| RILP | 0·00 | 0·95 | 0·98 | -8·58 |
| DECR1 | -0·00 | 0·95 | 0·98 | -8·58 |
| KIR2DL3 | -0·00 | 0·95 | 0·98 | -8·58 |
| PLIN3 | 0·00 | 0·95 | 0·98 | -8·58 |
| VNN2 | 0·00 | 0·95 | 0·98 | -8·58 |
| FXYD5 | -0·00 | 0·95 | 0·98 | -8·58 |
| HSD11B1 | 8·99e-05 | 0·95 | 0·98 | -8·58 |
| NT5C3A | 0·00 | 0·96 | 0·98 | -8·58 |
| TST | 0·00 | 0·96 | 0·98 | -8·58 |
| PDCD6 | -0·00 | 0·96 | 0·98 | -8·58 |
| LSP1 | -0·00 | 0·96 | 0·98 | -8·58 |
| CCL19 | -0·00 | 0·96 | 0·98 | -8·58 |
| NADK | -0·00 | 0·96 | 0·98 | -8·58 |
| DPEP2 | -4·38e-05 | 0·96 | 0·98 | -8·58 |
| SNAP29 | 0·00 | 0·96 | 0·98 | -8·58 |
| SAMD9L | -0·00 | 0·96 | 0·98 | -8·58 |
| IL17C | 0·00 | 0·96 | 0·98 | -8·58 |
| CRADD | 0·00 | 0·96 | 0·98 | -8·58 |
| CD69 | -0·00 | 0·97 | 0·98 | -8·58 |
| IDS | -2·25e-05 | 0·97 | 0·98 | -8·58 |
| STIP1 | -9·33e-05 | 0·97 | 0·98 | -8·58 |
| HDGF | 0·00 | 0·97 | 0·98 | -8·58 |
| TRAF2 | -9·31e-05 | 0·97 | 0·98 | -8·58 |
| SEZ6L | 4·78e-05 | 0·97 | 0·98 | -8·58 |
| AMIGO2 | -3·51e-05 | 0·97 | 0·98 | -8·58 |
| CRISP2 | 6·67e-05 | 0·97 | 0·98 | -8·58 |
| CD33 | 0·00 | 0·97 | 0·98 | -8·58 |
| IL18BP | 5·48e-05 | 0·97 | 0·98 | -8·58 |
| SPRY2 | -9·56e-05 | 0·97 | 0·98 | -8·58 |
| CDKN1A | -0·00 | 0·98 | 0·99 | -8·58 |
| JUN | -4·45e-05 | 0·98 | 0·99 | -8·58 |
| DARS | -6·01e-05 | 0·98 | 0·99 | -8·58 |
| HNRNPK | -5·97e-05 | 0·98 | 0·99 | -8·58 |
| B4GAT1 | 1·99e-05 | 0·98 | 0·99 | -8·58 |
| TNFRSF9 | -2·65e-05 | 0·98 | 0·99 | -8·58 |
| SPINT1 | 1·66e-05 | 0·98 | 0·99 | -8·58 |
| DOK2 | -6·59e-05 | 0·98 | 0·99 | -8·58 |
| CRACR2A | -5·35e-05 | 0·98 | 0·99 | -8·58 |
| NECTIN4 | -1·75e-05 | 0·99 | 0·99 | -8·58 |
| CXCL5 | -4·44e-05 | 0·99 | 0·99 | -8·58 |
| PCDH17 | 1·82e-05 | 0·99 | 0·99 | -8·58 |
| VPS53 | 1·81e-05 | 0·99 | 0·99 | -8·58 |
| SERPINE1 | -4·65e-06 | 0·99 | 0·99 | -8·58 |

## Supplementary Table 6. Meta-analysis results of proteins consistently associated with chronological age (77 proteins) using PLHIV (200HIV and 2000HIV cohort) and healthy controls (200FG cohort)

| Protein | log FC 2000HIV | p-value 2000HIV | FDR 2000HIV | log FC 200FG | p-value 200FG | FDR 200FG | log FC 200HIV | p-value 200HIV | FDR 200HIV | p-random model |
| --- | --- | --- | --- | --- | --- | --- | --- | --- | --- | --- |
| ADAMTS16 | 0·01 | 6·54e-24 | 4·49e-22 | 0·00 | 0·00 | 0·01 | 0·01 | 2·42e-07 | 8·95e-06 | 1·04e-12 |
| ADGRG1 | 0·03 | 4·48e-24 | 3·44e-22 | 0·03 | 5·54e-08 | 8·03e-06 | 0·02 | 2·87e-06 | 6·19e-05 | 2·34e-38 |
| ADM | 0·01 | 8·84e-19 | 3·39e-17 | 0·01 | 4·06e-05 | 0·00 | 0·01 | 1·61e-09 | 1·3e-07 | 4·54e-26 |
| AMBP | 0·00 | 1·70e-13 | 4·03e-12 | 0·00 | 0·00 | 0·00 | 0·00 | 3·23e-05 | 0·00 | 2·73e-21 |
| BOC | -0·00 | 0·00 | 0·00 | -0·00 | 0·00 | 0·02 | -0·00 | 0·00 | 0·00 | 2·65e-09 |
| CCL11 | 0·00 | 1·16e-11 | 2·23e-10 | 0·01 | 0·00 | 0·01 | 0·01 | 2·26e-06 | 5·04e-05 | 2·03e-19 |
| CCL13 | 0·01 | 1·36e-10 | 2·23e-09 | 0·02 | 0·00 | 0·01 | 0·01 | 1·3e-05 | 0·00 | 7·03e-18 |
| CCL2 | 0·00 | 0·00 | 0·02 | 0·01 | 0·00 | 0·01 | 0·00 | 0·00 | 0·00 | 1·86e-05 |
| CCL3 | 0·00 | 0·00 | 0·00 | 0·01 | 4·67e-06 | 0·00 | 0·01 | 3·64e-05 | 0·00 | 5·19e-08 |
| CD300E | 0·00 | 6·51e-08 | 7·03e-07 | 0·01 | 0·00 | 0·01 | 0·01 | 0·00 | 0·00 | 2·26e-13 |
| CD74 | 0·00 | 8·25e-06 | 5·95e-05 | 0·00 | 0·00 | 0·03 | 0·00 | 0·00 | 0·00 | 1·25e-10 |
| CDCP1 | 0·02 | 4·29e-25 | 3·73e-23 | 0·02 | 5·51e-06 | 0·00 | 0·02 | 4·98e-08 | 2·53e-06 | 4·27e-39 |
| CDON | -0·01 | 5·22e-14 | 1·31e-12 | -0·01 | 8·9e-06 | 0·00 | -0·01 | 1·16e-05 | 0·00 | 1·64e-23 |
| CGA | 0·02 | 1·76e-19 | 7·68e-18 | 0·02 | 0·00 | 0·01 | 0·01 | 5·04e-05 | 0·00 | 8·67e-27 |
| COL1A1 | -0·00 | 4·77e-10 | 7·25e-09 | -0·00 | 0·00 | 0·01 | -0·00 | 0·00 | 0·03 | 1·53e-14 |
| CRH | -0·01 | 2·02e-07 | 1·99e-06 | -0·03 | 2·22e-05 | 0·00 | -0·02 | 0·00 | 0·00 | 8·30e-08 |
| CTSV | -0·01 | 1·27e-12 | 2·70e-11 | -0·01 | 1·71e-05 | 0·00 | -0·01 | 0·00 | 0·00 | 2·58e-16 |
| CXCL10 | 0·01 | 2·80e-07 | 2·61e-06 | 0·02 | 6·11e-06 | 0·00 | 0·01 | 0·00 | 0·03 | 0·00 |
| CXCL14 | 0·02 | 1·42e-31 | 1·85e-29 | 0·03 | 2·23e-07 | 2·24e-05 | 0·02 | 4·09e-07 | 1·39e-05 | 9·62e-48 |
| CXCL17 | 0·02 | 6·33e-24 | 4·49e-22 | 0·03 | 3·09e-10 | 1·01e-07 | 0·02 | 2·9e-05 | 0·00 | 2·60e-36 |
| CXCL9 | 0·01 | 5·94e-09 | 7·46e-08 | 0·02 | 0·00 | 0·02 | 0·01 | 0·00 | 0·00 | 2·53e-14 |
| DCN | 0·00 | 1·23e-15 | 3·67e-14 | 0·00 | 0·00 | 0·03 | 0·00 | 0·00 | 0·00 | 1·09e-20 |
| EBI3_IL27 | 0·00 | 7·75e-11 | 1·33e-09 | 0·00 | 0·00 | 0·03 | 0·00 | 0·00 | 0·00 | 4·33e-16 |
| EDA2R | 0·03 | 5·48e-51 | 3·58e-48 | 0·02 | 4·83e-12 | 3·15e-09 | 0·03 | 3·68e-26 | 4·75e-23 | 2·36e-69 |
| EFEMP1 | 0·01 | 1·24e-08 | 1·45e-07 | 0·01 | 0·00 | 0·01 | 0·01 | 9·31e-09 | 6·34e-07 | 1·95e-19 |
| EFNA1 | 0·00 | 0·00 | 0·00 | 0·00 | 0·00 | 0·03 | 0·00 | 0·00 | 0·00 | 2·79e-06 |
| FAS | 0·00 | 1·28e-05 | 8·90e-05 | 0·00 | 0·00 | 0·00 | 0·00 | 0·00 | 0·00 | 6·38e-09 |
| FGF5 | 0·01 | 9·99e-14 | 2·41e-12 | 0·01 | 5·65e-06 | 0·00 | 0·01 | 4·37e-06 | 8·81e-05 | 5·21e-24 |
| FLT3LG | 0·01 | 5·48e-19 | 2·17e-17 | 0·01 | 4·04e-05 | 0·00 | 0·01 | 1·32e-06 | 3·42e-05 | 1·77e-29 |
| FSTL3 | 0·01 | 2·08e-12 | 4·32e-11 | 0·01 | 0·00 | 0·02 | 0·01 | 4·91e-06 | 9·48e-05 | 5·75e-20 |
| FUT3_FUT5 | 0·00 | 7·66e-06 | 5·55e-05 | 0·01 | 0·00 | 0·04 | 0·01 | 0·00 | 0·00 | 1·05e-10 |
| GDF15 | 0·03 | 1·38e-36 | 3·00e-34 | 0·02 | 1·84e-07 | 2·19e-05 | 0·03 | 2·35e-15 | 6·7e-13 | 2·54e-45 |
| GFAP | 0·02 | 5·49e-32 | 7·98e-30 | 0·02 | 7·02e-09 | 1·31e-06 | 0·02 | 1·76e-12 | 2·53e-10 | 1·74e-56 |
| HAVCR1 | 0·03 | 1·04e-30 | 1·24e-28 | 0·03 | 1·16e-09 | 2·54e-07 | 0·03 | 1·84e-10 | 1·98e-08 | 1·80e-53 |
| HAVCR2 | 0·00 | 2·72e-07 | 2·55e-06 | 0·00 | 0·00 | 0·03 | 0·01 | 1·7e-05 | 0·00 | 5·64e-13 |
| HGF | 0·00 | 3·93e-05 | 0·00 | 0·00 | 0·00 | 0·03 | 0·01 | 1·37e-07 | 5·58e-06 | 2·87e-05 |
| HSPB6 | 0·02 | 1·48e-22 | 9·69e-21 | 0·01 | 0·00 | 0·01 | 0·01 | 3·25e-08 | 1·75e-06 | 6·35e-16 |
| IGFBP4 | 0·01 | 1·27e-15 | 3·71e-14 | 0·01 | 0·00 | 0·00 | 0·01 | 1·33e-08 | 8·21e-07 | 5·69e-27 |
| IGFBPL1 | 0·01 | 5·06e-29 | 5·51e-27 | 0·01 | 3·62e-05 | 0·00 | 0·01 | 5·49e-08 | 2·63e-06 | 5·66e-19 |
| IL17D | 0·01 | 9·91e-34 | 1·61e-31 | 0·00 | 0·00 | 0·02 | 0·01 | 0·00 | 0·00 | 2·67e-06 |
| IL6 | 0·02 | 8·69e-10 | 1·27e-08 | 0·04 | 2·77e-07 | 2·55e-05 | 0·02 | 3·47e-06 | 7·23e-05 | 5·79e-07 |
| KIT | -0·01 | 1·69e-11 | 3·20e-10 | -0·00 | 0·00 | 0·03 | -0·01 | 4·63e-07 | 1·5e-05 | 1·19e-19 |
| KLK4 | 0·03 | 4·78e-37 | 1·25e-34 | 0·02 | 3·69e-07 | 3·01e-05 | 0·04 | 2·59e-15 | 6·7e-13 | 2·19e-60 |
| LAIR1 | 0·00 | 1·92e-05 | 0·00 | 0·01 | 0·00 | 0·03 | 0·00 | 0·00 | 0·00 | 6·61e-10 |
| LGALS9 | 0·00 | 1·22e-06 | 9·83e-06 | 0·01 | 0·00 | 0·01 | 0·00 | 4·58e-05 | 0·00 | 4·64e-11 |
| LTBP2 | 0·02 | 1·76e-53 | 2·30e-50 | 0·01 | 6·85e-08 | 8·95e-06 | 0·02 | 1·58e-17 | 1·02e-14 | 3·19e-29 |
| MLN | 0·03 | 4·79e-18 | 1·64e-16 | 0·03 | 4·73e-07 | 3·64e-05 | 0·02 | 6·62e-06 | 0·00 | 9·55e-30 |
| MOG | 0·00 | 1·35e-07 | 1·37e-06 | 0·01 | 0·00 | 0·01 | 0·01 | 1·61e-06 | 3·85e-05 | 7·85e-09 |
| MSR1 | 0·01 | 1·23e-21 | 6·70e-20 | 0·01 | 0·00 | 0·01 | 0·02 | 1·36e-06 | 3·44e-05 | 6·09e-31 |
| NEFL | 0·03 | 6·74e-44 | 2·20e-41 | 0·02 | 1·5e-14 | 1·96e-11 | 0·02 | 3·65e-16 | 1·57e-13 | 1·09e-85 |
| NFASC | 0·00 | 7·64e-14 | 1·88e-12 | 0·00 | 0·00 | 0·01 | 0·01 | 1·1e-07 | 4·93e-06 | 1·80e-23 |
| NTproBNP | 0·03 | 4·13e-17 | 1·31e-15 | 0·02 | 0·00 | 0·01 | 0·04 | 1·44e-07 | 5·64e-06 | 4·62e-26 |
| OGN | 0·01 | 5·49e-18 | 1·84e-16 | 0·01 | 0·00 | 0·00 | 0·02 | 8·15e-12 | 1·05e-09 | 5·80e-13 |
| PGF | 0·00 | 5·71e-16 | 1·73e-14 | 0·00 | 0·00 | 0·03 | 0·01 | 2·35e-08 | 1·32e-06 | 6·19e-26 |
| PIK3IP1 | 0·00 | 1·65e-09 | 2·34e-08 | 0·00 | 0·00 | 0·02 | 0·01 | 1·38e-07 | 5·58e-06 | 2·14e-10 |
| PODXL2 | -0·00 | 4·30e-07 | 3·79e-06 | -0·01 | 0·00 | 0·01 | -0·00 | 5·44e-05 | 0·00 | 7·29e-13 |
| PROK1 | -0·01 | 2·17e-07 | 2·09e-06 | -0·01 | 0·00 | 0·01 | -0·01 | 0·00 | 0·00 | 6·38e-13 |
| RET | -0·01 | 1·03e-11 | 2·01e-10 | -0·01 | 2·93e-07 | 2·55e-05 | -0·01 | 0·00 | 0·00 | 4·39e-10 |
| RSPO3 | 0·01 | 1·16e-27 | 1·17e-25 | 0·00 | 0·00 | 0·04 | 0·01 | 5·14e-10 | 4·75e-08 | 3·17e-08 |
| SCARB2 | 0·01 | 2·45e-19 | 1·00e-17 | 0·00 | 0·00 | 0·01 | 0·01 | 1·22e-07 | 5·25e-06 | 1·73e-29 |
| SCARF2 | 0·01 | 9·91e-36 | 1·84e-33 | 0·00 | 0·00 | 0·00 | 0·01 | 2·33e-13 | 4·3e-11 | 1·65e-11 |
| SMOC1 | 0·01 | 1·26e-16 | 3·93e-15 | 0·01 | 0·00 | 0·00 | 0·00 | 0·00 | 0·00 | 1·04e-22 |
| SNCG | 0·02 | 4·06e-18 | 1·43e-16 | 0·02 | 0·00 | 0·01 | 0·02 | 1·49e-05 | 0·00 | 3·73e-26 |
| TFF1 | 0·01 | 0·00 | 0·00 | 0·02 | 0·00 | 0·02 | 0·02 | 0·00 | 0·00 | 1·29e-05 |
| TGFB1 | 0·00 | 3·39e-05 | 0·00 | 0·01 | 0·00 | 0·01 | 0·01 | 1·05e-05 | 0·00 | 7·87e-08 |
| TIMP4 | 0·01 | 6·13e-18 | 2·00e-16 | 0·00 | 0·00 | 0·02 | 0·01 | 2·87e-09 | 2·18e-07 | 2·81e-11 |
| TNFRSF11A | 0·00 | 1·02e-06 | 8·40e-06 | 0·01 | 0·00 | 0·01 | 0·01 | 5·09e-08 | 2·53e-06 | 1·49e-06 |
| TNFRSF11B | 0·01 | 4·14e-22 | 2·46e-20 | 0·01 | 0·00 | 0·00 | 0·01 | 1·41e-08 | 8·31e-07 | 4·39e-34 |
| TNFRSF1A | 0·00 | 2·87e-07 | 2·63e-06 | 0·00 | 0·00 | 0·01 | 0·00 | 5·53e-05 | 0·00 | 1·49e-10 |
| TNFSF11 | -0·02 | 6·58e-13 | 1·48e-11 | -0·01 | 0·00 | 0·02 | -0·02 | 5·72e-09 | 4·11e-07 | 6·07e-19 |
| TNXB | -0·00 | 8·80e-09 | 1·08e-07 | -0·00 | 0·00 | 0·01 | -0·00 | 0·00 | 0·00 | 4·40e-15 |
| TREM2 | 0·02 | 3·21e-20 | 1·49e-18 | 0·02 | 0·00 | 0·00 | 0·02 | 4·41e-07 | 1·46e-05 | 9·96e-31 |
| TSPAN1 | 0·02 | 3·37e-22 | 2·09e-20 | 0·01 | 4·86e-05 | 0·00 | 0·04 | 2·89e-13 | 4·67e-11 | 0·00 |
| VSIG4 | 0·01 | 2·28e-11 | 4·27e-10 | 0·01 | 6·82e-06 | 0·00 | 0·01 | 9·6e-06 | 0·00 | 2·42e-11 |
| WFDC2 | 0·01 | 9·93e-15 | 2·75e-13 | 0·01 | 0·00 | 0·00 | 0·01 | 7·68e-05 | 0·00 | 5·51e-22 |
| WISP2 | 0·02 | 2·88e-20 | 1·39e-18 | 0·01 | 0·00 | 0·01 | 0·02 | 9·25e-11 | 1·09e-08 | 4·02e-32 |
| WNT9A | 0·01 | 4·29e-44 | 1·86e-41 | 0·01 | 4·21e-11 | 1·83e-08 | 0·01 | 5·07e-15 | 1·09e-12 | 6·51e-79 |

## Supplementary Table 7. Importance score calculated by lasso regression model using the relative concentration of 77 proteins that were found to be consistently associated with age in the 200HIV, 2000HIV and 200FG cohort

| Protein | Importance score (100%) |
| --- | --- |
| PODXL2 | 100 |
| TNXB | 96·34 |
| COL1A1 | 74·24 |
| GFAP | 65·27 |
| WNT9A | 63·3 |
| RET | 54·41 |
| SMOC1 | 49·55 |
| BOC | 48·92 |
| CTSV | 42·09 |
| WISP2 | 41·61 |
| ADGRG1 | 41·52 |
| LTBP2 | 34·88 |
| NEFL | 26·52 |
| FGF5 | 26·01 |
| TSPAN1 | 24·68 |
| CXCL17 | 21·71 |
| EBI3_IL27 | 18·6 |
| CCL13 | 13·73 |
| AMBP | 13·69 |
| CDON | 13·55 |
| KIT | 11·48 |
| CXCL14 | 11·22 |
| CRH | 10·72 |
| PROK1 | 9·01 |
| EDA2R | 6·63 |
| HAVCR1 | 6·15 |
| CXCL10 | 5·39 |
| IL6 | 4·93 |
| MOG | 2·2 |
| VSIG4 | 1·84 |
| ADAMTS16 | 0 |
| ADM | 0 |
| CCL11 | 0 |
| CCL2 | 0 |
| CCL3 | 0 |
| CD300E | 0 |
| CD74 | 0 |
| CDCP1 | 0 |
| CGA | 0 |
| CXCL9 | 0 |
| DCN | 0 |
| EFEMP1 | 0 |
| EFNA1 | 0 |
| FAS | 0 |
| FLT3LG | 0 |
| FSTL3 | 0 |
| FUT3_FUT5 | 0 |
| GDF15 | 0 |
| HAVCR2 | 0 |
| HGF | 0 |
| HSPB6 | 0 |
| IGFBP4 | 0 |
| IGFBPL1 | 0 |
| IL17D | 0 |
| KLK4 | 0 |
| LAIR1 | 0 |
| LGALS9 | 0 |
| MLN | 0 |
| MSR1 | 0 |
| NFASC | 0 |
| NTproBNP | 0 |
| OGN | 0 |
| PGF | 0 |
| PIK3IP1 | 0 |
| RSPO3 | 0 |
| SCARB2 | 0 |
| SCARF2 | 0 |
| SNCG | 0 |
| TFF1 | 0 |
| TGFB1 | 0 |
| TIMP4 | 0 |
| TNFRSF11A | 0 |
| TNFRSF11B | 0 |
| TNFRSF1A | 0 |
| TNFSF11 | 0 |
| TREM2 | 0 |
| WFDC2 | 0 |

## Supplementary Table 8. Association results between age advancement and clinical parameters using the 2000HIV cohort (n = 588 samples). A linear regression model was used with chronological age as confounder.

| Outcome | Covariate | Estimate | Std··Error | t·value | Pr(>\|t\|) | FDR |
| --- | --- | --- | --- | --- | --- | --- |
| Age Advancement  Age_Advancement | Sex Birth  Current smoking | 0·15  1·19 | 0·95  0·59 | 0·15  1·99 | 0·87  0·04 | 0·90  0·07 |
| Age_Advancement | BMI | -0·04 | 0·07 | -0·71 | 0·47 | 0·56 |
| Age_Advancement | HIV duration | 0·08 | 0·03 | 2·48 | 0·01 | 0·02 |
| Age_Advancement | CD4 nadir | -1·77 | 1·42 | -1·24 | 0·21 | 0·29 |
| Age_Advancement | ART duration | 0·12 | 0·04 | 2·92 | 0·00 | 0·00 |
| Age_Advancement | CD4 latest | 0·09 | 0·89 | 0·10 | 0·91 | 0·91 |
| Age_Advancement | CD8 latest | 3·40 | 0·62 | 5·43 | 8·81e-08 | 2·29e-06 |
| Age_Advancement | Type 2 Diabetes | 6·05 | 1·36 | 4·45 | 1·01e-05 | 8·82e-05 |
| Age_Advancement | Hypercholesterolemia | 2·55 | 0·61 | 4·19 | 3·21e-05 | 0·00 |
| Age_Advancement | Hypertriglyceridemia | 4·64 | 2·45 | 1·89 | 0·05 | 0·09 |
| Age_Advancement | Hypertension | 2·53 | 0·66 | 3·82 | 0·00 | 0·00 |
| Age_Advancement | Myocardial infarction | 5·40 | 1·50 | 3·59 | 0·00 | 0·00 |
| Age_Advancement | Stroke | 1·34 | 1·70 | 0·78 | 0·43 | 0·53 |
| Age_Advancement | Angina pectoris | 3·80 | 1·74 | 2·17 | 0·03 | 0·05 |
| Age_Advancement | Hepatitis C | 2·27 | 1·01 | 2·23 | 0·02 | 0·04 |
| Age_Advancement | Syphilis | 0·10 | 0·53 | 0·20 | 0·83 | 0·87 |
| Age_Advancement | IMT mean | 1·62 | 2·56 | 0·63 | 0·52 | 0·59 |
| Age_Advancement | Carotid plaque | 1·60 | 0·65 | 2·47 | 0·01 | 0·02 |
| Age_Advancement | Carotid bilateral plaque | 2·42 | 0·87 | 2·75 | 0·00 | 0·01 |
| Age_Advancement | Metabolic syndrome | 3·19 | 0·63 | 5·06 | 5·55e-07 | 7·22e-06 |
| Age_Advancement | Liver Stiffness measurement LSM | 0·60 | 0·19 | 3·16 | 0·00 | 0·00 |
| Age_Advancement | Controled attenuation parameter CAP | 0·00 | 0·00 | 0·58 | 0·55 | 0·60 |
| Age_Advancement | Liver Steatosis | 0·69 | 0·64 | 1·06 | 0·28 | 0·37 |
| Age_Advancement | Liver Fibrosis | 0·88 | 0·54 | 1·62 | 0·10 | 0·15 |
| Age_Advancement | CMV IgG UI_mL | 0·00 | 0·00 | 2·94 | 0·00 | 0·00 |
| Age_Advancement | HDL cholesterol | -3·00 | 0·82 | -3·65 | 0·00 | 0·00 |

## Supplementary Table 9. Association results between age advancement and clinical parameters using the 2000HIV cohort (n = 588 samples). A linear regression model was used with chronological age, sex, and cardiovascular risk factors (type 2 diabetes, hypercholesterolemia, hypertriglyceridemia, and hypertension) as confounders.

| Outcome | Covariate | Estimate | Std··Error | t·value | Pr(>\|t\|) | FDR |
| --- | --- | --- | --- | --- | --- | --- |
| Age_Advancement | Current smoking | 1·06 | 0·58 | 1·83 | 0·06 | 0·13 |
| Age_Advancement | BMI | -0·09 | 0·06 | -1·36 | 0·17 | 0·27 |
| Age_Advancement | HIV duration | 0·06 | 0·03 | 1·83 | 0·06 | 0·13 |
| Age_Advancement | CD4 nadir | -0·95 | 1·39 | -0·68 | 0·49 | 0·67 |
| Age_Advancement | ART duration | 0·08 | 0·04 | 1·90 | 0·05 | 0·13 |
| Age_Advancement | CD4 latest | -0·13 | 0·87 | -0·15 | 0·87 | 0·90 |
| Age_Advancement | CD8 latest | 3·06 | 0·61 | 5·01 | 7·56e-07 | 1·66e-05 |
| Age_Advancement | Myocardial infarction | 3·04 | 1·54 | 1·96 | 0·04 | 0·13 |
| Age_Advancement | Stroke | 0·72 | 1·69 | 0·43 | 0·66 | 0·77 |
| Age_Advancement | Angina pectoris | 2·83 | 1·74 | 1·62 | 0·10 | 0·17 |
| Age_Advancement | Hepatitis C | 2·56 | 0·98 | 2·59 | 0·00 | 0·04 |
| Age_Advancement | Syphilis | 0·31 | 0·53 | 0·59 | 0·55 | 0·67 |
| Age_Advancement | IMT mean | 0·29 | 2·50 | 0·11 | 0·90 | 0·90 |
| Age_Advancement | Carotid plaque | 1·03 | 0·63 | 1·62 | 0·10 | 0·17 |
| Age_Advancement | Carotid bilateral plaque | 1·66 | 0·86 | 1·93 | 0·05 | 0·13 |
| Age_Advancement | Metabolic syndrome | 1·78 | 0·72 | 2·47 | 0·01 | 0·05 |
| Age_Advancement | Liver Stiffness measurement LSM | 0·49 | 0·18 | 2·61 | 0·00 | 0·04 |
| Age_Advancement | Controled attenuation parameter CAP | 0·00 | 0·00 | 0·25 | 0·79 | 0·87 |
| Age_Advancement | Liver Steatosis | 0·39 | 0·63 | 0·61 | 0·53 | 0·67 |
| Age_Advancement | Liver Fibrosis | 0·55 | 0·53 | 1·04 | 0·29 | 0·43 |
| Age_Advancement | CMV IgG UI_mL | 0·00 | 0·00 | 2·78 | 0·00 | 0·04 |
| Age_Advancement | HDL cholesterol | -2·62 | 0·85 | -3·07 | 0·00 | 0·02 |

## Supplementary Table 10. Association results between age advancement and cumulative exposure to drugs using the 2000HIV cohort (n = 588 samples). A linear regression model was used with chronological age as confounder

| Outcome | Covariate | Estimate | Std··Error | t·value | Pr(>\|t\|) | FDR |
| --- | --- | --- | --- | --- | --- | --- |
| Age_Advancement | X3TC_cum | 0·64 | 0·47 | 1·35 | 0·17 | 0·33 |
| Age_Advancement | ABC_cum | 0·99 | 0·61 | 1·63 | 0·10 | 0·25 |
| Age_Advancement | COBI_cum | 0·28 | 0·75 | 0·37 | 0·71 | 0·89 |
| Age_Advancement | DTG_cum | -1·04 | 0·52 | -2·00 | 0·04 | 0·13 |
| Age_Advancement | EFV_cum | -0·72 | 0·63 | -1·14 | 0·25 | 0·39 |
| Age_Advancement | EVG_cum | 1·13 | 0·79 | 1·42 | 0·15 | 0·33 |
| Age_Advancement | FTC_cum | -0·33 | 0·39 | -0·84 | 0·40 | 0·56 |
| Age_Advancement | NVP_cum | -0·02 | 0·69 | -0·03 | 0·97 | 0·97 |
| Age_Advancement | RPV_cum | -1·67 | 0·71 | -2·34 | 0·02 | 0·07 |
| Age_Advancement | RTV_cum | 2·20 | 0·60 | 3·61 | 0·00 | 0·00 |
| Age_Advancement | TAF_cum | 0·64 | 0·56 | 1·14 | 0·25 | 0·39 |
| Age_Advancement | TDF_cum | -0·13 | 0·39 | -0·33 | 0·73 | 0·89 |
| Age_Advancement | ZDV_cum | 1·90 | 0·80 | 2·36 | 0·02 | 0·07 |
| Age_Advancement | PI_cum | 1·62 | 0·62 | 2·60 | 0·01 | 0·05 |
| Age_Advancement | INSTI_cum | 0·07 | 0·44 | 0·16 | 0·86 | 0·92 |
| Age_Advancement | booster_cum | 1·47 | 0·50 | 2·90 | 0·00 | 0·03 |
| Age_Advancement | NNRTI_cum | 0·10 | 0·45 | 0·23 | 0·81 | 0·92 |

## Supplementary Table 11. Association results between age advancement and SASP cytokines produced by PBMCs upon 24-hour stimulation with various stimuli using the 2000HIV cohort (n = 588 samples). A linear regression model was used with chronological age and sex as confounders.

| Outcome | Covariate | Estimate | Std· Error | t value | Pr(>\|t\|) | FDR |
| --- | --- | --- | --- | --- | --- | --- |
| Age_Advancement | IL1b_CMV | 0·20 | 0·28 | 0·71 | 0·47 | 0·61 |
| Age_Advancement | IL1b_Spneu | 0·75 | 0·27 | 2·72 | 0·00 | 0·03 |
| Age_Advancement | IL1b_LPS | 0·50 | 0·27 | 1·82 | 0·06 | 0·16 |
| Age_Advancement | IL1b_IMQ | 1·19 | 0·27 | 4·36 | 1·51e-05 | 0·00 |
| Age_Advancement | IL1b_HIVENV | 1·00 | 0·28 | 3·59 | 0·00 | 0·00 |
| Age_Advancement | IL6_Spneu | 0·57 | 0·27 | 2·08 | 0·03 | 0·14 |
| Age_Advancement | IL6_LPS | -0·11 | 0·28 | -0·41 | 0·67 | 0·72 |
| Age_Advancement | IL8_CMV | 0·15 | 0·28 | 0·55 | 0·58 | 0·66 |
| Age_Advancement | IL8_Spneu | 0·15 | 0·27 | 0·55 | 0·57 | 0·66 |
| Age_Advancement | IL8_LPS | 0·42 | 0·27 | 1·51 | 0·13 | 0·23 |
| Age_Advancement | IL8_IMQ | 0·93 | 0·29 | 3·16 | 0·00 | 0·01 |
| Age_Advancement | IL8_HIVENV | 0·50 | 0·27 | 1·80 | 0·07 | 0·16 |
| Age_Advancement | IL8_IL1a | -0·11 | 0·29 | -0·39 | 0·69 | 0·72 |
| Age_Advancement | MCP1_CMV | 0·40 | 0·27 | 1·45 | 0·14 | 0·24 |
| Age_Advancement | MCP1_Spneu | 0·80 | 0·28 | 2·84 | 0·00 | 0·02 |
| Age_Advancement | MCP1_LPS | 0·50 | 0·27 | 1·79 | 0·07 | 0·16 |
| Age_Advancement | MCP1_IMQ | 0·46 | 0·29 | 1·60 | 0·10 | 0·22 |
| Age_Advancement | MCP1_HIVENV | 0·43 | 0·28 | 1·53 | 0·12 | 0·23 |
| Age_Advancement | MCP1_PolyIC | 0·53 | 0·28 | 1·84 | 0·06 | 0·16 |
| Age_Advancement | MCP1_IL1a | 0·03 | 0·28 | 0·13 | 0·89 | 0·89 |
| Age_Advancement | MIP1a_CMV | 0·30 | 0·28 | 1·06 | 0·28 | 0·42 |
| Age_Advancement | MIP1a_Spneu | 0·28 | 0·28 | 1·01 | 0·30 | 0·42 |
| Age_Advancement | MIP1a_LPS | 0·27 | 0·27 | 1·00 | 0·31 | 0·42 |

## Supplementary Table 12. Association results between age advancement and T cell-secreted cytokines upon 7-day stimulation with various stimuli using the 2000HIV cohort (n = 588 samples). A linear regression model was used with chronological age and sex as confounders.

| Outcome | Covariate | Estimate | Std· Error | t value | Pr(>\|t\|) | FDR |
| --- | --- | --- | --- | --- | --- | --- |
| Age_Advancement | S·aureus_IFNy | -0·29 | 0·28 | -1·04 | 0·29 | 0·59 |
| Age_Advancement | S·pneu_IFNy | -0·65 | 0·28 | -2·30 | 0·02 | 0·06 |
| Age_Advancement | C·alb·con_IL5 | -1·02 | 0·27 | -3·71 | 0·00 | 0·00 |
| Age_Advancement | MTB_IL5 | 0·15 | 0·35 | 0·43 | 0·66 | 0·87 |
| Age_Advancement | PHA_IL5 | 0·89 | 0·27 | 3·21 | 0·00 | 0·01 |
| Age_Advancement | S·pneu_IL5 | -0·66 | 0·35 | -1·89 | 0·05 | 0·13 |
| Age_Advancement | C·alb·con_IL10 | -0·09 | 0·28 | -0·33 | 0·73 | 0·87 |
| Age_Advancement | E·coli_IL10 | -0·24 | 0·27 | -0·89 | 0·37 | 0·69 |
| Age_Advancement | MTB_IL10 | -0·04 | 0·28 | -0·14 | 0·88 | 0·91 |
| Age_Advancement | PHA_IL10 | -0·65 | 0·27 | -2·34 | 0·01 | 0·06 |
| Age_Advancement | S·aureus_IL10 | -0·09 | 0·27 | -0·35 | 0·72 | 0·87 |
| Age_Advancement | S·pneu_IL10 | 0·19 | 0·28 | 0·69 | 0·48 | 0·79 |
| Age_Advancement | C·alb·con_IL17 | -0·57 | 0·29 | -1·91 | 0·05 | 0·13 |
| Age_Advancement | E·coli_IL17 | -0·11 | 0·36 | -0·31 | 0·75 | 0·87 |
| Age_Advancement | MTB_IL17 | 0·08 | 0·34 | 0·25 | 0·80 | 0·89 |
| Age_Advancement | PHA_IL17 | -0·77 | 0·30 | -2·54 | 0·01 | 0·05 |
| Age_Advancement | S·aureus_IL17 | -0·19 | 0·30 | -0·64 | 0·51 | 0·80 |
| Age_Advancement | S·pneu_IL17 | 0·05 | 0·30 | 0·16 | 0·86 | 0·91 |
| Age_Advancement | C·alb·con_IL22 | -0·50 | 0·29 | -1·72 | 0·08 | 0·18 |
| Age_Advancement | E·coli_IL22 | -0·10 | 0·30 | -0·35 | 0·72 | 0·87 |
| Age_Advancement | MTB_IL22 | -0·94 | 0·29 | -3·21 | 0·00 | 0·01 |
| Age_Advancement | PHA_IL22 | -0·21 | 0·28 | -0·74 | 0·45 | 0·79 |
| Age_Advancement | S·aureus_IL22 | -0·15 | 0·28 | -0·52 | 0·59 | 0·87 |
| Age_Advancement | S·pneu_IL22 | -0·65 | 0·29 | -2·21 | 0·02 | 0·07 |
| Age_Advancement | C·alb·con_IFNy | -0·73 | 0·30 | -2·39 | 0·01 | 0·06 |
| Age_Advancement | E·coli_IFNy | -0·80 | 0·29 | -2·78 | 0·00 | 0·03 |
| Age_Advancement | MTB_IFNy | -0·72 | 0·28 | -2·55 | 0·01 | 0·05 |
| Age_Advancement | PHA_IFNy | 0·01 | 0·28 | 0·06 | 0·94 | 0·94 |
